# Supplementary material for: Effectiveness and cost-effectiveness of a 12-month automated text message intervention for weight management in postpartum women with overweight or obesity: protocol for the Supporting MumS (SMS) multisite, parallel-group, randomised controlled trial
Source: BMJ Open. 2024 May 6;14(5):e084075. doi: 10.1136/bmjopen-2024-084075 (PMC11086389; doi:10.1136/bmjopen-2024-084075)
Supplement: Supplementary data [file bmjopen-2024-084075supp001.pdf]

Supplemental appendix 1- SMS Logic Model and sample text messages

a) Supporting MumS intervention logic model

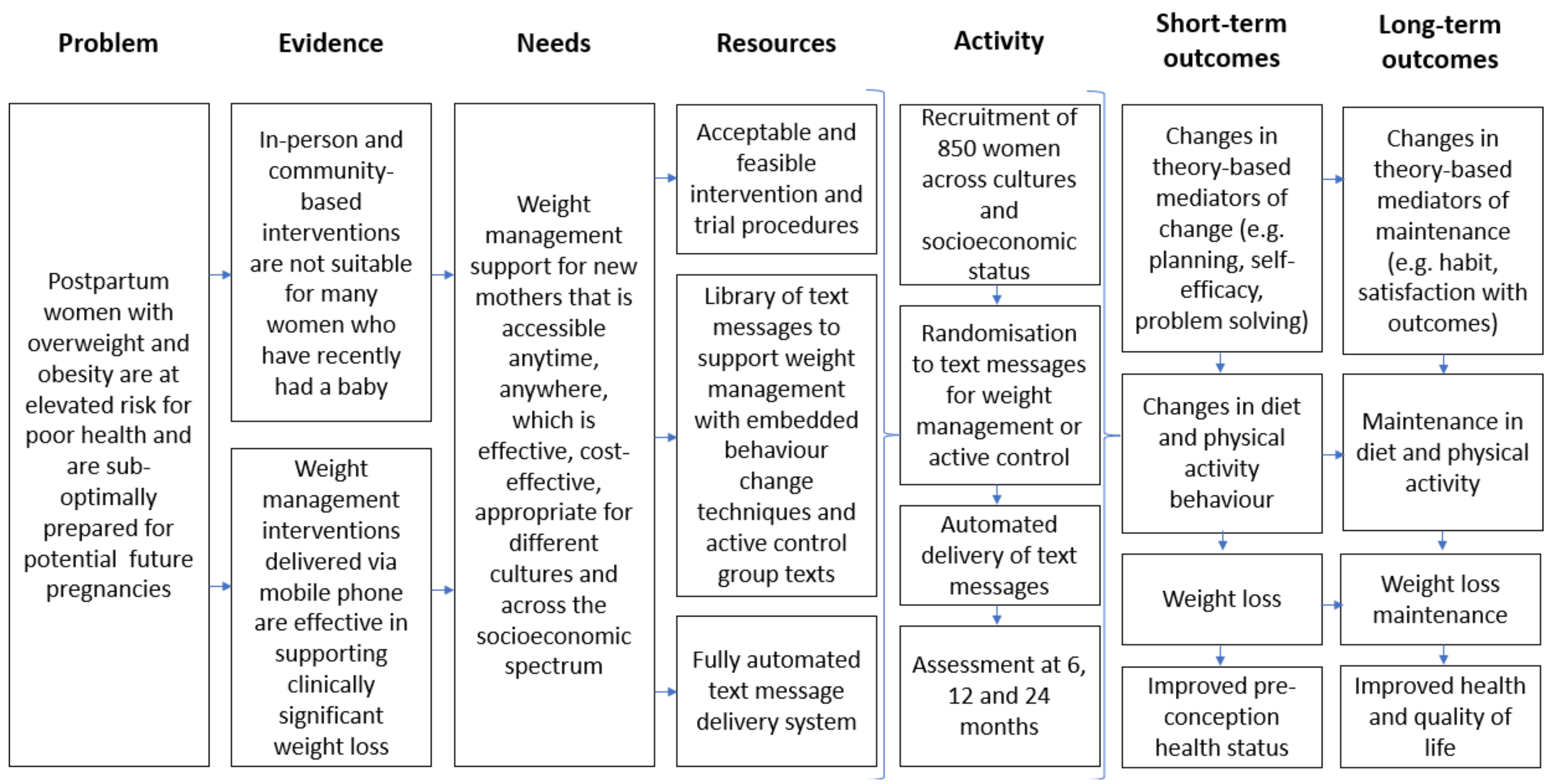

4 **b) Supporting MumS sample text messages**

Type of text message		Example of message sent	Example of automated responses (if applicable)
**Core intervention text message**		*'Another tip for success: Keep track of what you eat. People who regularly monitor what they eat are more likely to meet their weight loss target. Stay tuned for more texts on food tracking'.*	
**Weekly self-weighing reminder**	**Months 0-6**	*'What are the scales saying this week? Text us e.g. 11 st 5, or 73.3 kg'.*	N/A
	**Months 7-12**	*'Weekly weighing = your very own early warning system! Step on the scales today and text us your weight followed by UP, DOWN, SAME. e.g. "10 st 7 SAME" or "66.7 kg SAME'.*	Up: *'Don't give up! The best thing to do is prevent these small regains from turning into bigger regains. What could you do this week? E.g. maybe exercise a little more or be more mindful of portion sizes?'*
			Down: *'Success! Your efforts are paying off! All the hard work is worth it :)'.*

			Same: *'Maintaining your weight is something to be proud of! Treat yourself to a reward e.g. a relaxing bath or simply 10 minutes to yourself!'*
**Yes/No question**		'*WEEK 14. Have you set your activity goal for the week ahead? Text Y11 for yes or N11 for no'*.	Y11 (yes): '*Great job, setting goals is clearly becoming one of your weekly habits :) Alongside your activity goal, set a food goal too and you're onto a double winner!'*
			N11 (no): '*New to the whole activity thing? Set a goal to do a small amount of an activity that you enjoy - for example, a brisk 15-minute walk or a zumba routine when your little one goes to sleep. It will help motivate you and give you a boost of confidence to do more next time'*.

**Trigger words**	*'You can always text us back for more help – text us with one of these trigger words and texts with instant support will appear on your phone: 'BAD DAY', 'SLIP-UP', 'TIRED' and 'CRAVE'.*	Bad day*: 'Everyone has these type of days. Don't be hard on yourself! Press delete for the day so far and start afresh!'*
		Slip-up*: 'Don't let one slip-up discourage you. You have the ability to pick up right where you left off!'*
		Tired response: '*Fresh air can work wonders! Getting out of the house for a while can help you think more clearly and boost your energy'.*
		Crave response: *'Tried brushing your teeth or chewing a piece of sugar free gum? It might work!'*
**Weight management when breastfeeding**	*'Breastfeeding can make you hungry. Just fill up on the good stuff (fruit and vegetables, wholegrain cereals) and the good eating habits will stay with you even when the breastfeeding stops :) '*	N/A

**Smoking cessation and weight management**	*'It is only normal to have cravings! Why not exercise to combat it? Try a short walk (even for just 5 mins) if a craving hits, the distraction can really help.'*	N/A
**Intervention "buddy system"**	*'Welcome to WEEK 34. Many people that have lost weight and kept it off have done so with support from family and friends! You can opt in to get your friend/partner/family member to receive SMS texts for extra support - text 'SUPPORT' followed by the person's mobile number to sign up (ask their permission first). E.g. Support 0780000000'.*	N/A
**Active control**	*'1, 2, 3! Your toddler might know how to count to 3 or more...or they might get some of the numbers mixed up. Whatever stage they are at, just keep encouraging them by counting from 1 to 10'.*	N/A

Supplemental appendix 2- Informed consent materials [32]

a) Participant Information Sheet

PARTICIPANT INFORMATION SHEET

The Supporting MumS (SMS) study

We would like to invite you to take part in this research study which is taking place in Northern Ireland, England, Scotland and Wales.

Before you decide whether to take part, it is important that you understand why we are doing this research and what it will involve.

Please take time to read the information carefully and discuss it with friends/relatives, if you wish. If you have any questions, please contact us using the details given at the end of the sheet.

Gallagher D, *et al. BMJ Open* 2024; 14:e084075. doi: 10.1136/bmjopen-2024-084075

Why are we doing this study?

Welcoming a baby brings lots of joy and happiness but being a new mum also means life is a bit busy! There may be areas where mums could benefit from more support. Mums often talk about having a baby as a time when their weight started to creep up and how they would welcome support with this. We are interested in finding out if text messages can help support weight management in the first two years after having a baby.

For this study, the term 'mum' or 'mother' includes people who do not identify as women but are pregnant or have given birth. The term 'your child' means your youngest child at the time of signing up to the study.

Why have I been asked to take part?

You have been chosen to take part, as you are over 18 years old and have had a baby within the past two years and have a body mass index over 25kg/m^2.

Do I have to take part?

No, it is up to you whether or not you want to take part. If you do decide to take part, you are free to leave the study at any stage without giving a reason for doing so. Your usual health care will not be affected at any time.

What will happen if I take part?

After you read this leaflet we will contact you to discuss the study in more detail and answer any further questions you may have.

If you decide to take part, you will be asked to sign the consent form for the study and complete some study measurements (described below). You will then receive text messages to your mobile phone for 12 months. The number of text messages you receive each week will vary between 3 and 14 per week. The text messages are delivered to you by a secure text message system owned and operated by the London School of Hygiene and Tropical Medicine.

Mums who take part in the study will get text messages on **ONE** of the two topics shown below. It is important that you understand that the topic is randomly chosen for you and that there is a 50:50 chance you will get texts on either topic 1 or topic 2:

1. Information and advice on making healthy food choices and keeping physically active to help you lose weight.

OR

2. Information and advice on your child's health and development.

What information will you collect from me if I take part?

We would meet with you 4 times (during a 24 month period) to collect some measurements and information. These visits will take place at the start of the study, at 6 months, at 12 months (when the text messages stop) and at 24 months (12 months after you stopped receiving the text messages).

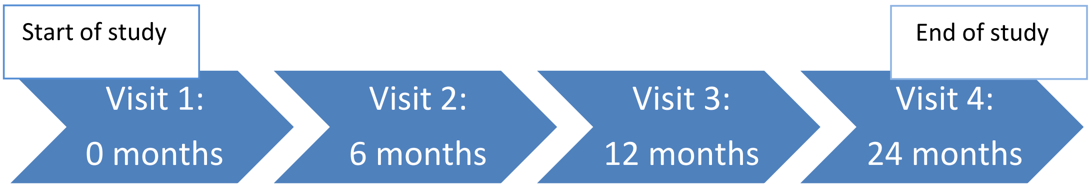

For the visits, the researcher will either visit you at your home or you can come and meet the researcher at a convenient location such as a University building or a local community venue; whichever is most convenient for you.

You will receive a £25 voucher on completion of each visit in recognition of your time to complete the research measures. This will be a total of £100 over the 24-month study, if all visits are completed. Each visit will last approximately 1 hour.

At each visit we will collect the following information:

a) We will measure your height (only at visit 1) and will ask you to step on the scales to collect information on weight and will measure your waist circumference (this will take about 10 minutes).
b) We will ask you to complete a questionnaire booklet about your physical and mental health, lifestyle and wellbeing. The booklet will take approximately 35 minutes to complete. You will have the option of:
    - completing a printed questionnaire booklet in your own time and posting it back to us in a stamped addressed envelope we provide; OR
    - completing the questionnaire online by accessing a link we give you; OR
    - completing the questionnaire with the researcher at the study visit or over the phone.

When (a) and (b) above has been collected from you, we will arrange for the £25 voucher to be sent to you.

We would also ask you to consider taking part in a short (approximately 20 minutes) telephone interview twice during the study (at the 6 month visit and the 12 month visit) to help us understand how you found the text messages. *This part is optional.*

Gallagher D, *et al. BMJ Open* 2024; 14:e084075. doi: 10.1136/bmjopen-2024-084075

Linking to routine health-related data in the future (this part is optional)

We would also ask you to consider allowing us to collect health-related data about you and your child in the future. We would do this by linking with organisations that collect data about the services they provide to women and children. For example, the NHS keeps records, known as 'routine data' about how we use different services such as the health service.

Routine data is stored electronically on different systems. To obtain your routine data from other systems, we would share some personally-identifiable details such as name, date of birth, address and NHS number to the organisations that manage these systems and they will send us data back. This will all be done using secure data transfer systems that have been set up carefully to keep your information safe.

Doing this helps us to understand if a study like this has any longer-term benefits and how we might improve health services in the future.

We may access routine data from these sources:

- Health records such as GP and dental records, maternity and health visiting records and disease registers
- Local authority and social care
- Family/children's centres
- Education or school
- National child measurement programme
- Voluntary organisations

For example, from maternity records we may collect information on any further pregnancies you have and from GP records we may collect information on any diagnosed conditions such as high blood pressure or type 2 diabetes.

If you consent to us linking to routine health-related data on the consent form, we will ask you for your NHS number. This number will only be stored by QUB for 15 years and will only be used for this purpose. It will be stored securely, separately from your other data, in a password protected, encrypted file on computers that are only accessible to authorised members of the research team. If at any time you want us to remove your NHS number from our records, you can do so by contacting us as per details given at the end of this information sheet. Otherwise, it will be removed from our records after 15 years. An example of how we would link your routine data is shown below.

You do not have to consent to data linkage to participate in this study. This part is optional.

BMJ Publishing Group Limited (BMJ) disclaims all liability and responsibility arising from any reliance placed on this supplemental material which has been supplied by the author(s)

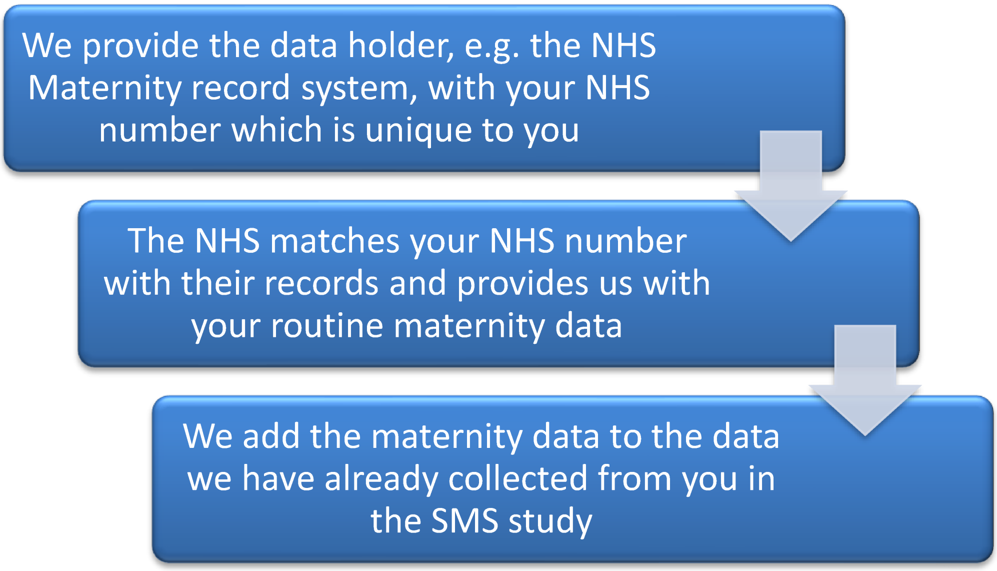

Example of routine data linkage process

What are the possible advantages and disadvantages of taking part?

By taking part in this research you will be helping us find the best ways to support new mums. In the study questionnaires and interviews, we will collect information about your postnatal physical and mental health. This information will be used to help us understand how we might improve services for women after they have a baby. Some of the messages you receive may be helpful for you and/or your baby by helping you to lose weight or by providing you with information about child health and development. We do not anticipate any risks from taking part in this study.

How will we use information about you?

We will need to use information from you for this research project. This information will include your name, address, NHS number, date of birth, telephone number, email address and date of birth of your youngest child. People will use this information to do the research or to check your records to make sure that the research is being done properly.

People who do not need to know who you are will not be able to see your name or contact details. Your data will have a code number instead.

We will keep all information about you safe and secure.

Gallagher D, *et al. BMJ Open* 2024; 14:e084075. doi: 10.1136/bmjopen-2024-084075

Once we have finished the study, we will keep some of the data so we can check the results. We will write our reports in a way that no-one can work out that you took part in the study.

Will my taking part in the study be kept confidential?

Yes, all your data will be treated with the strictest confidence and your details will not be shared with anybody outside of the research team, unless there is a serious risk of harm to you or others.

Any information collected from you will be stored securely on password protected files on password protected computers that only the research team can access. Hard copies of documents will be kept in locked filing cabinets in locked offices that are only accessible to the research team and are located in a building that is locked outside normal working hours.

Any interviews conducted over the telephone will be audio-recorded and the recording will then be typed up for research purposes. The typed transcript will not contain any names and will be labelled with your unique study number. The recording will be destroyed once the typed transcript is prepared.

Study data will be kept separate from personal information (such as name and address). Only members of the research team will have access to view identifiable data. However, in some instances, inspectors from regulatory authorities may need to access data for checking the quality of the research. All members of the research team and regulatory bodies are trained in data protection and will comply with the requirements of data protection legislation.

Once the study is complete and it is no longer necessary to keep identifiable information or contact details, we will destroy our records of this personal information.

The study questionnaire asks some questions about mental and physical health. If we have any concerns that your responses to such questions may indicate you are at risk of postpartum depression, we will inform you and your GP by letter as part of our duty of care to you. If at any stage we have concerns that you, or someone else, is at risk of harm then we are obliged to tell the relevant services, for example your GP or social services.

What are your choices about how your information is used?

- You can stop being part of the study at any time, without giving a reason, but we will keep information about you that we already have.

- We need to manage your records in specific ways for the research to be reliable. This means that we won't be able to let you see or change the data we hold about you.
- If you agree to take part in this study, you will have the option to take part in future research using your data saved from this study.

Where can you find out more about how your information is used?

You can find out more about how we use your information:

- at www.hra.nhs.uk/information-about-patients/
- in our privacy notice from go.qub.ac.uk/SMSstudy or ask the research team for a copy
- by asking one of the research team
- by sending an email to info.compliance@qub.ac.uk, or
- by ringing us on 07341 888415.

How will I find out the results of this project?

When the study is finished and the study information has been analysed, we will send you a summary of the results.

Who is organising and funding the research?

For this study we will be recruiting women from all four countries in the UK. The work is being led by Professor Michelle McKinley from Queen's University Belfast. Researchers from Universities in London, Cardiff, Stirling and the Bradford Teaching Hospitals NHS Foundation Trust are leading recruitment in their areas.

Mums have helped us design our text messages and advise on how the study is conducted and will continue to do so.

The study is funded by National Institute of Health Research and the Public Health Agency Northern Ireland.

Has this study been approved for safety by an ethics committee? Is this study safe?

This study has been reviewed and approved by the West of Scotland Research Ethics Committee 4 and IRAS ID 305557.

What will happen if I don't want to carry on with the study?

You can withdraw from the study at any time, without giving a reason by contacting the research team (contact details at the end of this leaflet). If you do decide to withdraw from the study, we will use the data collected up to that point, but we will not collect any more data.

What if something goes wrong?

There are no special compensation arrangements. Queen's University Belfast will provide indemnity for this study. If you are harmed due to someone's negligence, then you may have grounds for legal action but you may have to pay for it.

What if there is a problem?

If you have a concern about any aspect of this study, you can speak to the researchers who will do their best to answer your questions (contact details on the last page).

Should you remain unhappy and wish to make a formal complaint, you can contact:
Research Governance Team at Queen's University Belfast,
63 University Road, Belfast
BT7 1NF
(Telephone: 028 9097 2529; Email: researchgovernance@qub.ac.uk).

Contact for Further Information:

Researchers' Name, Address, Email address, Telephone (site specific)	Dr Dunla Gallagher<br>SMS Trial Manager<br>Centre for Public Health<br>Queen's University Belfast<br>School of Medicine, Dentistry & Biomedical Sciences<br>Institute of Clinical Sciences (Block B)<br>Grosvenor Road<br>Belfast, N.Ireland<br>BT12 6BA<br>Tel: 07341 888415<br>Email: d.gallagher@qub.ac.uk

Please ask us if there is anything that is not clear or if you would like more information.

b) Privacy Notice

PRIVACY NOTICE

The Supporting MumS (SMS) study

Queen's University Belfast, Bradford Teaching Hospitals NHS Foundation Trust, Cardiff University, the University of Stirling, and the London School of Hygiene & Tropical Medicine ("we", "us" and "our") are committed to protecting your personal data.

The notice is addressed to individuals who are taking part in the research study entitled 'The Supporting MumS (SMS) study' ("you" and "your").

This Privacy Notice tells you why we need to collect personal information about you, what we will do with it, and how we will look after it. It also tells you about your legal rights in relation to your Personal Data.

If you have any questions about this privacy notice, please contact us. Contact details are provided below.

WHO WE ARE

1. We are a group of expert researchers from the organisations listed above. The study is led by Queen's University Belfast and is funded by National Institute of Health Research and the Public Health Agency Northern Ireland.

2. All our research is underpinned by policies and procedures that ensure we comply with regulations and legislation that govern the conduct of research. This includes data protection legislation: the UK General Data Protection Regulation (GDPR) and the Data Protection Act 2018 (DPA). In the case of health and social care research, which should serve the public interest, where we have to demonstrate that our research serves the interests of society as a whole.

HOW YOUR PERSONAL DATA IS COLLECTED

3. **Information you provide:** When you consent to take part in the SMS study we will ask for information about you, such as your name, address, NHS number, date of birth, telephone number, email address and date of birth of your youngest child. This is known as your "Personal Data". We may also ask you for some special categories of information (for example information about your physical and mental health). This is known as your "Sensitive Personal Data" and has additional protections.

4. **Data from other sources:** We will also collect information about you from other sources and this also forms part of "Personal Data" and "Sensitive Personal Data". This includes information from:

- the system we are using to send and receive text messages for the study, for example we will automatically collect the replies you send to the text messages. This system is owned and operated by the London School of Hygiene & Tropical Medicine.
- routine health-related data (if you have consented to this option) such as NHS records, GP and dental records, maternity and health visiting records and disease registers. To obtain your routine data from these systems, we will share some personally-identifiable details such as name, date of birth, address and NHS number to each of these organisations and they will send us your data back. This will all be done using secure data transfer systems that have been set up carefully to keep your information safe and complies with regulations and legislation governing the conduct of research.

HOW WE USE YOUR PERSONAL DATA

5. We use your Personal Data and Sensitive Personal Data in the following ways:
   - to contact you to invite you to take part in the study and to discuss research-related queries. If you do not consent to take part in the study, your personal data, such as contact details (e.g. name, address, phone number), will be confidentially destroyed and will not be retained by us.
   - after consent to take part is provided, personal data is used to contact you to arrange and conduct study visits, to request information or to send you relevant study documents or equipment and to inform you about any relevant study developments.
   - access to, and sharing of, special category and sensitive personal data is controlled very carefully. Sensitive personal data is collected and pseudonymised – this means each person who takes part in this study will be given a unique study number and this unique study number is used on all the study paperwork and in study databases. Only authorised members of the SMS research team will be able to link your study number to your name, contact details and NHS number.

6. In order to protect your rights and freedoms when using your personal information for research and to process special category information we have special safeguards in place to protect your information, and all information is kept in line with our policies and regulatory requirements.

7. In addition to the above safeguards, data protection legislation requires us to meet the following standards when we conduct research with your personal information:
   - the research will not cause damage or distress to someone (e.g., physical harm, financial loss or psychological pain).
   - the research is not carried out in order to do or decide something in relation to an individual person, unless the processing is for medical research approved by a research ethics committee.
   - the Data Controller has technical and organisational safeguards in place (e.g. appropriate staff training and security measures).
   - if processing a special category of data, this must be subject to a further public interest test to make sure this particularly sensitive information is required to meet the research objectives.

LEGAL BASIS FOR COLLECTING AND USING YOUR PERSONAL DATA

8. We will only use your Personal Data if we have valid reasons for doing so. These reasons are known as our "legal basis for processing".

9. In the context of research, the lawful basis upon which we will process your personal information is usually where "Processing is necessary for the performance of a task carried out in the public interest or in the exercise of official authority vested in the controller" (Article 6 of GDPR).

10. We also collect and use more sensitive personal information (Special Category data) where "the processing is necessary for archiving purposes in the public interest, scientific or historical research purposes or statistical purposes… which shall be proportionate to the aim pursued, respect the essence of the right to data protection and provide for suitable and specific measures to safeguard the fundamental rights and the interests of the data subject". (Article 9 of GDPR).

WHO WE SHARE YOUR DATA WITH

11. In line with our Data Protection Policy and Procedures we can share your information, including Personal Data and Sensitive Personal Data, with the following parties for the following research purposes:
    - London School of Hygiene and Tropical Medicine (LSHTM) text message system – to allow you to receive and reply to the text messages (the LSHTM is a study collaborator)
    - Designated research personnel for the purposes of achieving the research outcomes
    - The Northern Ireland Clinical Trials Unit (NICTU) for the purposes of entering your completed questionnaires onto a study database (NICTU is a study collaborator)
    - Qualtrics research software supplier for the purposes of the online completion of the study questionnaires (privacy notice can be viewed here - https://www.qualtrics.com/support/survey-platform/getting-started/data-protection-privacy/).
    - Transcription services for the purpose of transcribing recorded interviews (if consent provided for this optional element)
    - Organisations in-charge of the collection and administration of routine data for the purposes of achieving the research outcomes (if consent provided for this optional element)

12. In all cases, information shared will be on a need to know basis, not excessive and with all appropriate safeguards in place to ensure the security of your information.

13. When we use third parties (known as data processors) to carry out a task on our behalf, such as a transcription service as indicated above, we have contractual terms, policies and procedures to ensure confidentiality is respected.

DATA PROCESSING OUTSIDE EUROPE

14. We will not transfer your Personal Data and Sensitive Personal Data outside of the United Kingdom and European Economic Area.

HOW LONG YOUR INFORMATION WILL BE KEPT?

15. Information where you can be identified will be kept for a minimum amount of time and in accordance with research objectives. Researchers will de-identify information, i.e. anonymise or pseudonymise, as soon as possible as described in point 5.

16. We will keep your Personal Data and Sensitive Personal Data for up to 10 years from when the research has been completed to allow for full and final publication of the research results. If you have provided consent (this is optional), we will retain your record of consent and personal data (name, telephone number, email address, address) for the purpose of conducting longer-term follow-up for the SMS study or to inform you about future studies related to health and lifestyle that we may be conducting. If you have consented, we will retain your NHS number for data linkage purposes. You can decline to receive communications at any stage. We will only keep your information if we need it for one of the reasons described above.

17. We place great importance on the security of the Personal Data that we hold, including the use of physical, technological and organisational measures to ensure your information is protected from unauthorised access and against unlawful processing, accidental loss, alteration, disclosure, destruction and damage.

YOUR RIGHTS

18. The DPA provides you with a number of legal rights in relation to your Personal Data, including the right:

- to request access to your Personal Data;
- to request correction of your Personal Data that is wrong or incomplete;
- to request erasure or the restriction of processing of your Personal Data;
- to request the transfer of your Personal Data in a structured; commonly used machine-readable format;
- not to be subject to automated decision making; and
- to withdraw your consent.

19. If you wish to exercise any of the rights set out above, or require further information about any of the rights, please contact us.

20. There may also be times where we cannot stop using your Personal Data when you ask us to, but we will tell you about this if you make a request.

CONTACTING US

21. If you have any questions or comments about this privacy notice, please contact the SMS team:

Dr Dunla Gallagher
SMS Trial Manager
Centre for Public Health
Institute Clinical Science A
School of Medicine, Dentistry & Biomedical Sciences
Queen’s University Belfast
d.gallagher@qub.ac.uk

Professor Michelle McKinley
SMS Chief Investigator
Centre for Public Health
Institute Clinical Science A
School of Medicine, Dentistry & Biomedical Sciences
Queen’s University Belfast
m.mckinley@qub.ac.uk

Alternatively, you can contact:
Data Protection Officer
Information Compliance Unit
Lanyon South
Queen’s University Belfast
University Road
BT7 1NN
Info.compliance@qub.ac.uk

COMPLAINTS

22. You have the right to complain about how we treat your Personal Data and Sensitive Personal Data to the Information Commissioner’s Office (ICO). The ICO can be contacted at:

    Information Commissioner's Office
    Wycliffe House
    Water Lane
    Wilmslow
    Cheshire
    SK9 5AF

CHANGES TO THIS NOTICE

23. We may update this Privacy Notice from time to time. We will notify you of the changes where we are required by law to do so.

c) Study Consent Form

CONSENT FORM
The Supporting MumS (SMS) study

Screening Number: __ __ __ __ __ Participant ID number: __ __ __ __ __

		Please **initial** box
1.	I confirm that I have read and understood the Participant Information Sheet dated 05/04/2022 (Version 4.0) and privacy notice dated 30/11/2021 (Version 1.0) for the above study and have been given copies to keep.	
2.	I have had the opportunity to consider the information, ask questions and have had these answered to my satisfaction.	
3.	I understand that my participation is voluntary and that I am free to withdraw at any time, without giving any reason and without my medical care or legal rights being affected.	
4.	I understand that information collected about me for the study (including personally identifiable information) may be looked at by responsible individuals in the study team and regulatory authorities supervising the study.	
5.	I understand that all information collected about me during the course of the research will be kept strictly confidential and processed in compliance with data protection legislation.	
6.	I understand that some of the data collected about me will be processed by third parties (known as data processors), such as software and transcription companies, and that contractual terms, policies and procedures will be put in place to ensure confidentiality is respected.	
7.	I give permission for my GP to be informed of any concerning medical issues detected during study visits.	
8.	I am aware of the potential risks and benefits of this research study, as described in the Participant Information Sheet and discussed with the researcher.	
9.	I understand that I will not be identifiable in any published report using data from this study.	
10.	I agree to my contact details being kept so I can be informed of the study findings.	
**11.**	**I consent to take part in the above study.**	

	**OPTIONAL ELEMENTS (please initial box if you consent)**	
12.	I am willing to be contacted to take part in two short (20-30 minute) telephone interviews during the study to provide feedback to the research team.	☐
13.	I am willing to be contacted in the future about a follow-up to this study.	☐
14.	I consent to be contacted by the University about future research studies related to diet or lifestyle and health for which I may be eligible.	☐
15.	I consent to my personal health information, including my NHS number and date of birth, being released to organisations that hold routine health-related data so they can locate information about me that is held in their database.	☐
16.	I consent to organisations holding my routine health-related data performing data linkage and releasing the information about me from its databases to the SMS Study research team.	☐

Name of Participant (Block capitals)	Signature	Date

To be completed by the Principal Investigator or nominee

I, the undersigned, have taken the time to fully explain to the above patient the nature and purpose of the study in a way that they could understand. I have explained the risks involved as well as the possible benefits. I have invited them to ask questions on any aspect of the study that concerned them.

Name of Researcher (Block capitals)	Signature	Date

Researchers' Name, Address, Email address, Telephone (site specific)	Dr Dunla Gallagher<br>SMS Trial Manager<br>Centre for Public Health, Queen's University Belfast<br>School of Medicine, Dentistry & Biomedical Sciences<br>Institute of Clinical Sciences (Block B)<br>Grosvenor Road, Belfast, N.Ireland, BT12 6BA<br>Tel: 07341 888415<br>Email: d.gallagher@qub.ac.uk

BMJ Publishing Group Limited (BMJ) disclaims all liability and responsibility arising from any reliance placed on this supplemental material which has been supplied by the author(s)

Supplemental appendix 3- Participant self-report questionnaires (baseline and 12 months)

Supporting MumS (SMS) study

Questionnaire Booklet

Month 0

**Screening ID:**	_ _ _ _ _
**Participant ID:**	_ _ _ _ _
**Researcher name:**	
**Today's date:**	_ _ / _ _ / _ _ _ _<br>**e.g. 05 / 01 / 2017**

About this questionnaire booklet

Thank you for taking the time to fill out this questionnaire booklet.

The questionnaire booklet is made up of questions that will help us find out more about your experience of the postpartum period. Please complete the questions as accurately as you can. **There are no right or wrong answers**. If there are any questions you do not wish to answer, you can leave them blank. Note that your responses will be **anonymous,** this means your name will never be given with your responses.

The questionnaire booklet is split up into the short sections shown below. You can fill it all out at once, or do it in chunks, as your time allows.

**Section 1**	**Health and well-being**	**Pages 3-5**
**Section 2**	**Health resources**	**Pages 6-8**
**Section 3**	**Eating and activity approaches**	**Pages 9-13**
**Section 4**	**Social support**	**Page 14-15**
**Section 5**	**Lifestyle behaviours and attitudes**	**Page 16-18**
**Section 6**	**Food patterns**	**Page 19-23**
**Section 7**	**Physical activity**	**Page 24-26**
**Section 8**	**Infant feeding**	**Page 27-28**
**Section 9**	**Mood**	**Page 29-33**
**Section 10**	**Self-esteem**	**Page 34**
**Section 11**	**Sleep**	**Page 35-38**

Section 1: Health and well-being

For questions 1-5, under each heading, please tick (✓) the **ONE** box that best describes your health TODAY.

**1. Mobility**	
I have no problems in walking about	☐
I have slight problems in walking about	☐
I have moderate problems in walking about	☐
I have severe problems in walking about	☐
I am unable to walk about	☐

**2. Self-care**	
I have no problems washing or dressing myself	☐
I have slight problems washing or dressing myself	☐
I have moderate problems washing or dressing myself	☐
I have severe problems washing or dressing myself	☐
I am unable to wash or dress myself	☐

**3. Usual Activities** *(e.g. work, study, housework, family or leisure activities)*	
I have no problems doing my usual activities	☐
I have slight problems doing my usual activities	☐
I have moderate problems doing my usual activities	☐
I have severe problems doing my usual activities	☐
I am unable to do my usual activities	☐

**4. Pain/ Discomfort**	
I have no pain or discomfort	☐
I have slight pain or discomfort	☐
I have moderate pain or discomfort	☐
I have severe pain or discomfort	☐
I have extreme pain or discomfort	☐

**5. Anxiety/ Depression**	
I am not anxious or depressed	☐
I am slightly anxious or depressed	☐
I am moderately anxious or depressed	☐
I am severely anxious or depressed	☐
I am extremely anxious or depressed	☐

572

6. We would like to know how good or bad your health is TODAY.

This scale is numbered from 0 to 100.

100 means the <u>best</u> health you can imagine.

0 means the <u>worst</u> health you can imagine.

Please mark an X on the scale to indicate how your health is TODAY.

Now, write the number you marked on the scale in the box below.

Your health today = ☐

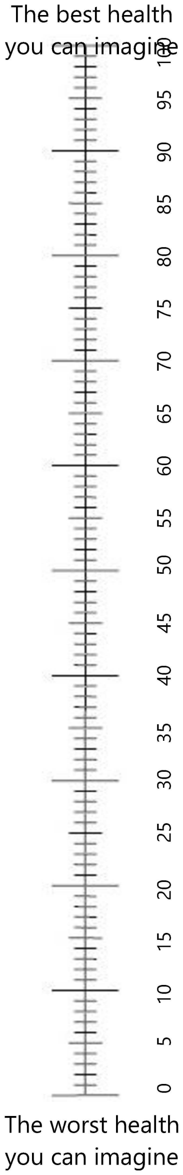

**7. Feeling settled and secure** *(Please tick (✓)* ***one*** *box that best describes your overall quality of life at the moment).*	
I am able to feel settled and secure in **all** areas of my life	☐
I am able to feel settled and secure in **many** areas of my life	☐
I am able to feel settled and secure in **a few** areas of my life	☐
I am unable to feel settled and secure in **any** areas of my life	☐

**8. Love, friendship and support** *Please tick (✓)* ***one*** *box that best describes your overall quality of life at the moment).*	
I can have **a lot** of love, friendship and support	☐
I can have **quite a lot** of love, friendship and support	☐
I can have **a little** love, friendship and support	☐
I **cannot** have **any** love, friendship and support	☐

**9. Being independent** *Please tick (✓)* ***one*** *box that best describes your overall quality of life at the moment).*	
I am able to be **completely** independent	☐
I am able to be independent in **many** things	☐
I am able to be independent in **a few** things	☐
I am **unable** to be at all independent	☐

**10. Achievement and progress** *Please tick (✓)* ***one*** *box that best describes your overall quality of life at the moment).*	
I can achieve and progress in **all** aspects of my life	☐
I can achieve and progress in **many** aspects of my life	☐
I can achieve and progress in **a few** aspects of my life	☐
I cannot achieve and progress in **any** aspects of my life	☐

**11. Enjoyment and pleasure** *Please tick (✓)* ***one*** *box that best describes your overall quality of life at the moment).*	
I can have **a lot** of enjoyment and pleasure	☐
I can have **quite a lot** of enjoyment and pleasure	☐
I can have **a little** enjoyment and pleasure	☐
I **cannot** have **any** enjoyment and pleasure	☐

577 ## Section 2: Health resources

578

1. In the last 3 months, have you seen any health professional at your GP surgery? (this includes phone/online or face-to-face appointments) (*Please tick (✓) **one** box).*

Yes	☐
No	☐

If YES, how many times were you seen by: *(Please enter a number)*

GP	
Practice nurse	
Other (please state)	
Other (please state)	

2. In the last 3 months, have you attended an Accident and Emergency (Casualty) department? *(Please tick (✓) **one** box).*

Yes	☐	If YES, how many times?	
No	☐		

579

3. In the last 3 months, have you attended hospital as an out-patient? *(Please tick (✓) **one** box).*

Yes	☐	If YES, how many times?	
No	☐		

580

4. In the last 3 months, have you attended hospital as an in-patient? *(Note: do not include stays for, or immediately after, giving birth)* *(Please tick (✓) **one** box).*

Yes	☐	If YES, how many times?	
		If YES, how many nights did you spend in hospital in the last 3 months in total?	
No	☐		

581

5. In the last 3 months, have you received any prescriptions for medicine? *(Please tick (✓) **one** box)***.**

Yes	☐	If YES, how many prescriptions have you received?	
No	☐		

6. In the last 3 months, have you purchased any over-the-counter medications or lifestyle products (e.g. slimming aids, vitamin supplements)? *(Please tick (✓) **one** box).*

Yes	☐	If YES, please state total amount spent in last 3 months	£
No	☐		

7. In the last 3 months have you paid for any services or clubs for the specific purpose of helping you with your lifestyle - for example slimming clubs, health clubs, gyms, swimming pools, exercise classes? *(Please tick (✓) **one** box).*

Yes	☐	If YES, approximately how much have you spent in <u>total</u> on all of these services/clubs in the <u>last 3 months?</u>	£
No	☐		

8. Please think about <u>last week's</u> food and drink purchases for your household (i.e. you and your family) and tell us the cost to your household of the following: *(Please write '**0**' if you don't purchase a specific type of food/product) .*

Type of food and drink purchased	Weekly Cost to you (your household)
Food and non-alcoholic drinks (e.g. supermarket shopping)	£
Alcoholic drinks e.g. wine & beer	£
Takeaway meals and snacks eaten AT HOME e.g. pizza delivery	£
Meals, snacks and drinks CONSUMED AWAY FROM HOME (e.g. restaurant)	£
Cigarettes or vaping products	£

9. Have you previously purchased any apps for improving your fitness/health or wellbeing? *(Please tick (✓) **one** box).*

Yes	☐	If YES, Please state the total amount you spent on fitness/health apps in the <u>last year?</u>	£
No	☐		

10. On average, <u>per day</u> how much time have you spent exercising/walking in the last 3 months in total?

*(Please answer in hours **AND** minutes. E.g. If you did 30 minutes of exercise per day, you can enter '0' under Hours and '30' under the Minutes column)*

On average per day: Hours ☐ Minutes ☐

11. Has your employment status changed in the last three months? *(Please tick (✓) **one** box).*

Yes ☐

No ☐

Section 3: Eating and activity approaches

This section asks you about your diet. *(Please circle **one** number in each row to indicate how much you agree or disagree with the following statements).*

**1. Eating healthier is something…**	**Strongly Disagree**						**Strongly Agree**
I do automatically	1	2	3	4	5	6	7
I do without having to consciously remember	1	2	3	4	5	6	7
I do without thinking	1	2	3	4	5	6	7
I start doing before I realise I'm doing it	1	2	3	4	5	6	7

**2. Over the next few months I intend to…**	**Strongly Disagree**						**Strongly Agree**
eat a healthy diet (e.g. reduce portion size, eat less sugary and high fact snacks and eat more fruit and vegetables)	1	2	3	4	5	6	7
be physically active (e.g. by walking more or taking exercise classes)	1	2	3	4	5	6	7

**3. I already have concrete plans…**	**Strongly Disagree**						**Strongly Agree**
on how to eat a healthy diet	1	2	3	4	5	6	7
on when to eat a healthy diet	1	2	3	4	5	6	7

**4. I already have concrete plans…**	**Strongly Disagree**						**Strongly Agree**
on when I need to be especially careful to maintain my healthy diet	1	2	3	4	5	6	7
about what to do in difficult situations to stick to my healthy diet	1	2	3	4	5	6	7
on how I will cope if I slip back into old eating habits	1	2	3	4	5	6	7

**5. I am confident that I can stick to a healthy diet even…**	**Strongly Disagree**						**Strongly Agree**
if I have to learn a lot about nutrition	1	2	3	4	5	6	7
if I initially have to watch out in many situations	1	2	3	4	5	6	7
if I have to start all over again several times until I succeed	1	2	3	4	5	6	7
if I initially have to make plans	1	2	3	4	5	6	7
if initially food doesn't taste as good	1	2	3	4	5	6	7
if I initially don't get much support	1	2	3	4	5	6	7
if it takes a long time to get used to it	1	2	3	4	5	6	7
if I have worries and troubles	1	2	3	4	5	6	7
if my partner/ my family don't change their nutrition habits	1	2	3	4	5	6	7
if I am tired	1	2	3	4	5	6	7
if I am stressed out	1	2	3	4	5	6	7
if I don't lose weight initially	1	2	3	4	5	6	7

6. In spite of good intentions when losing weight, slip-ups may occur. Imagine you relapse back into your old eating habits. How confident are you about restarting your new healthy eating habits?

**I am sure I can start my new healthy eating habits again regularly, even if I…**	**Strongly Disagree**						**Strongly Agree**
have several bad days in a row	1	2	3	4	5	6	7
lose my motivation to keep up my new healthy eating habits	1	2	3	4	5	6	7
have had a relapse and have fallen back into old eating habits for several weeks	1	2	3	4	5	6	7

7. For the next few questions:

'Tempting foods' are any food you want to eat more of than you think you should.
'Eating intentions' refer to the way you are aiming to eat, for example you may intend to avoid tempting foods or eat healthy foods.

*(Please tick (✓) **one** box for each of the following statements).*	**Never**	**Rarely**	**Sometimes**	**Often**	**Always**
I give up too easily on my eating intentions	☐	☐	☐	☐	☐
I'm good at resisting tempting food	☐	☐	☐	☐	☐
I easily get distracted from the way I intend to eat	☐	☐	☐	☐	☐
If I am not eating in the way I intend to I make changes	☐	☐	☐	☐	☐
I find it hard to remember what I have eaten throughout the day	☐	☐	☐	☐	☐

<u>This section asks about your physical activity</u>. *(Please circle **one** number in each row to indicate how much you agree or disagree with the following statements).*

**1. Being active every day is something…**	**Strongly Disagree**						**Strongly Agree**
I do automatically	1	2	3	4	5	6	7
I do without having to consciously remember	1	2	3	4	5	6	7
I do without thinking	1	2	3	4	5	6	7
I start doing before I realise I'm doing it	1	2	3	4	5	6	7

**2. I already have concrete plans…**	**Strongly Disagree**						**Strongly Agree**
on when to be physically active	1	2	3	4	5	6	7
on where to be physically active	1	2	3	4	5	6	7
on how to be physically active	1	2	3	4	5	6	7
on how often be physically active	1	2	3	4	5	6	7
on who I can be physically active with	1	2	3	4	5	6	7
**3. I already have concrete plans…**	**Strongly Disagree**						**Strongly Agree**

about what to do if something gets in the way	1	2	3	4	5	6	7
about what to do if I miss a physical activity session	1	2	3	4	5	6	7
about what to do in difficult situations to stick to my physical activity intentions	1	2	3	4	5	6	7
for times when I will need to be extra careful to stay committed	1	2	3	4	5	6	7

4a. Certain barriers make it hard to begin physical activity. How sure are you that you can begin exercising regularly?

**I am sure that…**	**Strongly Disagree**						**Strongly Agree**
I can change to a physically active lifestyle	1	2	3	4	5	6	7
I can be physically active once a week	1	2	3	4	5	6	7
I can be physically active at least 3 times a week for 30 minutes	1	2	3	4	5	6	7

**4b. I am sure I can start being physically active immediately, even if…**	**Strongly Disagree**						**Strongly Agree**
the planning for this takes a lot of time and effort	1	2	3	4	5	6	7
I have to force myself to start immediately	1	2	3	4	5	6	7
I have to push myself	1	2	3	4	5	6	7

5. Are you confident that you can manage staying physically active?

**I am sure I can keep being physically active regularly, even if...**	**Strongly Disagree**						**Strongly Agree**
it takes me a long time to make a habit	1	2	3	4	5	6	7
I am worried and troubled	1	2	3	4	5	6	7
I am tired	1	2	3	4	5	6	7
I am stressed out	1	2	3	4	5	6	7
I don't lose weight at once	1	2	3	4	5	6	7
I have to start all over again several times until I succeed	1	2	3	4	5	6	7
my partner/family isn't physically active	1	2	3	4	5	6	7

6. In spite of good intentions, slip ups may occur. How confident are you about restarting exercises?

**I am sure I can keep being physically active regularly, even if...**	**Strongly Disagree**						**Strongly Agree**
I postpone my plans several times	1	2	3	4	5	6	7
I lose my motivation	1	2	3	4	5	6	7
I have not been active for several weeks	1	2	3	4	5	6	7

Section 4: Social Support

1. Below is a list of statements about social support. *(Please tick (✓) **one** box in each row to indicate how much you agree or disagree with the following statements).*

Within the past 3 months, I have got support from my friends and/or family to help me:

	Strongly Agree	Agree	Neither agree or disagree	Disagree	Strongly disagree
eat well	☐	☐	☐	☐	☐
be physically active	☐	☐	☐	☐	☐
lose weight	☐	☐	☐	☐	☐

2. How often have you felt lonely during the past week?

*(Please tick (✓) **one** box).*

None, or almost none of the time	☐
Some of the time	☐
Most of the time	☐
All, or almost all of the time	☐
Don’t know	☐
Do not wish to answer	☐

3. How many people can you count on in times of need?

*(Please tick (✓) **one** box).*

0	☐	→ Please go to Section 5 on Page 16
1	☐	
2	☐	
3	☐	
4	☐	
5	☐	→ Please complete the next question in this section (i.e. question 4)
6	☐	
7	☐	
8	☐	
9	☐	
10 or more	☐	

**4. How many of these people live in your local area? That is, within about a mile or a 20 minute walk from your home.** *(Please tick (✓)* ***one*** *box).*	
0	☐
1	☐
2	☐
3	☐
4	☐
5	☐
6	☐
7	☐
8	☐
9	☐
10 or more	☐

Section 5: Lifestyle behaviours and attitudes

1. How often do you weigh yourself currently? *(Please tick (✓) **one** box).*

Never	☐
About once a year or less	☐
Every couple of months	☐
Every month	☐
Every week	☐
Every day	☐
More than once a day	☐

2. Within the past 3 months, have you taken part in any weight loss programmes (e.g. Slimming World). *(Please tick (✓) **one** box).*

Yes	☐
No	☐

If yes, please give details below;

Name of programme	*How long you attended for*

3. Do you want to lose weight? *(Please tick (✓) **one** box).*

Yes	☐	→ Please complete the rest of the questions in this section (i.e. questions 4, 5 & 6)
No	☐	→ Please go to Section 6 on Page 19

4. How confident are you in your ability to:

*(Please circle **one** number in each row to indicate how confident you feel about each of the following statements).*

	**Not confident**						**Very confident**
Lose weight	1	2	3	4	5	6	7
Keep lost weight off in the long term	1	2	3	4	5	6	7
Stay at current weight	1	2	3	4	5	6	7

5. How important is losing weight for you at the moment? *(Please circle **one** number).*

Not important 1	2	3	4	5	6	Very important 7
1	2	3	4	5	6	7

6. The questions below ask you about why you want to lose weight. *(Please tick (✓) **one** box for each statement).*

I want to lose weight…

	Absolutely not	Somewhat	Moderately	Strongly
Because it is commonly said that living with overweight is unhealthy	☐	☐	☐	☐
To be healthier	☐	☐	☐	☐
To be more agile	☐	☐	☐	☐
For health reasons	☐	☐	☐	☐
Because I read that it is healthier	☐	☐	☐	☐
To decrease my health risks	☐	☐	☐	☐
To live long	☐	☐	☐	☐
Because acquaintances have advised me to	☐	☐	☐	☐
To not attract attention	☐	☐	☐	☐
Because I'll be more successful in my job	☐	☐	☐	☐
So I will be accepted by society	☐	☐	☐	☐
To dare to socialise again	☐	☐	☐	☐
Because I would be luckier in love	☐	☐	☐	☐
To be more appreciated/liked	☐	☐	☐	☐
To have more friends	☐	☐	☐	☐
To have better success with others	☐	☐	☐	☐
So that other people will think better of me	☐	☐	☐	☐
To be more attractive	☐	☐	☐	☐
To like to look at myself in the mirror again	☐	☐	☐	☐
Because I want to like myself more	☐	☐	☐	☐

**I want to lose weight…**	**Absolutely not**	**Somewhat**	**Moderately**	**Strongly**
Because I want to be more attractive	☐	☐	☐	☐
To be able to dress more fashionably	☐	☐	☐	☐
To fit into my clothes again	☐	☐	☐	☐
To feel more self-confident	☐	☐	☐	☐

Section 6: Food patterns

*(Please tick (✓) **one** box for each of the following questions).*

**1. How many times a week do you eat two or more pieces of fruit?**	
6 or more days a week	☐
3-5 days a week	☐
1-2 days a week	☐
Less than one day a week	☐
Never	☐

**2. When eating cheese, how often do you choose reduced fat cheese in preference to regular cheese?**	
Always	☐
Usually	☐
Occasionally	☐
Rarely	☐
Never	☐
I don't eat cheese	☐

**3. How many days a week do you eat fried food with a batter or bread crumb coating?**	
4 or more days a week	☐
2 or 3 days a week	☐
Once a week	☐
Less than once a week	☐
Never	☐

**4. How often do you eat fried or roasted vegetables?**	
Always	☐
Usually	☐
Occasionally	☐
Rarely	☐
Never	☐

**5. When eating bread (as toast, sandwiches or a snack) how often do you spread butter or margarine on it?**	
Always	☐
Usually	☐
Occasionally	☐
Rarely	☐
Never	☐

Gallagher D, *et al. BMJ Open* 2024; 14:e084075. doi: 10.1136/bmjopen-2024-084075

**6. How many portions of vegetables do you eat in a typical day? (a portion is about three tablespoons of vegetables or a small bowl of salad).**	
5 or more portions	☐
3 or 4 portions	☐
1 or 2 portions	☐
Less than one portion per day	☐
None	☐

662

**7. How often do you trim all the visible fat off the meat you eat?**	
Always	☐
Usually	☐
Occasionally	☐
Rarely	☐
Never	☐
I don't eat meat	☐

663

**8. How many times a week do you eat meat pies, pasties or sausage rolls?**	
3 or more times a week	☐
Once or twice a week	☐
Once a fortnight	☐
Less than once a fortnight	☐
Never	☐

664

**9. How often do you (or the person who cooks for you) remove the skin from chicken before it is cooked?**	
Always	☐
Usually	☐
Occasionally	☐
Rarely	☐
Never	☐
I don’t eat chicken	☐

665

**10. How many days a week do you eat fried potato (e.g. hot chips or potato crisps)?**	
6 or more days a week	☐
3-5 days a week	☐
1-2 days a week	☐
Less than one day a week	☐
Never	☐

**11. How many days a week do you eat take-away foods such as: fried or BBQ chicken; fish and chips; Chinese; pizza; hamburger etc.?**	
6 or more days a week	☐
3-5 days a week	☐
1-2 days a week	☐
Less than one day a week	☐
Never	☐

**12. How often do you (or the person who cooks for you) use fat when cooking? (e.g. butter, margarine, oil, lard etc)**	
Always	☐
Usually	☐
Occasionally	☐
Rarely	☐
Never	☐

**13. How often do you choose wholemeal spaghetti or pasta in preference to regular spaghetti or pasta?**	
Always	☐
Usually	☐
Occasionally	☐
Rarely	☐
Never	☐
I don't eat spaghetti/pasta	☐

**14. How often do you choose wholemeal bread in preference to white bread?**	
Always	☐
Usually	☐
Occasionally	☐
Rarely	☐
Never	☐

**15. How many days a week do you eat legumes? (e.g. baked beans, three bean mix, lentils, split peas, dried beans etc)**	
4 or more days a week	☐
2 or 3 days a week	☐
Once a week	☐
Less than once a week	☐
Never	☐

**16. How many days a week do you eat a high fibre breakfast cereal? (e.g. Weetabix, All-Bran, untoasted muesli, porridge)**	
6 or more days a week	☐
3-5 days a week	☐
1-2 days a week	☐
Less than one day a week	☐
Never	☐

**17. How many different types of vegetables would you eat on a typical day?**	
5 or more types	☐
4 types	☐
3 types	☐
1 or 2 types	☐
None	☐

**18. How many days a week do you eat high fat cheeses? (e.g. cheddar or cream cheese)**	
6 or more days a week	☐
3-5 days a week	☐
1-2 days a week	☐
Less than one day a week	☐
Never	☐

**19. How often do you choose low-fat milk (semi-skimmed or skimmed) in preference to whole milk?**	
Always	☐
Usually	☐
Occasionally	☐
Rarely	☐
Never	☐
I don't drink milk	☐

**20. How many days a week do you eat processed meats? (e.g. bacon, salami, ham etc)**	
4 or more days a week	☐
2 or 3 days a week	☐
Once a week	☐
Less than one day a week	☐
Never	☐

**21. How often do you eat or drink any of the following?** *(Please tick (✓)* ***one*** *box for each row).*	**More than once a day**	**Once a day**	**3-6 days a week**	**1-2 days a week**	**less than weekly**	**Never**
Sweets, chocolate bars or biscuits (including wrapped chocolate biscuits like Twix or KitKat)	☐	☐	☐	☐	☐	☐
Buns, cakes or pastries or desserts	☐	☐	☐	☐	☐	☐
Fizzy drinks or squashes that contain sugar (e.g. coca cola, Ribena, Club Orange)	☐	☐	☐	☐	☐	☐
Diet drinks (e.g. Diet Coke, Sprite Zero, Diet Club)	☐	☐	☐	☐	☐	☐

**22. How often do you have a drink containing alcohol?**	
Never	☐
Monthly or less	☐
2 - 4 times a month	☐
2 – 3 times a week	☐
4 or more times a week	☐

**23. Do you keep a record of what you eat and drink? (e.g. writing it down, using an App etc).**	
Yes	☐
No	☐

**24. Do you set yourself food and drink related goals?** *(Please tick (✓)* ***one*** *box).*	
Yes	☐
No	☐

Section 7: Physical activity

We are interested in finding out about the kinds of physical activities that people do as part of their everyday lives. The questions will ask you about the time you spent being physically active **in the last 7 days**. Please answer each question even if you do not consider yourself to be an active person. Please think about the activities you do at work, as part of your house and yard work, to get from place to place, and in your spare time for recreation, exercise or sport.

VIGOROUS ACTIVITIES

Think about all the **vigorous** activities that you did **in the last 7 days**. **Vigorous** physical activities refer to activities that take hard physical effort and make you breathe much harder than normal. Think *only* about those physical activities that you did for at least 10 minutes at a time.

1. During the last 7 days, on how many days did you do vigorous physical activities like heavy lifting, digging, aerobics, or fast bicycling?

________	Days per week	
☐	No vigorous physical activities	⟶ ***Skip to question 3***

2. How much time did you usually spend doing vigorous physical activities on one of those days?

(Please answer in hours ***AND*** *minutes. E.g. If you did 30 minutes of vigorous exercise, you can enter '0' in the Hours box and '30' in the Minutes box)*

________	Hours per day
________	Minutes per day
☐	Don't know/not sure

<u>MODERATE ACTIVITIES</u>

Think about all the **moderate** activities that you did **in the last 7 days**. **Moderate** activities refer to activities that take moderate physical effort and make you breathe somewhat harder than normal. Think only about those physical activities that you did for at least 10 minutes at a time.

3. During the <u>last 7 days</u>, on how many days did you do <u>moderate</u> physical activities like carrying light loads, bicycling at a regular pace, or doubles tennis? Do not include walking.

______ Days per week

☐ No moderate physical activities ⟶ ***Skip to question 5***

4. How much time did you usually spend doing moderate physical activities on one of those days?

*(Please answer in hours **AND** minutes. E.g. If you did 30 minutes of moderate exercise, you can enter '0' in the Hours box and '30' in the Minutes box)*

______ Hours per day

______ Minutes per day

☐ Don't know/not sure

<u>WALKING</u>

Think about the time you spent **walking in the last 7 days**. This includes at work and at home, walking to travel from place to place, and any other walking that you have done solely for recreation, sport, exercise, or leisure.

5. During the <u>last 7 days</u>, on how many days did you <u>walk</u> for at least 10 minutes at a time?

______ Days per week

☐ No walking ⟶ ***Skip to question 7***

6. How much time did you usually spend walking on one of those days?

*(Please answer in hours **AND** minutes. E.g. If you walked for 2 hours, you can enter '2' in the Hours box and '0' in the Minutes box)*

______ Hours per day

______ Minutes per day

☐ Don't know/not sure

SITTING

The last question is about the time you spent **sitting** on weekdays during the **last 7 days**. Include time spent at work, at home, while doing course work and during leisure time. This may include time spent sitting at a desk, visiting friends, reading, or sitting or lying down to watch television.

**7. During the last 7 days, how much time did you spend sitting on a week day?** *(Please answer in hours **AND** minutes. E.g. If you spent 8 hours sitting, you can enter '8' in the Hours box and '0' in the Minutes box)*	
________	Hours per day
________	Minutes per day
☐	Don't know/not sure

STEPS

**8. Do you use a step counter? (e.g. pedometer, App, Fitbit etc)** *(Please tick (✓) **one** box).*	
Yes	☐
No	☐

**9. Do you set yourself physical activity related goals?** *(Please tick (✓) **one** box).*	
Yes	☐
No	☐

**10. Did you have any problem/s that limited your physical activity during the past 3 months?** *(Please tick (✓) **one** box).*		
Yes	☐	If yes, please give details:
No	☐	

Section 8: Infant Feeding

The questions in this section are designed to explore how you feed your youngest child. If your most recent pregnancy was a twin/multiple pregnancy, please complete these questions for the youngest twin/multiple.

1. What is your current method of feeding for your youngest child? *(Please tick (✓) **one** box for each row).*

Breast feeding	☐ yes	☐ no
Infant formula feeding	☐ yes	☐ no
Combined feeding (Breast and infant formula)	☐ yes	☐ no
Follow-on milk	☐ yes	☐ no
Baby is on solids	☐ yes	☐ no

NOTE: If you ticked 'Baby is on solids', **please go to question 2 below.**
If you did not tick 'Baby is on solids', **please go to Section 9 on Page 29.**

2. How often do you usually give your baby these particular TYPES of solid food? *(Please tick (✓) **one** box for each food).*

	**More than once a day**	**Once a day**	**3 or more times a week**	**Once or twice a week**	**Less than once a week**	**Never**
Breakfast Cereals	☐	☐	☐	☐	☐	☐
Rice or Pasta	☐	☐	☐	☐	☐	☐
Bread	☐	☐	☐	☐	☐	☐
Potatoes	☐	☐	☐	☐	☐	☐
Potato products (incl. chips, waffles, shapes)	☐	☐	☐	☐	☐	☐
Butter/Margarine and other spreads	☐	☐	☐	☐	☐	☐
Red meat	☐	☐	☐	☐	☐	☐
Processed meat (e.g. ham)	☐	☐	☐	☐	☐	☐

	**More than once a day**	**Once a day**	**3 or more times a week**	**Once or twice a week**	**Less than once a week**	**Never**
Chicken/other poultry	☐	☐	☐	☐	☐	☐
Fish (incl. tuna)	☐	☐	☐	☐	☐	☐
Eggs	☐	☐	☐	☐	☐	☐
Beans, lentils, chickpeas	☐	☐	☐	☐	☐	☐
Tofu, Quorn, textured vegetable protein	☐	☐	☐	☐	☐	☐
Nuts	☐	☐	☐	☐	☐	☐
Fruit	☐	☐	☐	☐	☐	☐
Vegetables	☐	☐	☐	☐	☐	☐
Cheese, yoghurt, fromage frais	☐	☐	☐	☐	☐	☐
Puddings or desserts	☐	☐	☐	☐	☐	☐
Biscuits, sweets, chocolate or cakes	☐	☐	☐	☐	☐	☐
Crisps and corn snacks	☐	☐	☐	☐	☐	☐
Follow on formula	☐	☐	☐	☐	☐	☐
Or something else (please tick and then write below)	☐	☐	☐	☐	☐	☐

**3. Do you ever add salt to your baby's solid food, including adding salt when the food is being cooked?** *(Please tick (✓)* ***one*** *box).*	
Yes, often	☐
Yes, sometimes	☐
Never	☐

Section 9: Mood

*(Please tick (✓) **one** box for each statement below).*

In the past 7 days…

**1. I have been able to laugh and see the funny side of things.**	
As much as I always could	☐
Not quite so much now	☐
Definitely not so much now	☐
Not at all	☐

**2. I have looked forward with enjoyment to things.**	
As much as I ever did	☐
Rather less than I used to	☐
Definitely less than I used to	☐
Hardly at all	☐

**3. I have blamed myself unnecessarily when things went wrong.**	
Yes, most of the time	☐
Yes, some of the time	☐
Not very often	☐
No, never	☐

**4. I have been anxious or worried for no good reason.**	
No, not at all	☐
Hardly ever	☐
Yes, sometimes	☐
Yes, very often	☐

**5. I have felt scared or panicky for no very good reason.**	
Yes, quite a lot	☐
Yes, sometimes	☐
No, not much	☐
No, not at all	☐

**6. Things have been getting on top of me.**	
Yes, most of the time I haven’t been able to cope at all	☐
Yes, sometimes I haven’t been coping as well as usual	☐
No, most of the time I have coped quite well	☐
No, I have been coping as well as ever	☐

**7. I have been so unhappy that I have had difficulty sleeping.**	
Yes, most of the time	☐
Yes, sometimes	☐
Not very often	☐
No, not at all	☐

**8. I have felt sad or miserable.**	
Yes, most of the time	☐
Yes, quite often	☐
Not very often	☐
No, not at all	☐

**9. I have been so unhappy that I have been crying.**	
Yes, most of the time	☐
Yes, quite often	☐
Only occasionally	☐
No, never	☐

**10. The thought of harming myself has occurred to me.**	
Yes, quite often	☐
Sometimes	☐
Hardly ever	☐
Never	☐

*(Please tick (✓) **one** box for each statement below).*

<u>Over the last two weeks</u>, how often have you been bothered by the following problems?

**11. Feeling nervous, anxious, or on edge?**	
Not at all	☐
Several days	☐
More than half of the days	☐
Nearly every day	☐

**12. Not being able to stop or control worrying?**	
Not at all	☐
Several days	☐
More than half of the days	☐
Nearly every day	☐

**13. Worrying too much about different things?**	
Not at all	☐
Several days	☐
More than half of the days	☐
Nearly every day	☐

**14. Trouble relaxing?**	
Not at all	☐
Several days	☐
More than half of the days	☐
Nearly every day	☐

**15. Being so restless that it is hard to sit still?**	
Not at all	☐
Several days	☐
More than half of the days	☐
Nearly every day	☐

**16. Becoming easily annoyed or irritable?**	
Not at all	☐
Several days	☐
More than half of the days	☐
Nearly every day	☐

**17. Feeling afraid as if something awful might happen?**	
Not at all	☐
Several days	☐
More than half of the days	☐
Nearly every day	☐

Having a new baby can bring up lots of different feelings and emotions. The next questions are designed to explore how you are feeling about being a parent to your baby. Answering these questions will help us to understand how things are going for you. If your most recent pregnancy was a twin/multiple pregnancy, please complete these questions for the youngest twin/multiple.

18. Thinking about your feelings about your baby, choose the response for each statement that feels right to you. *(Please tick (✓)* ***one*** *box for each of the following statements).*

	**Never**	**Sometimes**	**Always**
I enjoy looking after my baby	☐	☐	☐
I feel irritated with my baby when we are together	☐	☐	☐
I feel affectionate towards my baby	☐	☐	☐
I feel that my baby is being difficult or trying to upset me on purpose	☐	☐	☐
I can work out what my baby needs from me	☐	☐	☐
I feel like I can't do things I enjoy because of my baby.	☐	☐	☐
I feel the changes in my life are worth it to look after my baby.	☐	☐	☐
I miss my baby when we are not together	☐	☐	☐
I feel like I'm looking after my baby for someone else.	☐	☐	☐

	Never	Sometimes	Always
When we've been apart I look forward to seeing my baby again.	☐	☐	☐
I enjoy playing with my baby.	☐	☐	☐

19. Thinking about your baby, please tick one of the choices for each of the questions below. There are no 'right' or 'wrong' answers; many of these are true of all babies at times. *(Please tick (✓)* ***one*** *box for each of the following statements).*

	Always	Very often	Quite often	Sometimes	Rarely	Never
My baby smiles at me.	☐	☐	☐	☐	☐	☐
My baby annoys me.	☐	☐	☐	☐	☐	☐
My baby likes doing things with me.	☐	☐	☐	☐	☐	☐
My baby 'talks' to me.	☐	☐	☐	☐	☐	☐
My baby irritates me.	☐	☐	☐	☐	☐	☐
My baby likes me.	☐	☐	☐	☐	☐	☐
My baby wants too much attention.	☐	☐	☐	☐	☐	☐
My baby laughs.	☐	☐	☐	☐	☐	☐
My baby gets moody.	☐	☐	☐	☐	☐	☐
My baby dominates me.	☐	☐	☐	☐	☐	☐
My baby likes to please me.	☐	☐	☐	☐	☐	☐
My baby cries for no obvious reason.	☐	☐	☐	☐	☐	☐
My baby is affectionate towards me.	☐	☐	☐	☐	☐	☐
My baby winds me up.	☐	☐	☐	☐	☐	☐

Section 10: Self-esteem

Below is a list of statements dealing with your general feelings about yourself.

*(Please tick (✓) **one** box to indicate how much you agree or disagree with each of the following statements).*

	Strongly Agree	Agree	Disagree	Strongly disagree
I feel that I'm a person of worth, at least on an equal plane with others.	☐	☐	☐	☐
I feel that I have a number of good qualities.	☐	☐	☐	☐
All in all, I am inclined to feel that I am a failure.	☐	☐	☐	☐
I am able to do things as well as most other people.	☐	☐	☐	☐
I feel I do not have much to be proud of.	☐	☐	☐	☐
I take a positive attitude toward myself.	☐	☐	☐	☐
On the whole I am satisfied with myself.	☐	☐	☐	☐
I wish I could have more respect for myself.	☐	☐	☐	☐
I certainly feel useless at times.	☐	☐	☐	☐
At times I think I am no good at all.	☐	☐	☐	☐

Section 11: Sleep

Gallagher D, *et al. BMJ Open* 2024; 14:e084075. doi: 10.1136/bmjopen-2024-084075

1. At the moment, how often does your baby sleep all night? *(Please tick (✓) **one** box).*

Always	☐
Mostly	☐
Sometimes	☐
Rarely	☐
Never	☐

The following questions relate to **your** usual sleep habits during the **<u>past month only</u>**. Your answers should indicate the most accurate reply for the <u>majority</u> of days and nights in the past month. Please answer all questions.

2. During the past month, what time have you usually gone to bed at night?

Usual bed time ☐

3. During the past month, how long (in minutes) has it usually taken you to fall asleep each night?

Number of minutes ☐

4. During the past month, what has been your usual getting up time in the morning?

Usual getting up time ☐

5. During the past month, on average how many hours of <u>actual sleep</u> did you get at night? (This may be different than the number of hours you spent in bed.)

Hours of sleep per night ☐

6. During the past month, how often have you had trouble sleeping because you…

(Please tick (✓) ***one*** *box for each statement).*	**Not during the past month**	**Less than once a week**	**Once or twice a week**	**Three or more times a week**
Cannot get to sleep within 30 minutes	☐	☐	☐	☐
Wake up in the middle of the night or early morning	☐	☐	☐	☐
Have to get up to use the bathroom	☐	☐	☐	☐
Cannot breathe comfortably	☐	☐	☐	☐
Cough or snore loudly	☐	☐	☐	☐
Feel too cold	☐	☐	☐	☐
Feel too hot	☐	☐	☐	☐
Had bad dreams	☐	☐	☐	☐
Have pain	☐	☐	☐	☐
Have to take care of your baby	☐	☐	☐	☐
Other reason(s), please describe and indicate how often during the past month you had trouble sleeping because of this:				
☐	☐	☐	☐	☐
☐	☐	☐	☐	☐
☐	☐	☐	☐	☐

7. During the past month, how would you rate your sleep quality overall?
(Please tick (✓) ***one*** *box).*

Very Good	Fairly Good	Fairly Bad	Very Bad
☐	☐	☐	☐

**8**. (Please tick *(✓)* ***one*** *box for each question below.)*	Not during the past month	Less than once a week	Once or twice a week	Three or more times a week
**During the past month, how often have you taken medicine (prescribed or "over the counter") to help you sleep?**	☐	☐	☐	☐
**During the past month, how often have you had trouble staying awake while driving, eating meals, or engaging in social activity?**	☐	☐	☐	☐

9. During the past month, how much of a problem has it been for you to keep up enough enthusiasm to get things done? (Please tick *(✓)* ***one*** *box).*

No problem at all	Only a very slight problem	Somewhat of a problem	A very big problem
☐	☐	☐	☐

10. Do you usually have a bed partner or room-mate? *(Please tick (✓)* ***one*** *box).*

No bed partner or room-mate	Partner/room-mate in other room	Partner in same room but not in same bed	Partner in same bed
☐	☐	☐	☐

11. If you usually have a room-mate or bed partner, ask him/her how often in the past month you have had:

Please tick *(✓)* ***one*** *box for each statement).*	Not during the	Less than	Once or	Three or more

57

	past month	once a week	twice a week	times a week
Loud snoring	☐	☐	☐	☐
Long pauses between breaths while asleep	☐	☐	☐	☐
Legs twitching or jerking while you sleep	☐	☐	☐	☐
Episodes of confusion during sleep	☐	☐	☐	☐
Other restlessness while you sleep, please describe and indicate how often in the past month you have had this:				
☐	☐	☐	☐	☐
☐	☐	☐	☐	☐
☐	☐	☐	☐	☐

You have now completed the questionnaire. Thank you very much for your time and effort! We really appreciate your help with our research.

Supporting MumS (SMS) study Questionnaire Booklet

Month 12 Intervention

**Participant ID:**	_ _ _ _ _
**Researcher name:**	
**Today's date:**	_ _ / _ _ / _ _ _ _<br>**e.g. 05 / 01 / 2017**

60

About this questionnaire booklet

Thank you for taking the time to fill out this questionnaire booklet.

The questionnaire booklet is made up of questions that will help us find out more about your experience of the postpartum period. Please complete the questions as accurately as you can. **There are no right or wrong answers**. If there are any questions you do not wish to answer, you can leave them blank. Note that your responses will be **anonymous,** this means your name will never be given with your responses.

The questionnaire booklet is split up into the short sections shown below. You can fill it all out at once, or do it in chunks, as your time allows.

**Section 1**	**SMS evaluation**	**Pages 3-8**
**Section 2**	**Health and well-being**	**Pages 9-11**
**Section 3**	**Health resources**	**Pages 12-14**
**Section 4**	**Eating and activity approaches**	**Pages 15-19**
**Section 5**	**Social support**	**Page 20-21**
**Section 6**	**Lifestyle behaviours and attitudes**	**Page 22-24**
**Section 7**	**Food patterns**	**Page 25-29**
**Section 8**	**Physical activity**	**Page 30-32**
**Section 9**	**Infant feeding**	**Page 33-34**
**Section 10**	**Mood**	**Page 35-42**
**Section 11**	**Self-esteem**	**Page 43**
**Section 12**	**Sleep**	**Page 44-47**

Section 1: SMS Evaluation

Thank you for participating in our study. We would like to ask you some questions about how you have found participating in this research. Your responses to this survey will help us to improve the studies we carry out in the future.

1. How satisfied were you with your overall experience of the SMS study? *(Please tick (✓) **one** box).*

Very Dissatisfied	Mostly Dissatisfied	Neither Satisfied Nor Dissatisfied	Mostly Satisfied	Very Satisfied
☐	☐	☐	☐	☐

If you were very or mostly dissatisfied, please explain what could have been improved:

____

____

____

2. Please answer the questions below about your experience of taking part in the SMS study. *(Please tick (✓) **one** box to indicate how much you agree or disagree with each of the following statements).*

	Strongly disagree	Disagree	Neither agree or disagree	Agree	Strongly Agree
At the start of the study, the information I was given about the study was clear and informative	☐	☐	☐	☐	☐
The amount of information and level of support provided by the researchers during the study was suited to my needs	☐	☐	☐	☐	☐

If you have disagreed or strongly disagreed with any of the above statements, please explain what could have been improved:

____

____

____

909

3. How satisfied were you with the text messages? *(Please tick (✓)* ***one*** *box).*

Very Dissatisfied	Mostly Dissatisfied	Neither Satisfied Nor Dissatisfied	Mostly Satisfied	Very Satisfied
☐	☐	☐	☐	☐

910

If you were very or mostly dissatisfied, please explain what could have been improved:

____

____

____

911

4. **Please answer the questions below about your experience of the text messages you received during the SMS study.** *(Please tick (✓)* ***one*** *box to indicate how much you agree or disagree with each of the following statements).*

	Strongly Disagree	Disagree	Neither agree or disagree	Agree	Strongly agree
SMS texts were easy to understand	☐	☐	☐	☐	☐
SMS texts were helpful	☐	☐	☐	☐	☐
SMS texts were interesting	☐	☐	☐	☐	☐
An appropriate amount of SMS texts were sent during the study	☐	☐	☐	☐	☐
SMS texts were delivered at appropriate times of the day	☐	☐	☐	☐	☐

912

If you have disagreed or strongly disagreed with any of the above statements, please explain what could have been improved:

____

____

____

913

Gallagher D, *et al. BMJ Open* 2024; 14:e084075. doi: 10.1136/bmjopen-2024-084075

5. **How helpful did you find the following parts of SMS in helping you to lose weight and maintain weight loss?** *(Please tick (✓) **one** response for each statement below)*

	Very unhelpful	Somewhat unhelpful	Neither helpful or unhelpful	Somewhat helpful	Very helpful	Didn't use it
Reminders to set goals	☐	☐	☐	☐	☐	☐
Reminders to weigh yourself weekly	☐	☐	☐	☐	☐	☐
Trigger words (e.g. 'crave', 'tired' etc.)	☐	☐	☐	☐	☐	☐
Support system (i.e. having a friend receive SMS messages as well)	☐	☐	☐	☐	☐	☐
The Yes/No replies	☐	☐	☐	☐	☐	☐
Web links provided within the text messages	☐	☐	☐	☐	☐	☐
The feedback messages about your weight (i.e. replies to UP, DOWN, SAME texts)	☐	☐	☐	☐	☐	☐

If you have felt any aspect was not helpful, please explain what could have been improved:

____

____

____

6. **During the study you were asked to attend 3 study visits. Please rate how easy or difficult you found each aspect of these visits.** *(Please tick (✓) **one** response that most applies to you in relation to your experience).*

	Very difficult	Difficult	No strong opinion	Easy	Very easy
Location of visits	☐	☐	☐	☐	☐
Length of visits	☐	☐	☐	☐	☐
Having your height measurement taken	☐	☐	☐	☐	☐
Having your waist measurements taken	☐	☐	☐	☐	☐
Having your weight taken	☐	☐	☐	☐	☐
Completing the questionnaires	☐	☐	☐	☐	☐

If you have felt that any aspects of the visits were difficult, please explain how they could have been improved:

____

____

____

7. Did you sign-up to receive additional text messages on giving up smoking during SMS? *(Please tick (✓)* ***one*** *box).*

Yes	☐	→ Please complete Question 8.
No	☐	→ Please go to Question 9.

8. Did you give up smoking during SMS? *(Please tick (✓)* ***one*** *box).*

Yes	☐
No	☐

9. Did you sign-up to receive additional text messages on breastfeeding during SMS? *(Please tick (✓)* ***one*** *box).*

Yes	☐	→ Please complete Question 10.
No	☐	→ Please go to Question 11.

10. How helpful did you find the breastfeeding messages? *(Please tick (✓)* ***one*** *box).*

Very Unhelpful	Somewhat Unhelpful	Neither helpful or unhelpful	Somewhat helpful	Very helpful
☐	☐	☐	☐	☐

If you felt these messages were not helpful, please explain what could have been improved:

____

____

____

11. Based on your experience in taking part in SMS, do you think the programme should be offered to other mums? (*Please tick (✓)* ***one*** *response).*

Yes	☐
No	☐

If you answered no, please explain why:

____
____
____

12. Do you have any other comments? We would welcome any suggestions you have regarding how we could improve future studies for those taking part:

____
____
____

Gallagher D, *et al. BMJ Open* 2024; 14:e084075. doi: 10.1136/bmjopen-2024-084075

Section 2: Health and well-being

For questions 1-5, under each heading, please tick (✓) the **ONE** box that best describes your health TODAY.

**1. Mobility**	
I have no problems in walking about	☐
I have slight problems in walking about	☐
I have moderate problems in walking about	☐
I have severe problems in walking about	☐
I am unable to walk about	☐

**2. Self-care**	
I have no problems washing or dressing myself	☐
I have slight problems washing or dressing myself	☐
I have moderate problems washing or dressing myself	☐
I have severe problems washing or dressing myself	☐
I am unable to wash or dress myself	☐

**3. Usual Activities** *(e.g. work, study, housework, family or leisure activities)*	
I have no problems doing my usual activities	☐
I have slight problems doing my usual activities	☐
I have moderate problems doing my usual activities	☐
I have severe problems doing my usual activities	☐
I am unable to do my usual activities	☐

**4. Pain/ Discomfort**	
I have no pain or discomfort	☐
I have slight pain or discomfort	☐
I have moderate pain or discomfort	☐
I have severe pain or discomfort	☐
I have extreme pain or discomfort	☐

**5. Anxiety/ Depression**	
I am not anxious or depressed	☐
I am slightly anxious or depressed	☐
I am moderately anxious or depressed	☐
I am severely anxious or depressed	☐
I am extremely anxious or depressed	☐

6. We would like to know how good or bad your health is TODAY.

This scale is numbered from 0 to 100.

100 means the <u>best</u> health you can imagine.

0 means the <u>worst</u> health you can imagine.

Please mark an X on the scale to indicate how your health is TODAY.

Now, write the number you marked on the scale in the box below.

Your health today = ☐

The best health you can imagine

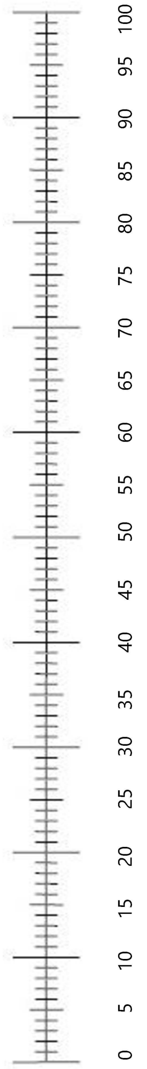

The worst health you can imagine

**7. Feeling settled and secure** *(Please tick (✓) **one** box that best describes your overall quality of life at the moment).*	
I am able to feel settled and secure in **all** areas of my life	☐
I am able to feel settled and secure in **many** areas of my life	☐
I am able to feel settled and secure in **a few** areas of my life	☐
I am unable to feel settled and secure in **any** areas of my life	☐

**8. Love, friendship and support** *Please tick (✓) **one** box that best describes your overall quality of life at the moment).*	
I can have **a lot** of love, friendship and support	☐
I can have **quite a lot** of love, friendship and support	☐
I can have **a little** love, friendship and support	☐
I **cannot** have **any** love, friendship and support	☐

**9. Being independent** *Please tick (✓) **one** box that best describes your overall quality of life at the moment).*	
I am able to be **completely** independent	☐
I am able to be independent in **many** things	☐
I am able to be independent in **a few** things	☐
I am **unable** to be at all independent	☐

**10. Achievement and progress** *Please tick (✓) **one** box that best describes your overall quality of life at the moment).*	
I can achieve and progress in **all** aspects of my life	☐
I can achieve and progress in **many** aspects of my life	☐
I can achieve and progress in **a few** aspects of my life	☐
I cannot achieve and progress in **any** aspects of my life	☐

**11. Enjoyment and pleasure** *Please tick (✓) **one** box that best describes your overall quality of life at the moment).*	
I can have **a lot** of enjoyment and pleasure	☐
I can have **quite a lot** of enjoyment and pleasure	☐
I can have **a little** enjoyment and pleasure	☐
I **cannot** have **any** enjoyment and pleasure	☐

Section 3: Health resources

1. 1. In the last 3 months, have you seen any health professional at your GP surgery? (this includes phone/online or face-to-face appointments) (*Please tick (✓) **one** box).*

Yes	☐
No	☐

If YES, how many times were you seen by: *(Please enter a number)*

GP	
Practice nurse	
Other (please state)	
Other (please state)	

2. In the last 3 months, have you attended an Accident and Emergency (Casualty) department? *(Please tick (✓) **one** box).*

Yes	☐	If YES, how many times?	
No	☐		

3. In the last 3 months, have you attended hospital as an out-patient? *(Please tick (✓) **one** box).*

Yes	☐	If YES, how many times?	
No	☐		

4. In the last 3 months, have you attended hospital as an in-patient? *(Please tick (✓) **one** box).*

Yes	☐	If YES, how many times?	
		If YES, how many nights did you spend in hospital in the last 3 months in total?	
No	☐		

5. In the last 3 months, have you received any prescriptions for medicine? *(Please tick (✓) **one** box)***.**

Yes	☐	If YES, how many prescriptions have you received?	
No	☐		

Gallagher D, *et al. BMJ Open* 2024; 14:e084075. doi: 10.1136/bmjopen-2024-084075

6. In the last 3 months, have you purchased any over-the-counter medications or lifestyle products (e.g. slimming aids, vitamin supplements)? *(Please tick (✓) **one** box).*

Yes	☐	If YES, please state total amount spent in last 3 months	£
No	☐		

7. In the last 3 months have you paid for any services or clubs for the specific purpose of helping you with your lifestyle - for example slimming clubs, health clubs, gyms, swimming pools, exercise classes? *(Please tick (✓) **one** box).*

Yes	☐	If YES, approximately how much have you spent in total on all of these services/clubs in the last 3 months?	£
No	☐		

8. Please think about last week's food and drink purchases for your household (i.e. you and your family) and tell us the cost to your household of the following:
*(Please write '**0**' if you don't purchase a specific type of food/product) .*

Type of food and drink purchased	Weekly Cost to you (your household)
Food and non-alcoholic drinks (e.g. supermarket shopping)	£
Alcoholic drinks e.g. wine & beer	£
Takeaway meals and snacks eaten AT HOME e.g. pizza delivery	£
Meals, snacks and drinks CONSUMED AWAY FROM HOME (e.g. restaurant)	£
Cigarettes or vaping products	£

9. Have you previously purchased any apps for improving your fitness/health or wellbeing? *(Please tick (✓) **one** box).*

Yes	☐	If YES, Please state the total amount you spent on fitness/health apps in the last year?	£
No	☐		

Gallagher D, *et al. BMJ Open* 2024; 14:e084075. doi: 10.1136/bmjopen-2024-084075

10. On average, <u>per day</u> how much time have you spent exercising/walking in the last 3 months in total?

*(Please answer in hours **AND** minutes. E.g. If you did 30 minutes of exercise per day, you can enter '0' under Hours and '30' under the Minutes column)*

On average per day: Hours ☐ Minutes ☐

11. Has your employment status changed in the last three months?

(Note: returning to work after maternity leave should **not** be included as a change) *(Please tick (✓) **one** box).*

- Yes ☐
- No ☐

If YES, what is your **current** employment status? *(Please tick (✓) **one** box)*

- ☐ Full-time employment
- ☐ Part-time employment
- ☐ Unemployed
- ☐ Student or training
- ☐ Retired
- ☐ Not working due to illness
- ☐ Prefer not to answer
- ☐ Other (please specify) ______

Section 4: Eating and activity approaches

This section asks you about your diet. *(Please circle **one** number in each row to indicate how much you agree or disagree with the following statements).*

**1. Eating healthier is something...**	**Strongly Disagree**						**Strongly Agree**
I do automatically	1	2	3	4	5	6	7
I do without having to consciously remember	1	2	3	4	5	6	7
I do without thinking	1	2	3	4	5	6	7
I start doing before I realise I'm doing it	1	2	3	4	5	6	7

**2. Over the next few months I intend to...**	**Strongly Disagree**						**Strongly Agree**
eat a healthy diet (e.g. reduce portion size, eat less sugary and high fact snacks and eat more fruit and vegetables)	1	2	3	4	5	6	7
be physically active (e.g. by walking more or taking exercise classes)	1	2	3	4	5	6	7

**3. I already have concrete plans...**	**Strongly Disagree**						**Strongly Agree**
on how to eat a healthy diet	1	2	3	4	5	6	7
on when to eat a healthy diet	1	2	3	4	5	6	7

**4. I already have concrete plans...**	**Strongly Disagree**						**Strongly Agree**
on when I need to be especially careful to maintain my healthy diet	1	2	3	4	5	6	7
about what to do in difficult situations to stick to my healthy diet	1	2	3	4	5	6	7
on how I will cope if I slip back into old eating habits	1	2	3	4	5	6	7

Gallagher D, *et al. BMJ Open* 2024; 14:e084075. doi: 10.1136/bmjopen-2024-084075

**5. I am confident that I can stick to a healthy diet even…**	**Strongly Disagree**						**Strongly Agree**
if I have to learn a lot about nutrition	1	2	3	4	5	6	7
if I initially have to watch out in many situations	1	2	3	4	5	6	7
if I have to start all over again several times until I succeed	1	2	3	4	5	6	7
if I initially have to make plans	1	2	3	4	5	6	7
if initially food doesn't taste as good	1	2	3	4	5	6	7
if I initially don't get much support	1	2	3	4	5	6	7
if it takes a long time to get used to it	1	2	3	4	5	6	7
if I have worries and troubles	1	2	3	4	5	6	7
if my partner/ my family don't change their nutrition habits	1	2	3	4	5	6	7
if I am tired	1	2	3	4	5	6	7
if I am stressed out	1	2	3	4	5	6	7
if I don't lose weight initially	1	2	3	4	5	6	7

6. In spite of good intentions when losing weight, slip-ups may occur. Imagine you relapse back into your old eating habits. How confident are you about restarting your new healthy eating habits?

**I am sure I can start my new healthy eating habits again regularly, even if I…**	**Strongly Disagree**						**Strongly Agree**
have several bad days in a row	1	2	3	4	5	6	7
lose my motivation to keep up my new healthy eating habits	1	2	3	4	5	6	7
have had a relapse and have fallen back into old eating habits for several weeks	1	2	3	4	5	6	7

7. For the next few questions:

'Tempting foods' are any food you want to eat more of than you think you should. **'Eating intentions'** refer to the way you are aiming to eat, for example you may intend to avoid tempting foods or eat healthy foods.

*(Please tick (✓) **one** box for each of the following statements).*	**Never**	**Rarely**	**Sometimes**	**Often**	**Always**
I give up too easily on my eating intentions	☐	☐	☐	☐	☐
I'm good at resisting tempting food	☐	☐	☐	☐	☐
I easily get distracted from the way I intend to eat	☐	☐	☐	☐	☐
If I am not eating in the way I intend to I make changes	☐	☐	☐	☐	☐
I find it hard to remember what I have eaten throughout the day	☐	☐	☐	☐	☐

<u>This section asks about your physical activity</u>. *(Please circle **one** number in each row to indicate how much you agree or disagree with the following statements).*

**1. Being active every day is something…**	**Strongly Disagree**						**Strongly Agree**
I do automatically	1	2	3	4	5	6	7
I do without having to consciously remember	1	2	3	4	5	6	7
I do without thinking	1	2	3	4	5	6	7
I start doing before I realise I'm doing it	1	2	3	4	5	6	7

**2. I already have concrete plans…**	**Strongly Disagree**						**Strongly Agree**
on when to be physically active	1	2	3	4	5	6	7
on where to be physically active	1	2	3	4	5	6	7
on how to be physically active	1	2	3	4	5	6	7
on how often be physically active	1	2	3	4	5	6	7
on who I can be physically active with	1	2	3	4	5	6	7

**3. I already have concrete plans…**	**Strongly Disagree**						**Strongly Agree**
about what to do if something gets in the way	1	2	3	4	5	6	7
about what to do if I miss a physical activity session	1	2	3	4	5	6	7
about what to do in difficult situations to stick to my physical activity intentions	1	2	3	4	5	6	7
for times when I will need to be extra careful to stay committed	1	2	3	4	5	6	7

4a. Certain barriers make it hard to begin physical activity. How sure are you that you can begin exercising regularly?

**I am sure that…**	**Strongly Disagree**						**Strongly Agree**
I can change to a physically active lifestyle	1	2	3	4	5	6	7
I can be physically active once a week	1	2	3	4	5	6	7
I can be physically active at least 3 times a week for 30 minutes	1	2	3	4	5	6	7

**4b. I am sure I can start being physically active immediately, even if…**	**Strongly Disagree**						**Strongly Agree**
the planning for this takes a lot of time and effort	1	2	3	4	5	6	7
I have to force myself to start immediately	1	2	3	4	5	6	7
I have to push myself	1	2	3	4	5	6	7

5. Are you confident that you can manage staying physically active?

**I am sure I can keep being physically active regularly, even if…**	**Strongly Disagree**						**Strongly Agree**
it takes me a long time to make a habit	1	2	3	4	5	6	7
I am worried and troubled	1	2	3	4	5	6	7
I am tired	1	2	3	4	5	6	7
I am stressed out	1	2	3	4	5	6	7
I don't lose weight at once	1	2	3	4	5	6	7
I have to start all over again several times until I succeed	1	2	3	4	5	6	7
my partner/family isn't physically active	1	2	3	4	5	6	7

6. In spite of good intentions, slip ups may occur. How confident are you about restarting exercises?

**I am sure I can keep being physically active regularly, even if…**	**Strongly Disagree**						**Strongly Agree**
I postpone my plans several times	1	2	3	4	5	6	7
I lose my motivation	1	2	3	4	5	6	7
I have not been active for several weeks	1	2	3	4	5	6	7

Section 5: Social Support

1. Below is a list of statements about social support. *(Please tick (✓) **one** box in each row to indicate how much you agree or disagree with the following statements).*
Within the past 3 months, I have got support from my friends and/or family to help me:

	Strongly Agree	Agree	Neither agree or disagree	Disagree	Strongly disagree
...eat well	☐	☐	☐	☐	☐
...be physically active	☐	☐	☐	☐	☐
...lose weight	☐	☐	☐	☐	☐

2. How often have you felt lonely during the past week?
*(Please tick (✓) **one** box).*

None, or almost none of the time	☐
Some of the time	☐
Most of the time	☐
All, or almost all of the time	☐
Don't know	☐
suDo not wish to answer	☐

3. How many people can you count on in times of need?
*(Please tick (✓) **one** box).*

0	☐	→ Please go to Section 6 on Page 22
1	☐	→ Please complete the next question in this section (i.e. question 4)
2	☐	
3	☐	
4	☐	
5	☐	
6	☐	
7	☐	
8	☐	
9	☐	
10 or more	☐	

4. How many of these people live in your local area? That is, within about a mile or a 20 minute walk from your home.
*(Please tick (✓) **one** box).*

0	☐
1	☐
2	☐
3	☐
4	☐
5	☐
6	☐
7	☐
8	☐
9	☐
10 or more	☐

Section 6: Lifestyle behaviours and attitudes

4. How often do you weigh yourself currently? *(Please tick (✓) **one** box).*

Never	☐
About once a year or less	☐
Every couple of months	☐
Every month	☐
Every week	☐
Every day	☐
More than once a day	☐

5. Within the past 3 months, have you taken part in any weight loss programmes (e.g. Slimming World). *(Please tick (✓) **one** box).*

Yes	☐
No	☐

If yes, please give details below;

Name of programme	*How long you attended for*

6. Do you want to lose weight? *(Please tick (✓) **one** box).*

Yes	☐	→ Please complete the rest of the questions in this section (i.e. questions 4, 5 & 6)
No	☐	→ Please go to Section 7 on Page 25

4. How confident are you in your ability to:

*(Please circle **one** number in each row to indicate how confident you feel about each of the following statements).*	**Not confident**						**Very confident**
Lose weight	1	2	3	4	5	6	7
Keep lost weight off in the long term	1	2	3	4	5	6	7
Stay at current weight	1	2	3	4	5	6	7

5. How important is losing weight for you at the moment? *(Please circle **one** number).*

	Not important						Very important
	1	2	3	4	5	6	7

6. The questions below ask you about why you want to lose weight. *(Please tick (✓) **one** box for each statement).*

I want to lose weight…

	Absolutely not	Somewhat	Moderately	Strongly
Because it is commonly said that living with overweight is unhealthy	☐	☐	☐	☐
To be healthier	☐	☐	☐	☐
To be more agile	☐	☐	☐	☐
For health reasons	☐	☐	☐	☐
Because I read that it is healthier	☐	☐	☐	☐
To decrease my health risks	☐	☐	☐	☐
To live long	☐	☐	☐	☐
Because acquaintances have advised me to	☐	☐	☐	☐
To not attract attention	☐	☐	☐	☐
Because I'll be more successful in my job	☐	☐	☐	☐
So I will be accepted by society	☐	☐	☐	☐
To dare to socialise again	☐	☐	☐	☐
Because I would be luckier in love	☐	☐	☐	☐
To be more appreciated/liked	☐	☐	☐	☐
To have more friends	☐	☐	☐	☐
To have better success with others	☐	☐	☐	☐
So that other people will think better of me	☐	☐	☐	☐
To be more attractive	☐	☐	☐	☐
To like to look at myself in the mirror again	☐	☐	☐	☐
Because I want to like myself more	☐	☐	☐	☐

Gallagher D, *et al. BMJ Open* 2024; 14:e084075. doi: 10.1136/bmjopen-2024-084075

**I want to lose weight…**	**Absolutely not**	**Somewhat**	**Moderately**	**Strongly**
Because I want to be more attractive	☐	☐	☐	☐
To be able to dress more fashionably	☐	☐	☐	☐
To fit into my clothes again	☐	☐	☐	☐
To feel more self-confident	☐	☐	☐	☐

Gallagher D, *et al. BMJ Open* 2024; 14:e084075. doi: 10.1136/bmjopen-2024-084075

Section 7: Food patterns

*(Please tick (✓) **one** box for each of the following questions).*

**25. How many times a week do you eat two or more pieces of fruit?**	
6 or more days a week	☐
3-5 days a week	☐
1-2 days a week	☐
Less than one day a week	☐
Never	☐

**26. When eating cheese, how often do you choose reduced fat cheese in preference to regular cheese?**	
Always	☐
Usually	☐
Occasionally	☐
Rarely	☐
Never	☐
I don't eat cheese	☐

**27. How many days a week do you eat fried food with a batter or bread crumb coating?**	
4 or more days a week	☐
2 or 3 days a week	☐
Once a week	☐
Less than once a week	☐
Never	☐

**28. How often do you eat fried or roasted vegetables?**	
Always	☐
Usually	☐
Occasionally	☐
Rarely	☐
Never	☐

**29. When eating bread (as toast, sandwiches or a snack) how often do you spread butter or margarine on it?**	
Always	☐
Usually	☐
Occasionally	☐
Rarely	☐
Never	☐

**30. How many portions of vegetables do you eat in a typical day? (a portion is about three tablespoons of vegetables or a small bowl of salad).**	
5 or more portions	☐
3 or 4 portions	☐
1 or 2 portions	☐
Less than one portion per day	☐
None	☐

**31. How often do you trim all the visible fat off the meat you eat?**	
Always	☐
Usually	☐
Occasionally	☐
Rarely	☐
Never	☐
I don't eat meat	☐

**32. How many times a week do you eat meat pies, pasties or sausage rolls?**	
3 or more times a week	☐
Once or twice a week	☐
Once a fortnight	☐
Less than once a fortnight	☐
Never	☐

**33. How often do you (or the person who cooks for you) remove the skin from chicken before it is cooked?**	
Always	☐
Usually	☐
Occasionally	☐
Rarely	☐
Never	☐
I don’t eat chicken	☐

**34. How many days a week do you eat fried potato (e.g. hot chips or potato crisps)?**	
6 or more days a week	☐
3-5 days a week	☐
1-2 days a week	☐
Less than one day a week	☐
Never	☐

35. How many days a week do you eat take-away foods such as: fried or BBQ chicken; fish and chips; Chinese; pizza; hamburger etc.?

6 or more days a week	☐
3-5 days a week	☐
1-2 days a week	☐
Less than one day a week	☐
Never	☐

36. How often do you (or the person who cooks for you) use fat when cooking? (e.g. butter, margarine, oil, lard etc)

Always	☐
Usually	☐
Occasionally	☐
Rarely	☐
Never	☐

37. How often do you choose wholemeal spaghetti or pasta in preference to regular spaghetti or pasta?

Always	☐
Usually	☐
Occasionally	☐
Rarely	☐
Never	☐
I don’t eat spaghetti/pasta	☐

38. How often do you choose wholemeal bread in preference to white bread?

Always	☐
Usually	☐
Occasionally	☐
Rarely	☐
Never	☐

39. How many days a week do you eat legumes? (e.g. baked beans, three bean mix, lentils, split peas, dried beans etc)

4 or more days a week	☐
2 or 3 days a week	☐
Once a week	☐
Less than once a week	☐
Never	☐

**40. How many days a week do you eat a high fibre breakfast cereal? (e.g. Weetabix, All-Bran, untoasted muesli, porridge)**	
6 or more days a week	☐
3-5 days a week	☐
1-2 days a week	☐
Less than one day a week	☐
Never	☐

1065

**41. How many different types of vegetables would you eat on a typical day?**	
5 or more types	☐
4 types	☐
3 types	☐
1 or 2 types	☐
None	☐

1066

**42. How many days a week do you eat high fat cheeses? (e.g. cheddar or cream cheese)**	
6 or more days a week	☐
3-5 days a week	☐
1-2 days a week	☐
Less than one day a week	☐
Never	☐

1067

**43. How often do you choose low-fat milk (semi-skimmed or skimmed) in preference to whole milk?**	
Always	☐
Usually	☐
Occasionally	☐
Rarely	☐
Never	☐
I don't drink milk	☐

1068

**44. How many days a week do you eat processed meats? (e.g. bacon, salami, ham etc)**	
4 or more days a week	☐
2 or 3 days a week	☐
Once a week	☐
Less than one day a week	☐
Never	☐

**45. How often do you eat or drink any of the following?** *(Please tick (✓)* ***one*** *box for each row).*	**More than once a day**	**Once a day**	**3-6 days a week**	**1-2 days a week**	**less than weekly**	**Never**
Sweets, chocolate bars or biscuits (including wrapped chocolate biscuits like Twix or KitKat)	☐	☐	☐	☐	☐	☐
Buns, cakes or pastries or desserts	☐	☐	☐	☐	☐	☐
Fizzy drinks or squashes that contain sugar (e.g. coca cola, Ribena, Club Orange)	☐	☐	☐	☐	☐	☐
Diet drinks (e.g. Diet Coke, Sprite Zero, Diet Club)	☐	☐	☐	☐	☐	☐

**46. How often do you have a drink containing alcohol?**	
Never	☐
Monthly or less	☐
2 - 4 times a month	☐
2 - 3 times a week	☐
4 or more times a week	☐

**47. Do you keep a record of what you eat and drink? (e.g. writing it down, using an App etc).**	
Yes	☐
No	☐

**48. Do you set yourself food and drink related goals?** *(Please tick (✓)* ***one*** *box).*	
Yes	☐
No	☐

Section 8: Physical activity

We are interested in finding out about the kinds of physical activities that people do as part of their everyday lives. The questions will ask you about the time you spent being physically active **in the last 7 days**. Please answer each question even if you do not consider yourself to be an active person. Please think about the activities you do at work, as part of your house and yard work, to get from place to place, and in your spare time for recreation, exercise or sport.

VIGOROUS ACTIVITIES

Think about all the **vigorous** activities that you did **in the last 7 days**. **Vigorous** physical activities refer to activities that take hard physical effort and make you breathe much harder than normal. Think *only* about those physical activities that you did for at least 10 minutes at a time.

1. During the last 7 days, on how many days did you do vigorous physical activities like heavy lifting, digging, aerobics, or fast bicycling?

________	Days per week	
☐	No vigorous physical activities	→ ***Skip to question 3***

2. How much time did you usually spend doing vigorous physical activities on one of those days?
*(Please answer in hours **AND** minutes. E.g. If you did 30 minutes of vigorous exercise, you can enter '0' in the Hours box and '30' in the Minutes box)*

________	Hours per day
________	Minutes per day
☐	Don't know/not sure

MODERATE ACTIVITIES

Think about all the **moderate** activities that you did **in the last 7 days**. **Moderate** activities refer to activities that take moderate physical effort and make you breathe somewhat harder than normal. Think only about those physical activities that you did for at least 10 minutes at a time.

3. During the last 7 days, on how many days did you do moderate physical activities like carrying light loads, bicycling at a regular pace, or doubles tennis? Do not include walking.

_____ Days per week

☐ No moderate physical activities ⟶ ***Skip to question 5***

4. How much time did you usually spend doing moderate physical activities on one of those days?

*(Please answer in hours **AND** minutes. E.g. If you did 30 minutes of moderate exercise, you can enter '0' in the Hours box and '30' in the Minutes box)*

_____ Hours per day

_____ Minutes per day

☐ Don't know/not sure

WALKING

Think about the time you spent **walking in the last 7 days**. This includes at work and at home, walking to travel from place to place, and any other walking that you have done solely for recreation, sport, exercise, or leisure.

5. During the last 7 days, on how many days did you walk for at least 10 minutes at a time?

_____ Days per week

☐ No walking ⟶ ***Skip to question 7***

6. How much time did you usually spend walking on one of those days?

*(Please answer in hours **AND** minutes. E.g. If you walked for 2 hours, you can enter '2' in the Hours box and '0' in the Minutes box)*

_____ Hours per day

_____ Minutes per day

☐ Don't know/not sure

SITTING

The last question is about the time you spent **sitting** on weekdays during the **last 7 days**. Include time spent at work, at home, while doing course work and during leisure time. This may include time spent sitting at a desk, visiting friends, reading, or sitting or lying down to watch television.

**7. During the last 7 days, how much time did you spend sitting on a week day?** *(Please answer in hours **AND** minutes. E.g. If you spent 8 hours sitting, you can enter '8' in the Hours box and '0' in the Minutes box)*	
__________	Hours per day
__________	Minutes per day
☐	Don't know/not sure

STEPS

**8. Do you use a step counter? (e.g. pedometer, App, Fitbit etc)** *(Please tick (✓) **one** box).*	
Yes	☐
No	☐

**9. Do you set yourself physical activity related goals?** *(Please tick (✓) **one** box).*	
Yes	☐
No	☐

**10. Did you have any problem/s that limited your physical activity during the past 3 months?** *(Please tick (✓) **one** box).*		
Yes	☐	If yes, please give details:
No	☐	

Gallagher D, *et al. BMJ Open* 2024; 14:e084075. doi: 10.1136/bmjopen-2024-084075

Section 9: Infant Feeding

The questions in this section are designed to explore how you feed your youngest child. If your most recent pregnancy was a twin/multiple pregnancy, please complete these questions for the youngest twin/multiple.

4. What is your current method of feeding for your youngest child? *(Please tick (✓)* ***one*** *box for each row).*

Breast feeding	☐ yes	☐ no
Infant formula feeding	☐ yes	☐ no
Combined feeding (Breast and infant formula)	☐ yes	☐ no
Follow-on milk	☐ yes	☐ no
Baby is on solids	☐ yes	☐ no

NOTE: If you ticked 'Baby is on solids', **please go to question 2 below.**

If you did not tick 'Baby is on solids', **please go to Section 10 on Page 35.**

5. How often do you usually give your baby these particular TYPES of solid food? *(Please tick (✓)* ***one*** *box for each food).*

	**More than once a day**	**Once a day**	**3 or more times a week**	**Once or twice a week**	**Less than once a week**	**Never**
Breakfast Cereals	☐	☐	☐	☐	☐	☐
Rice or Pasta	☐	☐	☐	☐	☐	☐
Bread	☐	☐	☐	☐	☐	☐
Potatoes	☐	☐	☐	☐	☐	☐
Potato products (incl. chips, waffles, shapes)	☐	☐	☐	☐	☐	☐
Butter/Margarine and other spreads	☐	☐	☐	☐	☐	☐
Red meat	☐	☐	☐	☐	☐	☐
Processed meat (e.g. ham)	☐	☐	☐	☐	☐	☐

Gallagher D, *et al. BMJ Open* 2024; 14:e084075. doi: 10.1136/bmjopen-2024-084075

	**More than once a day**	**Once a day**	**3 or more times a week**	**Once or twice a week**	**Less than once a week**	**Never**
Chicken/other poultry	☐	☐	☐	☐	☐	☐
Fish (incl. tuna)	☐	☐	☐	☐	☐	☐
Eggs	☐	☐	☐	☐	☐	☐
Beans, lentils, chickpeas	☐	☐	☐	☐	☐	☐
Tofu, Quorn, textured vegetable protein	☐	☐	☐	☐	☐	☐
Nuts	☐	☐	☐	☐	☐	☐
Fruit	☐	☐	☐	☐	☐	☐
Vegetables	☐	☐	☐	☐	☐	☐
Cheese, yoghurt, fromage frais	☐	☐	☐	☐	☐	☐
Puddings or desserts	☐	☐	☐	☐	☐	☐
Biscuits, sweets, chocolate or cakes	☐	☐	☐	☐	☐	☐
Crisps and corn snacks	☐	☐	☐	☐	☐	☐
Follow on formula	☐	☐	☐	☐	☐	☐
Or something else (please tick and then write below)	☐	☐	☐	☐	☐	☐

1132

**6. Do you ever add salt to your baby's solid food, including adding salt when the food is being cooked?** *(Please tick (✓)* ***one*** *box).*	
Yes, often	☐
Yes, sometimes	☐
Never	☐

Section 10: Mood

*(Please tick (✓) **one** box for each statement below).*

In the past 7 days…

**9. I have been able to laugh and see the funny side of things.**	
As much as I always could	☐
Not quite so much now	☐
Definitely not so much now	☐
Not at all	☐

**10. I have looked forward with enjoyment to things.**	
As much as I ever did	☐
Rather less than I used to	☐
Definitely less than I used to	☐
Hardly at all	☐

**11. I have blamed myself unnecessarily when things went wrong.**	
Yes, most of the time	☐
Yes, some of the time	☐
Not very often	☐
No, never	☐

**12. I have been anxious or worried for no good reason.**	
No, not at all	☐
Hardly ever	☐
Yes, sometimes	☐
Yes, very often	☐

**13. I have felt scared or panicky for no very good reason.**	
Yes, quite a lot	☐
Yes, sometimes	☐
No, not much	☐
No, not at all	☐

**14. Things have been getting on top of me.**	
Yes, most of the time I haven't been able to cope at all	☐
Yes, sometimes I haven't been coping as well as usual	☐
No, most of the time I have coped quite well	☐
No, I have been coping as well as ever	☐

**15. I have been so unhappy that I have had difficulty sleeping.**	
Yes, most of the time	☐
Yes, sometimes	☐
Not very often	☐
No, not at all	☐

**16. I have felt sad or miserable.**	
Yes, most of the time	☐
Yes, quite often	☐
Not very often	☐
No, not at all	☐

**9. I have been so unhappy that I have been crying.**	
Yes, most of the time	☐
Yes, quite often	☐
Only occasionally	☐
No, never	☐

**10. The thought of harming myself has occurred to me.**	
Yes, quite often	☐
Sometimes	☐
Hardly ever	☐
Never	☐

Gallagher D, *et al. BMJ Open* 2024; 14:e084075. doi: 10.1136/bmjopen-2024-084075

*(Please tick (✓) **one** box for each statement below).*

Over the last two weeks, how often have you been bothered by the following problems?

**18. Feeling nervous, anxious, or on edge?**	
Not at all	☐
Several days	☐
More than half of the days	☐
Nearly every day	☐

**19. Not being able to stop or control worrying?**	
Not at all	☐
Several days	☐
More than half of the days	☐
Nearly every day	☐

**20. Worrying too much about different things?**	
Not at all	☐
Several days	☐
More than half of the days	☐
Nearly every day	☐

**21. Trouble relaxing?**	
Not at all	☐
Several days	☐
More than half of the days	☐
Nearly every day	☐

**22. Being so restless that it is hard to sit still?**	
Not at all	☐
Several days	☐
More than half of the days	☐
Nearly every day	☐

Gallagher D, *et al. BMJ Open* 2024; 14:e084075. doi: 10.1136/bmjopen-2024-084075

**23. Becoming easily annoyed or irritable?**	
Not at all	☐
Several days	☐
More than half of the days	☐
Nearly every day	☐

**24. Feeling afraid as if something awful might happen?**	
Not at all	☐
Several days	☐
More than half of the days	☐
Nearly every day	☐

Having a young baby or child can bring up lots of different feelings and emotions. The next questions are designed to explore how you are feeling about being a parent to your baby/child. Answering these questions will help us to understand how things are going for you. If your most recent pregnancy was a twin/multiple pregnancy, please complete these questions for the youngest twin/multiple.

**25. Has your child reached their second birthday?** *(Please tick (✓) **one** box).*		
No, my child has not yet had their second birthday	☐	→ Please go to question 19 on page 39.
Yes, my child has celebrated their second birthday	☐	→ Please go to question 21 on Page 41.

19. Thinking about your feelings about your baby, choose the response for each statement that feels right to you. *(Please tick (✓)* ***one*** *box for each of the following statements).*

	**Never**	**Sometimes**	**Always**
I enjoy looking after my baby	☐	☐	☐
I feel irritated with my baby when we are together	☐	☐	☐
I feel affectionate towards my baby	☐	☐	☐
I feel that my baby is being difficult or trying to upset me on purpose	☐	☐	☐
I can work out what my baby needs from me	☐	☐	☐
I feel like I can't do things I enjoy because of my baby.	☐	☐	☐
I feel the changes in my life are worth it to look after my baby.	☐	☐	☐
I miss my baby when we are not together	☐	☐	☐
I feel like I'm looking after my baby for someone else.	☐	☐	☐
When we've been apart I look forward to seeing my baby again.	☐	☐	☐
I enjoy playing with my baby.	☐	☐	☐

20. Thinking about your baby, please tick one of the choices for each of the questions below. There are no 'right' or 'wrong' answers; many of these are

true of all babies at times. *(Please tick (✓)* ***one*** *box for each of the following statements).*

	**Always**	**Very often**	**Quite often**	**Sometimes**	**Rarely**	**Never**
My baby smiles at me.	☐	☐	☐	☐	☐	☐
My baby annoys me.	☐	☐	☐	☐	☐	☐
My baby likes doing things with me.	☐	☐	☐	☐	☐	☐
My baby 'talks' to me.	☐	☐	☐	☐	☐	☐
My baby irritates me.	☐	☐	☐	☐	☐	☐
My baby likes me.	☐	☐	☐	☐	☐	☐
My baby wants too much attention.	☐	☐	☐	☐	☐	☐
My baby laughs.	☐	☐	☐	☐	☐	☐
My baby gets moody.	☐	☐	☐	☐	☐	☐
My baby dominates me.	☐	☐	☐	☐	☐	☐
My baby likes to please me.	☐	☐	☐	☐	☐	☐
My baby cries for no obvious reason.	☐	☐	☐	☐	☐	☐
My baby is affectionate towards me.	☐	☐	☐	☐	☐	☐
My baby winds me up.	☐	☐	☐	☐	☐	☐

Please go to section 11 on Page 43

Gallagher D, *et al. BMJ Open* 2024; 14:e084075. doi: 10.1136/bmjopen-2024-084075

21. Thinking about your feelings about your child, choose the response for each statement that feels right to you. *(Please tick (✓)* ***one*** *box for each of the following statements).*

	Never	Sometimes	Always
I enjoy looking after my child	☐	☐	☐
I feel irritated with my child when we are together	☐	☐	☐
I feel affectionate towards my child	☐	☐	☐
I feel that my child is being difficult or trying to upset me on purpose	☐	☐	☐
I can work out what my child needs from me	☐	☐	☐
I feel like I can’t do things I enjoy because of my child.	☐	☐	☐
I feel the changes in my life are worth it to look after my child.	☐	☐	☐
I miss my child when we are not together	☐	☐	☐
I feel like I’m looking after my child for someone else.	☐	☐	☐
When we’ve been apart I look forward to seeing my child again.	☐	☐	☐
I enjoy playing with my child.	☐	☐	☐

22. Thinking about your child, please tick one of the choices for each of the questions below. There are no ‘right’ or ‘wrong’ answers; many of these are

true of all children at times. *(Please tick (✓)* ***one*** *box for each of the following statements).*

	Always	Very often	Quite often	Sometimes	Rarely	Never
My child smiles at me.	☐	☐	☐	☐	☐	☐
My child annoys me.	☐	☐	☐	☐	☐	☐
My child likes doing things with me.	☐	☐	☐	☐	☐	☐
My child talks to me.	☐	☐	☐	☐	☐	☐
My child irritates me.	☐	☐	☐	☐	☐	☐
My child likes me.	☐	☐	☐	☐	☐	☐
My child wants too much attention.	☐	☐	☐	☐	☐	☐
My child laughs.	☐	☐	☐	☐	☐	☐
My child gets moody.	☐	☐	☐	☐	☐	☐
My child dominates me.	☐	☐	☐	☐	☐	☐
My child likes to please me.	☐	☐	☐	☐	☐	☐
My child cries for no obvious reason.	☐	☐	☐	☐	☐	☐
My child is affectionate towards me.	☐	☐	☐	☐	☐	☐
My child winds me up.	☐	☐	☐	☐	☐	☐

Section 11: Self-esteem

Below is a list of statements dealing with your general feelings about yourself.

*(Please tick (✓) **one** box to indicate how much you agree or disagree with each of the following statements).*

	**Strongly Agree**	**Agree**	**Disagree**	**Strongly disagree**
I feel that I'm a person of worth, at least on an equal plane with others.	☐	☐	☐	☐
I feel that I have a number of good qualities.	☐	☐	☐	☐
All in all, I am inclined to feel that I am a failure.	☐	☐	☐	☐
I am able to do things as well as most other people.	☐	☐	☐	☐
I feel I do not have much to be proud of.	☐	☐	☐	☐
I take a positive attitude toward myself.	☐	☐	☐	☐
On the whole I am satisfied with myself.	☐	☐	☐	☐
I wish I could have more respect for myself.	☐	☐	☐	☐
I certainly feel useless at times.	☐	☐	☐	☐
At times I think I am no good at all.	☐	☐	☐	☐

Section 12: Sleep

1. At the moment, how often does your baby sleep all night? *(Please tick (✓)* ***one*** *box).*

Always	☐
Mostly	☐
Sometimes	☐
Rarely	☐
Never	☐

The following questions relate to **your** usual sleep habits during the **past month only**. Your answers should indicate the most accurate reply for the majority of days and nights in the past month. Please answer all questions.

2. During the past month, what time have you usually gone to bed at night?

Usual bed time ☐

3. During the past month, how long (in minutes) has it usually taken you to fall asleep each night?

Number of minutes ☐

4. During the past month, what has been your usual getting up time in the morning?

Usual getting up time ☐

5. During the past month, on average how many hours of actual sleep did you get at night? (This may be different than the number of hours you spent in bed.)

Hours of sleep per night ☐

Gallagher D, *et al. BMJ Open* 2024; 14:e084075. doi: 10.1136/bmjopen-2024-084075

6. During the past month, how often have you had trouble sleeping because you…

*(Please tick (✓) **one** box for each statement).*

	Not during the past month	Less than once a week	Once or twice a week	Three or more times a week
Cannot get to sleep within 30 minutes	☐	☐	☐	☐
Wake up in the middle of the night or early morning	☐	☐	☐	☐
Have to get up to use the bathroom	☐	☐	☐	☐
Cannot breathe comfortably	☐	☐	☐	☐
Cough or snore loudly	☐	☐	☐	☐
Feel too cold	☐	☐	☐	☐
Feel too hot	☐	☐	☐	☐
Had bad dreams	☐	☐	☐	☐
Have pain	☐	☐	☐	☐
Have to take care of your baby	☐	☐	☐	☐
Other reason(s), please describe and indicate how often during the past month you had trouble sleeping because of this:				
______	☐	☐	☐	☐
______	☐	☐	☐	☐
______	☐	☐	☐	☐

1241
1242

7. During the past month, how would you rate your sleep quality overall? *(Please tick (✓) **one** box).*

Very Good	Fairly Good	Fairly Bad	Very Bad

☐	☐	☐	☐

**8**. (Please tick *(✓)* ***one*** *box for each question below.)*	**Not during the past month**	**Less than once a week**	**Once or twice a week**	**Three or more times a week**
**During the past month, how often have you taken medicine (prescribed or "over the counter") to help you sleep?**	☐	☐	☐	☐
**During the past month, how often have you had trouble staying awake while driving, eating meals, or engaging in social activity?**	☐	☐	☐	☐

9. During the past month, how much of a problem has it been for you to keep up enough enthusiasm to get things done? (Please tick *(✓)* ***one*** *box).*

**No problem at all**	**Only a very slight problem**	**Somewhat of a problem**	**A very big problem**
☐	☐	☐	☐

10. Do you usually have a bed partner or room-mate? *(Please tick (✓)* ***one*** *box).*

**No bed partner or room-mate**	**Partner/room-mate in other room**	**Partner in same room but not in same bed**	**Partner in same bed**
☐	☐	☐	☐

11. If you usually have a room-mate or bed partner, ask him/her how often in the past month you have had:

Please tick (✓) ***one*** *box for each statement).*	**Not during the past month**	**Less than once a week**	**Once or twice a week**	**Three or more times a week**
Loud snoring	☐	☐	☐	☐
Long pauses between breaths while asleep	☐	☐	☐	☐
Legs twitching or jerking while you sleep	☐	☐	☐	☐
Episodes of confusion during sleep	☐	☐	☐	☐
Other restlessness while you sleep, please describe and indicate how often in the past month you have had this:				
______	☐	☐	☐	☐
______	☐	☐	☐	☐
______	☐	☐	☐	☐

You have now completed the questionnaire. Thank you very much for your time and effort! We really appreciate your help with our research.

Supporting MumS (SMS) study Questionnaire Booklet

Month 12 Control Group

**Participant ID:**	_ _ _ _ _
**Researcher name:**	
**Today's date:**	_ _ / _ _ / _ _ _ _<br>**e.g. 05 / 01 / 2017**

About this questionnaire booklet

Thank you for taking the time to fill out this questionnaire booklet.

The questionnaire booklet is made up of questions that will help us find out more about your experience of the postpartum period. Please complete the questions as accurately as you can. **There are no right or wrong answers**. If there are any questions you do not wish to answer, you can leave them blank. Note that your responses will be **anonymous,** this means your name will never be given with your responses.

The questionnaire booklet is split up into the short sections shown below. You can fill it all out at once, or do it in chunks, as your time allows.

**Section 1**	**SMS evaluation**	**Pages 3-6**
**Section 2**	**Health and well-being**	**Pages 7-9**
**Section 3**	**Health resources**	**Pages 10-12**
**Section 4**	**Eating and activity approaches**	**Pages 13-17**
**Section 5**	**Social support**	**Page 18-19**
**Section 6**	**Lifestyle behaviours and attitudes**	**Page 20-22**
**Section 7**	**Food patterns**	**Page 23-27**
**Section 8**	**Physical activity**	**Page 28-30**
**Section 9**	**Infant feeding**	**Page 31-32**
**Section 10**	**Mood**	**Page 33-40**
**Section 11**	**Self-esteem**	**Page 41**
**Section 12**	**Sleep**	**Page 42-45**

Section 1: SMS Evaluation

Thank you for participating in our study. We would like to ask you some questions about how you have found participating in this research. Your responses to this survey will help us to improve the studies we carry out in the future.

3. How satisfied were you with your overall experience of the SMS study? *(Please tick (✓) **one** box).*

Very Dissatisfied	Mostly Dissatisfied	Neither Satisfied Nor Dissatisfied	Mostly Satisfied	Very Satisfied
☐	☐	☐	☐	☐

If you were very or mostly dissatisfied, please explain what could have been improved:

____

____

____

4. Please answer the questions below about your experience of taking part in the SMS study. *(Please tick (✓) **one** box to indicate how much you agree or disagree with each of the following statements).*

	Strongly disagree	Disagree	Neither agree or disagree	Agree	Strongly Agree
At the start of the study, the information I was given about the study was clear and informative	☐	☐	☐	☐	☐
The amount of information and level of support provided by the researchers during the study was suited to my needs	☐	☐	☐	☐	☐

If you have disagreed or strongly disagreed with any of the above statements, please explain what could have been improved:

____

____

____

3. How satisfied were you with the text messages? *(Please tick (✓) **one** box).*

Very Dissatisfied	Mostly Dissatisfied	Neither Satisfied Nor Dissatisfied	Mostly Satisfied	Very Satisfied
☐	☐	☐	☐	☐

1331

If you were very or mostly dissatisfied, please explain what could have been improved:

____

____

____

1332

13. Please answer the questions below about your experience of the text messages you received during the SMS study. *(Please tick (✓) **one** box to indicate how much you agree or disagree with each of the following statements).*

	Strongly Disagree	Disagree	Neither agree or disagree	Agree	Strongly agree
SMS texts were easy to understand	☐	☐	☐	☐	☐
SMS texts were helpful	☐	☐	☐	☐	☐
SMS texts were interesting	☐	☐	☐	☐	☐
An appropriate amount of SMS texts were sent during the study	☐	☐	☐	☐	☐
SMS texts were delivered at appropriate times of the day	☐	☐	☐	☐	☐

1333

If you have disagreed or strongly disagreed with any of the above statements, please explain what could have been improved:

____

____

____

1334

14. During the study you were asked to attend 3 study visits. Please rate how easy or difficult you found each aspect of these visits. *(Please tick (✓) **one** response that most applies to you in relation to your experience).*

	**Very difficult**	**Difficult**	**No strong opinion**	**Easy**	**Very easy**
Location of visits	☐	☐	☐	☐	☐
Length of visits	☐	☐	☐	☐	☐
Having your height measurement taken	☐	☐	☐	☐	☐
Having your waist measurements taken	☐	☐	☐	☐	☐
Having your weight taken	☐	☐	☐	☐	☐
Completing the questionnaires	☐	☐	☐	☐	☐

If you have felt that any aspects of the visits were difficult, please explain how they could have been improved:

____

____

____

Gallagher D, *et al. BMJ Open* 2024; 14:e084075. doi: 10.1136/bmjopen-2024-084075

15. Based on your experience in taking part in SMS, do you think the programme should be offered to other mums? *(Please tick (✓)* ***one*** *response).*

Yes	☐
No	☐

If you answered no, please explain why:

____

____

____

16. Do you have any other comments? We would welcome any suggestions you have regarding how we could improve future studies for those taking part:

____

____

____

Section 2: Health and well-being

For questions 1-5, under each heading, please tick (✓) the **ONE** box that best describes your health TODAY.

**1. Mobility**	
I have no problems in walking about	☐
I have slight problems in walking about	☐
I have moderate problems in walking about	☐
I have severe problems in walking about	☐
I am unable to walk about	☐

**2. Self-care**	
I have no problems washing or dressing myself	☐
I have slight problems washing or dressing myself	☐
I have moderate problems washing or dressing myself	☐
I have severe problems washing or dressing myself	☐
I am unable to wash or dress myself	☐

**3. Usual Activities** *(e.g. work, study, housework, family or leisure activities)*	
I have no problems doing my usual activities	☐
I have slight problems doing my usual activities	☐
I have moderate problems doing my usual activities	☐
I have severe problems doing my usual activities	☐
I am unable to do my usual activities	☐

**4. Pain/ Discomfort**	
I have no pain or discomfort	☐
I have slight pain or discomfort	☐
I have moderate pain or discomfort	☐
I have severe pain or discomfort	☐
I have extreme pain or discomfort	☐

**5. Anxiety/ Depression**	
I am not anxious or depressed	☐
I am slightly anxious or depressed	☐
I am moderately anxious or depressed	☐
I am severely anxious or depressed	☐
I am extremely anxious or depressed	☐

6. We would like to know how good or bad your health is TODAY.

This scale is numbered from 0 to 100.

100 means the <u>best</u> health you can imagine.

0 means the <u>worst</u> health you can imagine.

Please mark an X on the scale to indicate how your health is TODAY.

Now, write the number you marked on the scale in the box below.

Your health today = ☐

The best health you can imagine

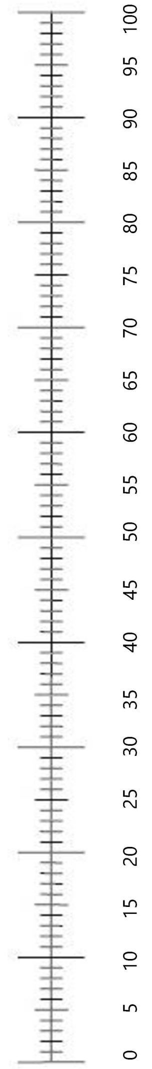

The worst health you can imagine

115

**7. Feeling settled and secure** *(Please tick (✓)* ***one*** *box that best describes your overall quality of life at the moment).*	
I am able to feel settled and secure in **all** areas of my life	☐
I am able to feel settled and secure in **many** areas of my life	☐
I am able to feel settled and secure in **a few** areas of my life	☐
I am unable to feel settled and secure in **any** areas of my life	☐

**8. Love, friendship and support** *Please tick (✓)* ***one*** *box that best describes your overall quality of life at the moment).*	
I can have **a lot** of love, friendship and support	☐
I can have **quite a lot** of love, friendship and support	☐
I can have **a little** love, friendship and support	☐
I **cannot** have **any** love, friendship and support	☐

**9. Being independent** *Please tick (✓)* ***one*** *box that best describes your overall quality of life at the moment).*	
I am able to be **completely** independent	☐
I am able to be independent in **many** things	☐
I am able to be independent in **a few** things	☐
I am **unable** to be at all independent	☐

**10. Achievement and progress** *Please tick (✓)* ***one*** *box that best describes your overall quality of life at the moment).*	
I can achieve and progress in **all** aspects of my life	☐
I can achieve and progress in **many** aspects of my life	☐
I can achieve and progress in **a few** aspects of my life	☐
I cannot achieve and progress in **any** aspects of my life	☐

**11. Enjoyment and pleasure** *Please tick (✓)* ***one*** *box that best describes your overall quality of life at the moment).*	
I can have **a lot** of enjoyment and pleasure	☐
I can have **quite a lot** of enjoyment and pleasure	☐
I can have **a little** enjoyment and pleasure	☐
I **cannot** have **any** enjoyment and pleasure	☐

Section 3: Health resources

2. 1. In the last 3 months, have you seen <u>any</u> health professional at your GP surgery? (this includes phone/online or face-to-face appointments) (*Please tick (✓)* ***one*** *box).*

Yes	☐
No	☐

If YES, <u>how many times</u> were you seen by: *(Please enter a number)*

GP	☐
Practice nurse	☐
Other (please state)	☐
Other (please state)	☐

2. In the last 3 months, have you attended an Accident and Emergency (Casualty) department? *(Please tick (✓)* ***one*** *box).*

Yes	☐	If YES, how many <u>times?</u>	☐
No	☐		

3. In the last 3 months, have you attended hospital as an <u>out-patient?</u> *(Please tick (✓)* ***one*** *box).*

Yes	☐	If YES, how many <u>times?</u>	☐
No	☐		

4. In the last 3 months, have you attended hospital as an <u>in-patient?</u> *(Please tick (✓)* ***one*** *box).*

Yes	☐	If YES, how many <u>times?</u>	☐
		If YES, how many nights did you spend in hospital in the last 3 months in total?	☐
No	☐		

5. In the last 3 months, have you received any prescriptions for medicine? *(Please tick (✓)* ***one*** *box)***.**

Yes	☐	If YES, how many prescriptions have you received?	☐
No	☐		

6. In the last 3 months, have you purchased any over-the-counter medications or lifestyle products (e.g. slimming aids, vitamin supplements)? *(Please tick (✓) **one** box).*

Yes	☐	If YES, please state total amount spent in last 3 months	£
No	☐		

1374

7. In the last 3 months have you paid for any services or clubs for the specific purpose of helping you with your lifestyle - for example slimming clubs, health clubs, gyms, swimming pools, exercise classes? *(Please tick (✓) **one** box).*

Yes	☐	If YES, approximately how much have you spent in <u>total</u> on all of these services/clubs in the <u>last 3 months?</u>	£
No	☐		

8. Please think about <u>last week's</u> food and drink purchases for your household (i.e. you and your family) and tell us the cost to your household of the following:
*(Please write '**0**' if you don't purchase a specific type of food/product) .*

Type of food and drink purchased	Weekly Cost to you (your household)
Food and non-alcoholic drinks (e.g. supermarket shopping)	£
Alcoholic drinks e.g. wine & beer	£
Takeaway meals and snacks eaten AT HOME e.g. pizza delivery	£
Meals, snacks and drinks CONSUMED AWAY FROM HOME (e.g. restaurant)	£
Cigarettes or vaping products	£

1375

9. Have you previously purchased any apps for improving your fitness/health or wellbeing? *(Please tick (✓) **one** box).*

Yes	☐	If YES, Please state the total amount you spent on fitness/health apps in the <u>last year?</u>	£
No	☐		

1376

10. On average, <u>per day</u> how much time have you spent exercising/walking in the last 3 months in total?

*(Please answer in hours **AND** minutes. E.g. If you did 30 minutes of exercise per day, you can enter '0' under Hours and '30' under the Minutes column)*

On average per day: Hours [ ] Minutes [ ]

11. Has your employment status changed in the last three months?
(Note: returning to work after maternity leave should **not** be included as a change) *(Please tick (✓) **one** box).*

Yes ☐

No ☐

If YES, what is your **current** employment status? *(Please tick (✓) **one** box)*

☐ Full-time employment
☐ Part-time employment
☐ Unemployed
☐ Student or training
☐ Retired
☐ Not working due to illness
☐ Prefer not to answer
☐ Other (please specify) [ ]

Section 4: Eating and activity approaches

This section asks you about your diet. *(Please circle **one** number in each row to indicate how much you agree or disagree with the following statements).*

**1. Eating healthier is something…**	**Strongly Disagree**						**Strongly Agree**
I do automatically	1	2	3	4	5	6	7
I do without having to consciously remember	1	2	3	4	5	6	7
I do without thinking	1	2	3	4	5	6	7
I start doing before I realise I'm doing it	1	2	3	4	5	6	7

**2. Over the next few months I intend to…**	**Strongly Disagree**						**Strongly Agree**
eat a healthy diet (e.g. reduce portion size, eat less sugary and high fact snacks and eat more fruit and vegetables)	1	2	3	4	5	6	7
be physically active (e.g. by walking more or taking exercise classes)	1	2	3	4	5	6	7

**3. I already have concrete plans…**	**Strongly Disagree**						**Strongly Agree**
on how to eat a healthy diet	1	2	3	4	5	6	7
on when to eat a healthy diet	1	2	3	4	5	6	7

**4. I already have concrete plans…**	**Strongly Disagree**						**Strongly Agree**
on when I need to be especially careful to maintain my healthy diet	1	2	3	4	5	6	7
about what to do in difficult situations to stick to my healthy diet	1	2	3	4	5	6	7
on how I will cope if I slip back into old eating habits	1	2	3	4	5	6	7

**5. I am confident that I can stick to a healthy diet even…**	**Strongly Disagree**						**Strongly Agree**
if I have to learn a lot about nutrition	1	2	3	4	5	6	7
if I initially have to watch out in many situations	1	2	3	4	5	6	7
if I have to start all over again several times until I succeed	1	2	3	4	5	6	7
if I initially have to make plans	1	2	3	4	5	6	7
if initially food doesn’t taste as good	1	2	3	4	5	6	7
if I initially don’t get much support	1	2	3	4	5	6	7
if it takes a long time to get used to it	1	2	3	4	5	6	7
if I have worries and troubles	1	2	3	4	5	6	7
if my partner/ my family don’t change their nutrition habits	1	2	3	4	5	6	7
if I am tired	1	2	3	4	5	6	7
if I am stressed out	1	2	3	4	5	6	7
if I don't lose weight initially	1	2	3	4	5	6	7

6. In spite of good intentions when losing weight, slip-ups may occur. Imagine you relapse back into your old eating habits. How confident are you about restarting your new healthy eating habits?

**I am sure I can start my new healthy eating habits again regularly, even if I…**	**Strongly Disagree**						**Strongly Agree**
have several bad days in a row	1	2	3	4	5	6	7
lose my motivation to keep up my new healthy eating habits	1	2	3	4	5	6	7
have had a relapse and have fallen back into old eating habits for several weeks	1	2	3	4	5	6	7

7. For the next few questions:

'Tempting foods' are any food you want to eat more of than you think you should.
'Eating intentions' refer to the way you are aiming to eat, for example you may intend to avoid tempting foods or eat healthy foods.

*(Please tick (✓) **one** box for each of the following statements).*	**Never**	**Rarely**	**Sometimes**	**Often**	**Always**
I give up too easily on my eating intentions	☐	☐	☐	☐	☐
I'm good at resisting tempting food	☐	☐	☐	☐	☐
I easily get distracted from the way I intend to eat	☐	☐	☐	☐	☐
If I am not eating in the way I intend to I make changes	☐	☐	☐	☐	☐
I find it hard to remember what I have eaten throughout the day	☐	☐	☐	☐	☐

<u>This section asks about your physical activity</u>. *(Please circle **one** number in each row to indicate how much you agree or disagree with the following statements).*

**1. Being active every day is something…**	**Strongly Disagree**						**Strongly Agree**
I do automatically	1	2	3	4	5	6	7
I do without having to consciously remember	1	2	3	4	5	6	7
I do without thinking	1	2	3	4	5	6	7
I start doing before I realise I'm doing it	1	2	3	4	5	6	7

**2. I already have concrete plans…**	**Strongly Disagree**						**Strongly Agree**
on when to be physically active	1	2	3	4	5	6	7
on where to be physically active	1	2	3	4	5	6	7
on how to be physically active	1	2	3	4	5	6	7
on how often be physically active	1	2	3	4	5	6	7
on who I can be physically active with	1	2	3	4	5	6	7
**3. I already have concrete plans…**	**Strongly Disagree**						**Strongly Agree**

BMJ Publishing Group Limited (BMJ) disclaims all liability and responsibility arising from any reliance placed on this supplemental material which has been supplied by the author(s)

about what to do if something gets in the way	1	2	3	4	5	6	7
about what to do if I miss a physical activity session	1	2	3	4	5	6	7
about what to do in difficult situations to stick to my physical activity intentions	1	2	3	4	5	6	7
for times when I will need to be extra careful to stay committed	1	2	3	4	5	6	7

4a. Certain barriers make it hard to begin physical activity. How sure are you that you can begin exercising regularly?

**I am sure that...**	**Strongly Disagree**						**Strongly Agree**
I can change to a physically active lifestyle	1	2	3	4	5	6	7
I can be physically active once a week	1	2	3	4	5	6	7
I can be physically active at least 3 times a week for 30 minutes	1	2	3	4	5	6	7

**4b. I am sure I can start being physically active immediately, even if...**	**Strongly Disagree**						**Strongly Agree**
the planning for this takes a lot of time and effort	1	2	3	4	5	6	7
I have to force myself to start immediately	1	2	3	4	5	6	7
I have to push myself	1	2	3	4	5	6	7

5. Are you confident that you can manage staying physically active?

**I am sure I can keep being physically active regularly, even if…**	**Strongly Disagree**						**Strongly Agree**
it takes me a long time to make a habit	1	2	3	4	5	6	7
I am worried and troubled	1	2	3	4	5	6	7
I am tired	1	2	3	4	5	6	7
I am stressed out	1	2	3	4	5	6	7
I don't lose weight at once	1	2	3	4	5	6	7
I have to start all over again several times until I succeed	1	2	3	4	5	6	7
my partner/family isn't physically active	1	2	3	4	5	6	7

6. In spite of good intentions, slip ups may occur. How confident are you about restarting exercises?

**I am sure I can keep being physically active regularly, even if…**	**Strongly Disagree**						**Strongly Agree**
I postpone my plans several times	1	2	3	4	5	6	7
I lose my motivation	1	2	3	4	5	6	7
I have not been active for several weeks	1	2	3	4	5	6	7

Section 5: Social Support

1. Below is a list of statements about social support. *(Please tick (✓)* ***one*** *box in each row to indicate how much you agree or disagree with the following statements).*
Within the past 3 months, I have got support from my friends and/or family to help me:

	Strongly Agree	Agree	Neither agree or disagree	Disagree	Strongly disagree
…eat well	☐	☐	☐	☐	☐
…be physically active	☐	☐	☐	☐	☐
…lose weight	☐	☐	☐	☐	☐

2. How often have you felt lonely during the past week?
(Please tick (✓) ***one*** *box).*

None, or almost none of the time	☐
Some of the time	☐
Most of the time	☐
All, or almost all of the time	☐
Don't know	☐
Do not wish to answer	☐

3. How many people can you count on in times of need?
(Please tick (✓) ***one*** *box).*

0	☐	→ Please go to Section 6 on Page 20
1	☐	
2	☐	
3	☐	
4	☐	
5	☐	→ Please complete the next question in this section (i.e. question 4)
6	☐	
7	☐	
8	☐	
9	☐	
10 or more	☐	

4. How many of these people live in your local area? That is, within about a mile or a 20 minute walk from your home.
*(Please tick (✓) **one** box).*

0	☐
1	☐
2	☐
3	☐
4	☐
5	☐
6	☐
7	☐
8	☐
9	☐
10 or more	☐

Section 6: Lifestyle behaviours and attitudes

7. How often do you weigh yourself currently? *(Please tick (✓) **one** box).*

Never	☐
About once a year or less	☐
Every couple of months	☐
Every month	☐
Every week	☐
Every day	☐
More than once a day	☐

8. Within the past 3 months, have you taken part in any weight loss programmes (e.g. Slimming World). *(Please tick (✓) **one** box).*

Yes	☐
No	☐

If yes, please give details below;

Name of programme	*How long you attended for*

9. Do you want to lose weight? *(Please tick (✓) **one** box).*

Yes	☐	→ Please complete the rest of the questions in this section (i.e. questions 4, 5 & 6)
No	☐	→ Please go to Section 7 on Page 23

4. How confident are you in your ability to:

*(Please circle **one** number in each row to indicate how confident you feel about each of the following statements).*	**Not confident**						**Very confident**
Lose weight	1	2	3	4	5	6	7
Keep lost weight off in the long term	1	2	3	4	5	6	7
Stay at current weight	1	2	3	4	5	6	7

5. How important is losing weight for you at the moment? *(Please circle **one** number).*

	Not important						Very important
	1	2	3	4	5	6	7

6. The questions below ask you about why you want to lose weight. *(Please tick (✓) **one** box for each statement).*

I want to lose weight…

	Absolutely not	Somewhat	Moderately	Strongly
Because it is commonly said that living with overweight is unhealthy	☐	☐	☐	☐
To be healthier	☐	☐	☐	☐
To be more agile	☐	☐	☐	☐
For health reasons	☐	☐	☐	☐
Because I read that it is healthier	☐	☐	☐	☐
To decrease my health risks	☐	☐	☐	☐
To live long	☐	☐	☐	☐
Because acquaintances have advised me to	☐	☐	☐	☐
To not attract attention	☐	☐	☐	☐
Because I'll be more successful in my job	☐	☐	☐	☐
So I will be accepted by society	☐	☐	☐	☐
To dare to socialise again	☐	☐	☐	☐
Because I would be luckier in love	☐	☐	☐	☐
To be more appreciated/liked	☐	☐	☐	☐
To have more friends	☐	☐	☐	☐
To have better success with others	☐	☐	☐	☐
So that other people will think better of me	☐	☐	☐	☐
To be more attractive	☐	☐	☐	☐
To like to look at myself in the mirror again	☐	☐	☐	☐
Because I want to like myself more	☐	☐	☐	☐

**I want to lose weight…**	**Absolutely not**	**Somewhat**	**Moderately**	**Strongly**
Because I want to be more attractive	☐	☐	☐	☐
To be able to dress more fashionably	☐	☐	☐	☐
To fit into my clothes again	☐	☐	☐	☐
To feel more self-confident	☐	☐	☐	☐

Section 7: Food patterns

*(Please tick (✓) **one** box for each of the following questions).*

49. How many times a week do you eat two or more pieces of fruit?

6 or more days a week	☐
3-5 days a week	☐
1-2 days a week	☐
Less than one day a week	☐
Never	☐

50. When eating cheese, how often do you choose reduced fat cheese in preference to regular cheese?

Always	☐
Usually	☐
Occasionally	☐
Rarely	☐
Never	☐
I don't eat cheese	☐

51. How many days a week do you eat fried food with a batter or bread crumb coating?

4 or more days a week	☐
2 or 3 days a week	☐
Once a week	☐
Less than once a week	☐
Never	☐

52. How often do you eat fried or roasted vegetables?

Always	☐
Usually	☐
Occasionally	☐
Rarely	☐
Never	☐

53. When eating bread (as toast, sandwiches or a snack) how often do you spread butter or margarine on it?

Always	☐
Usually	☐
Occasionally	☐
Rarely	☐
Never	☐

1461

**54. How many portions of vegetables do you eat in a typical day? (a portion is about three tablespoons of vegetables or a small bowl of salad).**	
5 or more portions	☐
3 or 4 portions	☐
1 or 2 portions	☐
Less than one portion per day	☐
None	☐

1462

**55. How often do you trim all the visible fat off the meat you eat?**	
Always	☐
Usually	☐
Occasionally	☐
Rarely	☐
Never	☐
I don't eat meat	☐

1463

**56. How many times a week do you eat meat pies, pasties or sausage rolls?**	
3 or more times a week	☐
Once or twice a week	☐
Once a fortnight	☐
Less than once a fortnight	☐
Never	☐

1464

**57. How often do you (or the person who cooks for you) remove the skin from chicken before it is cooked?**	
Always	☐
Usually	☐
Occasionally	☐
Rarely	☐
Never	☐
I don't eat chicken	☐

1465

**58. How many days a week do you eat fried potato (e.g. hot chips or potato crisps)?**	
6 or more days a week	☐
3-5 days a week	☐
1-2 days a week	☐
Less than one day a week	☐
Never	☐

59. How many days a week do you eat take-away foods such as: fried or BBQ chicken; fish and chips; Chinese; pizza; hamburger etc.?

6 or more days a week	☐
3-5 days a week	☐
1-2 days a week	☐
Less than one day a week	☐
Never	☐

1466

60. How often do you (or the person who cooks for you) use fat when cooking? (e.g. butter, margarine, oil, lard etc)

Always	☐
Usually	☐
Occasionally	☐
Rarely	☐
Never	☐

1467

61. How often do you choose wholemeal spaghetti or pasta in preference to regular spaghetti or pasta?

Always	☐
Usually	☐
Occasionally	☐
Rarely	☐
Never	☐
I don’t eat spaghetti/pasta	☐

1468

62. How often do you choose wholemeal bread in preference to white bread?

Always	☐
Usually	☐
Occasionally	☐
Rarely	☐
Never	☐

1469

63. How many days a week do you eat legumes? (e.g. baked beans, three bean mix, lentils, split peas, dried beans etc)

4 or more days a week	☐
2 or 3 days a week	☐
Once a week	☐
Less than once a week	☐
Never	☐

1470

**64. How many days a week do you eat a high fibre breakfast cereal? (e.g. Weetabix, All-Bran, untoasted muesli, porridge)**	
6 or more days a week	☐
3-5 days a week	☐
1-2 days a week	☐
Less than one day a week	☐
Never	☐

**65. How many different types of vegetables would you eat on a typical day?**	
5 or more types	☐
4 types	☐
3 types	☐
1 or 2 types	☐
None	☐

**66. How many days a week do you eat high fat cheeses? (e.g. cheddar or cream cheese)**	
6 or more days a week	☐
3-5 days a week	☐
1-2 days a week	☐
Less than one day a week	☐
Never	☐

**67. How often do you choose low-fat milk (semi-skimmed or skimmed) in preference to whole milk?**	
Always	☐
Usually	☐
Occasionally	☐
Rarely	☐
Never	☐
I don't drink milk	☐

**68. How many days a week do you eat processed meats? (e.g. bacon, salami, ham etc)**	
4 or more days a week	☐
2 or 3 days a week	☐
Once a week	☐
Less than one day a week	☐
Never	☐

**69. How often do you eat or drink any of the following?** *(Please tick (✓)* ***one*** *box for each row).*	**More than once a day**	**Once a day**	**3-6 days a week**	**1-2 days a week**	**less than weekly**	**Never**
Sweets, chocolate bars or biscuits (including wrapped chocolate biscuits like Twix or KitKat)	☐	☐	☐	☐	☐	☐
Buns, cakes or pastries or desserts	☐	☐	☐	☐	☐	☐
Fizzy drinks or squashes that contain sugar (e.g. coca cola, Ribena, Club Orange)	☐	☐	☐	☐	☐	☐
Diet drinks (e.g. Diet Coke, Sprite Zero, Diet Club)	☐	☐	☐	☐	☐	☐

**70. How often do you have a drink containing alcohol?**	
Never	☐
Monthly or less	☐
2 - 4 times a month	☐
2 - 3 times a week	☐
4 or more times a week	☐

**71. Do you keep a record of what you eat and drink? (e.g. writing it down, using an App etc).**	
Yes	☐
No	☐

**72. Do you set yourself food and drink related goals?** *(Please tick (✓)* ***one*** *box).*	
Yes	☐
No	☐

Section 8: Physical activity

We are interested in finding out about the kinds of physical activities that people do as part of their everyday lives. The questions will ask you about the time you spent being physically active **in the last 7 days**. Please answer each question even if you do not consider yourself to be an active person. Please think about the activities you do at work, as part of your house and yard work, to get from place to place, and in your spare time for recreation, exercise or sport.

VIGOROUS ACTIVITIES

Think about all the **vigorous** activities that you did **in the last 7 days**. **Vigorous** physical activities refer to activities that take hard physical effort and make you breathe much harder than normal. Think *only* about those physical activities that you did for at least 10 minutes at a time.

1. During the last 7 days, on how many days did you do vigorous physical activities like heavy lifting, digging, aerobics, or fast bicycling?

________	Days per week	
☐	No vigorous physical activities ⟶	***Skip to question 3***

2. How much time did you usually spend doing vigorous physical activities on one of those days?

*(Please answer in hours **AND** minutes. E.g. If you did 30 minutes of vigorous exercise, you can enter '0' in the Hours box and '30' in the Minutes box)*

________	Hours per day
________	Minutes per day
☐	Don’t know/not sure

MODERATE ACTIVITIES

Think about all the **moderate** activities that you did **in the last 7 days**. **Moderate** activities refer to activities that take moderate physical effort and make you breathe somewhat harder than normal. Think only about those physical activities that you did for at least 10 minutes at a time.

3. During the last 7 days, on how many days did you do moderate physical activities like carrying light loads, bicycling at a regular pace, or doubles tennis? Do not include walking.

_________ Days per week

☐ No moderate physical activities ⟶ ***Skip to question 5***

4. How much time did you usually spend doing moderate physical activities on one of those days?

*(Please answer in hours **AND** minutes. E.g. If you did 30 minutes of moderate exercise, you can enter '0' in the Hours box and '30' in the Minutes box)*

_________ Hours per day

_________ Minutes per day

☐ Don't know/not sure

WALKING

Think about the time you spent **walking in the last 7 days**. This includes at work and at home, walking to travel from place to place, and any other walking that you have done solely for recreation, sport, exercise, or leisure.

5. During the last 7 days, on how many days did you walk for at least 10 minutes at a time?

_________ Days per week

☐ No walking ⟶ ***Skip to question 7***

6. How much time did you usually spend walking on one of those days?

*(Please answer in hours **AND** minutes. E.g. If you walked for 2 hours, you can enter '2' in the Hours box and '0' in the Minutes box)*

_________ Hours per day

_________ Minutes per day

☐ Don't know/not sure

SITTING

The last question is about the time you spent **sitting** on weekdays during the **last 7 days**. Include time spent at work, at home, while doing course work and during leisure time. This may include time spent sitting at a desk, visiting friends, reading, or sitting or lying down to watch television.

7. During the last 7 days, how much time did you spend sitting on a week day?
*(Please answer in hours **AND** minutes. E.g. If you spent 8 hours sitting, you can enter '8' in the Hours box and '0' in the Minutes box)*

________	Hours per day
________	Minutes per day
☐	Don't know/not sure

STEPS

8. Do you use a step counter? (e.g. pedometer, App, Fitbit etc) *(Please tick (✓) **one** box).*

Yes	☐
No	☐

9. Do you set yourself physical activity related goals? *(Please tick (✓) **one** box).*

Yes	☐
No	☐

10. Did you have any problem/s that limited your physical activity during the past 3 months? *(Please tick (✓) **one** box).*

Yes	☐	If yes, please give details:
No	☐	

Section 9: Infant Feeding

The questions in this section are designed to explore how you feed your youngest child. If your most recent pregnancy was a twin/multiple pregnancy, please complete these questions for the youngest twin/multiple.

7. What is your current method of feeding for your youngest child? *(Please tick (✓)* ***one*** *box for each row).*

Breast feeding	☐ yes	☐ no
Infant formula feeding	☐ yes	☐ no
Combined feeding (Breast and infant formula)	☐ yes	☐ no
Follow-on milk	☐ yes	☐ no
Baby is on solids	☐ yes	☐ no

NOTE: If you ticked 'Baby is on solids', **please go to question 2 below.**

If you did not tick 'Baby is on solids', **please go to Section 10 on Page 33**.

8. How often do you usually give your baby these particular TYPES of solid food? *(Please tick (✓)* ***one*** *box for each food).*

	More than once a day	Once a day	3 or more times a week	Once or twice a week	Less than once a week	Never
Breakfast Cereals	☐	☐	☐	☐	☐	☐
Rice or Pasta	☐	☐	☐	☐	☐	☐
Bread	☐	☐	☐	☐	☐	☐
Potatoes	☐	☐	☐	☐	☐	☐
Potato products (incl. chips, waffles, shapes)	☐	☐	☐	☐	☐	☐
Butter/Margarine and other spreads	☐	☐	☐	☐	☐	☐
Red meat	☐	☐	☐	☐	☐	☐
Processed meat (e.g. ham)	☐	☐	☐	☐	☐	☐

Gallagher D, *et al. BMJ Open* 2024; 14:e084075. doi: 10.1136/bmjopen-2024-084075

	More than once a day	Once a day	3 or more times a week	Once or twice a week	Less than once a week	Never
Chicken/other poultry	☐	☐	☐	☐	☐	☐
Fish (incl. tuna)	☐	☐	☐	☐	☐	☐
Eggs	☐	☐	☐	☐	☐	☐
Beans, lentils, chickpeas	☐	☐	☐	☐	☐	☐
Tofu, Quorn, textured vegetable protein	☐	☐	☐	☐	☐	☐
Nuts	☐	☐	☐	☐	☐	☐
Fruit	☐	☐	☐	☐	☐	☐
Vegetables	☐	☐	☐	☐	☐	☐
Cheese, yoghurt, fromage frais	☐	☐	☐	☐	☐	☐
Puddings or desserts	☐	☐	☐	☐	☐	☐
Biscuits, sweets, chocolate or cakes	☐	☐	☐	☐	☐	☐
Crisps and corn snacks	☐	☐	☐	☐	☐	☐
Follow on formula	☐	☐	☐	☐	☐	☐
Or something else (please tick and then write below)	☐	☐	☐	☐	☐	☐

1542

9. Do you ever add salt to your baby's solid food, including adding salt when the food is being cooked? *(Please tick (✓)* ***one*** *box).*

Yes, often	☐
Yes, sometimes	☐
Never	☐

Section 10: Mood

*(Please tick (✓) **one** box for each statement below).*

In the past 7 days...

**17. I have been able to laugh and see the funny side of things.**	
As much as I always could	☐
Not quite so much now	☐
Definitely not so much now	☐
Not at all	☐

**18. I have looked forward with enjoyment to things.**	
As much as I ever did	☐
Rather less than I used to	☐
Definitely less than I used to	☐
Hardly at all	☐

**19. I have blamed myself unnecessarily when things went wrong.**	
Yes, most of the time	☐
Yes, some of the time	☐
Not very often	☐
No, never	☐

**20. I have been anxious or worried for no good reason.**	
No, not at all	☐
Hardly ever	☐
Yes, sometimes	☐
Yes, very often	☐

**21. I have felt scared or panicky for no very good reason.**	
Yes, quite a lot	☐
Yes, sometimes	☐
No, not much	☐
No, not at all	☐

**22.Things have been getting on top of me.**	
Yes, most of the time I haven't been able to cope at all	☐
Yes, sometimes I haven't been coping as well as usual	☐
No, most of the time I have coped quite well	☐
No, I have been coping as well as ever	☐

**23.I have been so unhappy that I have had difficulty sleeping.**	
Yes, most of the time	☐
Yes, sometimes	☐
Not very often	☐
No, not at all	☐

**24.I have felt sad or miserable.**	
Yes, most of the time	☐
Yes, quite often	☐
Not very often	☐
No, not at all	☐

**9. I have been so unhappy that I have been crying.**	
Yes, most of the time	☐
Yes, quite often	☐
Only occasionally	☐
No, never	☐

**10. The thought of harming myself has occurred to me.**	
Yes, quite often	☐
Sometimes	☐
Hardly ever	☐
Never	☐

*(Please tick (✓) **one** box for each statement below).*

<u>Over the last two weeks</u>, how often have you been bothered by the following problems?

**26. Feeling nervous, anxious, or on edge?**	
Not at all	☐
Several days	☐
More than half of the days	☐
Nearly every day	☐

**27. Not being able to stop or control worrying?**	
Not at all	☐
Several days	☐
More than half of the days	☐
Nearly every day	☐

**28. Worrying too much about different things?**	
Not at all	☐
Several days	☐
More than half of the days	☐
Nearly every day	☐

**29. Trouble relaxing?**	
Not at all	☐
Several days	☐
More than half of the days	☐
Nearly every day	☐

**30. Being so restless that it is hard to sit still?**	
Not at all	☐
Several days	☐
More than half of the days	☐
Nearly every day	☐

**31. Becoming easily annoyed or irritable?**	
Not at all	☐
Several days	☐
More than half of the days	☐
Nearly every day	☐

**32. Feeling afraid as if something awful might happen?**	
Not at all	☐
Several days	☐
More than half of the days	☐
Nearly every day	☐

Having a young baby or child can bring up lots of different feelings and emotions. The next questions are designed to explore how you are feeling about being a parent to your baby/child. Answering these questions will help us to understand how things are going for you. If your most recent pregnancy was a twin/multiple pregnancy, please complete these questions for the youngest twin/multiple.

**33. Has your child reached their second birthday?** *(Please tick (✓) **one** box).*		
No, my child has not yet had their second birthday	☐	→ Please go to question 19 on page 37.
Yes, my child has celebrated their second birthday	☐	→ Please go to question 21 on Page 39

19. Thinking about your feelings about your baby, choose the response for each statement that feels right to you. *(Please tick (✓)* ***one*** *box for each of the following statements).*

	Never	Sometimes	Always
I enjoy looking after my baby	☐	☐	☐
I feel irritated with my baby when we are together	☐	☐	☐
I feel affectionate towards my baby	☐	☐	☐
I feel that my baby is being difficult or trying to upset me on purpose	☐	☐	☐
I can work out what my baby needs from me	☐	☐	☐
I feel like I can't do things I enjoy because of my baby.	☐	☐	☐
I feel the changes in my life are worth it to look after my baby.	☐	☐	☐
I miss my baby when we are not together	☐	☐	☐
I feel like I'm looking after my baby for someone else.	☐	☐	☐
When we've been apart I look forward to seeing my baby again.	☐	☐	☐
I enjoy playing with my baby.	☐	☐	☐

20. Thinking about your baby, please tick one of the choices for each of the questions below. There are no 'right' or 'wrong' answers; many of these are

true of all babies at times. *(Please tick (✓)* ***one*** *box for each of the following statements).*

	Always	Very often	Quite often	Sometimes	Rarely	Never
My baby smiles at me.	☐	☐	☐	☐	☐	☐
My baby annoys me.	☐	☐	☐	☐	☐	☐
My baby likes doing things with me.	☐	☐	☐	☐	☐	☐
My baby ‘talks’ to me.	☐	☐	☐	☐	☐	☐
My baby irritates me.	☐	☐	☐	☐	☐	☐
My baby likes me.	☐	☐	☐	☐	☐	☐
My baby wants too much attention.	☐	☐	☐	☐	☐	☐
My baby laughs.	☐	☐	☐	☐	☐	☐
My baby gets moody.	☐	☐	☐	☐	☐	☐
My baby dominates me.	☐	☐	☐	☐	☐	☐
My baby likes to please me.	☐	☐	☐	☐	☐	☐
My baby cries for no obvious reason.	☐	☐	☐	☐	☐	☐
My baby is affectionate towards me.	☐	☐	☐	☐	☐	☐
My baby winds me up.	☐	☐	☐	☐	☐	☐

Please go to section 11 on Page 41

21. Thinking about your feelings about your child, choose the response for each statement that feels right to you. *(Please tick (✓)* ***one*** *box for each of the following statements).*

	Never	Sometimes	Always
I enjoy looking after my child	☐	☐	☐
I feel irritated with my child when we are together	☐	☐	☐
I feel affectionate towards my child	☐	☐	☐
I feel that my child is being difficult or trying to upset me on purpose	☐	☐	☐
I can work out what my child needs from me	☐	☐	☐
I feel like I can't do things I enjoy because of my child.	☐	☐	☐
I feel the changes in my life are worth it to look after my child.	☐	☐	☐
I miss my child when we are not together	☐	☐	☐
I feel like I'm looking after my child for someone else.	☐	☐	☐
When we've been apart I look forward to seeing my child again.	☐	☐	☐
I enjoy playing with my child.	☐	☐	☐

22. Thinking about your child, please tick one of the choices for each of the questions below. There are no 'right' or 'wrong' answers; many of these are

true of all children at times. *(Please tick (✓)* ***one*** *box for each of the following statements).*

	Always	Very often	Quite often	Sometimes	Rarely	Never
My child smiles at me.	☐	☐	☐	☐	☐	☐
My child annoys me.	☐	☐	☐	☐	☐	☐
My child likes doing things with me.	☐	☐	☐	☐	☐	☐
My child talks to me.	☐	☐	☐	☐	☐	☐
My child irritates me.	☐	☐	☐	☐	☐	☐
My child likes me.	☐	☐	☐	☐	☐	☐
My child wants too much attention.	☐	☐	☐	☐	☐	☐
My child laughs.	☐	☐	☐	☐	☐	☐
My child gets moody.	☐	☐	☐	☐	☐	☐
My child dominates me.	☐	☐	☐	☐	☐	☐
My child likes to please me.	☐	☐	☐	☐	☐	☐
My child cries for no obvious reason.	☐	☐	☐	☐	☐	☐
My child is affectionate towards me.	☐	☐	☐	☐	☐	☐
My child winds me up.	☐	☐	☐	☐	☐	☐

Section 11: Self-esteem

Below is a list of statements dealing with your general feelings about yourself.

(Please tick (✓) ***one*** *box to indicate how much you agree or disagree with each of the following statements).*

	Strongly Agree	Agree	Disagree	Strongly disagree
I feel that I'm a person of worth, at least on an equal plane with others.	☐	☐	☐	☐
I feel that I have a number of good qualities.	☐	☐	☐	☐
All in all, I am inclined to feel that I am a failure.	☐	☐	☐	☐
I am able to do things as well as most other people.	☐	☐	☐	☐
I feel I do not have much to be proud of.	☐	☐	☐	☐
I take a positive attitude toward myself.	☐	☐	☐	☐
On the whole I am satisfied with myself.	☐	☐	☐	☐
I wish I could have more respect for myself.	☐	☐	☐	☐
I certainly feel useless at times.	☐	☐	☐	☐
At times I think I am no good at all.	☐	☐	☐	☐

Section 12: Sleep

1. At the moment, how often does your baby sleep all night? *(Please tick (✓) **one** box).*

Always	☐
Mostly	☐
Sometimes	☐
Rarely	☐
Never	☐

The following questions relate to **your** usual sleep habits during the **past month only**. Your answers should indicate the most accurate reply for the majority of days and nights in the past month. Please answer all questions.

2. During the past month, what time have you usually gone to bed at night?

Usual bed time ☐

3. During the past month, how long (in minutes) has it usually taken you to fall asleep each night?

Number of minutes ☐

4. During the past month, what has been your usual getting up time in the morning?

Usual getting up time ☐

5. During the past month, on average how many hours of actual sleep did you get at night? (This may be different than the number of hours you spent in bed.)

Hours of sleep per night ☐

Gallagher D, *et al. BMJ Open* 2024; 14:e084075. doi: 10.1136/bmjopen-2024-084075

6. During the past month, how often have you had trouble sleeping because you…

*(Please tick (✓) **one** box for each statement).*

	Not during the past month	Less than once a week	Once or twice a week	Three or more times a week
Cannot get to sleep within 30 minutes	☐	☐	☐	☐
Wake up in the middle of the night or early morning	☐	☐	☐	☐
Have to get up to use the bathroom	☐	☐	☐	☐
Cannot breathe comfortably	☐	☐	☐	☐
Cough or snore loudly	☐	☐	☐	☐
Feel too cold	☐	☐	☐	☐
Feel too hot	☐	☐	☐	☐
Had bad dreams	☐	☐	☐	☐
Have pain	☐	☐	☐	☐
Have to take care of your baby	☐	☐	☐	☐
Other reason(s), please describe and indicate how often during the past month you had trouble sleeping because of this:				
[ ]	☐	☐	☐	☐
[ ]	☐	☐	☐	☐
[ ]	☐	☐	☐	☐

7. During the past month, how would you rate your sleep quality overall? *(Please tick (✓)* ***one*** *box).*

Very Good	Fairly Good	Fairly Bad	Very Bad
☐	☐	☐	☐

**8**. (Please tick *(✓)* ***one*** *box for each question below.)*	Not during the past month	Less than once a week	Once or twice a week	Three or more times a week
**During the past month, how often have you taken medicine (prescribed or "over the counter") to help you sleep?**	☐	☐	☐	☐
**During the past month, how often have you had trouble staying awake while driving, eating meals, or engaging in social activity?**	☐	☐	☐	☐

9. During the past month, how much of a problem has it been for you to keep up enough enthusiasm to get things done? (Please tick *(✓)* ***one*** *box).*

No problem at all	Only a very slight problem	Somewhat of a problem	A very big problem
☐	☐	☐	☐

10. Do you usually have a bed partner or room-mate? *(Please tick (✓)* ***one*** *box).*

No bed partner or room-mate	Partner/room-mate in other room	Partner in same room but not in same bed	Partner in same bed
☐	☐	☐	☐

Gallagher D, *et al. BMJ Open* 2024; 14:e084075. doi: 10.1136/bmjopen-2024-084075

11. If you usually have a room-mate or bed partner, ask him/her how often in the past month you have had:

Please tick (✓) ***one*** *box for each statement).*	**Not during the past month**	**Less than once a week**	**Once or twice a week**	**Three or more times a week**
Loud snoring	☐	☐	☐	☐
Long pauses between breaths while asleep	☐	☐	☐	☐
Legs twitching or jerking while you sleep	☐	☐	☐	☐
Episodes of confusion during sleep	☐	☐	☐	☐
Other restlessness while you sleep, please describe and indicate how often in the past month you have had this:				
☐ (text box)	☐	☐	☐	☐
☐ (text box)	☐	☐	☐	☐
☐ (text box)	☐	☐	☐	☐

152

You have now completed the questionnaire. Thank you very much for your time and effort! We really appreciate your help with our research.

Supplemental appendix 4- Interview guides

SEMI-STRUCTURED INTERVIEW TOPIC GUIDE

6 months – Intervention Group

The facilitator will introduce him/herself and reiterate the purpose of the interview i.e. to gather thoughts and opinions on the Supporting MumS study.

The facilitator will emphasise that although the interview will be audio-recorded for research purposes, all information provided by the respondent will be kept anonymous (i.e. their name will never be disclosed). The approximate time of the interview (20-30 minutes) will be stated. Individuals will also be informed that if at any stage they wish to discontinue with the interview they are free to do so. In addition, it will be made clear to respondents that they can inform the facilitator if there are any particular statements they do not wish to be transcribed at the end of the session.

Before commencing, participants will be asked to verbally confirm consent as below:

- Do you agree to the interview being audio-recorded?
- Do you agree that anonymised quotations from these discussions can be used in research publications?

The interview will only proceed if the participant answers yes to the statements above.

Topic Guide:

Opinions on the SMS study

- Why did you sign up to take part in the SMS study? *(Prompt: What were your motivations for signing up to take part?)*
- What do you think of the information you received at the beginning of the study? (i.e. the poster, information sheet, flyer, info from researchers via telephone etc.)
- What do you think of the SMS study so far?
- Did you have a preference for which group you would be assigned to at the start (intervention/control – remind participants of the groups)?

Opinions on the text messages

- Overall, how have you found the text messages you have received so far? (*Prompt: content, tone, length, clarity, relevance)*
- Have you been receiving any of the messages related specifically to breastfeeding or smoking? *(Prompt: If yes, were they useful?)*
- What type of text messages do you like the most/least? *(Prompt: Why?)*
- What do you think of the messages that prompt you to check your weight each week? *(Probe: to what extent do you engage with these messages? Prompt: do you reply to these messages? Why?)*
- What do you think of the messages that prompt you to set goals and review your goals each week? *(Probe: to what extent do you engage with these messages? Prompt: do you reply to these messages? Why?)*
- When you get a text message, how likely are you to read it?
- Do you ever go back and re-read messages? *(Prompt: Do you keep the messages or delete them?)*
- Have you used any of the trigger words?
- Do you click on the links in the messages to websites? *If not, why not?*
- What did you think about the frequency of text messages (i.e. how often you are receiving them)?
- Could the text messages you have received so far have been improved in any way? If so, how?
- At any stage, did you notice or were you informed of an issue with the text messages? E.g. text about a glitch in the system, repeated messages? *Probe: what did this make you think about the messages? Did it impact how you engage with the messages?*
- *Mothers of twins/multiples only:* What do you think of the suitability of the messages for women with twins or multiples?
- *Mothers of twins/multiples only:* How do feel having twins/multiples has impacted your receipt of the intervention? *(Prompts: in what ways has it impacted your ability to make changes to your eating habits or physical activity?)*

<u>Behaviour change</u>

- How do you feel your eating habits or physical activity have changed as a result of the text messages? (*Prompts: in what way? What are the main changes you have made)*
- Have there been any negatives about signing up for the messages?

BMJ Publishing Group Limited (BMJ) disclaims all liability and responsibility arising from any reliance placed on this supplemental material which has been supplied by the author(s)

- What do you think are the main benefits, if any, you have gained so far from signing up for the messages?
- Do you think receiving the messages has had any wider impacts or benefits for your family?

Social support

- You were given the opportunity to have a friend or family member also receive the messages. Have you done this so far? *(Prompts: If so how are you finding it? If not, why not?)*
- To what extent do you feel your friends and family supported you to make changes to your eating habits or physical activity?

Postnatal mental health

- How do you feel your mental health has been since having your baby?
- How do you feel your mental health has impacted your ability to follow the weight management advice that you received?
- In what ways has receiving the intervention impacted your mental health, if at all?
- *For women who discuss experiencing postnatal mental health issues:* what support services have you accessed to help with your mental health since having a baby? *(Prompts: to what extent did this help you? What further support do you feel you would have benefitted from?)*

Data collection

- How did you find the study visits (i.e. meeting with a researcher) so far?
- What did you think of having the option of being able to meet a researcher at your home instead of travelling somewhere for the visit?
- What do you think of the questionnaires you have had to complete? (*Prompts: what did you think of the length? How easy were they to understand?)*
- Did you receive a voucher following your study visit? What did you think of this part of the study i.e. receiving vouchers? *Prompts: Did it influence your decision to sign-up to the study? Why do you think we gave vouchers to participants taking part?)*

INTERVIEW ENDS

SEMI-STRUCTURED INTERVIEW TOPIC GUIDE

6 months – Control Group

The facilitator will introduce him/herself and reiterate the purpose of the interview i.e. to gather thoughts and opinions on the Supporting MumS study.

The facilitator will emphasise that although the interview will be audio-recorded for research purposes, all information provided by the respondent will be kept anonymous (i.e. their name will never be disclosed). The approximate time of the interview (20-30 minutes) will be stated. Individuals will also be informed that if at any stage they wish to discontinue with the interview they are free to do so. In addition, it will be made clear to respondents that they can inform the facilitator if there are any particular statements they do not wish to be transcribed at the end of the session.

Before commencing, participants will be asked to verbally confirm consent as below:

- Do you agree to the interview being audio-recorded?
- Do you agree that anonymised quotations from these discussions can be used in research publications?

The interview will only proceed if the participant answers yes to the statements above.

Topic Guide:

<u>Opinions on the SMS study</u>

- Why did you sign up to take part in the SMS study? *(Prompt: what were your motivations for signing up to take part?)*
- What do you think of the information you received at the beginning of the study? (i.e. the poster, information sheet, flyer, info from researchers via telephone etc.)
- What do you think of the SMS study so far?
- Did you have a preference for which group you were assigned to at the start (intervention/control – remind participants of the groups)?

<u>Opinions on the text messages</u>

- Overall, how have you found the text messages you have received so far? (*Prompt: content, tone, length, clarity, relevance)*

- What type of text messages do you like the most/least? *(Prompts: e.g. developmental messages; play ideas; Why do you like these the most/least?)*
- When you receive a text message, how likely are you to read it?
- Do you click on the links in the messages to websites? *(Prompt: If not, why not?)*
- What do you think about the frequency of text messages (i.e. how often you are receiving them)?
- Could the text messages you have received so far have been improved in any way? *(Prompt: If so, how?)*
- *Mothers of twins/multiples only:* What do you think of the suitability of the messages for women with twins or multiples?

<u>Impact of study</u>

- Have there been any negatives about signing up for the messages?
- What do you think are the main benefits, if any, you have gained so far from signing up for the messages?
- Do you think receiving the messages has had any wider impacts or benefits for your family?

<u>Postnatal mental health</u>

- How do you feel your mental health has been since having your baby?
- *For women who discuss experiencing postnatal mental health issues:* what support services have you accessed to help with your mental health since having a baby? *(Prompts: to what extent did this help you? What further support do you feel you would have benefitted from?)*

<u>Data collection</u>

- How have you found the study visits (i.e. meeting with a researcher) so far?
- What did you think of having the option of being able to meet a researcher at your home instead of travelling somewhere for the visit?
- What do you think of the questionnaires you have had to complete? (*Prompts: what did you think of the length? How easy were they to understand?)*
- Did you receive a voucher following your study visit? *(Prompts: What did you think of this part of the study i.e. receiving vouchers? Did it influence your decision to sign-up to the study? Why do you think we gave vouchers to participants taking part?)*

INTERVIEW ENDS

<u>SEMI-STRUCTURED INTERVIEW TOPIC GUIDE</u>

12 months – Intervention Group

The facilitator will introduce him/herself and reiterate the purpose of the interview i.e. to gather thoughts and opinions on the Supporting MumS study.

The facilitator will emphasise that although the interview will be audio-recorded for research purposes, all information provided by the respondent will be kept anonymous (i.e. their name will never be disclosed). The approximate time of the interview (20-30 minutes) will be stated. Individuals will also be informed that if at any stage they wish to discontinue with the interview they are free to do so. In addition, it will be made clear to respondents that they can inform the facilitator if there are any particular statements they do not wish to be transcribed at the end of the session.

Before commencing, participants will be asked to verbally confirm consent as below:

- Do you agree to the interview being audio-recorded?
- Do you agree that anonymised quotations from these discussions can be used in research publications?

The interview will only proceed if the participant answers yes to the statements above.

Topic Guide:

<u>Opinions on the SMS study</u>

- What did you think of the SMS study?
- What did you think of the information you received at the beginning of the study?
- Did you have a preference for which group you were assigned to at the start (intervention/control – remind participants of the groups)?

<u>Opinions on the text messages</u>

- Overall, did you like the messages you received during the SMS study? (*Prompt: tone, length, clarity, relevance)*
- Did you receive any of the messages related specifically to breastfeeding or smoking? *(Prompt: If yes, were they useful?)*
- Overall, did you find the text messages useful or not useful?
- What type of text message did you like the most/least? *(Prompt: Why did you like these the most/least?)*

- What do you think of the messages that prompt you to check your weight each week? *(Probe: to what extent do you engage with these messages? Prompt: do you reply to these messages? Why?)*
- What do you think of the messages that prompt you to set goals and review your goals each week? *(Probe: to what extent do you engage with these messages? Prompt: do you reply to these messages? Why?)*
- When you got a text message, how likely were you to read it?
- Do you ever go back and re-read messages? *(Prompt: Do you keep the messages or delete them?)*
- Have you used any trigger words (i.e. tired, bad day, crave, slip-up)?
- What do you think about the frequency of text messages (i.e. how often you received them)?
- What do you think of the texts that had links to the SMS website? (*Prompt: did you click on the links?)*
- Could the text messages you received have been improved in any way? *(Prompt: If so, how?)*
- Women were able to join the Supporting MumS study up to two years after having a baby. How useful do you think that is? *(Prompt: do you think there is a best time for receiving the text messages? How ready were you to engage with the advice?)*
- *Mothers of twins/multiples only:* What do you think of the suitability of the messages for women with twins or multiples?
- *Mothers of twins/multiples only:* How do feel having twins/multiples has impacted your receipt of the intervention? *(Prompt: in what ways has it impacted your ability to make changes to your eating habits or physical activity?).*

<u>Behaviour change</u>

- Have you changed your eating habits or physical activity as a result of the text messages? *(Prompts: In what way? What are the main changes you have made?)*
- Have there been any negatives about signing up for the messages?
- What do you think are the main benefits, if any, you gained from signing up for the messages?
- Do you think receiving the messages has had any wider impacts or benefits for your family?
- Do you think you will continue with the changes you have made now that the study has finished?

<u>Social support</u>

- You were given the opportunity to have a friend or family member also receive the messages, did you do this? *(Prompt: If not, why not?)*
- To what extent do you feel your friends and family supported you to make changes to your eating habits or physical activity?

<u>Postnatal mental health</u>

- How do you feel your mental health has been since having your baby?
- How do you feel your mental health has impacted your ability to follow the weight management advice that you received?
- In what ways has receiving the intervention impacted your mental health, if at all?
- *For women who discuss experiencing postnatal mental health issues:* what support services have you accessed to help with your mental health since having a baby? *(Prompt: to what extent did this help you? What further support do you feel you would have benefitted from?)*

<u>Data collection</u>

- What did you think about having your measurements taken? (Prompts: How did you feel about being weighed? How did you feel about having your waist circumference measured? Is there anything that could have been done better in taking these measurements?)
- What do you think of the questionnaires you have had to complete during the study visits? (*Prompts: what did you think of the length? How easy were they to understand?)*
- What did you think of having the option of being able to meet a researcher at your home instead of travelling somewhere for the visit?

<u>Overall evaluation</u>

- What was the best thing about the SMS study?
- What was the worst thing about the SMS study?

Gallagher D, *et al. BMJ Open* 2024; 14:e084075. doi: 10.1136/bmjopen-2024-084075

- Is there anything we could have done that would have made the experience better? (*Prompts: is there anything you feel should be added to the intervention? Is there any part of the study you would recommend changing?)*
- Would you take part in the SMS study again if you had the chance?
- Would you recommend the SMS study to new mums?

INTERVIEW ENDS

SEMI-STRUCTURED INTERVIEW TOPIC GUIDE

12 months – Control Group

The facilitator will introduce him/herself and reiterate the purpose of the interview i.e. to gather thoughts and opinions on the Supporting MumS study.

The facilitator will emphasise that although the interview will be audio-recorded for research purposes, all information provided by the respondent will be kept anonymous (i.e. their name will never be disclosed). The approximate time of the interview (20 minutes) will be stated. Individuals will also be informed that if at any stage they wish to discontinue with the interview they are free to do so. In addition, it will be made clear to respondents that they can inform the facilitator if there are any particular statements they do not wish to be transcribed at the end of the session.

Before commencing, participants will be asked to verbally confirm consent as below:

- Do you agree to the interview being audio-recorded?
- Do you agree that anonymised quotations from these discussions can be used in research publications?

The interview will only proceed if the participant answers yes to the statements above.

Topic Guide:

<u>Opinions on the SMS study</u>

- What did you think of the SMS study?
- What did you think of the information you received at the beginning of the study?
- Did you have a preference for which group you would be assigned to at the start (intervention/control – remind participants of the groups)?

<u>Opinions on the text messages</u>

- Overall, did you like the messages you received during the SMS study? *(Prompt: tone, length, clarity, relevance)*
- Did you find the text messages useful?
- Which type of text messages did you like the most/least? *(Prompt: Why did you like these the most/least?)*
- When you received a text message, how likely were you to read the message?
- What did you think of the texts that had links to the SMS website? (*Prompt: did you click on the links?)*
- What did you think about the frequency of text messages (i.e. how often you received them)?
- Could the text messages have been improved? *(Prompt: If so, how?)*

- *Mothers of twins/multiples only:* What do you think of the suitability of the messages for women with twins or multiples?

Impact of the study

- Have there been any negatives about signing up for the messages?
- What do you think are the main benefits, if any, you have gained from signing up for the messages?
- Do you think receiving the messages has had any wider impacts or benefits for your family?

Postnatal mental health

- How do you feel your mental health has been since having your baby?
- *For women who discuss experiencing postnatal mental health issues:* what support services have you accessed to help with your mental health since having a baby? *(Prompts: to what extent did this help you? What further support do you feel you would have benefitted from?)*

Data collection

- What did you think about having your measurements taken? (Prompts: How did you feel about being weighed? How did you feel about having your waist circumference measured? Is there anything that could have been done better in taking these measurements?)
- What did you think of the questionnaires you had to complete during the study visits? (*Prompts: what did you think of the length? How easy were they to understand?*
- What did you think of having the option of being able to meet a researcher at your home instead of travelling somewhere for the visit?

Overall evaluation

- What was the best thing about the SMS study?
- What was the worst thing about the SMS study?
- Is there anything we could have done that would have made the experience better? *(Prompts: is there anything you feel could be added to the text messages? Is there any part of the study you would recommend changing?)*
- Would you take part in the SMS study again if you had the chance?
- Would you recommend the SMS study to new mums?

INTERVIEW ENDS

2024 **Supplemental appendix 5- World Health Organization Trial Registration Data Set**

**Data Category**	**Information**
Primary Registry and Trial Identifying Number	ISRCTN Registry; ISRCTN16299220
Date of Registration in Primary Registry	10/02/2022
Secondary Identifying Numbers	FUNDERS Number: NIHR131509<br>Sponsor Number: B21/24<br>IRAS Number: 305557<br>REC Reference: 22/WS/0003
Source(s) of Monetary or Material Support	National Institute for Health and Care Research (NIHR) Public Health Research (PHR). Intervention costs provided by the Public Health Agency (Northern Ireland).
Primary Sponsor	Queen's University Belfast
Secondary Sponsor(s)	None
Contact for Public Queries	Dr Dunla Gallagher<br>SMS Trial Manager<br>d.gallagher@qub.ac.uk<br>07341 888415<br>Centre for Public Health<br>Queen's University Belfast<br>Institute of Clinical Sciences B<br>Grosvenor Road<br>Belfast<br>BT12 6BJ
Contact for Scientific Queries	Professor Michelle McKinley<br>Chief Investigator<br>m.mckinley@qub.ac.uk<br>028 9097 8936<br>Centre for Public Health<br>Queen's University Belfast<br>Institute of Clinical Sciences A<br>Grosvenor Road<br>Belfast<br>BT12 6BA

Gallagher D, *et al. BMJ Open* 2024; 14:e084075. doi: 10.1136/bmjopen-2024-084075

Public Title	The Supporting MumS (SMS) study: can we use an automated text message intervention for weight management in women after childbirth?
Scientific Title	Effectiveness and cost effectiveness of an automated text message intervention for weight management in postpartum women with overweight or obesity: the Supporting MumS (SMS) Randomised Controlled Trial.
Countries of Recruitment	United Kingdom (Northern Ireland, Scotland, England and Wales).
Health Condition(s) or Problem(s) Studied	Overweight and obesity in postpartum women.
Intervention(s)	Supporting MumS intervention group- will receive automated text messages about weight loss and maintenance of weight loss for 12 months. The text messages will focus on diet and physical activity with embedded behaviour change techniques known to be positively associated with weight management.<br><br>Active control group- will receive automated text messages about child health and development for 12 months.
Key Inclusion and Exclusion Criteria	Inclusion criteria<br>• Women (as per NICE Postnatal care guideline NG194[1], the term 'woman' is taken to include people who do not identify as women but who are pregnant or have given birth)<br>• Aged > 18 years old<br>• BMI ≥25 kg/m^2<br>• Have had a baby within the last two years<br><br>Exclusion criteria<br>• Baby less than 6 weeks old<br>• No access to a mobile phone to receive personal messages<br>• Insufficient English to understand short written messages<br>• Currently pregnant<br>• Recent or planned bariatric surgery<br>• Previous diagnosis of anorexia or bulimia by a doctor

	• On a specialist diet and receiving dietetic care<br>• Taking part in another weight management research study currently, or in the last 3 months
Study Type	Two-arm parallel groups randomised controlled trial to test the effectiveness and cost-effectiveness of a 12-month text-messaging behavioural weight management intervention in supporting weight loss for women with overweight and obesity in the postpartum period, compared with an active control group receiving text messages related to child health and development for 12 months.<br><br>Women will be recruited from all four countries in the UK using community-based recruitment as well as signposting via routine contact with health professionals.<br><br>Written informed consent will be obtained, baseline data collected and then participants will be randomised. Participants will be block randomised and randomisation will be stratified by site. The randomisation sequence will be developed in STATA by a statistician who is independent of the study team. The randomisation will be implemented via the London School of Hygiene and Tropical Medicine secure remote web-based system which will link directly with the text message database and will deliver the intervention or active control content according to the random allocation sequence.<br><br>Participants will become aware of their group allocation when they start to receive the messages. Researchers who are recruiting women to the study and collecting outcome data are blinded to group allocation.
Date of First Enrolment	Actual date of enrolment of the first participant: 20/05/2022
Sample Size	Target sample size: 888<br>Final number of participants that the trial enrolled: 892
Recruitment Status	Complete: participants are no longer being recruited or enrolled.

Primary Outcome	Weight change from baseline to 12 months (kg), measured using calibrated scales.
Secondary Outcome	1. Waist circumference measured by a flexible measuring tape at baseline, 6, 12 and 24 months, and mean BMI (kg/m^2) and the proportions of women gaining a substantial amount of weight (>5kg) at 12 and 24 months.<br><br>2. Health behaviours are measured by: Fat and Fibre Barometer; self-report questionnaire on sugar and alcohol intake; International Physical Activity Questionnaire (IPAQ) - Short form; and self-report questionnaire on infant feeding practices at baseline, 6, 12 and 24 months.<br><br>3. Study acceptability is measured by: recruitment; retention; engagement with the two-way text messages over 12 months; self-reported questionnaire on participant satisfaction with SMS messages (at 6 and 12 months) and rating of intervention (at 12 months); and qualitative interviews (at 6 and 12 months).<br><br>4. Economic evaluation outcomes are measured by: self-report questionnaire on health service resources use, medication usage and lifestyle-related costs; the EuroQol 5-dimension (EQ-5D) quality of life questionnaire; and the ICEpop Capability Measure for Adults (ICECAP-A) at baseline, 6, 12 and 24 months.<br><br>5. Moderators of intervention effect are measured by: Edinburgh Postnatal Depression Scale (EPDS); Generalised Anxiety Disorder (GAD-7); Pittsburgh Sleep Quality Index; and self-report questionnaire on confidence and desire for weight loss and maintenance at baseline, 6, 12 and 24 months.<br><br>6. Mediators of intervention effect are measured by: Health Action Process Approach (HAPA) for diet and exercise; Self-Report Behavioural Automaticity Index for diet and exercise; Self-regulation of eating behaviour questionnaire; self-report questionnaire on monitoring and goal setting for diet and

	exercise; Motivation for weight loss scale; Social support for eating & exercise questionnaire; and Rosenberg Self-Esteem Scale at baseline, 6, 12 and 24 months.
Ethics Review	Approved 24/02/2022 (Ref: 22/WS/0003) by West of Scotland REC 4 (West of Scotland Research Ethics Service, Ward 11, Dykebar Hospital, Grahamston Road, Paisley, PA2 7DE, United Kingdom; +44 (0)141 3140213; WoSREC4@ggc.scot.nhs.uk).
Completion date	30/04/2025
Summary results	Intention to publish date: 30/04/2026.
IPD Sharing Statement	Following publication of the results, the anonymised participant-level datasets generated during and/or analysed during the current study, along with the statistical code used for generating the results, will be available upon request from the Chief Investigator on reasonable request, as assessed by the Chief Investigator and Project Management Team and subject to any necessary data sharing agreements. Formal requests to be made in writing to the Chief Investigator (Prof M McKinley; m.mckinley@qub.ac.uk). Data availability will be consistent with timelines for storage of research data.

Gallagher D, *et al. BMJ Open* 2024; 14:e084075. doi: 10.1136/bmjopen-2024-084075
