# Supplementary material for: Effectiveness and cost-effectiveness of a 12-month automated text message intervention for weight management in postpartum women with overweight or obesity: protocol for the Supporting MumS (SMS) multisite, parallel-group, randomised controlled trial
Source: BMJ Open. 2024 May 6;14(5):e084075. doi: 10.1136/bmjopen-2024-084075 (PMC11086389; doi:10.1136/bmjopen-2024-084075)
Supplement: Author's manuscript [file bmjopen-2024-084075.draft_revisions.pdf]

BMJ Open is committed to open peer review. As part of this commitment we make the peer review history of every article we publish publicly available.

When an article is published we post the peer reviewers' comments and the authors' responses online. We also post the versions of the paper that were used during peer review. These are the versions that the peer review comments apply to.

The versions of the paper that follow are the versions that were submitted during the peer review process. They are not the versions of record or the final published versions. They should not be cited or distributed as the published version of this manuscript.

BMJ Open is an open access journal and the full, final, typeset and author-corrected version of record of the manuscript is available on our site with no access controls, subscription charges or pay-per-view fees (http://bmjopen.bmj.com).

If you have any questions on BMJ Open's open peer review process please email info.bmjopen@bmj.com

BMJ Open

Effectiveness and cost effectiveness of a 12 month automated text message intervention for weight management in postpartum women with overweight or obesity: protocol for the Supporting MumS (SMS) multi-site, parallel-group, randomised controlled trial.

Journal:	*BMJ Open*
Manuscript ID	bmjopen-2024-084075
Article Type:	Protocol
Date Submitted by the Author:	08-Jan-2024
Complete List of Authors:	Gallagher, Dunla; Queen's University Belfast, Centre for Public Health<br>Spyreli, Eleni; Queen's University Belfast, Centre for Public Health<br>Anderson, Annie; University of Dundee, CPHNR<br>Bridges, Sally; Bradford Institute for Health Research<br>Cardwell, Christopher; Queen's University Belfast, Centre for Public Health<br>Coulman, Elinor ; Cardiff University School of Medicine, Centre for Trials Research (CTR)<br>Dombrowski, Stephan; University of New Brunswick Fredericton, Kinesiology<br>Free, Caroline; London School of Hygiene & Tropical Medicine, Public Health interventions Unit<br>Heaney, Suzie; Queen's University Belfast, Centre for Public Health<br>Hoddinott, Pat; University of Stirling, Nursing, Midwifery and Allied Health Professional Research Unit<br>Kee, Frank; Queen's University Belfast, Centre for Public Health<br>McDowell, Clíona; Northern Ireland Clinical Trials Unit<br>McIntosh, Emma; University of Glasgow Institute of Health and Wellbeing, Health Economics and Health Technology Assessment (HEHTA)<br>Woodside, Jayne; Queen's University Belfast, Centre for Public Health<br>McKinley, Michelle C.; Queen's University Belfast, Centre for Public Health
Keywords:	Obesity, Randomized Controlled Trial, Postpartum Women < Postpartum Period, Behavior, HEALTH ECONOMICS, QUALITATIVE RESEARCH

SCHOLARONE™
Manuscripts

Effectiveness and cost effectiveness of a 12 month automated text message intervention for weight management in postpartum women with overweight or obesity: protocol for the Supporting MumS (SMS) multi-site, parallel-group, randomised controlled trial.

Dunla Gallagher, Eleni Spyreli, Annie S Anderson, Sally Bridges, Chris Cardwell, Elinor Coulman, Stephan U Dombrowski, Caroline Free, Suzie Heaney, Pat Hoddinott, Frank Kee, Cliona McDowell, Emma McIntosh, Jayne V Woodside and Michelle C McKinley.

Correspondence to Prof Michelle McKinley; m.mckinley@qub.ac.uk

Abstract

Introduction

The reproductive years can increase women's weight-related risk. Evidence for effective postpartum weight management interventions is lacking and engaging women at this time is challenging. Following a promising pilot evaluation of the Supporting MumS intervention, we assess if theory-based and bidirectional text messages to support diet and physical activity behaviour change for weight loss and weight loss maintenance, are effective and cost-effective for weight change in postpartum women with overweight or obesity, compared with an active control arm receiving text messages on child health and development.

Methods and analysis

Two-arm, parallel group, assessor-blind randomised controlled trial with cost-effectiveness and process evaluations. Women (n=888) with body mass index ≥25 kg/m2 and within 24 months of giving birth, recruited via community and NHS pathways through five United Kingdom sites targeting areas of ethnic and socioeconomic diversity. Women are 1:1 randomised to the intervention or active control groups, each receiving automated text

For peer review only - http://bmjopen.bmj.com/site/about/guidelines.xhtml

messages for 12 months. Data is collected at 0, 6, 12 and 24 months. The primary outcome is weight change at 12 months from baseline, compared between-groups. Secondary outcomes include weight change (24 months) and waist circumference (cm), proportional weight gain (>5kg), BMI (kg/m2), dietary intake, physical activity, infant feeding and mental health (12 and 24 months respectively). Economic evaluation examines health service usage and personal expenditure, health-related quality of life and capability wellbeing to assess cost-effectiveness over the trial and modelled lifetime. Cost-utility analysis examines cost per quality-adjusted life years gained over 24 months. Mixed method process evaluation explores participants' experiences and contextual factors impacting outcomes and implementation. Stakeholder interviews examine scale-up and implementation.

Ethics and dissemination

Ethical approval obtained before data collection (West of Scotland Research Ethics Service REC 4 22/WS/0003). Results will be published via a range of outputs and audiences.

Trial Registration [2a]: ISRCTN16299220 (prospectively registered).

For peer review only

For peer review only - http://bmjopen.bmj.com/site/about/guidelines.xhtml

Strengths and Limitations

- This is the first trial to examine the effectiveness and cost-effectiveness of a behavioural intervention for supporting weight loss in postpartum women with overweight or obesity that is delivered solely by text messages.
- The trial tests a novel evidence and theory-based text message intervention with embedded behaviour change techniques that is fully automated and incorporates two-way messaging to encourage engagement and delivery of specific behaviour change techniques.
- Researchers conducting participant recruitment and outcome data collection are blind to group allocation. Any situations where blinding is not maintained are recorded and reported. Due to the nature of the intervention, it is not possible to blind participants to randomisation.
- The trial includes an active control comparator to minimise disappointment bias and attrition related to randomisation experienced in previous weight loss intervention trials.(1)
- Home visits by the research team are offered to participants for data collection to facilitate participation in research assessments, helping to overcome barriers to taking part and address issues in participant retention seen in other postpartum trials.

For peer review only - http://bmjopen.bmj.com/site/about/guidelines.xhtml

Administrative information

This protocol is written in line with the SPIRIT checklist,(2) with recommended content indicated by the numbers in square brackets after section titles.

Title [1]: Effectiveness and cost effectiveness of a 12 month automated text message intervention for weight management in postpartum women with overweight or obesity: protocol for the Supporting MumS (SMS) multi-site, parallel-group, randomised controlled trial.

Protocol version [3]: Version 3.0 dated 10th May 2023.

Trial status: Opened to recruitment in April 2022, with the first participant randomised in May 2022 and participant recruitment completed in May 2023. The trial is in follow-up until May 2025, with primary outcome data collection to be completed in May 2024.

Table 1: Author details [5a]

Author details	Dr Dunla Gallagher<br>Supporting MumS Trial Manager<br>Centre for Public Health, Queen's University Belfast, ICSB, Grosvenor Road, Belfast, BT12 6BA<br>d.gallagher@qub.ac.uk<br>Tel: 07341 888415
	Dr Eleni Spyreli<br>Postdoctoral Research Fellow<br>Centre for Public Health, Queen's University Belfast, ICSB, Grosvenor Road, Belfast, BT12 6BA<br>Eleni.spyreli@qub.ac.uk<br>Tel: 028 9097 1608

For peer review only - http://bmjopen.bmj.com/site/about/guidelines.xhtml

	Prof Annie Anderson<br>Professor of Public Health Nutrition<br>Division of Population Health and Genomics, University of Dundee<br>a.s.anderson@dundee.ac.uk<br>Tel: +44 (0)1382 383299
	Ms Sally Bridges (PI SMS Bradford)<br>Programme Coordinator<br>Better Start Bradford Innovation Hub, Bradford Teaching Hospitals NHS Foundation Trust<br>sally.bridges@bthft.nhs.uk<br>Tel: +44 (0)1274 383914
	Prof Chris Cardwell<br>Professor of Medical Statistics<br>Centre for Public Health, Queen's University Belfast<br>c.cardwell@qub.ac.uk<br>Tel: +44 (0)28 9063 2620
	Dr Elinor Coulman (PI SMS Cardiff)<br>Research Fellow<br>Centre for Trials Research, Cardiff University<br>johne1@cardiff.ac.uk<br>Tel: +44(0)29 2068 7624
	Assoc Prof Stephan Dombrowski<br>Associate Professor in Health Psychology<br>Faculty of Kinesiology, University of New Brunswick, Canada<br>stephan.dombrowski@unb.ca

For peer review only - http://bmjopen.bmj.com/site/about/guidelines.xhtml

	Tel: +1 506 453 4803
	Prof Caroline Free (PI SMS London)<br>Professor of Primary Care and Epidemiology<br>Maternal, Adolescent, Reproductive & Child Health,<br>London School of Hygiene & Tropical Medicine<br>caroline.free@lshtm.ac.uk<br>Tel: +44 (0)20 7958 8109
	Dr Suzie Heaney<br>Personal and Public Involvement (PPI) Representative<br>suzieheaney@gmail.com
	Prof Pat Hoddinott (PI SMS Scotland)<br>Chair in Primary Care<br>NMAHP Research Unit, University of Stirling<br>p.m.hoddinott@stir.ac.uk<br>Tel: +44 (0)1786 466394
	Prof Frank Kee<br>Professor of Public Health<br>Centre for Public Health, Queen's University Belfast<br>F.Kee@qub.ac.uk<br>Tel: +44 (0)28 9097 8943
	Ms Cliona McDowell<br>Head of Statistics<br>Northern Ireland Clinical Trials Unit (NICTU)<br>Cliona.mcdowell@nictu.hscni.net<br>Tel: +44 (0)28 9615 1447
	Prof Emma McIntosh<br>Professor of Health Economics

For peer review only - http://bmjopen.bmj.com/site/about/guidelines.xhtml

	Institute of Health and Wellbeing, University of Glasgow<br>emma.mcIntosh@glasgow.ac.uk<br>Tel: +44 (0)141 3302455
	Prof Jayne Woodside<br>Professor of Human Nutrition<br>Centre for Public Health, Queen's University Belfast<br>j.woodside@qub.ac.uk<br>Tel: +44 (0)28 9097 8942
	Prof Michelle McKinley (CI and PI SMS Belfast)<br>Professor of Nutrition<br>Centre for Public Health, Queen's University Belfast<br>m.mckinley@qub.ac.uk<br>Tel: +44 (0)2890978936
Name and contact information for the trial sponsor [5b]	Dr Paula Tighe<br>Research Governance, Ethics and Integrity Manager<br>Queen's University Belfast<br>p.tighe@qub.ac.uk<br>Tel: +44 (0)28 9097 3861
Role of sponsor and funder [5c]	The sponsor and funder are not responsible for the trial design, collection, management, analysis or interpretation of data or report writing. However, they oversee the trial progress, including publications.

For peer review only - http://bmjopen.bmj.com/site/about/guidelines.xhtml

Introduction

Background [6a]

This trial addresses the development of overweight and obesity across the childbearing years. Entering pregnancy with a high body mass index (BMI) increases many health risks for these mothers and their babies, including gestational diabetes, hypertensive disorders, caesarean section and birth complications, compared with recommended weight women.(3, 4, 5, 6) Excessive gestational weight gain (GWG) and postpartum weight retention (PPWR) are common (7, 8) and many women are at risk of further weight gain across the extended postpartum period.(9) By 18 months postpartum, about one in five women have moved into a higher BMI category (8) which increases the risk of complications in subsequent pregnancies and contributes to long-term overweight and obesity.(9)

Effective and appropriate weight management interventions in women during the postpartum period, which account for the well-recognised barriers for new mums such as time constraints and childcare, are lacking.(9, 10, 11, 12, 13, 14) Previous intervention studies are characterised by poor recruitment and high rates of attrition (1, 15, 16, 17, 18) and have not adequately considered the difficulties in reaching this population and specific barriers to lifestyle behaviour change that come with having a baby.(19, 20, 21, 22, 23) In-person and structured weight management approaches, such as those delivered in community and group settings, are unlikely to be successful with this population due to the commitment required, and such approaches may exacerbate health inequalities.(24, 25, 26, 27) More appropriate ways of recruiting and engaging with postpartum women to achieve sustained behaviour change are required (1, 9) and a need to evidence cost-effectiveness to inform implementation.(12)

Recent evidence, mostly from feasibility or pilot studies, indicates that technology usage shows promise in postpartum weight management, perhaps because this form of delivery fits

more seamlessly with women's lives at this stage.(17, 28, 29, 30) Mobile technologies can offer a more flexible and individualised 'any time, any place' approach to behavioural weight management interventions.(9) Text message interventions have high reach potential and allow for flexible scheduling and interactivity,(31) potentially making them convenient for mums with limited time and helpful for overcoming health inequalities. Systematic reviews support the use of text messaging in behavioural weight management interventions but indicate further evaluation is required.(32, 33, 34, 35, 36). They are cost-effective and readily scalable for changing other health behaviours.(37, 38)

The Supporting MumS (SMS) intervention, consisting of a library of text messages, was developed with personal and public involvement (PPI) to support weight management in the postpartum period.(28) Messages focus on diet and physical activity with embedded behaviour change techniques (BCTs) informed by behaviour change theory and evidence. In a previous pilot evaluation, one hundred women with overweight or obesity from Northern Ireland (NI), who had a baby within the previous 24 months, were recruited and randomised to receive text messages about weight loss (intervention group) or child health and development (active control) for 12 months. The study demonstrated the feasibility and acceptability of the intervention and an independent Trial Steering Committee (TSC) judged that the pre-specified progression criteria to proceed to a full randomised controlled trial (RCT) (i.e., successful recruitment, high retention and no differential attrition, high acceptability of the intervention, and evidence of positive indicative effects) were met.(28)

Rationale [6a]

This RCT addresses the following research question: Is an automated 12 month text message intervention, designed to support weight loss and weight loss maintenance for postpartum women with overweight or obesity, effective and cost-effective for weight loss at 12 months, compared with an active control group receiving text messages on child health and development?

For peer review only - http://bmjopen.bmj.com/site/about/guidelines.xhtml

SMS is a parallel, 2-arm multi-site RCT using automated and bidirectional text messages to support weight management over a 12 month period in postpartum women with overweight or obesity who have had a baby in the last 24 months, comparing weight change at 12 months from baseline between the intervention and active control groups. This RCT protocol is based on the pilot RCT methodology, but changes were to remove: 1) use of a sealed pedometer to assess physical activity as this was not acceptable to women and, 2) a discussion forum intervention component as it was not used by pilot RCT participants. We added considerations for data collection in the event of COVID-19 restrictions. In moving from a single site pilot study to a multi-site RCT recruiting an ethnically and socioeconomically representative sample, we conducted a six-month period of intervention and recruitment method adaptation, with PPI, ensuring the broad acceptability and cultural relevance of the messages and enhancing the generalisability of the findings to different groups of women across the United Kingdom (UK), thus informing implementation.

The increased prevalence of obesity, its associated complications and weight management services, is costly to the National Health Service (NHS). NHS costs associated with caring for a pregnant woman with a BMI of ≥25 kg/m^2 are up to 37% higher than caring for a woman with a healthy BMI.(39) Both National Institute for Health and Care Excellence (NICE) guidance (13) and an associated review of the cost-effectiveness of weight management interventions following childbirth,(40) outline the potential of postpartum interventions to be a cost-effective way of reducing the long-term risks of obesity, heart disease, cancer and diabetes, and recommend health professionals should advise women within two years of having a baby to eat a nutritious diet and keep active to encourage postpartum weight reduction.(13, 41)

Postpartum weight management interventions combining dietary and activity behaviour change have moderate positive influence on maternal weight (12, 15) and those including self-regulatory BCTs are likely to be more successful.(10, 12) The postpartum period could

For peer review only - http://bmjopen.bmj.com/site/about/guidelines.xhtml

be an ideal time to intervene to shape new health behaviours as women may be motivated and receptive to health information.(42) However, women highlight a need for additional weight management support during the postpartum period as little is currently provided.(43)

The present trial allows women to take part up to 24 months postpartum. Qualitative evidence suggests that every woman's postpartum journey is different (19, 20, 21, 22, 23) and the optimal time to engage women in postpartum weight management is currently unknown.(9, 11) Allowing this extended inclusion window accommodates different maternal weight trajectories across this time.(44, 45) Furthermore, women may prepare for another pregnancy during this period, so there is potential to support better health for subsequent pregnancies.(9)

Mobile phone usage and ownership are widespread (95% of UK households) amongst all sectors of society, irrespective of socioeconomic status.(31) Text messaging is a simple communication mode that does not require smartphone or tablet technology, unlike web-based interventions or apps. This supports health equity through high reach potential at a low cost, making it conducive with scale-up and implementation, unlike some weight loss interventions which can be resource intensive and expensive (46) and can exacerbate health inequalities.(47, 48) A text messaging intervention can be delivered flexibly and on a sustained basis, can be reactive and proactive, and does not necessarily rely on participant initiation.(31, 49) Using the real-time advantages offered by mobile technologies to deliver behavioural weight management support to postpartum women has the potential to encourage behaviours that may improve maternal health in both the short and long-term.(50) Trial results are relevant for other population groups given the possible advantages of this weight management approach. The NHS aims to embrace the potential of digital strategies for improving UK health (51) and text message interventions could complement any weight management support currently delivered across the NHS, to help ease the current burden on an over-stretched service.

For peer review only - http://bmjopen.bmj.com/site/about/guidelines.xhtml

The SMS trial adopts a fully automated approach to intervention delivery whilst still offering feedback to participants. The trial makes a novel and important contribution to the field of behavioural text message interventions in a number of ways: there are currently few such interventions that are fully automated, where text messaging is the main mode of delivery and two-way messaging is used to encourage engagement and delivery of specific BCTs; there are also few which consider both weight loss and weight loss maintenance, last 12 months or more and use an active control to minimise disappointment bias.(28)

The National Institute of Health and Care Research (NIHR) awarded further funding to explore postnatal mental health outcomes and needs to inform application of the priorities in the NHS Long Term Plan via existing studies. We added data collection methods related to mental health (postnatal depression, depression and anxiety, mother and child relationships, service and treatment experiences) in February 2022, allowing an opportunity to examine poor postnatal mental health prevalence rates, trajectories, predictors, inequalities and service needs, in an ethnically and socioeconomically diverse sample from all four UK countries. The trial also allows further validity and reliability testing of the Me and My Baby (MaMB)/Me and My Child (MaMC) screening tool which was co-produced by health visitors and the community to assess the mother and child relationship. Funding for this workstream was contracted in March 2022.

Explanation for the choice of comparators [6b]

An active control comparator is used as pilot study findings indicate that this design aspect supports good participant retention, acceptability and satisfaction, where previous weight loss trials with postpartum women have reported high levels of attrition and disappointment bias in those allocated to no treatment or usual care control groups.(1) Active control participants are assessed for the same outcome measures as the intervention group at 6, 12 and 24 months. We advertise the purpose of the trial as testing two new text message services to minimise women's preference for one group over another. The participant

information sheet (PIS) clearly describes that women have a 50:50 chance of being assigned to receive messages about child health and development or messages about weight management, thus preserving participant autonomy.

Objectives [7]

Primary objective

To conduct a 2-arm parallel-group RCT comparing weight change at 12 months for postpartum women with overweight or obesity who receive text messages about weight management with an active control.

Secondary objectives

- To assess differences between-groups in secondary outcomes.
- To assess the cost-effectiveness of the SMS intervention compared with an active control comparator.
- To assess the prevalence and trajectories of postnatal mental health in women across the UK, particularly those from marginalised groups, including assessment of the mother-child relationship.
- To conduct a process evaluation to explore women's experiences of the intervention, the pathways through which the intervention effects are mediated and contextual factors affecting the outcomes or future implementation of the intervention.
- To seek permission for routine data linkage for long term health outcomes (mother and youngest child).
- To conduct a follow-up of women at 24 months (12 months after the intervention has ceased) to examine the long-term effect of the intervention and to conduct interviews with stakeholders to explore scale-up and implementation; this stage is contingent upon TSC and NIHR assessment of the 12 month primary outcome data and any other core

outcome data required to make a fully informed decision about effectiveness and the clinical significance of weight change.

Trial Design [8]

A pragmatic, multi-site, parallel, 2-arm, assessor-blind, 1:1 superiority RCT comparing weight change at 12 months for the SMS intervention and active control groups, with mixed methods process evaluation, cost-effectiveness modelling, consent for future data linkage to longer-term health outcomes for mothers and babies, 24 month participant follow-up (i.e. 12 months post-intervention) and implementation consultations with stakeholders.

Methods and analysis

Trial setting [9]

A multi-site trial with five recruitment sites in the UK: 1) Belfast, Northern Ireland (trial co-ordinating site); 2) Stirling, Scotland; 3) Cardiff, Wales; 4) London, England; and 5) Bradford, England. Recruitment sites were selected to target geographical areas of ethnic and socioeconomic diversity for the purposes of participant recruitment via community and NHS pathways.

Eligibility criteria [10]

Women (in accordance with the NICE Postnatal care guideline NG1941, the term 'woman' used in this trial includes people who do not identify as women but who are pregnant or have given birth) are eligible to participate in the trial if they are aged 18 years or over, have a BMI of ≥25 kg/m^2, have had a baby within the last 24 months and own a mobile phone to allow them to receive personal text messages.

Women are excluded if their baby is less than 6 weeks old or if they have insufficient English language to understand short written messages, if they have had or plan to have any type of weight loss surgery, if they report having ever received a diagnosis of anorexia nervosa or bulimia from a doctor, or, if they are on a specialist diet and receiving dietetic care. Women are excluded if they are participating in any other weight management research study/programme currently or in the previous three months. Women are not eligible if they are pregnant and any consented participant becoming pregnant during the trial is excluded, as the intervention has not been designed for use in pregnancy.

Interventions

Supporting MumS (SMS) intervention [11a]

A full description of the SMS intervention development has been reported elsewhere.(28) The intervention is described here in accordance with the Template for Intervention Description and Replication (TIDieR) guidance.(52) The intervention group receive fully automated text messages, including bidirectional and interactive features, about weight loss and maintenance of weight loss for 12 months. The evidence and theory-based intervention consists of a library of text messages focused on diet and physical activity with embedded BCTs known to be positively associated with weight management. The intervention logic model and sample intervention and active control text messages are available in supplemental appendix 1. Examples of how BCTs were incorporated into the messages is available elsewhere.(28)

Why- theory and components

The intervention is based on the Health Action Process Approach (HAPA) and a systematic review of over 100 behavioural theories which synthesised theoretical explanations for maintenance of behaviour change.(53) The intervention encourages a self-guided approach

For peer review only - http://bmjopen.bmj.com/site/about/guidelines.xhtml

to weight-related behaviour change, as supported by the literature.(54, 55, 56, 57) The intervention focuses on dietary intake and physical activity to address energy balance related behaviour, with embedded evidence-based BCTs specifically linked to the relevant phases and psychological processes of behaviour change alongside consideration of barriers for this population.(28)

The messages adopt a friendly tone including humour to foster engagement and provide information, practical tips and advice including quotes from other mothers. They signpost to external resources and provide encouragement and motivation, discourage guilt and promote self-reflection. A core library of text messages (n= 353) to be delivered by the automated system was created. These include a weekly text message asking women to self-weigh and report their weight (n=50) and bidirectional messages to encourage engagement, self-monitoring, relapse prevention and to provide feedback, as follows: 1) “Yes/No” questions (n=36) which trigger an automated response to the participant based on their reply, 2) trigger words (‘slip-up’; ‘crave’; ‘bad day’ or ‘tired’) which can be texted by women at any time to prompt a reply designed to address these barriers and prevent relapses, 3) During months 7-12 when the focus shifts to maintenance strategies, the weekly text message asking women to report their weight asks them to add the word ‘up’ or ‘down’ or ‘same’ in relation to how their weight compares with the week prior. These keywords trigger an automated response. Responses for bidirectional messages are sent from pre-loaded message banks and are not individually scripted or tailored.

Women can also opt-in to receive messages related to weight management when breastfeeding (n=10) or stopping smoking (n=15). These messages are intended to alleviate participant concerns in relation to weight management whilst breastfeeding and to address vulnerabilities around weight gain during smoking cessation.

For peer review only

For peer review only - http://bmjopen.bmj.com/site/about/guidelines.xhtml

What- materials and procedures

The intervention consists of 353 core messages delivered to all women allocated to this group, in addition to messages associated with the interactive and optional components described above.

Social support is facilitated throughout the intervention using a "buddy system" (informed by a successful smoking cessation text message intervention (38)) where a participant can nominate a friend or family member to receive the same messages they are receiving, putting that person in a better position to offer support. Participants are sent instructions and reminders on how to do this within the core messages, along with information on the value that support can play in weight loss success. A 'buddy' can be requested at any stage throughout the intervention period.

Who- intervention provider

Intervention costs are provided by the NI Public Health Agency (PHA). Intervention delivery is fully automated via the existing pilot study text message platform developed by the London School of Hygiene and Tropical Medicine (LSHTM). The intervention package is designed so that it can be readily delivered by other text message providers to facilitate future implementation by the NHS or public services.

How - mode of delivery

Message exchange with participants is managed via a secure server at the LSHTM, using Firetext (58) to distribute messages to participants.

Where– setting

For peer review only - http://bmjopen.bmj.com/site/about/guidelines.xhtml

The intervention can occur any place and any time. Participants require a mobile phone with the function to receive text messages.

When and how much

Text message frequency starts at 14-15 sent per week during months one and two, tapering to 9-10 per week in months three to six. Fewer messages, four to five per week, are sent in the weight loss maintenance phase (months 7 to 12) when the emphasis shifts to reinforcing self-regulation techniques developed during the first six months of the intervention and encouraging maintenance relevant strategies such as relapse prevention.

The system delivers messages any time between 10am and 11pm, with messages programmed to arrive at varied times to avoid predictability. Participants can choose to block an additional period of the day that they want to be kept message-free e.g., 10am to 5pm. Following randomisation, women allocated to the intervention group are programmed to start receiving messages on the subsequent Monday which schedules the weekly message asking participants to report their weight to be sent on Fridays.

Active control [11a]

The active control messages were also developed by the trial team with PPI input (28), for mothers with babies aged six weeks up to 36 months (based on participant inclusion criterion). The messages relate to general childcare and development, with content consistent with evidence-based information provided by the NHS Start4life service (59) including weekly play ideas/activities and information on milestones, home safety, separation anxiety and similar topics. Active control messages do not address the intervention target or active ingredients (i.e. diet, physical activity and BCT content related to weight loss) nor do they prompt bidirectional functionality. Women receive three messages each week over 12

For peer review only - http://bmjopen.bmj.com/site/about/guidelines.xhtml

months with messages corresponding to the age of their baby, i.e., if their baby is six months old at randomisation, the participant starts to receive messages corresponding to this age. Women allocated to the control group are offered to receive a booklet summarising the intervention content on completion of the trial.

Criteria for discontinuing or modifying allocated interventions [11b]

Participants in both groups have the ability to pause, stop and restart the messages throughout by sending an instruction to the text system e.g., ‘STOP’, ‘START’. Researchers ask women choosing to discontinue the messages to provide any reason for doing so, to inform the process evaluation. Participants discontinuing messages remain in the trial unless they formally withdraw from the trial assessments.

Strategies to improve adherence to interventions [11c]

Text message delivery is fully automated and centrally delivered via the LSHTM system which is intended to ensure treatment fidelity. Regular monitoring of the text message system is completed by the system developer and trial team members (CI and clerical officer) to check the content of sent and received messages (e.g., ensure web links are working, review spontaneous participant responses). Intervention engagement is assessed via text messages received from participants in response to the weekly weight prompt and the replies to bidirectional messages as described in [11a], self-report items in questionnaires at 6, 12 and 24 months and qualitative interviews at 6 and 12 months.

Relevant concomitant care permitted or prohibited during the trial [11d]

For peer review only - http://bmjopen.bmj.com/site/about/guidelines.xhtml

Women who become pregnant during the trial will be excluded, as text message content has not been designed for pregnancy and primary outcome data (weight) can only be collected from women who are not pregnant. This is a self-directed intervention and women are permitted to access any other weight-loss services via NHS, voluntary or private sector pathways. Details of engagement with other services are captured at each data collection timepoint. Any weight loss surgery undergone by participants during the trial, and prescribed or over-the-counter medications that may affect weight, are documented at 6, 12 and 24 months to assess the impact on trial outcomes.

Outcomes [12]

Trial outcomes, measures and the assessment schedule at 0, 6, 12 and 24 months are outlined in Table 2.

- *Primary outcome:* between-group difference in mean weight change (kg) from baseline to 12 months.
- *Secondary outcomes:* between-groups difference in mean weight change (kg) from baseline to 24 months and between-groups differences in mean waist circumference (cm), mean BMI (kg/m^2), in the proportions of women gaining a substantial amount of weight (>5kg), dietary intake (Fat and Fibre Barometer plus questions on sugar intake), physical activity (IPAQ-SF) and infant feeding practices (Infant Feeding Survey), by the 12 and 24 month follow-up timepoints respectively.
- *Health economic outcomes:* NHS resource use and personal expenditure (GP usage, hospitalisations, prescribed and over-the-counter medications, lifestyle products and services, expenditure on food and drink, alcohol and smoking products); health-related quality of life (EQ-5D-5L with visual analogue scale) and Capability (ICECAP-A).

- *Process Evaluation:* to explore participants' experiences and interactions with the intervention by site, ethnicity and socioeconomic status, including evaluating dose (number of women who choose to receive all text messages and those who pause or stop the messages), engagement with the intervention (text message responses and qualitative data), reach (trial records of recruitment, loss to follow-up and withdrawal) and acceptability (participants' satisfaction ratings and qualitative data).
- *Exploratory outcomes:* moderator and mediator analysis (Table 2)
- *Qualitative sub-study:* telephone interviews with a purposive sample of participants at 6 and 12 months to understand the experiences of women receiving the intervention and active control text messages and the contextual factors related to engagement including postnatal mental health, to inform trial and process evaluation outcomes.
- *Stakeholder consultation:* if the intervention is shown to be effective at 12 months, stakeholder interviews will be conducted to explore factors relevant to intervention scalability and implementation, including any further work needed to develop a coherent implementation model.

For peer review only - http://bmjopen.bmj.com/site/about/guidelines.xhtml

413 **Table 2: Supporting MumS trial assessments**

Outcome collected	Measure/equipment used	Time point (month)			
		0	6	12	24
Anthropometric measures and Demographics*					
Height (m)	Portable standing stadiometer (Seca 213, Birmingham, UK)	✓			
Body weight (kg) (primary endpoint)	Calibrated scales (Seca 875, Birmingham, UK)	✓	✓	✓	✓
Waist circumference (cm)	Tape measure (Seca 201, Birmingham, UK)	✓	✓	✓	✓
Demographic characteristics**	Trial-specific questions	✓			
Acceptability outcomes					
Satisfaction with text messages¥	Trial-specific questions		✓	✓	
Acceptability of intervention, active control and study methods¥	Trial-specific questions			✓	
Longer-term use of text messages¥	Trial-specific questions				✓
Qualitative interviews	-		✓	✓	
Interviews with stakeholders	-				✓
Economic evaluation - Within trial health and social care cost data¥					
Health service resources use	Questions on health resource usage	✓	✓	✓	✓
Medication usage	Questions on over-the-counter medications	✓	✓	✓	✓
Healthy lifestyle-related costs	Questions on personal expenditure	✓	✓	✓	✓
Exercise-related costs	Questions on costs of exercise	✓	✓	✓	✓
Food and drink costs	Questions on weekly cost of groceries, alcohol and smoking products	✓	✓	✓	✓
Change of employment status	Trial-specific questions	✓	✓	✓	✓
Health-related quality of life	EQ-5D-5L (60)	✓	✓	✓	✓
Capability well-being	ICEpop CAPability measure Adults (ICECAP-A)(61)	✓	✓	✓	✓
Secondary outcome measures¥					
Dietary intake	Fat and fibre barometer(62)	✓	✓	✓	✓
Sugar intake	Trial-specific questions	✓	✓	✓	✓
Alcohol consumption	Trial-specific questions	✓	✓	✓	✓
Physical activity	International Physical Activity Questionnaire (IPAQ) - Short form(63)	✓	✓	✓	✓
Infant feeding	Infant feeding survey(64)	✓	✓	✓	✓
Moderators of intervention effect¥					
Mental health – depression	Edinburgh Postnatal Depression Scale (EPDS)(65)	✓	✓	✓	✓
Mental health - anxiety	Generalised Anxiety Disorder (GAD-7)(66)	✓	✓	✓	✓
Mother and child relationship	MaMB/MaMC(67) and Mothers Object Relations Scale (MORS-Baby/Child)(68)	✓	✓	✓	✓
Sleep	Pittsburgh Sleep Quality Index(69)	✓	✓	✓	✓
Confidence/importance/desire for weight loss and maintenance	Trial-specific questions	✓	✓	✓	✓
Mediators of intervention effect¥					

For peer review only - http://bmjopen.bmj.com/site/about/guidelines.xhtml

Intention and self-efficacy for diet and physical activity	Health Action Process Approach (HAPA) including action and coping planning and self-efficacy (70, 71)	✓	✓	✓	✓
Habit behaviour	Self-report behavioural automaticity index(72)	✓	✓	✓	✓
Self-regulation of eating behaviour	Self-regulation of eating behaviour questionnaire(73)	✓	✓	✓	✓
Weight self-monitoring	Trial-specific questions	✓	✓	✓	✓
Diet and exercise monitoring	Trial-specific questions	✓	✓	✓	✓
Goal-setting for diet and exercise	Trial-specific questions	✓	✓	✓	✓
Weight loss motivation	Motivation for weight loss scale(74)	✓	✓	✓	✓
Taking part in other weight loss programme	Trial-specific questions	✓	✓	✓	✓
Social support	Social support for eating & exercise(75) plus general social support (BiB trial-specific questions)	✓	✓	✓	✓
Self-esteem	Rosenberg Self-Esteem Scale(76)	✓	✓	✓	✓

**Recorded in case report form (CRF).*

***Date of birth, where they heard about the trial, NHS number, ethnicity, income, employment, education, relationship status, weeks postpartum at trial entry, parity, medical information (disability status, medication use), infant feeding, weight history, smoking status, alcohol intake, technology usage*

¥Recorded in participant self-reported questionnaires.

<u>Participant timeline [13]</u>

The trial flowchart includes the participant timeline for the RCT (Figure 1).

<u>Sample size [14]</u>

The target sample size is 888 women over 12 months, with site-specific targets across the five geographical areas (London and Bradford, n=189, respectively; Belfast, Stirling and Cardiff, n=170, respectively). Pregnancy-related withdrawals are not replaced unless the loss to follow-up (12%) or pregnancy rates (15%) observed in the pilot RCT (28) are exceeded by the 6 month timepoint, which is monitored during Project Management Team (PMT) meetings.

For peer review only - http://bmjopen.bmj.com/site/about/guidelines.xhtml

Sample size calculation

The pilot trial showed a mean weight loss of 1.75kg in the intervention group compared with a mean gain of 0.19kg in the active control group between baseline and 12 months, equating to a between-groups difference in mean weight change, adjusted for baseline, of -1.67 kg (95% CI -4.88 to 1.55)).(28) Based on pilot data for the active control group and a standard deviation of 7.5kg, 594 completing participants (297 per group) are required to give the trial over 90% power to detect a statistically significant difference of 2kg in mean weight change from baseline, at the 5% level, between-groups. A mean difference of 2kg is accepted as being associated with metabolic health benefits and is often used to power weight loss studies.(57)

We observed a 15% pregnancy dropout rate in the pilot study, based on a 99% white ethnicity population.(28) Data from South Asian women in the Born in Bradford's Better Start (BiBBS) cohort (77) indicates that pregnancy dropout rates could be higher in more ethnically diverse samples, around 22%. To have 119 participants completing per site, and accounting for a loss to follow-up rate of 15% and higher anticipated pregnancy exclusion rates in areas with greater ethnic diversity, the following site-specific sample sizes were calculated: 1) Belfast, Stirling and Cardiff; 15% pregnancy rate + 15% loss to follow-up = 170 women, 2) Bradford and London; 22% pregnancy rate + 15% loss to follow-up= 189 women. Therefore, the total sample size for the trial is 888 women (444 per group).

Recruitment [15]

Based on successful pilot study recruitment processes,(28) trial information is disseminated via community settings likely to be attended by mothers of young children (e.g., parent and

For peer review only - http://bmjopen.bmj.com/site/about/guidelines.xhtml

toddler groups, libraries, breastfeeding support groups), social media and through contacts with healthcare professionals and GP practices, including both routine appointments and contacting potentially eligible women identified using patient database searches. A range of recruitment pathways are used to target ethnically and socioeconomically diverse populations.

In Bradford, principal trial recruitment is through BiBBS, an existing birth cohort study, with trial information disseminated to participants who previously consented to be informed of other relevant research studies.

Monitoring recruitment progress

Based on the pilot study recruitment rate,(28) we plan to recruit the target sample over a 12 month period. We employ a recommended traffic light system (Figure 1) to review trial progression at the mid-point of the recruitment period,(78) with the following actions: proceed if >50% of target achieved; modify recruitment approach if 25-49% achieved and consider stopping (discuss with funder and TSC) if <25% of target recruited.

Screening [10]

All trial information materials disseminated via described recruitment pathways, direct women who are potentially interested in taking part to contact the research team via telephone, email or by accessing the trial website/scanning a QR code to submit a secure pro-forma with their contact details.

Site researchers contact women expressing an interest in their area to provide a PIS, privacy notice and copy of the consent form (supplemental appendix 2) via email or post. Audio versions of the PIS and privacy notice are available on the trial website to increase accessibility. To comply with the Welsh Language Act 1993, Welsh language versions of the PIS and Consent Forms are available, on request. Women are given at least 48 hours to

For peer review only - http://bmjopen.bmj.com/site/about/guidelines.xhtml

consider the information before being contacted again by telephone by the research team to discuss the trial procedures and any queries then invited to take part. Up to three contact attempts are made to invite all women expressing interest for screening. BMI eligibility is initially determined using women's self-reported height and weight but later measured and verified by the researcher prior to collecting informed consent. Women who are deemed ineligible due to their baby being less than six weeks old will be invited for rescreening when they reach six weeks postpartum.

Informed consent and withdrawal [26a]

For women meeting the eligibility criteria and willing to proceed with trial participation, a baseline researcher visit is arranged. Home visits are the primary approach to data collection, with women also offered the option of attending another venue if preferred (University building, community venue or their workplace).

Research staff, trained in Good Clinical Practice (GCP) guidelines, obtain written informed consent prior to collecting baseline data. At the baseline visit, women are offered the opportunity to ask further questions and confidentiality of data is explained. Informed consent is then taken. Women are reminded that they have the freedom to choose whether to take part or not. If willing to proceed, women are asked to initial and sign the trial consent form, with optional consent to be invited for telephone interview participation at 6 and 12 months. During the 6 and 12 month trial visits, women providing this optional consent at baseline are asked if they are still happy to be contacted to take part in a telephone interview. Participants completing telephone interviews are asked to provide their verbal consent for audio recording and for anonymised quotations to be used in trial publications before commencing interviews.

For peer review only - http://bmjopen.bmj.com/site/about/guidelines.xhtml

Once consented, participants are advised that they can withdraw from the trial at any time, without giving a reason, should they wish. Withdrawals are documented in the CRF along with any reason given for withdrawing. Participants excluded due to pregnancy are given the option to continue receiving the text messages. Anyone choosing this option needs to complete and return a pregnancy form to acknowledge that the messages have not been designed for pregnancy but may be useful after pregnancy. Data collected up to the point of withdrawal is retained for analysis unless the participant specifies otherwise.

Additional consent provisions for collection and use of participant data and biological specimens [26b]

Optional aspects of trial consent include permission to be informed of future follow-up to this trial or other relevant research studies and permission for future data linkage to routinely collected health records for long term health outcomes (mother and youngest child) which is not within the current trial timeline. No biological specimens are collected.

Assignment of interventions: allocation

Sequence generation [16a]

Participants are randomised in blocks of size 4 and randomisation is stratified by site, with the allocation sequence generated in STATA (using ralloc) by an independent statistician.

Concealment mechanism and allocation implementation [16b and 16c]

The allocation sequence is sent directly to the platform manager at LSHTM for upload to the secure web-based text message delivery system. After collecting consent and baseline data, site researchers enrol participants using the LSHTM randomisation system linked directly to the text message platform, implementing the random allocation sequence by accordingly sending automated intervention or control messages to the participant. Participants become aware of their group allocation when they start to receive the text messages; researchers do not have access to this information.

Blinding [17a]

Researchers responsible for recruitment and collecting outcome data are blinded to group allocation. It is not possible to blind participants, but they are requested not to discuss the text messages they are receiving with researchers at each visit. This worked well in the pilot study with blinding maintained.(28) All statistical analyses are conducted blinded to group allocation until analyses of the 12 month data point are complete at which point the randomisation sequence is provided by the independent statistician who prepared it. We record any circumstances in which unblinding has occurred. The trial clerical officer, Chief Investigator and LSHTM text message system developer are not blind; these individuals see what group individuals are assigned to on the text message system for the purposes of monitoring the intervention delivery and the clerical officer is responsible for preparing the group-specific 12 month questionnaires. Blinded researchers do not see these questionnaires until after primary outcome data is collected. Interviews at 6 and 12 months are carried out by a researcher who has not had any previous contact with the participant. Group allocation information for that participant is provided to the researcher before the interview so the correct interview guide can be used.

For peer review only - http://bmjopen.bmj.com/site/about/guidelines.xhtml

Emergency unblinding [17b]

Given the nature of the intervention, emergency unblinding is not required.

Data collection, management and analysis

Data collection methods [18a]

The details and schedule of trial assessments (Table 2) are based on the pilot RCT.(28) Assessments take place at baseline (before randomisation), 6 months, 12 months (end of intervention period) and 24 months (12 months after intervention end).

Screening is completed according to the trial eligibility criteria and guided by and documented in a trial screening sheet.

Researchers, trained in data collection processes, collect outcome data during visits to women's homes (or another venue of the participant's choosing e.g., University/Community venue). Height, weight and waist circumference are measured by the researcher using standardised protocols and calibrated scales. A hard board is available to place under the scales on uneven surfaces. Baseline demographic information is collected by the researcher. Health service identification number is collected from any woman who provides consent for future data linkage. All remaining outcome data including health economic outcomes are collected via participant self-report questionnaires, either on paper (during the visit or a hard copy left with the participant along with a paid return envelope) or online (link to questionnaire hosted on Qualtrics survey software sent to participant and managed by the trial coordinating team in Queen's University Belfast (QUB)), based on participant preference at each visit. Researchers offer any required assistance with paper questionnaire completion at the visit or via telephone.

For peer review only - http://bmjopen.bmj.com/site/about/guidelines.xhtml

Interviews are conducted with approximately 50 women at each timepoint, with participants purposively sampled to include ethnic, geographical and socio-demographic diversity (i.e., including a spread across trial sites, ethnicity, income level, parity, mental health status, randomised groups and intervention engagement e.g., those who have discontinued messages). Interviews follow a semi-structured interview guide and a translator can be used when any woman would prefer to be interviewed in a language other than English.

Text message engagement data is captured by the LSHTM system, including all messages sent and received organised by participant ID and date.

If the intervention is shown to be effective at 12 months, we will invite a range of relevant stakeholders (e.g., midwives, GPs, PPI, commissioners, policymakers) identified from research team and TSC networks to take part in interviews to explore factors relevant to engagement, scale-up and implementation.

Qualitative data is collected from participants and stakeholders using telephone/Microsoft teams interviews, which are audio-recorded and transcribed.

Plans to promote participant retention and complete follow-up [18b]

Home visits are offered for data collection to facilitate women's participation in research assessments, although visits can take place elsewhere if preferred by the participant. Visits are flexibly arranged for a date and time of convenience for the participant. At the end of each visit, the appointment for the next visit is scheduled if possible. Participants are sent reminders about scheduled visits at one month, one week and one day before the scheduled date and time. We attempt to collect data in a 2-week window either side of the follow-up due date i.e., date ±2 weeks, but allow flexibility outside of this window when women are actively engaging with the research team to arrange the visit. Participants' preferred

methods of contact are documented and prioritised. Three contact attempts using different methods e.g., text, phone, email, are made to all women to confirm scheduled visits and following non-attendance to reschedule. Women who are unable to complete a visit with the researcher at 6 months are sent a link to complete the questionnaire (via Qualtrics email link). As a last resort, when all attempts to arrange a face-to-face visit at 12 (primary endpoint) and 24 months are unsuccessful, participants are asked to provide self-reported weight and complete a questionnaire (via Qualtrics email link). Participants are sent a £25 voucher at each trial timepoint (0, 6, 12 and 24 months; £100 maximum over 24 months) as a token of appreciation for the time committed to completing trial assessments. Participants lost to follow-up at one trial timepoint but who have not withdrawn, are invited to attend future visits.

To maximise data completeness, site researchers follow-up on any non-returned questionnaires and check all received data forms/e-forms to document data queries (e.g., missing data, invalid ranges etc) and any attempts made to resolve them e.g. clarify responses with the participant, using a data query log.

Data Management [19]

The hard copy of the CRF which records data at each timepoint including weight is the source data and requires sign off by the completing researcher. For questionnaires, the hard copy or electronic record is considered the source data depending on mode of completion.

The Northern Ireland Clinical Trials Unit (NICTU) are responsible for developing and managing trial databases on validated and secure systems that back-up data, maintain audit trails of data changes and only allow password-protected access to designated trained team members. Screening and CRF data are entered into trial databases by site researchers and a 10% random quality control check of data completed by a different researcher. For

CONSORT reporting, screening data includes anonymised summary information for women who do not proceed to randomisation. Paper questionnaires are scanned at site then the original posted to NICTU for data entry directly into the trial database.

Entered data is processed as per the trial specific Data Management Plan. Data queries are generated electronically by NICTU and site staff are required to respond to clarify data, provide missing information or make relevant amendments within the trial databases.

Online questionnaire completion is stored by the Qualtrics system on EU based servers compliant with General Data Protection Regulation (GDPR) and exported by the Trial Manager for merging with the NICTU questionnaire database by the Trial Statistician (CC). Following completion of data collection, data is permanently deleted from Qualtrics.

Qualitative interview transcripts are anonymised and checked for accuracy against the audio recordings, then the recordings deleted.

A minimal amount of personal information (participant's first name, mobile phone number and weeks postpartum at baseline) is entered into the randomisation system to allow delivery of text messages according to group allocation. Only designated trained team members have password-protected access to the randomisation system.

Text message engagement data is downloaded from the LSHTM system at the end of the intervention period in the form of all messages sent to and replies received from participants.

At the trial end, sites and NICTU send original source documentation (hard copy or electronic) along with consent forms and contact details for individuals who provided consent to be contacted about follow-up to this trial/future-related research/future data linkage to routinely collected health records, to QUB for archiving in accordance with the Sponsor's requirements (10 years after end of trial/publication). The CI will conduct periodic reviews (every five years) to ensure necessity of data retention. Any other documents containing

personal data are confidentially destroyed. Sites are responsible for archiving general site files accumulated during the day-to-day operation of the trial.

Data protection and confidentiality [27]

All data is collected, stored and handled in accordance with the QUB Research Management Policy (2015), GDPR and any future relevant data protection legislation and all trial team members must be compliant with these regulations. Detailed description of the trial data processing is documented in a Data Privacy Impact Assessment and in the privacy notice given to women expressing an interest in the trial.

Participants are allocated a unique 5-digit participant identifier at randomisation which is used on all data collection forms/files to allow identification of data for each participant, with the key for unlocking pseudonymised data held by site teams in password-protected files. Hard copies of forms are securely stored in locked filing cabinets in locked offices in buildings requiring keypad access and which are alarmed outside normal working hours. Consent and contact details forms, containing identifiable information, are stored separately to other data (CRFs and questionnaires) and will not be kept for longer than is required. All electronic forms or data files are held on password-protected systems that are routinely backed up and are only accessible using username, password and/or multi-factor authentication. Third-party transcription providers sign a confidentiality agreement detailing data handling requirements. Any file transfer between QUB and other parties (sites/transcription provider) is done, subject to relevant data sharing agreements, via secure and encrypted file transfer systems (e.g., QUB Dropoff). Participants will not be identifiable from any published trial report.

Data collected from women expressing an interest in trial participation but who are not recruited will be confidentially shredded/deleted.

For peer review only - http://bmjopen.bmj.com/site/about/guidelines.xhtml

Access to data [29/31c]

Access to trial records and source data can be granted to authorised Sponsor (or delegate), REC, host institution and regulatory authority representatives for trial-related monitoring, audits and inspections, as per participant consent.

Only employed trial team members have access to personal/sensitive data of participants and data is shared with those responsible for the healthcare of the participants only when health concerns are raised e.g., EPDS score of nine or greater, see [30].

The Trial Manager, Statistician and Health Economist will have access to the anonymised dataset at the trial end, to permit analysis.

The CI will manage access rights to the trial data set, in collaboration with the sponsor, and formal requests for data access need to be made in writing to the CI. The trial will comply with the good practice principles for sharing individual participant data from publicly funded trials and data sharing will be undertaken in accordance with the required regulatory requirements. Any transfer of data to other institutions will be governed by a Data Access Agreement and will be in the form of an anonymised dataset. In the event of publications arising from further data analyses, those responsible will need to provide the CI with a copy of any intended manuscript for approval prior to submission.

Statistics and analysis

Aligned with best practice and following appropriate reporting guidelines, a Statistical Analysis Plan,(79) Health Economics Analysis Plan (80, 81) and Process Evaluation Analysis Plan are written and approved by the PMT and TSC prior to analysis. Trial results

For peer review only - http://bmjopen.bmj.com/site/about/guidelines.xhtml

688 will be reported in accordance with the CONSORT guidelines, with the flow of participants
689 summarised in a CONSORT flow diagram (http://www.consort-statement.org/) and tables of
690 summary baseline characteristics presented, by total recruited sample and 12 month
691 completers.

692

693 Primary outcome analysis [20a]

694 Weight change (kg) at 12 months will be compared on an intention to treat basis (i.e., all
695 participants as randomised and regardless of whether or not they engaged with the text
696 messages), between the intervention and active control groups, adjusted for weight at
697 baseline, site, recruitment method (community vs. NHS) and ethnicity. The adjusted
698 difference in means between the groups, corresponding 95% confidence intervals (CIs) and
699 P-value will be reported. A complete-case approach will be used. Sensitivity analysis
700 assessing the impact on the primary outcome analysis will be conducted to: 1) include
701 participant self-reported weight, only where researcher-measured weight is not available;
702 and 2) use last observation carried forward, multiple imputation to impute missing outcome
703 values and delta methods to explore the impact of worse or better outcomes in the
704 individuals with missing data.

705 The independent TSC in conjunction with NIHR will decide if the trial should progress to
706 stage 3 (24 month follow-up and stakeholder engagement) based on assessment of the 12
707 month primary outcome data and any other core outcome data required to make a fully
708 informed decision at 12 months (end of intervention). If the trial proceeds, a further analysis
709 of weight change (kg) will be conducted at 24 months. Despite the sequential nature of this
710 comparison, we do not intend to change the significance level because this test will be a
711 secondary analysis.

For peer review only - http://bmjopen.bmj.com/site/about/guidelines.xhtml

All testing will be done at the two-sided 5% significance level. All analysis will be conducted using STATA software (Statcorp).

Secondary outcome analysis [20a]

Similar analyses will be conducted for secondary continuous outcomes and for secondary analyses of outcomes at other time points. Binary outcomes will be analysed using logistic regression, comparing the intervention and active control groups whilst adjusting for site, recruitment method and ethnicity. Adjusted odds ratios with 95% CIs and corresponding P-values will be reported. Secondary analyses will be considered exploratory and hence the P-values will not be altered to control for multiple testing. The focus of all secondary analyses will be on estimating the difference between-groups with 95% CIs rather than hypothesis testing.

Methods for additional analyses (e.g., subgroup analyses) [20b]

To understand any differential effects of the intervention, subgroup analyses will be conducted stratifying by pre-specified variables including site, SES, ethnicity, recruitment method, weeks postpartum (at baseline), BMI (at baseline) and parity. Interaction tests will be conducted, separately, by including interaction terms within regression models. The significance level for subgroup analyses will not be reduced but will be interpreted cautiously. As recommended, all subgroup analyses will be reported.(82)

Statistical methods to handle protocol non-adherence and missing data [20c]

The primary analysis of weight change (kg) at 12 months will be on an intention to treat basis.

Interim analyses [21b]

No interim analyses are planned.

Economic evaluation

The within trial economic evaluation builds on the methods successfully used in the pilot economic evaluation (28) including the identification, measurement and valuation of resource use and expenditure and quality of life/capability wellbeing (Table 2). The economic evaluation is conducted from a UK NHS and personal social services perspective with the addition of a societal perspective applied to capture broader impacts, consistent with the UK's NICE guidance for public health economic evaluations.(83) Direct costs to the health care system include the intervention implementation costs and any follow-up service use costs. These health and personal perspectives will be assessed to investigate a broader impact of the intervention, including direct cost to participants and indirect costs. Both costs and health outcomes will be discounted at the same annual rate of 1.5% for the reference case, as recommended by the NICE public health methods guidelines.(83) The economic analysis uses a 'multi-pronged' approach. The total and mean per-participant costs for the intervention and control groups will be calculated. A sensitivity analysis will incorporate development costs. Regression analysis, controlling for baseline differences, will be used to estimate the average cost per-participant and the average quality-adjusted life year (QALY) achieved, by group. A cost-utility analysis will report cost per QALY gained over the 24 month period using the area under the curve approach.(84) Results will be presented on the

For peer review only - http://bmjopen.bmj.com/site/about/guidelines.xhtml

cost-effectiveness plan and 95% CIs for the incremental cost-effectiveness ratio will be determined.(85) Joint uncertainty in costs and outcomes will be represented using a cost-effectiveness acceptability curve to present the probability of the SMS intervention being cost-effective for prevailing UK ceiling thresholds for costs per QALY gained.(86) The cost-effectiveness analysis will align with the primary outcome, by examining differences in weight and BMI and total costs between treatment groups, the incremental cost per weight gain averted will be calculated. The mean costs and effects for each trial arm will be calculated and presented along with the incremental costs and effects between arms (including 95% CIs).

The within trial economic analysis will be carried out for the 24 months follow-up and will form the primary analysis. However, this short time horizon may be insufficient to capture the total costs and benefits related to the intervention. If differences in quality of life or capability wellbeing are identified, the results will be extrapolated to a lifetime horizon using modelling methods.

Monitoring and auditing [23]

As per contractual agreements and participant consent, direct access to trial records and source documents will be granted to the Sponsor or regulatory agencies by all research staff and trial sites, for the purposes of trial related monitoring, audits and inspections. The trial is monitored and audited in accordance with the Sponsor's policy, which is consistent with the UK Policy Framework for Health and Social Care Research, and the TSC informed of the main findings of any monitoring, audit or inspection.

Trial Steering Committee (TSC) and Data Monitoring [5d/21a]

For peer review only - http://bmjopen.bmj.com/site/about/guidelines.xhtml

The independent TSC meet face-to-face or online at least annually, as required, to provide trial oversight on behalf of the sponsor and funder. The TSC determine trial progression at the recruitment mid-point (see Figure 1 and [15]) and primary outcome stage. As this RCT involves a low-risk behaviour change lifestyle intervention which is unlikely to raise concerns for participant safety (no evidence of harm was reported in the pilot study or in any other text message behaviour change studies), the sponsor and funder agreed that a Data Monitoring Committee would not be convened [21a]. Participant safety is a standing agenda item at each meeting, see [22].

Project Management Team (PMT) [5d]

The PMT is the key decision-making group consisting of the co-applicants, Trial Manager and PPI representative. The PMT meet online every 2-4 months to oversee the trial design, management and conduct, including monitoring participant safety, see [22].

Trial coordinating centre [5d]

The trial is coordinated by the Chief Investigator (MMcK) and the Trial Manager (DG) in QUB. Weekly meetings between the CI and Trial Manager and regular meetings with site teams are held, to discuss trial activities across the five trial sites. Site PIs are responsible for managing staff and overseeing participant recruitment and follow-up at their sites.

Adverse event reporting and harms [22]

Serious adverse events (SAEs) are recorded by researchers from the time of the participant's consent into the trial until one month after the end of the 12 month intervention

For peer review only - http://bmjopen.bmj.com/site/about/guidelines.xhtml

period and reported in line with GCP guidelines and the Sponsor's adverse event reporting procedures. Site PIs review SAEs within 24 hours to determine severity, relatedness and expectedness. The CI (or delegate) is informed of any event evaluated as being severe, related and unexpected, to conduct a second review. Onward reporting of any SAEs categorised as related and expected by both the PI and CI is actioned by the CI or Trial Manager to the Sponsor and REC within 24 hours and 15 days respectively. All SAEs are summarised and included in annual reports to the REC and reported during meetings of the PMT and TSC.

Ancillary and post-trial care [30]

This is deemed a low-risk public health trial and no evidence of harm has been reported in text message studies for other health behaviours or in our pilot RCT, so no post-trial care is delivered. All women are given a signposting 'Useful Contacts' leaflet during trial visits advising them on relevant agencies and organisations that they might contact for support on a variety of issues e.g., bereavement. We monitor any emerging safety issues or unintended consequences and any participants experiencing such issues will be referred to their GP for appropriate treatment. This includes the onward reporting of EPDS scores of nine or above (Table 2). The EPDS is a screening questionnaire used to detect postnatal depressive symptoms and the scale guidance indicates that women with scores meeting this threshold would benefit from further clinical assessment. We calculate scores within one week of receiving the completed EPDS at each follow-up timepoint and letters are sent to the GP and the participant to inform of scores ≥9, as per PIS and consent processes.

Personal and Public Involvement (PPI)* Statement

(Note: In NI, PPI means Personal and Public Involvement due to integrated Health and Social Care systems. The rest of the UK use 'Patient and Public Involvement' also known as 'Patient and Public Involvement and Engagement')*

PPI was integral to the pilot study with guidance received on the development of the text message libraries and all trial methods and materials, as previously reported.(28) The design of the present trial mirrors the methods used in the pilot trial. We recruited PPI representatives from Scotland, London and Bradford to conduct a period of trial adaptation (Figure 1) whereby the library of intervention text messages and trial recruitment materials underwent further PPI to ensure content was acceptable and culturally relevant for women from a range of backgrounds and ethnicities across the UK.

We have PPI representatives on our PMT and independent TSC to contribute to decision making throughout the trial and we seek their advice on an ad-hoc basis, as matters arise. PPI representatives will help shape the dissemination plan for trial results and will review dissemination materials to ensure communication of findings in an appropriate manner.

PPI work is led by the trial team and conducted and reimbursed according to NIHR guidance for involving the public in research.(87)

Protocol amendments [25]

The CI, in consultation with the PMT and Sponsor, is responsible for the decision to amend the protocol and, in turn, for communicating substantive changes to relevant stakeholders. Substantial and non-substantial amendments are reviewed by the sponsor to determine the appropriate amendment category then submitted to the approving REC via the IRAS system for approval prior to implementation, and subsequently disseminated to participating centres (R&D offices, regulatory agencies). Version control and details of amendment history are

For peer review only - http://bmjopen.bmj.com/site/about/guidelines.xhtml

853 clearly documented in the protocol and are disseminated to the trial sites, PMT and TSC
854 members, funder and trial registries.

855 The current protocol is 3.0 (10/05/2023). Amendments to the protocol incorporated: 1) the
856 added mental health objectives resulting from the additional NIHR funding; 2) an increase in
857 trial sample size (from 850 to 888 women) to allow for higher estimated pregnancy exclusion
858 rates in more ethnically diverse populations than were originally accounted for; 3) to include
859 processes to collect participant self-reported weight at 12 and 24 months only as a last
860 resort in cases where it is not possible to obtain researcher-measured weight; and 4) to offer
861 a booklet summarising the intervention content to the active control group after completion of
862 data collection at 24 months.

863

864 Protocol training and deviations

865 All trial staff undergo training on the protocol, trial processes and standard operating
866 procedures (SOPs) for which they are responsible (detailed on the site delegation log). The
867 Trial Manager monitors trial activities across sites to ensure adherence and consistency to
868 the protocol. There are no planned protocol deviations. If a deviation occurs, it is
869 documented and reported immediately to the CI and Sponsor, including corrective and
870 preventative actions. Frequent recurrence of protocol deviations is unacceptable, requires
871 immediate action and could be classified as a serious breach i.e., one which is likely to
872 significantly affect the safety or physical or mental integrity of trial participants and/or the
873 scientific value of the trial. Any breach meeting this definition is immediately reported to the
874 Sponsor, as per the Sponsor's SOP on 'Matters of non-compliance with study protocol'.

875

876 Peer review

For peer review only - http://bmjopen.bmj.com/site/about/guidelines.xhtml

This trial has undergone independent, expert peer-review as part of the NIHR PHR funding process. It has also been reviewed and approved by the QUB Research Governance, Ethics and Integrity Manager, on behalf of the Sponsor.

Ethics and dissemination

Ethical and Regulatory considerations [24]

The trial is conducted in accordance with the recommendations for physicians involved in research on human participants adopted by the 18th World Medical Assembly, Helsinki 1964, and later revisions, the UK Policy Framework for Health and Social Care, relevant UK data protection legislation and the principles of GCP. QUB acts as sponsor for the trial and has in place comprehensive SOPs for the approval and monitoring of research. This protocol and other relevant trial documents were reviewed and approved by the West of Scotland Research Ethics Service REC 4 22/WS/0003. Correspondence with the REC is retained in the Trial Master File. An annual progress report is submitted to REC until the trial end. The CI will report to REC when the trial ends (or is terminated prematurely) and a final report with trial results submitted within one year of this.

Appropriate approvals from participating organisations e.g., NHS R&D approvals are obtained before recruiting via applicable routes.

Dissemination [31a]

Dissemination plans informed by PPI and participant and stakeholder engagement will be developed and reviewed annually. A range of outputs including NIHR final report, peer-reviewed open access publications, conference presentations, policy briefings, non-academic research summaries and a draft scaling-up plan, are anticipated to be disseminated relevant for different audiences, including trial participants, postpartum women

For peer review only - http://bmjopen.bmj.com/site/about/guidelines.xhtml

throughout the UK, healthcare professionals, service commissioners, academics, the Government and public health bodies.

References

1. Dodd JM, Deussen AR, O'Brien CM, Schoenaker D, Poprzeczny A, Gordon A, et al. Targeting the postpartum period to promote weight loss: a systematic review and meta-analysis. Nutr Rev. 2018;76(8):639-54.

2. Chan A-W, Tetzlaff JM, Altman DG, Laupacis A, Gøtzsche PC, Krleža-Jerić K, et al. SPIRIT 2013 Statement: Defining standard protocol items for clinical trials. . Ann Intern Med. 2013;158:200-7.

3. Scott-Pillai R, Spence D, Cardwell CR, Hunter A, Holmes VA. The impact of body mass index on maternal and neonatal outcomes: a retrospective study in a UK obstetric population, 2004-2011. Bjog. 2013;120(8):932-9.

4. Marchi J, Berg M, Dencker A, Olander EK, Begley C. Risks associated with obesity in pregnancy, for the mother and baby: a systematic review of reviews. Obes Rev. 2015;16(8):621-38.

5. Bhattacharya S, Campbell DM, Liston WA, Bhattacharya S. Effect of Body Mass Index on pregnancy outcomes in nulliparous women delivering singleton babies. BMC Public Health. 2007;7:168.

6. Aune D, Saugstad OD, Henriksen T, Tonstad S. Maternal body mass index and the risk of fetal death, stillbirth, and infant death: a systematic review and meta-analysis. Jama. 2014;311(15):1536-46.

7. Crozier SR, Inskip HM, Godfrey KM, Cooper C, Harvey NC, Cole ZA, et al. Weight gain in pregnancy and childhood body composition: findings from the Southampton Women's Survey123. The American Journal of Clinical Nutrition. 2010;91(6):1745-51.

8. Crosby DA, Collins M, O'Higgins A, Mullaney L, Farah N, Turner MJ. Interpregnancy changes in maternal weight and body mass index. Am J Perinatol. 2015;30(2):199-204.

9. McKinley MC, Allen-Walker V, McGirr C, Rooney C, Woodside JV. Weight loss after pregnancy: challenges and opportunities. Nutr Res Rev. 2018;31(2):225-38.

10. Lim S, O'Reilly S, Behrens H, Skinner T, Ellis I, Dunbar JA. Effective strategies for weight loss in post-partum women: a systematic review and meta-analysis. Obes Rev. 2015;16(11):972-87.

11. Nascimento SL, Pudwell J, Surita FG, Adamo KB, Smith GN. The effect of physical exercise strategies on weight loss in postpartum women: a systematic review and meta-analysis. Int J Obes (Lond). 2014;38(5):626-35.

12. van der Pligt P, Willcox J, Hesketh KD, Ball K, Wilkinson S, Crawford D, et al. Systematic review of lifestyle interventions to limit postpartum weight retention: implications for future opportunities to prevent maternal overweight and obesity following childbirth. Obes Rev. 2013;14(10):792-805.

13. National Institute for Health and Care Excellence (NICE). Weight Management Before, During and After Pregnancy: NICE Public Health Guidance 27. 2010 [Available from: https://www.nice.org.uk/guidance/ph27.

14. Amorim Adegboye AR, Linne YM. Diet or exercise, or both, for weight reduction in women after childbirth. Cochrane Database Syst Rev. 2013;2013(7):Cd005627.

15. Farpour-Lambert NJ, Ells LJ, Martinez de Tejada B, Scott C. Obesity and Weight Gain in Pregnancy and Postpartum: an Evidence Review of Lifestyle Interventions to Inform Maternal and Child Health Policies. Front Endocrinol (Lausanne). 2018;9:546.

For peer review only

For peer review only - http://bmjopen.bmj.com/site/about/guidelines.xhtml

16. Joshi PP, Quintiliani LM, McCarthy AC, Gilmore A, Mahesri M, Sullivan LM, et al. A Randomized Controlled Feasibility Trial in Behavioral Weight Management for Underserved Postpartum African American Women: The RENEW Study. Prev Chronic Dis. 2018;15:E77.
17. Wright C, Mogul M, Acevedo G, Aysola J, Momplaisir F, Schwartz S, et al. Preparing for a trial to test a postpartum weight retention intervention among low income women: feasibility of a protocol in a community-based organization. BMC Womens Health. 2018;18(1):27.
18. Haste A, Adamson AJ, McColl E, Araujo-Soares V, Bell R. Problems recruiting and retaining postnatal women to a pilot randomised controlled trial of a web-delivered weight loss intervention. BMC Res Notes. 2018;11(1):203.
19. Montgomery KS, Bushee TD, Phillips JD, Kirkpatrick T, Catledge C, Braveboy K, et al. Women's challenges with postpartum weight loss. Matern Child Health J. 2011;15(8):1176-84.
20. Neiterman E, Fox B. Controlling the unruly maternal body: Losing and gaining control over the body during pregnancy and the postpartum period. Soc Sci Med. 2017;174:142-8.
21. Christenson A, Johansson E, Reynisdottir S, Torgerson J, Hemmingsson E. Women's Perceived Reasons for Their Excessive Postpartum Weight Retention: A Qualitative Interview Study. PLoS One. 2016;11(12):e0167731.
22. Saligheh M, McNamara B, Rooney R. Perceived barriers and enablers of physical activity in postpartum women: a qualitative approach. BMC Pregnancy and Childbirth. 2016;16(1):131.
23. Devine CM, Bove CF, Olson CM. Continuity and change in women's weight orientations and lifestyle practices through pregnancy and the postpartum period: the influence of life course trajectories and transitional events. Soc Sci Med. 2000;50(4):567-82.
24. O'Toole ML, Sawicki MA, Artal R. Structured diet and physical activity prevent postpartum weight retention. J Womens Health (Larchmt). 2003;12(10):991-8.
25. Macleod M, Craigie AM, Barton KL, Treweek S, Anderson AS. Recruiting and retaining postpartum women from areas of social disadvantage in a weight-loss trial--an assessment of strategies employed in the WeighWell feasibility study. Matern Child Nutr. 2013;9(3):322-31.
26. Taylor C, Bhavnani V, Zasada M, Ussher M, Bick D. Barriers and facilitators to uptake and retention of inner-city ethnically diverse women in a postnatal weight management intervention: a mixed-methods process evaluation within a feasibility trial in England. BMJ Open. 2020;10(7):e034747.
27. Østbye T, Krause KM, Lovelady CA, Morey MC, Bastian LA, Peterson BL, et al. Active Mothers Postpartum: a randomized controlled weight-loss intervention trial. Am J Prev Med. 2009;37(3):173-80.
28. McGirr C, Rooney C, Gallagher D, Dombrowski SU, Anderson AS, Cardwell CR, et al. Text messaging to help women with overweight or obesity lose weight after childbirth: the intervention adaptation and SMS feasibility RCT. NIHR Journals Library: Public Health Research. 2020;8(4).
29. Herring SJ, Cruice JF, Bennett GG, Darden N, Wallen JJ, Rose MZ, et al. Intervening during and after pregnancy to prevent weight retention among African American women. Prev Med Rep. 2017;7:119-23.
30. Waring ME, Moore Simas TA, Oleski J, Xiao RS, Mulcahy JA, May CN, et al. Feasibility and Acceptability of Delivering a Postpartum Weight Loss Intervention via Facebook: A Pilot Study. J Nutr Educ Behav. 2018;50(1):70-4.e1.
31. Willcox JC, Dobson R, Whittaker R. Old-Fashioned Technology in the Era of "Bling": Is There a Future for Text Messaging in Health Care? J Med Internet Res. 2019;21(12):e16630.
32. Job JR, Fjeldsoe BS, Eakin EG, Reeves MM. Effectiveness of extended contact interventions for weight management delivered via text messaging: a systematic review and meta-analysis. Obesity Reviews. 2018;19(4):538-49.
33. Samuel Holmes W, Anne Moorhead S, Coates VE, Bond RR, Zheng H. Impact of digital technologies for communicating messages on weight loss maintenance: a systematic literature review. European Journal of Public Health. 2018;29(2):320-8.

For peer review only - http://bmjopen.bmj.com/site/about/guidelines.xhtml

34. Schippers M, Adam PC, Smolenski DJ, Wong HT, de Wit JB. A meta-analysis of overall effects of weight loss interventions delivered via mobile phones and effect size differences according to delivery mode, personal contact, and intervention intensity and duration. Obes Rev. 2017;18(4):450-9.

35. Siopis G, Chey T, Allman-Farinelli M. A systematic review and meta-analysis of interventions for weight management using text messaging. J Hum Nutr Diet. 2015;28 Suppl 2:1-15.

36. Skinner R, Gonet V, Currie S, Hoddinott P, Dombrowski SU. A systematic review with meta-analyses of text message-delivered behaviour change interventions for weight loss and weight loss maintenance. Obes Rev. 2020;21(6):e12999.

37. Guerriero C, Cairns J, Roberts I, Rodgers A, Whittaker R, Free C. The cost-effectiveness of smoking cessation support delivered by mobile phone text messaging: Txt2stop. Eur J Health Econ. 2013;14(5):789-97.

38. Free C, Knight R, Robertson S, Whittaker R, Edwards P, Zhou W, et al. Smoking cessation support delivered via mobile phone text messaging (txt2stop): a single-blind, randomised trial. The Lancet. 2011;378(9785):49-55.

39. Morgan KL, Rahman MA, Macey S, Atkinson MD, Hill RA, Khanom A, et al. Obesity in pregnancy: a retrospective prevalence-based study on health service utilisation and costs on the NHS. BMJ Open. 2014;4(2):e003983.

40. Rawdin AC, Duenas A, Chilcott JB. The cost-effectiveness of weight management programmes in a postnatal population. Public Health. 2014;128(9):804-10.

41. Denison FC, Aedla NR, Keag O, Hor K, Reynolds RM, Milne A, et al. Care of women with obesity in pregnancy: Green-top guideline no.72. BJOG. 2018.

42. Phelan S. Pregnancy: a "teachable moment" for weight control and obesity prevention. Am J Obstet Gynecol. 2010;202(2):135.e1-8.

43. Dinsdale S, Branch K, Cook L, Shucksmith J. “As soon as you’ve had the baby that’s it…” a qualitative study of 24 postnatal women on their experience of maternal obesity care pathways. BMC Public Health. 2016;16(1):625.

44. Lipsky LM, Strawderman MS, Olson CM. Maternal weight change between 1 and 2 years postpartum: the importance of 1 year weight retention. Obesity (Silver Spring). 2012;20(7):1496-502.

45. Østbye T, Peterson BL, Krause KM, Swamy GK, Lovelady CA. Predictors of postpartum weight change among overweight and obese women: results from the Active Mothers Postpartum study. J Womens Health (Larchmt). 2012;21(2):215-22.

46. Cobiac L, Vos T, Veerman L. Cost-effectiveness of Weight Watchers and the Lighten Up to a Healthy Lifestyle program. Aust N Z J Public Health. 2010;34(3):240-7.

47. Hoddinott P, Allan K, Avenell A, Britten J. Group interventions to improve health outcomes: a framework for their design and delivery. BMC Public Health. 2010;10(1):800.

48. White M, Adams J, Heywood P. How and why do interventions that increase health overall widen inequalities within populations? In: Babones SJ, editor. Social inequality and public health: Policy Press; 2009. p. 0.

49. Shaw R, Bosworth H. Short message service (SMS) text messaging as an intervention medium for weight loss: A literature review. Health Informatics J. 2012;18(4):235-50.

50. Sherifali D, Nerenberg KA, Wilson S, Semeniuk K, Ali MU, Redman LM, et al. The Effectiveness of eHealth Technologies on Weight Management in Pregnant and Postpartum Women: Systematic Review and Meta-Analysis. J Med Internet Res. 2017;19(10):e337.

51. Scottish Government. Scotland's Digital Future. A Strategy for Scotland. Edinburgh: Scottish Government; 2011.

52. Hoffmann TC, Glasziou PP, Boutron I, Milne R, Perera R, Moher D, et al. Better reporting of interventions: template for intervention description and replication (TIDieR) checklist and guide. BMJ : British Medical Journal. 2014;348:g1687.

For peer review only

For peer review only - http://bmjopen.bmj.com/site/about/guidelines.xhtml

53. Kwasnicka D, Dombrowski SU, White M, Sniehotta F. Theoretical explanations for maintenance of behaviour change: a systematic review of behaviour theories. Health Psychology Review. 2016;10(3):277-96.
54. Catenacci VA, Odgen L, Phelan S, Thomas JG, Hill J, Wing RR, et al. Dietary habits and weight maintenance success in high versus low exercisers in the National Weight Control Registry. J Phys Act Health. 2014;11(8):1540-8.
55. Marinilli Pinto A, Gorin AA, Raynor HA, Tate DF, Fava JL, Wing RR. Successful weight-loss maintenance in relation to method of weight loss. Obesity (Silver Spring). 2008;16(11):2456-61.
56. Tang J, Abraham C, Greaves C, Yates T. Self-directed interventions to promote weight loss: a systematic review of reviews. J Med Internet Res. 2014;16(2):e58.
57. Phelan S, Hagobian T, Brannen A, Hatley KE, Schaffner A, Muñoz-Christian K, et al. Effect of an Internet-Based Program on Weight Loss for Low-Income Postpartum Women: A Randomized Clinical Trial. Jama. 2017;317(23):2381-91.
58. Firetext. Firetext SMS provider 2023 [Available from: https://www.firetext.co.uk/.
59. National Health Service. Better Health: Start for Life 2023 [Available from: https://www.nhs.uk/start-for-life/.
60. Brooks R. EuroQol: the current state of play. Health Policy. 1996;37(1):53-72.
61. Al-Janabi H, Flynn TN, Coast J. Development of a self-report measure of capability wellbeing for adults: the ICECAP-A. Qual Life Res. 2012;21(1):167-76.
62. Wright JL, Scott JA. The Fat and Fibre Barometer, a short food behaviour questionnaire: reliability, relative validity and utility. Aus J Nutr Dietet. 2000;57:33-9.
63. Booth M. Assessment of physical activity: an international perspective. Res Q Exerc Sport. 2000;71 Suppl 2:114-20.
64. McAndrew F, Thompson J, Fellows L, Large A, Speed M, Renfrew MJ. Infant Feeding Survey 2010.: Health and Social Care Information Centre; 2012.
65. Cox JL, Holden JM, Sagovsky R. Detection of postnatal depression. Development of the 10-item Edinburgh Postnatal Depression Scale. Br J Psychiatry. 1987;150:782-6.
66. Spitzer RL, Kroenke K, Williams JB, Löwe B. A brief measure for assessing generalized anxiety disorder: the GAD-7. Arch Intern Med. 2006;166(10):1092-7.
67. Born in Bradford. Me and my Baby Measure 2023 [Available from: https://borninbradford.nhs.uk/gallery/me-and-my-baby-measure/.
68. Oates J, Gervai J. Mothers' Perceptions of Their Infants. Journal of Prenatal and Perinatal Psychology and Health. 2019;33(4):282-300.
69. Buysse DJ, Reynolds CF, 3rd, Monk TH, Berman SR, Kupfer DJ. The Pittsburgh Sleep Quality Index: a new instrument for psychiatric practice and research. Psychiatry Res. 1989;28(2):193-213.
70. Sniehotta FF, Schwarzer R, Scholz U, Schüz B. Action planning and coping planning for long-term lifestyle change: theory and assessment. European Journal of Social Psychology. 2005;35(4):565-76.
71. Schwarzer R, Renner B. Social-cognitive predictors of health behavior: action self-efficacy and coping self-efficacy. Health Psychol. 2000;19(5):487-95.
72. Gardner B, Abraham C, Lally P, de Bruijn G-J. Towards parsimony in habit measurement: Testing the convergent and predictive validity of an automaticity subscale of the Self-Report Habit Index. International Journal of Behavioral Nutrition and Physical Activity. 2012;9(1):102.
73. Kliemann N, Beeken RJ, Wardle J, Johnson F. Development and validation of the Self-Regulation of Eating Behaviour Questionnaire for adults. International Journal of Behavioral Nutrition and Physical Activity. 2016;13(1):87.
74. Meyer AH, Weissen-Schelling S, Munsch S, Margraf J. Initial development and reliability of a motivation for weight loss scale. Obes Facts. 2010;3(3):205-11.
75. Sallis JF, Grossman RM, Pinski RB, Patterson TL, Nader PR. The development of scales to measure social support for diet and exercise behaviors. Prev Med. 1987;16(6):825-36.
76. Rosenberg M. Conceiving the self. New York: Basic Books; 1979.

For peer review only

For peer review only - http://bmjopen.bmj.com/site/about/guidelines.xhtml

77. Born in Bradford. Born in Bradford's Better Start. 2023 [Available from: https://borninbradford.nhs.uk/what-we-do/cohort-studies/better-start/.

78. Avery KNL, Williamson PR, Gamble C, O'Connell Francischetto E, Metcalfe C, Davidson P, et al. Informing efficient randomised controlled trials: exploration of challenges in developing progression criteria for internal pilot studies. BMJ Open. 2017;7(2):e013537.

79. Gamble C, Krishan A, Stocken D, Lewis S, Juszczak E, Doré C, et al. Guidelines for the Content of Statistical Analysis Plans in Clinical Trials. JAMA. 2017;318(23):2337-43.

80. Thorn JC, Davies CF, Brookes ST, Noble SM, Dritsaki M, Gray E, et al. Content of Health Economics Analysis Plans (HEAPs) for Trial-Based Economic Evaluations: Expert Delphi Consensus Survey. Value Health. 2021;24(4):539-47.

81. Husereau D, Drummond M, Augustovski F, de Bekker-Grob E, Briggs AH, Carswell C, et al. Consolidated Health Economic Evaluation Reporting Standards (CHEERS) 2022 Explanation and Elaboration: A Report of the ISPOR CHEERS II Good Practices Task Force. Value in Health. 2022;25(1):10-31.

82. van Hoorn R, Tummers M, Booth A, Gerhardus A, Rehfuess E, Hind D, et al. The development of CHAMP: a checklist for the appraisal of moderators and predictors. BMC Medical Research Methodology. 2017;17(1):173.

83. National Institute for Health and Care Excellence (NICE). Methods for the development of NICE public health guidance (third edition): process and methods.; 2012.

84. Manca A, Hawkins N, Sculpher MJ. Estimating mean QALYs in trial-based cost-effectiveness analysis: the importance of controlling for baseline utility. Health Econ. 2005;14(5):487-96.

85. Drummond MF, Sculpher MJ, Claxton K, Stoddart GL, Torrance GW. Methods for the Economic Evaluation of Health Care Programmes (fourth edition). Oxford: Oxford University Press; 2015.

86. Fenwick E, Marshall DA, Levy AR, Nichol G. Using and interpreting cost-effectiveness acceptability curves: an example using data from a trial of management strategies for atrial fibrillation. BMC Health Services Research. 2006;6(1):52.

87. National Institute for Health and Care Research (NIHR). Payments guidance for members of the public, Version 1.4. 2023 [Available from: https://www.nihr.ac.uk/documents/payment-guidance-for-members-of-the-public-considering-involvement-in-research/27372.

Authors' contributions [31b]

MCM led the funding proposal and protocol development with support from all co-authors (DG, ES, ASA, SB, CC, EC, SUD, CF, SH, PH, FK, CMcD, EM, JVW) who provided expert input on trial design and intervention content. MCM, ES and DG co-ordinated the ethical approval. DG drafted the protocol manuscript. All authors (DG, ES, ASA, SB, CC, EC, SUD, CF, SH, PH, FK, CMcD, EM, JVW, MCM) contributed to the revision of the manuscript and have read and approved the final version.

For peer review only - http://bmjopen.bmj.com/site/about/guidelines.xhtml

Funding Statement [4]

This trial is supported by the NIHR Public Health Research (PHR) Programme (NIHR131509) and intervention costs provided by the Northern Ireland Public Health Agency.

Competing interests' statement [28]

The CI, PIs, co-investigators, trial staff and TSC are required to disclose information on competing interests that might influence the trial integrity, such as commercial ownership interests. A record of competing interests is retained by the Trial Manager for reporting in all trial publications.

<u>Acknowledgements</u>

For the purpose of open access, the author has applied a Creative Commons Attribution (CC BY) licence to any Author Accepted Manuscript version arising from this submission.

Word Count: 9118

For peer review only - http://bmjopen.bmj.com/site/about/guidelines.xhtml

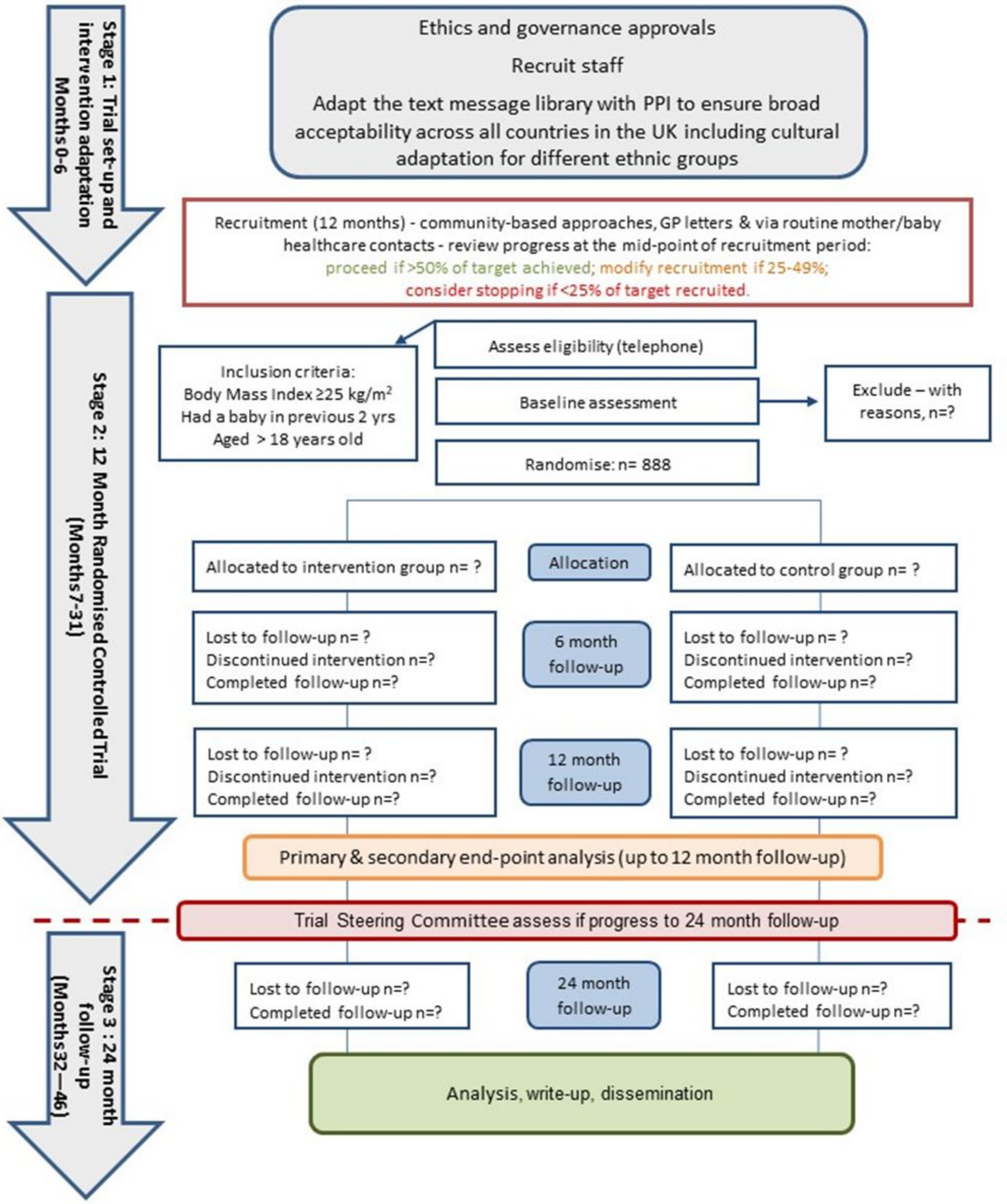
Stage 1: Trial set-up and intervention adaptation Months 0-6
Ethics and governance approvals
Recruit staff
Adapt the text message library with PPI to ensure broad acceptability across all countries in the UK including cultural adaptation for different ethnic groups
Recruitment (12 months) - community-based approaches, GP letters & via routine mother/baby healthcare contacts - review progress at the mid-point of recruitment period:
proceed if >50% of target achieved; modify recruitment if 25-49%;
consider stopping if <25% of target recruited.
Stage 2: 12 Month Randomised Controlled Trial (Months 7-31)
Assess eligibility (telephone)
Inclusion criteria:
Body Mass Index ≥25 kg/m²
Had a baby in previous 2 yrs
Aged > 18 years old
Baseline assessment
Exclude – with reasons, n=?
Randomise: n= 888
Allocated to intervention group n= ?
Allocation
Allocated to control group n= ?
Lost to follow-up n= ?
Discontinued intervention n=?
Completed follow-up n=?
6 month follow-up
Lost to follow-up n= ?
Discontinued intervention n=?
Completed follow-up n=?
Lost to follow-up n= ?
Discontinued intervention n=?
Completed follow-up n=?
12 month follow-up
Lost to follow-up n= ?
Discontinued intervention n=?
Completed follow-up n=?
Primary & secondary end-point analysis (up to 12 month follow-up)
Trial Steering Committee assess if progress to 24 month follow-up
Stage 3 : 24 month follow-up (Months 32—46)
Lost to follow-up n=?
Completed follow-up n=?
24 month follow-up
Lost to follow-up n=?
Completed follow-up n=?
Analysis, write-up, dissemination

Supplemental appendix 1- SPIRIT checklist (2)

SPIRIT 2013 Checklist: Recommended items to address in a clinical trial protocol and related documents*

Section/item	Item No	Description	Addressed on page number
**Administrative information**			
Title	1	Descriptive title identifying the study design, population, interventions, and, if applicable, trial acronym	4
Trial registration	2a	Trial identifier and registry name. If not yet registered, name of intended registry	2
	2b	All items from the World Health Organization Trial Registration Data Set	Supplemental appendix 5
Protocol version	3	Date and version identifier	4
Funding	4	Sources and types of financial, material, and other support	50
Roles and responsibilities	5a	Names, affiliations, and roles of protocol contributors	4-7, 49-50
	5b	Name and contact information for the trial sponsor	7
	5c	Role of study sponsor and funders, if any, in study design; collection, management, analysis, and interpretation of data; writing of the report; and the decision to submit the report for publication, including whether they will have ultimate authority over any of these activities	7
	5d	Composition, roles, and responsibilities of the coordinating centre, steering committee, endpoint adjudication committee, data management team, and other individuals or groups overseeing the trial, if applicable (see Item 21a for data monitoring committee)	40
**Introduction**			
Background and rationale	6a	Description of research question and justification for undertaking the trial, including summary of relevant studies (published and unpublished) examining benefits and harms for each intervention	8-12
	6b	Explanation for choice of comparators	12-13
Objectives	7	Specific objectives or hypotheses	13-14
Trial design	8	Description of trial design including type of trial (eg, parallel group, crossover, factorial, single group), allocation ratio, and framework (eg, superiority, equivalence, noninferiority, exploratory)	14

For peer review only

For peer review only - http://bmjopen.bmj.com/site/about/guidelines.xhtml

Methods: Participants, interventions, and outcomes

Study setting	9	Description of study settings (eg, community clinic, academic hospital) and list of countries where data will be collected. Reference to where list of study sites can be obtained	14
Eligibility criteria	10	Inclusion and exclusion criteria for participants. If applicable, eligibility criteria for study centres and individuals who will perform the interventions (eg, surgeons, psychotherapists)	14-15, 26-27
Interventions	11a	Interventions for each group with sufficient detail to allow replication, including how and when they will be administered	15-19
	11b	Criteria for discontinuing or modifying allocated interventions for a given trial participant (eg, drug dose change in response to harms, participant request, or improving/worsening disease)	19
	11c	Strategies to improve adherence to intervention protocols, and any procedures for monitoring adherence (eg, drug tablet return, laboratory tests)	19
	11d	Relevant concomitant care and interventions that are permitted or prohibited during the trial	19-20
Outcomes	12	Primary, secondary, and other outcomes, including the specific measurement variable (eg, systolic blood pressure), analysis metric (eg, change from baseline, final value, time to event), method of aggregation (eg, median, proportion), and time point for each outcome. Explanation of the clinical relevance of chosen efficacy and harm outcomes is strongly recommended	20-23
Participant timeline	13	Time schedule of enrolment, interventions (including any run-ins and washouts), assessments, and visits for participants. A schematic diagram is highly recommended (see Figure)	23-24
Sample size	14	Estimated number of participants needed to achieve study objectives and how it was determined, including clinical and statistical assumptions supporting any sample size calculations	25-26
Recruitment	15	Strategies for achieving adequate participant enrolment to reach target sample size	26

Methods: Assignment of interventions (for controlled trials)

Allocation:

For peer review only - http://bmjopen.bmj.com/site/about/guidelines.xhtml

Sequence generation	16a	Method of generating the allocation sequence (eg, computer-generated random numbers), and list of any factors for stratification. To reduce predictability of a random sequence, details of any planned restriction (eg, blocking) should be provided in a separate document that is unavailable to those who enrol participants or assign interventions	28-29
Allocation concealment mechanism	16b	Mechanism of implementing the allocation sequence (eg, central telephone; sequentially numbered, opaque, sealed envelopes), describing any steps to conceal the sequence until interventions are assigned	29
Implementation	16c	Who will generate the allocation sequence, who will enrol participants, and who will assign participants to interventions	29
Blinding (masking)	17a	Who will be blinded after assignment to interventions (eg, trial participants, care providers, outcome assessors, data analysts), and how	29-30
	17b	If blinded, circumstances under which unblinding is permissible, and procedure for revealing a participant's allocated intervention during the trial	30
**Methods: Data collection, management, and analysis**			
Data collection methods	18a	Plans for assessment and collection of outcome, baseline, and other trial data, including any related processes to promote data quality (eg, duplicate measurements, training of assessors) and a description of study instruments (eg, questionnaires, laboratory tests) along with their reliability and validity, if known. Reference to where data collection forms can be found, if not in the protocol	30-31
	18b	Plans to promote participant retention and complete follow-up, including list of any outcome data to be collected for participants who discontinue or deviate from intervention protocols	31-32
Data management	19	Plans for data entry, coding, security, and storage, including any related processes to promote data quality (eg, double data entry; range checks for data values). Reference to where details of data management procedures can be found, if not in the protocol	32-34
Statistical methods	20a	Statistical methods for analysing primary and secondary outcomes. Reference to where other details of the statistical analysis plan can be found, if not in the protocol	36-37
	20b	Methods for any additional analyses (eg, subgroup and adjusted analyses)	37-38

For peer review only - http://bmjopen.bmj.com/site/about/guidelines.xhtml

	20c	Definition of analysis population relating to protocol non-adherence (eg, as randomised analysis), and any statistical methods to handle missing data (eg, multiple imputation)	38
**Methods: Monitoring**			
Data monitoring	21a	Composition of data monitoring committee (DMC); summary of its role and reporting structure; statement of whether it is independent from the sponsor and competing interests; and reference to where further details about its charter can be found, if not in the protocol. Alternatively, an explanation of why a DMC is not needed	40
	21b	Description of any interim analyses and stopping guidelines, including who will have access to these interim results and make the final decision to terminate the trial	38
Harms	22	Plans for collecting, assessing, reporting, and managing solicited and spontaneously reported adverse events and other unintended effects of trial interventions or trial conduct	41
Auditing	23	Frequency and procedures for auditing trial conduct, if any, and whether the process will be independent from investigators and the sponsor	39-40
**Ethics and dissemination**			
Research ethics approval	24	Plans for seeking research ethics committee/institutional review board (REC/IRB) approval	2, 44
Protocol amendments	25	Plans for communicating important protocol modifications (eg, changes to eligibility criteria, outcomes, analyses) to relevant parties (eg, investigators, REC/IRBs, trial participants, trial registries, journals, regulators)	42-44
Consent or assent	26a	Who will obtain informed consent or assent from potential trial participants or authorised surrogates, and how (see Item 32)	27-28
	26b	Additional consent provisions for collection and use of participant data and biological specimens in ancillary studies, if applicable	28
Confidentiality	27	How personal information about potential and enrolled participants will be collected, shared, and maintained in order to protect confidentiality before, during, and after the trial	34-35
Declaration of interests	28	Financial and other competing interests for principal investigators for the overall trial and each study site	50
Access to data	29	Statement of who will have access to the final trial dataset, and disclosure of contractual agreements that limit such access for investigators	35

For peer review only - http://bmjopen.bmj.com/site/about/guidelines.xhtml

Ancillary and post-trial care	30	Provisions, if any, for ancillary and post-trial care, and for compensation to those who suffer harm from trial participation	41-42
Dissemination policy	31a	Plans for investigators and sponsor to communicate trial results to participants, healthcare professionals, the public, and other relevant groups (eg, via publication, reporting in results databases, or other data sharing arrangements), including any publication restrictions	44-45
	31b	Authorship eligibility guidelines and any intended use of professional writers	49-50
	31c	Plans, if any, for granting public access to the full protocol, participant-level dataset, and statistical code	35
**Appendices**			
Informed consent materials	32	Model consent form and other related documentation given to participants and authorised surrogates	Supplemental appendix 4
Biological specimens	33	Plans for collection, laboratory evaluation, and storage of biological specimens for genetic or molecular analysis in the current trial and for future use in ancillary studies, if applicable	N/A

For peer review only - http://bmjopen.bmj.com/site/about/guidelines.xhtml

Supplemental appendix 2- TIDieR checklist (52)

Item number	Item	Where located **	
		Primary paper (page or appendix number)	Other † (details)
	**BRIEF NAME**		
**1.**	Provide the name or a phrase that describes the intervention.	15	
	**WHY**		
**2.**	Describe any rationale, theory, or goal of the elements essential to the intervention.	15-16 and Supplemental appendix 3	https://doi.org/10.3310/phr08040 (28)
	**WHAT**		
**3.**	Materials: Describe any physical or informational materials used in the intervention, including those provided to participants or used in intervention delivery or in training of intervention providers. Provide information on where the materials can be accessed (e.g. online appendix, URL).	16-17 and Supplemental appendix 3	______________
**4.**	Procedures: Describe each of the procedures, activities, and/or processes used in the intervention, including any enabling or support activities.	16-17	______________
	**WHO PROVIDED**		
**5.**	For each category of intervention provider (e.g. psychologist, nursing assistant), describe their expertise, background and any specific training given.	17	______________
	**HOW**		
**6.**	Describe the modes of delivery (e.g. face-to-face or by some other mechanism, such as internet or telephone) of the intervention and whether it was provided individually or in a group.	17	______________
	**WHERE**		
**7.**	Describe the type(s) of location(s) where the intervention occurred, including any necessary infrastructure or relevant features.	17	______________
	**WHEN and HOW MUCH**		
**8.**	Describe the number of times the intervention was delivered and over what period of time including the number of sessions, their schedule, and their duration, intensity or dose.	18	https://doi.org/10.3310/phr08040 (28)

For peer review only

	**TAILORING**		
**9.**	If the intervention was planned to be personalised, titrated or adapted, then describe what, why, when, and how.	15-18	______________
	**MODIFICATIONS**		
**10.‡**	If the intervention was modified during the course of the study, describe the changes (what, why, when, and how).	N/A	______________
	**HOW WELL**		
**11.**	Planned: If intervention adherence or fidelity was assessed, describe how and by whom, and if any strategies were used to maintain or improve fidelity, describe them.	19	______________
**12.‡**	Actual: If intervention adherence or fidelity was assessed, describe the extent to which the intervention was delivered as planned.	N/A	______________

** **Authors** - use N/A if an item is not applicable for the intervention being described. **Reviewers** – use '?' if information about the element is not reported/not sufficiently reported.

† If the information is not provided in the primary paper, give details of where this information is available. This may include locations such as a published protocol or other published papers (provide citation details) or a website (provide the URL).

‡ If completing the TIDieR checklist for a protocol, these items are not relevant to the protocol and cannot be described until the study is complete.

For peer review only

For peer review only - http://bmjopen.bmj.com/site/about/guidelines.xhtml

Supplemental appendix 3- SMS Logic Model and sample text messages

a) Supporting MumS intervention logic model

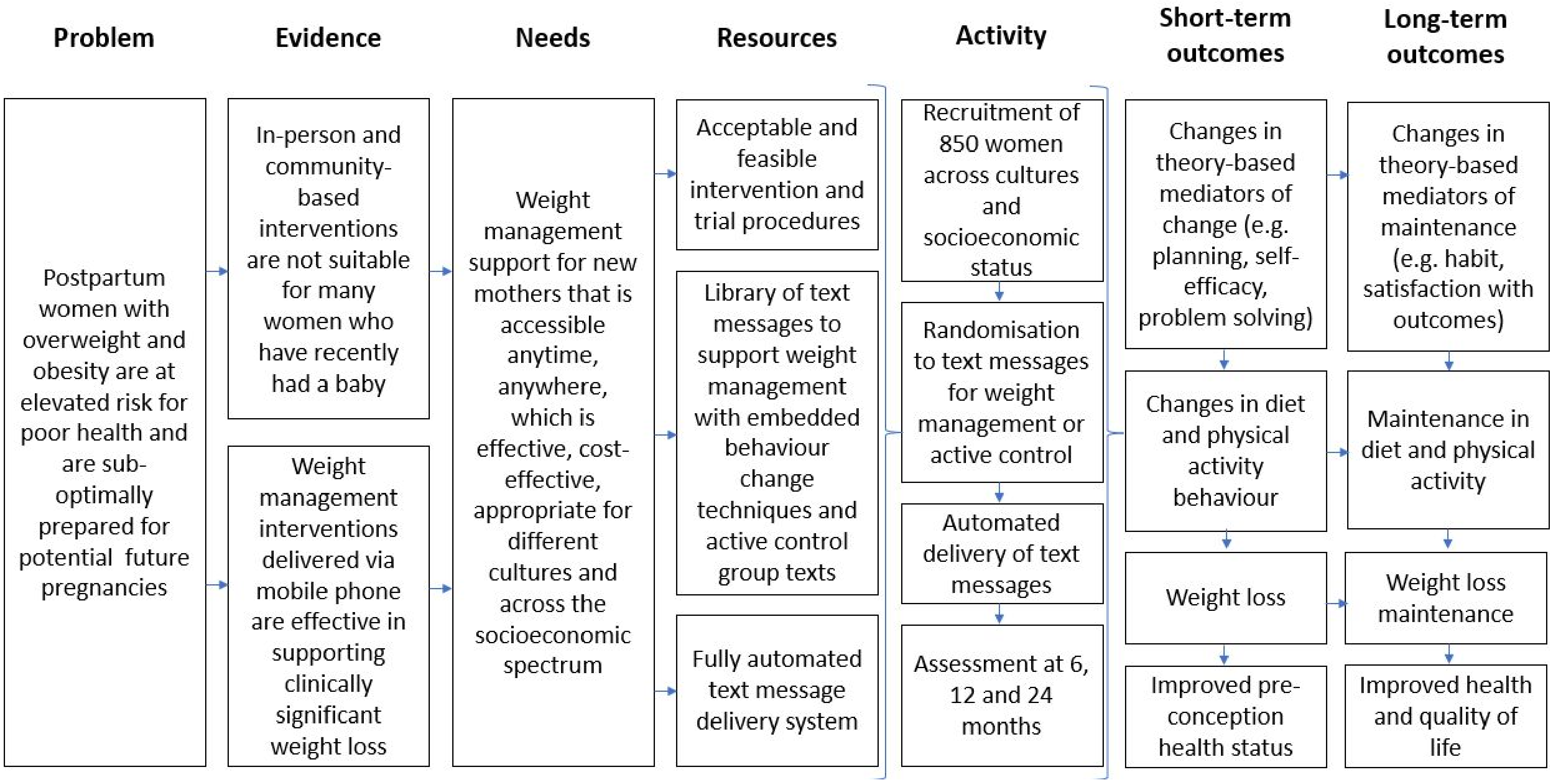

b) Supporting MumS sample text messages

Type of text message		Example of message sent	Example of automated responses (if applicable)
**Core intervention text message**		*'Another tip for success: Keep track of what you eat. People who regularly monitor what they eat are more likely to meet their weight loss target. Stay tuned for more texts on food tracking'.*	
**Weekly self-weighing reminder**	**Months 0-6**	*'What are the scales saying this week? Text us e.g. 11 st 5, or 73.3 kg'.*	N/A
	**Months 7-12**	*'Weekly weighing = your very own early warning system! Step on the scales today and text us your weight followed by UP, DOWN, SAME. e.g. "10 st 7 SAME" or "66.7 kg SAME'.*	Up: *'Don't give up! The best thing to do is prevent these small regains from turning into bigger regains. What could you do this week? E.g. maybe exercise a little more or be more mindful of portion sizes?'*
			Down: *'Success! Your efforts are paying off! All the hard work is worth it :)'.*

For peer review only

			Same: *'Maintaining your weight is something to be proud of! Treat yourself to a reward e.g. a relaxing bath or simply 10 minutes to yourself!'*
**Yes/No question**		*'WEEK 14. Have you set your activity goal for the week ahead? Text Y11 for yes or N11 for no'.*	Y11 (yes): *'Great job, setting goals is clearly becoming one of your weekly habits :) Alongside your activity goal, set a food goal too and you're onto a double winner!'*
			N11 (no): *'New to the whole activity thing? Set a goal to do a small amount of an activity that you enjoy - for example, a brisk 15-minute walk or a zumba routine when your little one goes to sleep. It will help motivate you and give you a boost of confidence to do more next time'.*

For peer review only - http://bmjopen.bmj.com/site/about/guidelines.xhtml

**Trigger words**	*'You can always text us back for more help – text us with one of these trigger words and texts with instant support will appear on your phone: 'BAD DAY', 'SLIP-UP', 'TIRED' and 'CRAVE'.*	Bad day: *'Everyone has these type of days. Don't be hard on yourself! Press delete for the day so far and start afresh!'*
		Slip-up: *'Don't let one slip-up discourage you. You have the ability to pick up right where you left off!'*
		Tired response: *'Fresh air can work wonders! Getting out of the house for a while can help you think more clearly and boost your energy'.*
		Crave response: *'Tried brushing your teeth or chewing a piece of sugar free gum? It might work!'*
**Weight management when breastfeeding**	*'Breastfeeding can make you hungry. Just fill up on the good stuff (fruit and vegetables, wholegrain cereals) and the good eating habits will stay with you even when the breastfeeding stops :) '*	N/A

For peer review only

**Smoking cessation and weight management**	*'It is only normal to have cravings! Why not exercise to combat it? Try a short walk (even for just 5 mins) if a craving hits, the distraction can really help.'*	N/A
**Intervention "buddy system"**	*'Welcome to WEEK 34. Many people that have lost weight and kept it off have done so with support from family and friends! You can opt in to get your friend/partner/family member to receive SMS texts for extra support - text 'SUPPORT' followed by the person's mobile number to sign up (ask their permission first). E.g. Support 0780000000'.*	N/A
**Active control**	*'1, 2, 3! Your toddler might know how to count to 3 or more...or they might get some of the numbers mixed up. Whatever stage they are at, just keep encouraging them by counting from 1 to 10'.*	N/A

For peer review only

For peer review only - http://bmjopen.bmj.com/site/about/guidelines.xhtml

Supplemental appendix 4- Informed consent materials [32]

a) Participant Information Sheet

PARTICIPANT INFORMATION SHEET

The Supporting MumS (SMS) study

We would like to invite you to take part in this research study which is taking place in Northern Ireland, England, Scotland and Wales.

Before you decide whether to take part, it is important that you understand why we are doing this research and what it will involve.

Please take time to read the information carefully and discuss it with friends/relatives, if you wish. If you have any questions, please contact us using the details given at the end of the sheet.

Why are we doing this study?

Welcoming a baby brings lots of joy and happiness but being a new mum also means life is a bit busy! There may be areas where mums could benefit from more support. Mums often talk about having a baby as a time when their weight started to creep up and how they would welcome support with this. We are interested in finding out if text messages can help support weight management in the first two years after having a baby.

For this study, the term 'mum' or 'mother' includes people who do not identify as women but are pregnant or have given birth. The term 'your child' means your youngest child at the time of signing up to the study.

Why have I been asked to take part?

You have been chosen to take part, as you are over 18 years old and have had a baby within the past two years and have a body mass index over 25kg/m^2.

Do I have to take part?

No, it is up to you whether or not you want to take part. If you do decide to take part, you are free to leave the study at any stage without giving a reason for doing so. Your usual health care will not be affected at any time.

What will happen if I take part?

After you read this leaflet we will contact you to discuss the study in more detail and answer any further questions you may have.

If you decide to take part, you will be asked to sign the consent form for the study and complete some study measurements (described below). You will then receive text messages to your mobile phone for 12 months. The number of text messages you receive each week will vary between 3 and 14 per week. The text messages are delivered to you by a secure text message system owned and operated by the London School of Hygiene and Tropical Medicine.

Mums who take part in the study will get text messages on **ONE** of the two topics shown below. It is important that you understand that the topic is randomly chosen for you and that there is a 50:50 chance you will get texts on either topic 1 or topic 2:

1. Information and advice on making healthy food choices and keeping physically active to help you lose weight.

OR

2. Information and advice on your child's health and development.

For peer review only - http://bmjopen.bmj.com/site/about/guidelines.xhtml

What information will you collect from me if I take part?

We would meet with you 4 times (during a 24 month period) to collect some measurements and information. These visits will take place at the start of the study, at 6 months, at 12 months (when the text messages stop) and at 24 months (12 months after you stopped receiving the text messages).

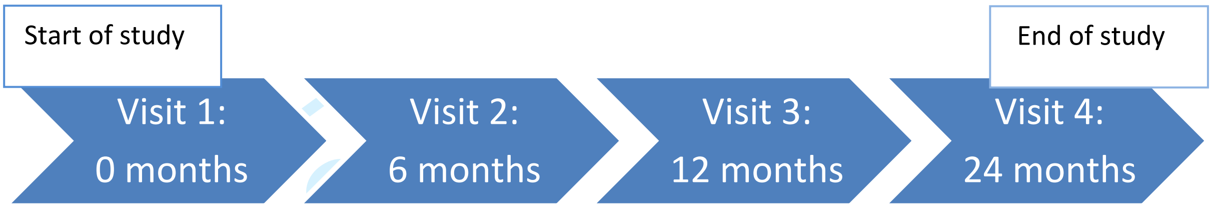

For the visits, the researcher will either visit you at your home or you can come and meet the researcher at a convenient location such as a University building or a local community venue; whichever is most convenient for you.

You will receive a £25 voucher on completion of each visit in recognition of your time to complete the research measures. This will be a total of £100 over the 24-month study, if all visits are completed. Each visit will last approximately 1 hour.

At each visit we will collect the following information:

a) We will measure your height (only at visit 1) and will ask you to step on the scales to collect information on weight and will measure your waist circumference (this will take about 10 minutes).
b) We will ask you to complete a questionnaire booklet about your physical and mental health, lifestyle and wellbeing. The booklet will take approximately 35 minutes to complete. You will have the option of:
   - completing a printed questionnaire booklet in your own time and posting it back to us in a stamped addressed envelope we provide; OR
   - completing the questionnaire online by accessing a link we give you; OR
   - completing the questionnaire with the researcher at the study visit or over the phone.

When (a) and (b) above has been collected from you, we will arrange for the £25 voucher to be sent to you.

We would also ask you to consider taking part in a short (approximately 20 minutes) telephone interview twice during the study (at the 6 month visit and the 12 month visit) to help us understand how you found the text messages. *This part is optional.*

For peer review only

For peer review only - http://bmjopen.bmj.com/site/about/guidelines.xhtml

Linking to routine health-related data in the future (this part is optional)

We would also ask you to consider allowing us to collect health-related data about you and your child in the future. We would do this by linking with organisations that collect data about the services they provide to women and children. For example, the NHS keeps records, known as 'routine data' about how we use different services such as the health service.

Routine data is stored electronically on different systems. To obtain your routine data from other systems, we would share some personally-identifiable details such as name, date of birth, address and NHS number to the organisations that manage these systems and they will send us data back. This will all be done using secure data transfer systems that have been set up carefully to keep your information safe.

Doing this helps us to understand if a study like this has any longer-term benefits and how we might improve health services in the future.

We may access routine data from these sources:

- Health records such as GP and dental records, maternity and health visiting records and disease registers
- Local authority and social care
- Family/children's centres
- Education or school
- National child measurement programme
- Voluntary organisations

For example, from maternity records we may collect information on any further pregnancies you have and from GP records we may collect information on any diagnosed conditions such as high blood pressure or type 2 diabetes.

If you consent to us linking to routine health-related data on the consent form, we will ask you for your NHS number. This number will only be stored by QUB for 15 years and will only be used for this purpose. It will be stored securely, separately from your other data, in a password protected, encrypted file on computers that are only accessible to authorised members of the research team. If at any time you want us to remove your NHS number from our records, you can do so by contacting us as per details given at the end of this information sheet. Otherwise, it will be removed from our records after 15 years. An example of how we would link your routine data is shown below.

You do not have to consent to data linkage to participate in this study. This part is optional.

For peer review only - http://bmjopen.bmj.com/site/about/guidelines.xhtml

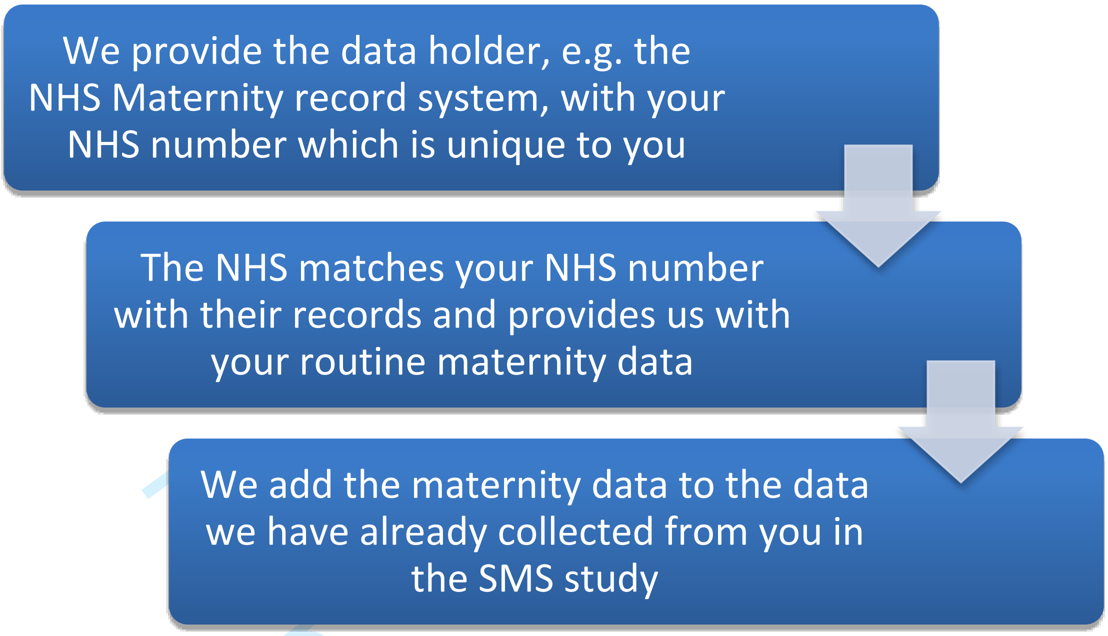

Example of routine data linkage process

What are the possible advantages and disadvantages of taking part?

By taking part in this research you will be helping us find the best ways to support new mums. In the study questionnaires and interviews, we will collect information about your postnatal physical and mental health. This information will be used to help us understand how we might improve services for women after they have a baby. Some of the messages you receive may be helpful for you and/or your baby by helping you to lose weight or by providing you with information about child health and development. We do not anticipate any risks from taking part in this study.

How will we use information about you?

We will need to use information from you for this research project. This information will include your name, address, NHS number, date of birth, telephone number, email address and date of birth of your youngest child. People will use this information to do the research or to check your records to make sure that the research is being done properly.

People who do not need to know who you are will not be able to see your name or contact details. Your data will have a code number instead.

We will keep all information about you safe and secure.

For peer review only - http://bmjopen.bmj.com/site/about/guidelines.xhtml

Once we have finished the study, we will keep some of the data so we can check the results. We will write our reports in a way that no-one can work out that you took part in the study.

Will my taking part in the study be kept confidential?

Yes, all your data will be treated with the strictest confidence and your details will not be shared with anybody outside of the research team, unless there is a serious risk of harm to you or others.

Any information collected from you will be stored securely on password protected files on password protected computers that only the research team can access. Hard copies of documents will be kept in locked filing cabinets in locked offices that are only accessible to the research team and are located in a building that is locked outside normal working hours.

Any interviews conducted over the telephone will be audio-recorded and the recording will then be typed up for research purposes. The typed transcript will not contain any names and will be labelled with your unique study number. The recording will be destroyed once the typed transcript is prepared.

Study data will be kept separate from personal information (such as name and address). Only members of the research team will have access to view identifiable data. However, in some instances, inspectors from regulatory authorities may need to access data for checking the quality of the research. All members of the research team and regulatory bodies are trained in data protection and will comply with the requirements of data protection legislation.

Once the study is complete and it is no longer necessary to keep identifiable information or contact details, we will destroy our records of this personal information.

The study questionnaire asks some questions about mental and physical health. If we have any concerns that your responses to such questions may indicate you are at risk of postpartum depression, we will inform you and your GP by letter as part of our duty of care to you. If at any stage we have concerns that you, or someone else, is at risk of harm then we are obliged to tell the relevant services, for example your GP or social services.

What are your choices about how your information is used?

- You can stop being part of the study at any time, without giving a reason, but we will keep information about you that we already have.

For peer review only - http://bmjopen.bmj.com/site/about/guidelines.xhtml

- We need to manage your records in specific ways for the research to be reliable. This means that we won't be able to let you see or change the data we hold about you.
- If you agree to take part in this study, you will have the option to take part in future research using your data saved from this study.

Where can you find out more about how your information is used?

You can find out more about how we use your information:

- at www.hra.nhs.uk/information-about-patients/
- in our privacy notice from go.qub.ac.uk/SMSstudy or ask the research team for a copy
- by asking one of the research team
- by sending an email to info.compliance@qub.ac.uk, or
- by ringing us on 07341 888415.

How will I find out the results of this project?

When the study is finished and the study information has been analysed, we will send you a summary of the results.

Who is organising and funding the research?

For this study we will be recruiting women from all four countries in the UK. The work is being led by Professor Michelle McKinley from Queen's University Belfast. Researchers from Universities in London, Cardiff, Stirling and the Bradford Teaching Hospitals NHS Foundation Trust are leading recruitment in their areas.

Mums have helped us design our text messages and advise on how the study is conducted and will continue to do so.

The study is funded by National Institute of Health Research and the Public Health Agency Northern Ireland.

Has this study been approved for safety by an ethics committee? Is this study safe?

This study has been reviewed and approved by the West of Scotland Research Ethics Committee 4 and IRAS ID 305557.

For peer review only - http://bmjopen.bmj.com/site/about/guidelines.xhtml

What will happen if I don't want to carry on with the study?

You can withdraw from the study at any time, without giving a reason by contacting the research team (contact details at the end of this leaflet). If you do decide to withdraw from the study, we will use the data collected up to that point, but we will not collect any more data.

What if something goes wrong?

There are no special compensation arrangements. Queen's University Belfast will provide indemnity for this study. If you are harmed due to someone's negligence, then you may have grounds for legal action but you may have to pay for it.

What if there is a problem?

If you have a concern about any aspect of this study, you can speak to the researchers who will do their best to answer your questions (contact details on the last page).

Should you remain unhappy and wish to make a formal complaint, you can contact:

Research Governance Team at Queen's University Belfast,
63 University Road, Belfast
BT7 1NF
(Telephone: 028 9097 2529; Email: researchgovernance@qub.ac.uk).

Contact for Further Information:

Researchers' Name, Address, Email address, Telephone (site specific)	Dr Dunla Gallagher<br>SMS Trial Manager<br>Centre for Public Health<br>Queen's University Belfast<br>School of Medicine, Dentistry & Biomedical Sciences<br>Institute of Clinical Sciences (Block B)<br>Grosvenor Road<br>Belfast, N.Ireland<br>BT12 6BA<br>Tel: 07341 888415<br>Email: d.gallagher@qub.ac.uk

Please ask us if there is anything that is not clear or if you would like more information.

For peer review only - http://bmjopen.bmj.com/site/about/guidelines.xhtml

b) Privacy Notice

PRIVACY NOTICE

The Supporting MumS (SMS) study

Queen's University Belfast, Bradford Teaching Hospitals NHS Foundation Trust, Cardiff University, the University of Stirling, and the London School of Hygiene & Tropical Medicine ("we", "us" and "our") are committed to protecting your personal data.

The notice is addressed to individuals who are taking part in the research study entitled 'The Supporting MumS (SMS) study' ("you" and "your").

This Privacy Notice tells you why we need to collect personal information about you, what we will do with it, and how we will look after it. It also tells you about your legal rights in relation to your Personal Data.

If you have any questions about this privacy notice, please contact us. Contact details are provided below.

WHO WE ARE

1. We are a group of expert researchers from the organisations listed above. The study is led by Queen's University Belfast and is funded by National Institute of Health Research and the Public Health Agency Northern Ireland.

2. All our research is underpinned by policies and procedures that ensure we comply with regulations and legislation that govern the conduct of research. This includes data protection legislation: the UK General Data Protection Regulation (GDPR) and the Data Protection Act 2018 (DPA). In the case of health and social care research, which should serve the public interest, where we have to demonstrate that our research serves the interests of society as a whole.

HOW YOUR PERSONAL DATA IS COLLECTED

3. **Information you provide:** When you consent to take part in the SMS study we will ask for information about you, such as your name, address, NHS number, date of birth, telephone number, email address and date of birth of your youngest child. This is known as your "Personal Data". We may also ask you for some special categories of information (for example information about your physical and mental health). This is known as your "Sensitive Personal Data" and has additional protections.

4. **Data from other sources:** We will also collect information about you from other sources and this also forms part of "Personal Data" and "Sensitive Personal Data". This includes information from:

For peer review only - http://bmjopen.bmj.com/site/about/guidelines.xhtml

- the system we are using to send and receive text messages for the study, for example we will automatically collect the replies you send to the text messages. This system is owned and operated by the London School of Hygiene & Tropical Medicine.
- routine health-related data (if you have consented to this option) such as NHS records, GP and dental records, maternity and health visiting records and disease registers. To obtain your routine data from these systems, we will share some personally-identifiable details such as name, date of birth, address and NHS number to each of these organisations and they will send us your data back. This will all be done using secure data transfer systems that have been set up carefully to keep your information safe and complies with regulations and legislation governing the conduct of research.

HOW WE USE YOUR PERSONAL DATA

5. We use your Personal Data and Sensitive Personal Data in the following ways:
    - to contact you to invite you to take part in the study and to discuss research-related queries. If you do not consent to take part in the study, your personal data, such as contact details (e.g. name, address, phone number), will be confidentially destroyed and will not be retained by us.
    - after consent to take part is provided, personal data is used to contact you to arrange and conduct study visits, to request information or to send you relevant study documents or equipment and to inform you about any relevant study developments.
    - access to, and sharing of, special category and sensitive personal data is controlled very carefully. Sensitive personal data is collected and pseudonymised – this means each person who takes part in this study will be given a unique study number and this unique study number is used on all the study paperwork and in study databases. Only authorised members of the SMS research team will be able to link your study number to your name, contact details and NHS number.

6. In order to protect your rights and freedoms when using your personal information for research and to process special category information we have special safeguards in place to protect your information, and all information is kept in line with our policies and regulatory requirements.

7. In addition to the above safeguards, data protection legislation requires us to meet the following standards when we conduct research with your personal information:
    - the research will not cause damage or distress to someone (e.g., physical harm, financial loss or psychological pain).
    - the research is not carried out in order to do or decide something in relation to an individual person, unless the processing is for medical research approved by a research ethics committee.
    - the Data Controller has technical and organisational safeguards in place (e.g. appropriate staff training and security measures).
    - if processing a special category of data, this must be subject to a further public interest test to make sure this particularly sensitive information is required to meet the research objectives.

For peer review only - http://bmjopen.bmj.com/site/about/guidelines.xhtml

LEGAL BASIS FOR COLLECTING AND USING YOUR PERSONAL DATA

8. We will only use your Personal Data if we have valid reasons for doing so. These reasons are known as our "legal basis for processing".

9. In the context of research, the lawful basis upon which we will process your personal information is usually where "Processing is necessary for the performance of a task carried out in the public interest or in the exercise of official authority vested in the controller" (Article 6 of GDPR).

10. We also collect and use more sensitive personal information (Special Category data) where "the processing is necessary for archiving purposes in the public interest, scientific or historical research purposes or statistical purposes… which shall be proportionate to the aim pursued, respect the essence of the right to data protection and provide for suitable and specific measures to safeguard the fundamental rights and the interests of the data subject". (Article 9 of GDPR).

WHO WE SHARE YOUR DATA WITH

11. In line with our Data Protection Policy and Procedures we can share your information, including Personal Data and Sensitive Personal Data, with the following parties for the following research purposes:
    - London School of Hygiene and Tropical Medicine (LSHTM) text message system – to allow you to receive and reply to the text messages (the LSHTM is a study collaborator)
    - Designated research personnel for the purposes of achieving the research outcomes
    - The Northern Ireland Clinical Trials Unit (NICTU) for the purposes of entering your completed questionnaires onto a study database (NICTU is a study collaborator)
    - Qualtrics research software supplier for the purposes of the online completion of the study questionnaires (privacy notice can be viewed here - https://www.qualtrics.com/support/survey-platform/getting-started/data-protection-privacy/).
    - Transcription services for the purpose of transcribing recorded interviews (if consent provided for this optional element)
    - Organisations in-charge of the collection and administration of routine data for the purposes of achieving the research outcomes (if consent provided for this optional element)

12. In all cases, information shared will be on a need to know basis, not excessive and with all appropriate safeguards in place to ensure the security of your information.

13. When we use third parties (known as data processors) to carry out a task on our behalf, such as a transcription service as indicated above, we have contractual terms, policies and procedures to ensure confidentiality is respected.

DATA PROCESSING OUTSIDE EUROPE

14. We will not transfer your Personal Data and Sensitive Personal Data outside of the United Kingdom and European Economic Area.

For peer review only - http://bmjopen.bmj.com/site/about/guidelines.xhtml

HOW LONG YOUR INFORMATION WILL BE KEPT?

15. Information where you can be identified will be kept for a minimum amount of time and in accordance with research objectives. Researchers will de-identify information, i.e. anonymise or pseudonymise, as soon as possible as described in point 5.

16. We will keep your Personal Data and Sensitive Personal Data for up to 10 years from when the research has been completed to allow for full and final publication of the research results. If you have provided consent (this is optional), we will retain your record of consent and personal data (name, telephone number, email address, address) for the purpose of conducting longer-term follow-up for the SMS study or to inform you about future studies related to health and lifestyle that we may be conducting. If you have consented, we will retain your NHS number for data linkage purposes. You can decline to receive communications at any stage. We will only keep your information if we need it for one of the reasons described above.

17. We place great importance on the security of the Personal Data that we hold, including the use of physical, technological and organisational measures to ensure your information is protected from unauthorised access and against unlawful processing, accidental loss, alteration, disclosure, destruction and damage.

YOUR RIGHTS

18. The DPA provides you with a number of legal rights in relation to your Personal Data, including the right:

- to request access to your Personal Data;
- to request correction of your Personal Data that is wrong or incomplete;
- to request erasure or the restriction of processing of your Personal Data;
- to request the transfer of your Personal Data in a structured; commonly used machine-readable format;
- not to be subject to automated decision making; and
- to withdraw your consent.

19. If you wish to exercise any of the rights set out above, or require further information about any of the rights, please contact us.

20. There may also be times where we cannot stop using your Personal Data when you ask us to, but we will tell you about this if you make a request.

For peer review only - http://bmjopen.bmj.com/site/about/guidelines.xhtml

CONTACTING US

21. If you have any questions or comments about this privacy notice, please contact the SMS team:

    Dr Dunla Gallagher
SMS Trial Manager
Centre for Public Health
Institute Clinical Science A
School of Medicine, Dentistry & Biomedical Sciences
Queen's University Belfast
d.gallagher@qub.ac.uk

    Professor Michelle McKinley
SMS Chief Investigator
Centre for Public Health
Institute Clinical Science A
School of Medicine, Dentistry & Biomedical Sciences
Queen's University Belfast
m.mckinley@qub.ac.uk

    Alternatively, you can contact:
Data Protection Officer
Information Compliance Unit
Lanyon South
Queen's University Belfast
University Road
BT7 1NN
Info.compliance@qub.ac.uk

COMPLAINTS

22. You have the right to complain about how we treat your Personal Data and Sensitive Personal Data to the Information Commissioner's Office (ICO). The ICO can be contacted at:

    Information Commissioner's Office
Wycliffe House
Water Lane
Wilmslow
Cheshire
SK9 5AF

CHANGES TO THIS NOTICE

23. We may update this Privacy Notice from time to time. We will notify you of the changes where we are required by law to do so.

For peer review only - http://bmjopen.bmj.com/site/about/guidelines.xhtml

c) Study Consent Form

CONSENT FORM
The Supporting MumS (SMS) study

Screening Number: __ __ __ __ __ Participant ID number: __ __ __ __ __

		Please **initial** box
1.	I confirm that I have read and understood the Participant Information Sheet dated 05/04/2022 (Version 4.0) and privacy notice dated 30/11/2021 (Version 1.0) for the above study and have been given copies to keep.	☐
2.	I have had the opportunity to consider the information, ask questions and have had these answered to my satisfaction.	☐
3.	I understand that my participation is voluntary and that I am free to withdraw at any time, without giving any reason and without my medical care or legal rights being affected.	☐
4.	I understand that information collected about me for the study (including personally identifiable information) may be looked at by responsible individuals in the study team and regulatory authorities supervising the study.	☐
5.	I understand that all information collected about me during the course of the research will be kept strictly confidential and processed in compliance with data protection legislation.	☐
6.	I understand that some of the data collected about me will be processed by third parties (known as data processors), such as software and transcription companies, and that contractual terms, policies and procedures will be put in place to ensure confidentiality is respected.	☐
7.	I give permission for my GP to be informed of any concerning medical issues detected during study visits.	☐
8.	I am aware of the potential risks and benefits of this research study, as described in the Participant Information Sheet and discussed with the researcher.	☐
9.	I understand that I will not be identifiable in any published report using data from this study.	☐
10.	I agree to my contact details being kept so I can be informed of the study findings.	☐
**11.**	**I consent to take part in the above study.**	☐

For peer review only - http://bmjopen.bmj.com/site/about/guidelines.xhtml

	OPTIONAL ELEMENTS (please initial box if you consent)	
12.	I am willing to be contacted to take part in two short (20-30 minute) telephone interviews during the study to provide feedback to the research team.	☐
13.	I am willing to be contacted in the future about a follow-up to this study.	☐
14.	I consent to be contacted by the University about future research studies related to diet or lifestyle and health for which I may be eligible.	☐
15.	I consent to my personal health information, including my NHS number and date of birth, being released to organisations that hold routine health-related data so they can locate information about me that is held in their database.	☐
16.	I consent to organisations holding my routine health-related data performing data linkage and releasing the information about me from its databases to the SMS Study research team.	☐

Name of Participant (Block capitals)	Signature	Date

To be completed by the Principal Investigator or nominee

I, the undersigned, have taken the time to fully explain to the above patient the nature and purpose of the study in a way that they could understand. I have explained the risks involved as well as the possible benefits. I have invited them to ask questions on any aspect of the study that concerned them.

Name of Researcher (Block capitals)	Signature	Date

Researchers' Name, Address, Email address, Telephone (site specific)	Dr Dunla Gallagher<br>SMS Trial Manager<br>Centre for Public Health, Queen's University Belfast<br>School of Medicine, Dentistry & Biomedical Sciences<br>Institute of Clinical Sciences (Block B)<br>Grosvenor Road, Belfast, N.Ireland, BT12 6BA<br>Tel: 07341 888415<br>Email: d.gallagher@qub.ac.uk

For peer review only - http://bmjopen.bmj.com/site/about/guidelines.xhtml

535 **Supplemental appendix 5- World Health Organization Trial Registration Data Set**

**Data Category**	**Information**
Primary Registry and Trial Identifying Number	ISRCTN Registry; ISRCTN16299220
Date of Registration in Primary Registry	10/02/2022
Secondary Identifying Numbers	FUNDERS Number: NIHR131509<br>Sponsor Number: B21/24<br>IRAS Number: 305557<br>REC Reference: 22/WS/0003
Source(s) of Monetary or Material Support	National Institute for Health and Care Research (NIHR) Public Health Research (PHR). Intervention costs provided by the Public Health Agency (Northern Ireland).
Primary Sponsor	Queen's University Belfast
Secondary Sponsor(s)	None
Contact for Public Queries	Dr Dunla Gallagher<br>SMS Trial Manager<br>d.gallagher@qub.ac.uk<br>07341 888415<br>Centre for Public Health<br>Queen's University Belfast<br>Institute of Clinical Sciences B<br>Grosvenor Road<br>Belfast<br>BT12 6BJ
Contact for Scientific Queries	Professor Michelle McKinley<br>Chief Investigator<br>m.mckinley@qub.ac.uk<br>028 9097 8936<br>Centre for Public Health<br>Queen's University Belfast<br>Institute of Clinical Sciences A<br>Grosvenor Road<br>Belfast<br>BT12 6BA

For peer review only

Public Title	The Supporting MumS (SMS) study: can we use an automated text message intervention for weight management in women after childbirth?
Scientific Title	Effectiveness and cost effectiveness of an automated text message intervention for weight management in postpartum women with overweight or obesity: the Supporting MumS (SMS) Randomised Controlled Trial.
Countries of Recruitment	United Kingdom (Northern Ireland, Scotland, England and Wales).
Health Condition(s) or Problem(s) Studied	Overweight and obesity in postpartum women.
Intervention(s)	Supporting MumS intervention group- will receive automated text messages about weight loss and maintenance of weight loss for 12 months. The text messages will focus on diet and physical activity with embedded behaviour change techniques known to be positively associated with weight management.<br><br>Active control group- will receive automated text messages about child health and development for 12 months.
Key Inclusion and Exclusion Criteria	Inclusion criteria<br>• Women (as per NICE Postnatal care guideline NG194[1], the term 'woman' is taken to include people who do not identify as women but who are pregnant or have given birth)<br>• Aged > 18 years old<br>• BMI ≥25 kg/m^2<br>• Have had a baby within the last two years<br><br>Exclusion criteria<br>• Baby less than 6 weeks old<br>• No access to a mobile phone to receive personal messages<br>• Insufficient English to understand short written messages<br>• Currently pregnant<br>• Recent or planned bariatric surgery<br>• Eating disorder

For peer review only

	• On a specialist diet and receiving dietetic care<br>• Taking part in another weight management research study currently, or in the last 3 months
Study Type	Two-arm parallel groups randomised controlled trial to test the effectiveness and cost-effectiveness of a 12-month text-messaging behavioural weight management intervention in supporting weight loss for women with overweight and obesity in the postpartum period, compared with an active control group receiving text messages related to child health and development for 12 months.<br><br>Women will be recruited from all four countries in the UK using community-based recruitment as well as signposting via routine contact with health professionals.<br><br>Written informed consent will be obtained, baseline data collected and then participants will be randomised. Participants will be block randomised and randomisation will be stratified by site. The randomisation sequence will be developed in STATA by a statistician who is independent of the study team. The randomisation will be implemented via the London School of Hygiene and Tropical Medicine secure remote web-based system which will link directly with the text message database and will deliver the intervention or active control content according to the random allocation sequence.<br><br>Participants will become aware of their group allocation when they start to receive the messages. Researchers who are recruiting women to the study and collecting outcome data are blinded to group allocation.
Date of First Enrolment	Actual date of enrolment of the first participant: 20/05/2022
Sample Size	Target sample size: 888<br>Final number of participants that the trial enrolled: 892
Recruitment Status	Complete: participants are no longer being recruited or enrolled.

For peer review only - http://bmjopen.bmj.com/site/about/guidelines.xhtml

Primary Outcome	Weight change from baseline to 12 months (kg), measured using calibrated scales.
Secondary Outcome	1. Waist circumference measured by a flexible measuring tape at baseline, 6, 12 and 24 months, and mean BMI (kg/m^2) and the proportions of women gaining a substantial amount of weight (>5kg) at 12 and 24 months.<br><br>2. Health behaviours are measured by: Fat and Fibre Barometer; self-report questionnaire on sugar and alcohol intake; International Physical Activity Questionnaire (IPAQ) - Short form; and self-report questionnaire on infant feeding practices at baseline, 6, 12 and 24 months.<br><br>3. Study acceptability is measured by: recruitment; retention; engagement with the two-way text messages over 12 months; self-reported questionnaire on participant satisfaction with SMS messages (at 6 and 12 months) and rating of intervention (at 12 months); and qualitative interviews (at 6 and 12 months).<br><br>4. Economic evaluation outcomes are measured by: self-report questionnaire on health service resources use, medication usage and lifestyle-related costs; the EuroQol 5-dimension (EQ-5D) quality of life questionnaire; and the ICEpop Capability Measure for Adults (ICECAP-A) at baseline, 6, 12 and 24 months.<br><br>5. Moderators of intervention effect are measured by: Edinburgh Postnatal Depression Scale (EPDS); Generalised Anxiety Disorder (GAD-7); Pittsburgh Sleep Quality Index; and self-report questionnaire on confidence and desire for weight loss and maintenance at baseline, 6, 12 and 24 months.<br><br>6. Mediators of intervention effect are measured by: Health Action Process Approach (HAPA) for diet and exercise; Self-Report Behavioural Automaticity Index for diet and exercise; Self-regulation of eating behaviour questionnaire; self-report questionnaire on monitoring and goal setting for diet and

For peer review only

	exercise; Motivation for weight loss scale; Social support for eating & exercise questionnaire; and Rosenberg Self-Esteem Scale at baseline, 6, 12 and 24 months.
Ethics Review	Approved 24/02/2022 (Ref: 22/WS/0003) by West of Scotland REC 4 (West of Scotland Research Ethics Service, Ward 11, Dykebar Hospital, Grahamston Road, Paisley, PA2 7DE, United Kingdom; +44 (0)141 3140213; WoSREC4@ggc.scot.nhs.uk).
Completion date	30/04/2025
Summary results	Intention to publish date: 30/04/2026.
IPD Sharing Statement	Available on request. Formal requests to be made in writing to the CI (Prof M McKinley; m.mckinley@qub.ac.uk).

For peer review only

SPIRIT checklist

SPIRIT 2013 Checklist: Recommended items to address in a clinical trial protocol and related documents*

Section/item	Item No	Description	Addressed on page number
**Administrative information**			
Title	1	Descriptive title identifying the study design, population, interventions, and, if applicable, trial acronym	4
Trial registration	2a	Trial identifier and registry name. If not yet registered, name of intended registry	2
	2b	All items from the World Health Organization Trial Registration Data Set	Supplemental appendix 3
Protocol version	3	Date and version identifier	4
Funding	4	Sources and types of financial, material, and other support	50
Roles and responsibilities	5a	Names, affiliations, and roles of protocol contributors	4-7, 49-50
	5b	Name and contact information for the trial sponsor	7
	5c	Role of study sponsor and funders, if any, in study design; collection, management, analysis, and interpretation of data; writing of the report; and the decision to submit the report for publication, including whether they will have ultimate authority over any of these activities	7
	5d	Composition, roles, and responsibilities of the coordinating centre, steering committee, endpoint adjudication committee, data management team, and other individuals or groups overseeing the trial, if applicable (see Item 21a for data monitoring committee)	40
**Introduction**			
Background and rationale	6a	Description of research question and justification for undertaking the trial, including summary of relevant studies (published and unpublished) examining benefits and harms for each intervention	8-12
	6b	Explanation for choice of comparators	12-13
Objectives	7	Specific objectives or hypotheses	13-14
Trial design	8	Description of trial design including type of trial (eg, parallel group, crossover, factorial, single group), allocation ratio, and framework (eg, superiority, equivalence, noninferiority, exploratory)	14

For peer review only

For peer review only - http://bmjopen.bmj.com/site/about/guidelines.xhtml

Methods: Participants, interventions, and outcomes

Study setting	9	Description of study settings (eg, community clinic, academic hospital) and list of countries where data will be collected. Reference to where list of study sites can be obtained	14
Eligibility criteria	10	Inclusion and exclusion criteria for participants. If applicable, eligibility criteria for study centres and individuals who will perform the interventions (eg, surgeons, psychotherapists)	14-15, 26-27
Interventions	11a	Interventions for each group with sufficient detail to allow replication, including how and when they will be administered	15-19
	11b	Criteria for discontinuing or modifying allocated interventions for a given trial participant (eg, drug dose change in response to harms, participant request, or improving/worsening disease)	19
	11c	Strategies to improve adherence to intervention protocols, and any procedures for monitoring adherence (eg, drug tablet return, laboratory tests)	19
	11d	Relevant concomitant care and interventions that are permitted or prohibited during the trial	19-20
Outcomes	12	Primary, secondary, and other outcomes, including the specific measurement variable (eg, systolic blood pressure), analysis metric (eg, change from baseline, final value, time to event), method of aggregation (eg, median, proportion), and time point for each outcome. Explanation of the clinical relevance of chosen efficacy and harm outcomes is strongly recommended	20-23
Participant timeline	13	Time schedule of enrolment, interventions (including any run-ins and washouts), assessments, and visits for participants. A schematic diagram is highly recommended (see Figure)	23-24
Sample size	14	Estimated number of participants needed to achieve study objectives and how it was determined, including clinical and statistical assumptions supporting any sample size calculations	25-26
Recruitment	15	Strategies for achieving adequate participant enrolment to reach target sample size	26

Methods: Assignment of interventions (for controlled trials)

Allocation:

For peer review only - http://bmjopen.bmj.com/site/about/guidelines.xhtml

Sequence generation	16a	Method of generating the allocation sequence (eg, computer-generated random numbers), and list of any factors for stratification. To reduce predictability of a random sequence, details of any planned restriction (eg, blocking) should be provided in a separate document that is unavailable to those who enrol participants or assign interventions	28-29
Allocation concealment mechanism	16b	Mechanism of implementing the allocation sequence (eg, central telephone; sequentially numbered, opaque, sealed envelopes), describing any steps to conceal the sequence until interventions are assigned	29
Implementation	16c	Who will generate the allocation sequence, who will enrol participants, and who will assign participants to interventions	29
Blinding (masking)	17a	Who will be blinded after assignment to interventions (eg, trial participants, care providers, outcome assessors, data analysts), and how	29-30
	17b	If blinded, circumstances under which unblinding is permissible, and procedure for revealing a participant's allocated intervention during the trial	30

Methods: Data collection, management, and analysis

Data collection methods	18a	Plans for assessment and collection of outcome, baseline, and other trial data, including any related processes to promote data quality (eg, duplicate measurements, training of assessors) and a description of study instruments (eg, questionnaires, laboratory tests) along with their reliability and validity, if known. Reference to where data collection forms can be found, if not in the protocol	30-31
	18b	Plans to promote participant retention and complete follow-up, including list of any outcome data to be collected for participants who discontinue or deviate from intervention protocols	31-32
Data management	19	Plans for data entry, coding, security, and storage, including any related processes to promote data quality (eg, double data entry; range checks for data values). Reference to where details of data management procedures can be found, if not in the protocol	32-34
Statistical methods	20a	Statistical methods for analysing primary and secondary outcomes. Reference to where other details of the statistical analysis plan can be found, if not in the protocol	36-37
	20b	Methods for any additional analyses (eg, subgroup and adjusted analyses)	37-38

For peer review only - http://bmjopen.bmj.com/site/about/guidelines.xhtml

	20c	Definition of analysis population relating to protocol non-adherence (eg, as randomised analysis), and any statistical methods to handle missing data (eg, multiple imputation)	38
**Methods: Monitoring**			
Data monitoring	21a	Composition of data monitoring committee (DMC); summary of its role and reporting structure; statement of whether it is independent from the sponsor and competing interests; and reference to where further details about its charter can be found, if not in the protocol. Alternatively, an explanation of why a DMC is not needed	40
	21b	Description of any interim analyses and stopping guidelines, including who will have access to these interim results and make the final decision to terminate the trial	38
Harms	22	Plans for collecting, assessing, reporting, and managing solicited and spontaneously reported adverse events and other unintended effects of trial interventions or trial conduct	41
Auditing	23	Frequency and procedures for auditing trial conduct, if any, and whether the process will be independent from investigators and the sponsor	39-40
**Ethics and dissemination**			
Research ethics approval	24	Plans for seeking research ethics committee/institutional review board (REC/IRB) approval	2, 44
Protocol amendments	25	Plans for communicating important protocol modifications (eg, changes to eligibility criteria, outcomes, analyses) to relevant parties (eg, investigators, REC/IRBs, trial participants, trial registries, journals, regulators)	42-44
Consent or assent	26a	Who will obtain informed consent or assent from potential trial participants or authorised surrogates, and how (see Item 32)	27-28
	26b	Additional consent provisions for collection and use of participant data and biological specimens in ancillary studies, if applicable	28
Confidentiality	27	How personal information about potential and enrolled participants will be collected, shared, and maintained in order to protect confidentiality before, during, and after the trial	34-35
Declaration of interests	28	Financial and other competing interests for principal investigators for the overall trial and each study site	50
Access to data	29	Statement of who will have access to the final trial dataset, and disclosure of contractual agreements that limit such access for investigators	35

For peer review only - http://bmjopen.bmj.com/site/about/guidelines.xhtml

Ancillary and post-trial care	30	Provisions, if any, for ancillary and post-trial care, and for compensation to those who suffer harm from trial participation	41-42
Dissemination policy	31a	Plans for investigators and sponsor to communicate trial results to participants, healthcare professionals, the public, and other relevant groups (eg, via publication, reporting in results databases, or other data sharing arrangements), including any publication restrictions	44-45
	31b	Authorship eligibility guidelines and any intended use of professional writers	49-50
	31c	Plans, if any, for granting public access to the full protocol, participant-level dataset, and statistical code	35
**Appendices**			
Informed consent materials	32	Model consent form and other related documentation given to participants and authorised surrogates	Supplemental appendix 2
Biological specimens	33	Plans for collection, laboratory evaluation, and storage of biological specimens for genetic or molecular analysis in the current trial and for future use in ancillary studies, if applicable	N/A

4

For peer review only

For peer review only - http://bmjopen.bmj.com/site/about/guidelines.xhtml

TIDieR checklist

Item number	Item	Where located ** Primary paper (page or appendix number)	Other † (details)
	**BRIEF NAME**		
**1.**	Provide the name or a phrase that describes the intervention.	15	
	**WHY**		
**2.**	Describe any rationale, theory, or goal of the elements essential to the intervention.	15-16 and Supplemental appendix 1	https://doi.org/10.3310/phr08040 (28)
	**WHAT**		
**3.**	Materials: Describe any physical or informational materials used in the intervention, including those provided to participants or used in intervention delivery or in training of intervention providers. Provide information on where the materials can be accessed (e.g. online appendix, URL).	16-17 and Supplemental appendix 1	______________
**4.**	Procedures: Describe each of the procedures, activities, and/or processes used in the intervention, including any enabling or support activities.	16-17	______________
	**WHO PROVIDED**		
**5.**	For each category of intervention provider (e.g. psychologist, nursing assistant), describe their expertise, background and any specific training given.	17	______________
	**HOW**		
**6.**	Describe the modes of delivery (e.g. face-to-face or by some other mechanism, such as internet or telephone) of the intervention and whether it was provided individually or in a group.	17	______________
	**WHERE**		
**7.**	Describe the type(s) of location(s) where the intervention occurred, including any necessary infrastructure or relevant features.	17	______________
	**WHEN and HOW MUCH**		
**8.**	Describe the number of times the intervention was delivered and over what period of time including the number of sessions, their schedule, and their duration, intensity or dose.	18	https://doi.org/10.3310/phr08040 (28)

For peer review only

	**TAILORING**		
**9.**	If the intervention was planned to be personalised, titrated or adapted, then describe what, why, when, and how.	15-18	______
	**MODIFICATIONS**		
**10.‡**	If the intervention was modified during the course of the study, describe the changes (what, why, when, and how).	N/A	______
	**HOW WELL**		
**11.**	Planned: If intervention adherence or fidelity was assessed, describe how and by whom, and if any strategies were used to maintain or improve fidelity, describe them.	19	______
**12.‡**	Actual: If intervention adherence or fidelity was assessed, describe the extent to which the intervention was delivered as planned.	N/A	______

** **Authors** - use N/A if an item is not applicable for the intervention being described. **Reviewers** – use '?' if information about the element is not reported/not sufficiently reported.

† If the information is not provided in the primary paper, give details of where this information is available. This may include locations such as a published protocol or other published papers (provide citation details) or a website (provide the URL).

‡ If completing the TIDieR checklist for a protocol, these items are not relevant to the protocol and cannot be described until the study is complete.

For peer review only

BMJ Open

Effectiveness and cost effectiveness of a 12 month automated text message intervention for weight management in postpartum women with overweight or obesity: protocol for the Supporting MumS (SMS) multi-site, parallel-group, randomised controlled trial.

Journal:	*BMJ Open*
Manuscript ID	bmjopen-2024-084075.R1
Article Type:	Protocol
Date Submitted by the Author:	24-Mar-2024

Complete List of Authors:	Gallagher, Dunla; Queen's University Belfast, Centre for Public Health<br>Spyreli, Eleni; Queen's University Belfast, Centre for Public Health<br>Anderson, Annie; University of Dundee, CPHNR<br>Bridges, Sally; Bradford Institute for Health Research<br>Cardwell, Christopher; Queen's University Belfast, Centre for Public Health<br>Coulman, Elinor ; Cardiff University School of Medicine, Centre for Trials Research (CTR)<br>Dombrowski, Stephan; University of New Brunswick Fredericton, Kinesiology<br>Free, Caroline; London School of Hygiene & Tropical Medicine, Public Health interventions Unit<br>Heaney, Suzie; Queen's University Belfast, Centre for Public Health<br>Hoddinott, Pat; University of Stirling, Nursing, Midwifery and Allied Health Professional Research Unit<br>Kee, Frank; Queen's University Belfast, Centre for Public Health<br>McDowell, Clíona; Northern Ireland Clinical Trials Unit<br>McIntosh, Emma; University of Glasgow Institute of Health and Wellbeing, Health Economics and Health Technology Assessment (HEHTA)<br>Woodside, Jayne; Queen's University Belfast, Centre for Public Health<br>McKinley, Michelle C.; Queen's University Belfast, Centre for Public Health

**Primary Subject Heading**:	Public health
Secondary Subject Heading:	Health economics, Qualitative research, Nutrition and metabolism, Mental health
Keywords:	Obesity, Randomized Controlled Trial, Postpartum Women < Postpartum Period, Behavior, HEALTH ECONOMICS, QUALITATIVE RESEARCH

SCHOLARONE™
Manuscripts

Effectiveness and cost effectiveness of a 12 month automated text message intervention for weight management in postpartum women with overweight or obesity: protocol for the Supporting MumS (SMS) multi-site, parallel-group, randomised controlled trial.

Dunla Gallagher, Eleni Spyreli, Annie S Anderson, Sally Bridges, Chris Cardwell, Elinor Coulman, Stephan U Dombrowski, Caroline Free, Suzie Heaney, Pat Hoddinott, Frank Kee, Cliona McDowell, Emma McIntosh, Jayne V Woodside and Michelle C McKinley.

Correspondence to Prof Michelle McKinley; m.mckinley@qub.ac.uk

Author Affiliations [5a]: Dr Dunla Gallagher; Dr Eleni Spyreli; Prof Chris Cardwell; Dr Suzie Heaney; Prof Frank Kee; Prof Jayne V Woodside; Prof Michelle C McKinley* (Centre for Public Health, Queen's University Belfast). Prof Annie S Anderson (Division of Population Health and Genomics, University of Dundee). Ms Sally Bridges** (Better Start Bradford Innovation Hub, Bradford Teaching Hospitals NHS Foundation Trust). Dr Elinor Coulman** (Centre for Trials Research, Cardiff University). Associate Prof Stephan U Dombrowski (Faculty of Kinesiology, University of New Brunswick). Prof Caroline Free** (Maternal, Adolescent, Reproductive & Child Health, London School of Hygiene & Tropical Medicine). Prof Pat Hoddinott** (NMAHP Research Unit, University of Stirling). Ms Cliona McDowell (Northern Ireland Clinical Trials Unit). Prof Emma McIntosh (Institute of Health and Wellbeing, University of Glasgow). (*Chief Investigator; **Participating Site Lead).

For peer review only

Abstract

Introduction

The reproductive years can increase women's weight-related risk. Evidence for effective postpartum weight management interventions is lacking and engaging women during this life stage is challenging. Following a promising pilot evaluation of the Supporting MumS intervention, we assess if theory-based and bidirectional text messages to support diet and physical activity behaviour change for weight loss and weight loss maintenance, are effective and cost-effective for weight change in postpartum women with overweight or obesity, compared with an active control arm receiving text messages on child health and development.

Methods and analysis

Two-arm, parallel group, assessor-blind randomised controlled trial with cost-effectiveness and process evaluations. Women (n=888) with body mass index ≥25 kg/m^2 and within 24 months of giving birth, recruited via community and NHS pathways through five United Kingdom sites targeting areas of ethnic and socioeconomic diversity. Women are 1:1 randomised to the intervention or active control groups, each receiving automated text messages for 12 months. Data is collected at 0, 6, 12 and 24 months. The primary outcome is weight change at 12 months from baseline, compared between-groups. Secondary outcomes include weight change (24 months) and waist circumference (cm), proportional weight gain (>5kg), BMI (kg/m2), dietary intake, physical activity, infant feeding and mental health (12 and 24 months respectively). Economic evaluation examines health service usage and personal expenditure, health-related quality of life and capability wellbeing to assess cost-effectiveness over the trial and modelled lifetime. Cost-utility analysis examines cost per quality-adjusted life years gained over 24 months. Mixed method process evaluation

For peer review only - http://bmjopen.bmj.com/site/about/guidelines.xhtml

explores participants' experiences and contextual factors impacting outcomes and implementation. Stakeholder interviews examine scale-up and implementation.

Ethics and dissemination

Ethical approval obtained before data collection (West of Scotland Research Ethics Service REC 4 22/WS/0003). Results will be published via a range of outputs and audiences.

Trial Registration [2a]: ISRCTN16299220 (prospectively).

For peer review only - http://bmjopen.bmj.com/site/about/guidelines.xhtml

Strengths and Limitations

- Trial measures will examine effectiveness outcomes of the intervention, along with cost-effectiveness and process evaluation.
- Including five trial sites across the four UK countries and using a variety of recruitment pathways, are designed to target and recruit a socioeconomically and ethnically diverse population of postpartum women.
- Home visits by the research team are offered to participants for data collection to facilitate participation in research assessments, helping to overcome barriers to taking part and address issues in participant retention seen in other postpartum trials.
- Researchers conducting participant recruitment and outcome data collection are blind to group allocation, however, due to the nature of the intervention it is not possible to blind participants to randomisation.
- The trial design includes an active control comparator to minimise disappointment bias and attrition related to randomisation experienced in previous weight loss intervention trials.

For peer review only

For peer review only - http://bmjopen.bmj.com/site/about/guidelines.xhtml

Administrative information

Title [1]: Effectiveness and cost effectiveness of a 12 month automated text message intervention for weight management in postpartum women with overweight or obesity: protocol for the Supporting MumS (SMS) multi-site, parallel-group, randomised controlled trial.

Protocol version [3]: Version 3.0 dated 10th May 2023.

Trial status: Opened to recruitment in April 2022, with the first participant randomised in May 2022 and participant recruitment completed in May 2023. Primary outcome data collection to be completed in May 2024.

Introduction

Background [6a]

This trial addresses the development of overweight and obesity across the childbearing years. Entering pregnancy with a high body mass index (BMI) increases many health risks for these mothers and their babies, including gestational diabetes, hypertensive disorders, caesarean section and birth complications, compared with recommended weight women.(1, 2, 3, 4) Excessive gestational weight gain (GWG) and postpartum weight retention (PPWR) are common (5, 6) and many women are at risk of further weight gain across the extended postpartum period.(7) By 18 months postpartum, about one in five women have moved into a higher BMI category (6) which increases the risk of complications in subsequent pregnancies and contributes to long-term overweight and obesity.(7)

Effective and appropriate weight management interventions in women during the postpartum period, which account for the well-recognised barriers for new mums such as time constraints and childcare, are lacking.(7, 8, 9, 10, 11, 12) Previous intervention studies are characterised by poor recruitment and high rates of attrition (13, 14, 15, 16, 17) and have not

For peer review only - http://bmjopen.bmj.com/site/about/guidelines.xhtml

adequately considered the difficulties in reaching this population and specific barriers to lifestyle behaviour change that come with having a baby.(18, 19, 20, 21, 22) In-person and structured weight management approaches, such as those delivered in community and group settings, are unlikely to be successful with this population due to the commitment required, and such approaches may exacerbate health inequalities.(23, 24, 25, 26) More appropriate ways of recruiting and engaging with postpartum women to achieve sustained behaviour change are required (7, 13) and a need to evidence cost-effectiveness to inform implementation.(10)

Recent evidence, mostly from feasibility or pilot studies, indicates that technology usage shows promise in postpartum weight management, perhaps because this form of delivery fits more seamlessly with women's lives at this stage.(16, 27, 28, 29) Mobile technologies can offer a more flexible and individualised 'any time, any place' approach to behavioural weight management interventions.(7) Text message interventions have high reach potential and allow for flexible scheduling and interactivity,(30) potentially making them convenient for mums with limited time and helpful for overcoming health inequalities. Systematic reviews support the use of text messaging in behavioural weight management interventions but indicate further evaluation is required.(31, 32, 33, 34, 35). They are cost-effective and readily scalable for changing other health behaviours.(36, 37)

The Supporting MumS (SMS) intervention, consisting of a library of text messages, was developed with personal and public involvement (PPI) to support weight management in the postpartum period.(27) Messages focus on diet and physical activity with embedded behaviour change techniques (BCTs) informed by behaviour change theory and evidence. In a previous pilot evaluation, one hundred women with overweight or obesity from Northern Ireland (NI), who had a baby within the previous 24 months, were recruited and randomised to receive text messages about weight loss (intervention group) or child health and development (active control) for 12 months. The study demonstrated the feasibility and

For peer review only - http://bmjopen.bmj.com/site/about/guidelines.xhtml

acceptability of the intervention and an independent Trial Steering Committee (TSC) judged that the pre-specified progression criteria to proceed to a full randomised controlled trial (RCT) (i.e., successful recruitment, high retention and no differential attrition, high acceptability of the intervention, and evidence of positive indicative effects) were met.(27)

Rationale [6a]

This RCT addresses the following research question: Is an automated 12 month text message intervention, designed to support weight loss and weight loss maintenance for postpartum women with overweight or obesity, effective and cost-effective for weight loss at 12 months, compared with an active control group receiving text messages on child health and development?

SMS is a parallel, 2-arm multi-site RCT using automated and bidirectional text messages to support weight management over a 12 month period in postpartum women with overweight or obesity who have had a baby in the last 24 months, comparing weight change at 12 months from baseline between the intervention and active control groups. This RCT protocol is based on the pilot RCT methodology, but changes were to remove: 1) use of a sealed pedometer to assess physical activity as this was not acceptable to women and, 2) a discussion forum intervention component as it was not used by pilot RCT participants. We added considerations for data collection in the event of COVID-19 restrictions. In moving from a single site pilot study to a multi-site RCT recruiting an ethnically and socioeconomically representative sample, we conducted a six-month period of intervention and recruitment method adaptation, with PPI, ensuring the broad acceptability and cultural relevance of the messages and enhancing the generalisability of the findings to different groups of women across the United Kingdom (UK), thus informing implementation.

The increased prevalence of obesity, its associated complications and weight management services, is costly to the National Health Service (NHS). NHS costs associated with caring

For peer review only - http://bmjopen.bmj.com/site/about/guidelines.xhtml

for a pregnant woman with a BMI of ≥25 kg/m^2 are up to 37% higher than caring for a woman with a healthy BMI.(38) Both National Institute for Health and Care Excellence (NICE) guidance (11) and an associated review of the cost-effectiveness of weight management interventions following childbirth,(39) outline the potential of postpartum interventions to be a cost-effective way of reducing the long-term risks of obesity, heart disease, cancer and diabetes, and recommend health professionals should advise women within two years of having a baby to eat a nutritious diet and keep active to encourage postpartum weight reduction.(11, 40)

Postpartum weight management interventions combining dietary and activity behaviour change have moderate positive influence on maternal weight (10, 14) and those including self-regulatory BCTs are likely to be more successful.(8, 10) The postpartum period could be an ideal time to intervene to shape new health behaviours as women may be motivated and receptive to health information.(41) However, women highlight a need for additional weight management support during the postpartum period as little is currently provided.(42)

The present trial allows women to take part up to 24 months postpartum. Qualitative evidence suggests that every woman's postpartum journey is different (19, 20, 21, 22, 23) and the optimal time to engage women in postpartum weight management is currently unknown.(9, 11) Allowing this extended inclusion window accommodates different maternal weight trajectories across this time.(43, 44) Furthermore, women may prepare for another pregnancy during this period, so there is potential to support better health for subsequent pregnancies.(7)

Mobile phone usage and ownership are widespread (95% of UK households) amongst all sectors of society, irrespective of socioeconomic status.(31) Text messaging is a simple communication mode that does not require smartphone or tablet technology, unlike web-based interventions or apps. This supports health equity through high reach potential at a low cost, making it conducive with scale-up and implementation, unlike some weight loss

For peer review only - http://bmjopen.bmj.com/site/about/guidelines.xhtml

interventions which can be resource intensive and expensive (45) and can exacerbate health inequalities.(46, 47) A text messaging intervention can be delivered flexibly and on a sustained basis, can be reactive and proactive, and does not necessarily rely on participant initiation.(30, 48) Using the real-time advantages offered by mobile technologies to deliver behavioural weight management support to postpartum women has the potential to encourage behaviours that may improve maternal health in both the short and long-term.(49) Trial results are relevant for other population groups given the possible advantages of this weight management approach. The NHS aims to embrace the potential of digital strategies for improving UK health (50) and text message interventions could complement any weight management support currently delivered across the NHS, to help ease the current burden on an over-stretched service.

The SMS trial adopts a fully automated approach to intervention delivery whilst still offering feedback to participants. The trial makes a novel and important contribution to the field of behavioural text message interventions in a number of ways: there are currently few such interventions that are fully automated, where text messaging is the main mode of delivery and two-way messaging is used to encourage engagement and delivery of specific BCTs; there are also few which consider both weight loss and weight loss maintenance, last 12 months or more and use an active control to minimise disappointment bias.(27)

The National Institute of Health and Care Research (NIHR) awarded further funding to explore postnatal mental health outcomes and needs to inform application of the priorities in the NHS Long Term Plan via existing studies. We added data collection methods related to mental health (postnatal depression, depression and anxiety, mother and child relationships, service and treatment experiences) in February 2022, allowing an opportunity to examine poor postnatal mental health prevalence rates, trajectories, predictors, inequalities and service needs, in an ethnically and socioeconomically diverse sample from all four UK countries. The trial also allows further validity and reliability testing of the Me and My Baby

For peer review only - http://bmjopen.bmj.com/site/about/guidelines.xhtml

(MaMB)/Me and My Child (MaMC) screening tool (51) which was co-produced by health visitors and the community to assess the mother and child relationship. Funding for this workstream was contracted in March 2022.

Explanation for the choice of comparators [6b]

An active control comparator is used as pilot study findings indicate that this design aspect supports good participant retention, acceptability and satisfaction, where previous weight loss trials with postpartum women have reported high levels of attrition and disappointment bias in those allocated to no treatment or usual care control groups.(13) Active control participants are assessed for the same outcome measures as the intervention group at 6, 12 and 24 months. We advertise the purpose of the trial as testing two new text message services to minimise women's preference for one group over another. The participant information sheet (PIS) clearly describes that women have a 50:50 chance of being assigned to receive messages about child health and development or messages about weight management, thus preserving participant autonomy.

Objectives [7]

Primary objective

To conduct a 2-arm parallel-group RCT comparing weight change at 12 months for postpartum women with overweight or obesity who receive text messages about weight management with an active control.

Secondary objectives

- To assess differences between-groups in secondary outcomes.
- To assess the cost-effectiveness of the SMS intervention compared with an active control comparator.

For peer review only - http://bmjopen.bmj.com/site/about/guidelines.xhtml

- To assess the prevalence and trajectories of postnatal mental health in women across the UK, particularly those from marginalised groups, including assessment of the mother-child relationship.
- To conduct a process evaluation to explore women's experiences of the intervention, the pathways through which the intervention effects are mediated and contextual factors affecting the outcomes or future implementation of the intervention.
- To seek permission for routine data linkage for long term health outcomes (mother and youngest child).
- To conduct a follow-up of women at 24 months (12 months after the intervention has ceased) to examine the long-term effect of the intervention and to conduct interviews with stakeholders to explore scale-up and implementation; this stage is contingent upon TSC and NIHR assessment of the 12 month primary outcome data and any other core outcome data required to make a fully informed decision about effectiveness and the clinical significance of weight change.

Trial Design [8]

A pragmatic, multi-site, parallel, 2-arm, assessor-blind, 1:1 superiority RCT comparing weight change at 12 months for the SMS intervention and active control groups, with mixed methods process evaluation, cost-effectiveness modelling, consent for future data linkage to longer-term health outcomes for mothers and babies, 24 month participant follow-up (i.e. 12 months post-intervention) and implementation consultations with stakeholders.

Methods and analysis

This protocol is written in line with the SPIRIT checklist,(52) with recommended content indicated by the numbers in square brackets after section titles.

For peer review only - http://bmjopen.bmj.com/site/about/guidelines.xhtml

Trial setting [9]

The trial will run between July 2021 and April 2025 with a planned 12 month recruitment period starting in April 2022. A multi-site trial with five recruitment sites in the UK: 1) Belfast, Northern Ireland (trial co-ordinating site); 2) Stirling, Scotland; 3) Cardiff, Wales; 4) London, England; and 5) Bradford, England. Recruitment sites were selected to target geographical areas of ethnic and socioeconomic diversity for the purposes of participant recruitment via community and NHS pathways.

Eligibility criteria [10]

Women (in accordance with the NICE Postnatal care guideline NG1941, the term 'woman' used in this trial includes people who do not identify as women but who are pregnant or have given birth) are eligible to participate in the trial if they are aged over 18 years, have a BMI of ≥25 kg/m^2 and have had a baby within the last two years.

Women are excluded if their baby is less than 6 weeks old, if they have no access to a mobile phone to allow them to receive personal text messages or if they have insufficient English language to understand short written messages, if they have had or plan to have any type of bariatric surgery, if they report having ever received a diagnosis of anorexia nervosa or bulimia from a doctor, or, if they are on a specialist diet and receiving dietetic care. Women are excluded if they are participating in any other weight management research study/programme currently or in the previous three months. Women are not eligible if they are pregnant and any consented participant becoming pregnant during the trial is excluded, as the intervention has not been designed for use in pregnancy.

Interventions

For peer review only - http://bmjopen.bmj.com/site/about/guidelines.xhtml

Supporting MumS (SMS) intervention [11a]

A full description of the SMS intervention development has been reported elsewhere.(27) The intervention is described here in accordance with the Template for Intervention Description and Replication (TIDieR) guidance.(53) The intervention group receive fully automated text messages, including bidirectional and interactive features, about weight loss and maintenance of weight loss for 12 months. The evidence and theory-based intervention consists of a library of text messages focused on diet and physical activity with embedded BCTs known to be positively associated with weight management. The intervention logic model and sample intervention and active control text messages are available in supplemental appendix 1. Examples of how BCTs were incorporated into the messages is available elsewhere.(27)

Why- theory and components

The intervention is based on the Health Action Process Approach (HAPA) and a systematic review of over 100 behavioural theories which synthesised theoretical explanations for maintenance of behaviour change.(54) The intervention encourages a self-guided approach to weight-related behaviour change, as supported by the literature.(55, 56, 57, 58) The intervention focuses on dietary intake and physical activity to address energy balance related behaviour, with embedded evidence-based BCTs specifically linked to the relevant phases and psychological processes of behaviour change alongside consideration of barriers for this population.(27)

The messages adopt a friendly tone including humour to foster engagement and provide information, practical tips and advice including quotes from other mothers. They signpost to external resources and provide encouragement and motivation, discourage guilt and promote self-reflection. A core library of text messages (n= 353) to be delivered by the automated system was created. These include a weekly text message asking women to self-weigh and report their weight (n=50) and bidirectional messages to encourage engagement, self-

For peer review only - http://bmjopen.bmj.com/site/about/guidelines.xhtml

monitoring, relapse prevention and to provide feedback, as follows: 1) “Yes/No” questions (n=36) which trigger an automated response to the participant based on their reply, 2) trigger words (‘slip-up’; ‘crave’; ‘bad day’ or ‘tired’) which can be texted by women at any time to prompt a reply designed to address these barriers and prevent relapses, 3) During months 7-12 when the focus shifts to maintenance strategies, the weekly text message asking women to report their weight asks them to add the word ‘up’ or ‘down’ or ‘same’ in relation to how their weight compares with the week prior. These keywords trigger an automated response. Responses for bidirectional messages are sent from pre-loaded message banks and are not individually scripted or tailored.

Women can also opt-in to receive messages related to weight management when breastfeeding (n=10) or stopping smoking (n=15). These messages are intended to alleviate participant concerns in relation to weight management whilst breastfeeding and to address vulnerabilities around weight gain during smoking cessation.

What- materials and procedures

The intervention consists of 353 core messages delivered to all women allocated to this group, in addition to messages associated with the interactive and optional components described above.

Social support is facilitated throughout the intervention using a “buddy system” (informed by a successful smoking cessation text message intervention (37)) where a participant can nominate a friend or family member to receive the same messages they are receiving, putting that person in a better position to offer support. Participants are sent instructions and reminders on how to do this within the core messages, along with information on the value that support can play in weight loss success. A ‘buddy’ can be requested at any stage throughout the intervention period.

For peer review only - http://bmjopen.bmj.com/site/about/guidelines.xhtml

Who- intervention provider

Intervention costs are provided by the NI Public Health Agency (PHA). Intervention delivery is fully automated via the existing pilot study text message platform developed by the London School of Hygiene and Tropical Medicine (LSHTM). The intervention package is designed so that it can be readily delivered by other text message providers to facilitate future implementation by the NHS or public services.

How - mode of delivery

Message exchange with participants is managed via a secure server at the LSHTM, using Firetext (59) to distribute messages to participants.

Where– setting

The intervention can occur any place and any time. Participants require a mobile phone with the function to receive text messages.

When and how much

Text message frequency starts at 14-15 sent per week during months one and two, tapering to 9-10 per week in months three to six. Fewer messages, four to five per week, are sent in the weight loss maintenance phase (months 7 to 12) when the emphasis shifts to reinforcing self-regulation techniques developed during the first six months of the intervention and encouraging maintenance relevant strategies such as relapse prevention.

The system delivers messages any time between 10am and 11pm, with messages programmed to arrive at varied times to avoid predictability. Participants can choose to block

For peer review only - http://bmjopen.bmj.com/site/about/guidelines.xhtml

For peer review only

an additional period of the day that they want to be kept message-free e.g., 10am to 5pm. Following randomisation, women allocated to the intervention group are programmed to start receiving messages on the subsequent Monday which schedules the weekly message asking participants to report their weight to be sent on Fridays.

Active control [11a]

The active control messages were also developed by the trial team with PPI input (27), for mothers with babies aged six weeks up to 36 months (based on participant inclusion criterion). The messages relate to general childcare and development, with content consistent with evidence-based information provided by the NHS Start4life service (60) including weekly play ideas/activities and information on milestones, home safety, separation anxiety and similar topics. Active control messages do not address the intervention target or active ingredients (i.e. diet, physical activity and BCT content related to weight loss) nor do they prompt bidirectional functionality. Women receive three messages each week over 12 months with messages corresponding to the age of their baby, i.e., if their baby is six months old at randomisation, the participant starts to receive messages corresponding to this age. Women allocated to the control group are offered to receive a booklet summarising the intervention content on completion of the trial.

Criteria for discontinuing or modifying allocated interventions [11b]

Participants in both groups have the ability to pause, stop and restart the messages throughout by sending an instruction to the text system e.g., ‘STOP’, ‘START’. Researchers ask women choosing to discontinue the messages to provide any reason for doing so, to

For peer review only - http://bmjopen.bmj.com/site/about/guidelines.xhtml

inform the process evaluation. Participants discontinuing messages remain in the trial unless they formally withdraw from the trial assessments.

Strategies to improve adherence to interventions [11c]

Text message delivery is fully automated and centrally delivered via the LSHTM system which is intended to ensure treatment fidelity. Regular monitoring of the text message system is completed by the system developer and trial team members (CI and clerical officer) to check the content of sent and received messages (e.g., ensure web links are working, review spontaneous participant responses). Intervention engagement is assessed via text messages received from participants in response to the weekly weight prompt and the replies to bidirectional messages as described in [11a], self-report items in questionnaires at 6, 12 and 24 months and qualitative interviews at 6 and 12 months.

Relevant concomitant care permitted or prohibited during the trial [11d]

Women who become pregnant during the trial will be excluded, as text message content has not been designed for pregnancy and primary outcome data (weight) can only be collected from women who are not pregnant. This is a self-directed intervention and women are permitted to access any other weight-loss services via NHS, voluntary or private sector pathways. Details of engagement with other services are captured at each data collection timepoint. Any weight loss surgery undergone by participants during the trial, and prescribed or over-the-counter medications that may affect weight, are documented at 6, 12 and 24 months to assess the impact on trial outcomes.

For peer review only - http://bmjopen.bmj.com/site/about/guidelines.xhtml

Outcomes [12]

Trial outcomes, measures and the assessment schedule at 0, 6, 12 and 24 months are outlined in Table 1.

- *Primary outcome:* between-group difference in mean weight change (kg) from baseline to 12 months.
- *Secondary outcomes:* between-groups difference in mean weight change (kg) from baseline to 24 months and between-groups differences in mean waist circumference (cm), mean BMI (kg/m^2), in the proportions of women gaining a substantial amount of weight (>5kg), dietary intake (Fat and Fibre Barometer plus questions on sugar intake), physical activity (IPAQ-SF) and infant feeding practices (Infant Feeding Survey), by the 12 and 24 month follow-up timepoints respectively.
- *Health economic outcomes:* NHS resource use and personal expenditure (GP usage, hospitalisations, prescribed and over-the-counter medications, lifestyle products and services, expenditure on food and drink, alcohol and smoking products); health-related quality of life (EQ-5D-5L with visual analogue scale) and Capability (ICECAP-A).
- *Process Evaluation:* to explore participants' experiences and interactions with the intervention by site, ethnicity and socioeconomic status, including evaluating dose (number of women who choose to receive all text messages and those who pause or stop the messages), engagement with the intervention (text message responses and qualitative data), reach (trial records of recruitment, loss to follow-up and withdrawal) and acceptability (participants' satisfaction ratings and qualitative data).
- *Exploratory outcomes:* moderator and mediator analysis (Table 1)
- *Qualitative sub-study:* telephone interviews with a purposive sample of participants at 6 and 12 months to understand the experiences of women receiving the intervention and active control text messages and the contextual factors related to engagement including postnatal mental health, to inform trial and process evaluation outcomes.

For peer review only - http://bmjopen.bmj.com/site/about/guidelines.xhtml

- *Stakeholder consultation:* if the intervention is shown to be effective at 12 months, stakeholder interviews will be conducted to explore factors relevant to intervention scalability and implementation, including any further work needed to develop a coherent implementation model.

For peer review only

For peer review only - http://bmjopen.bmj.com/site/about/guidelines.xhtml

427 **Table 1: Supporting MumS trial assessments**

Outcome collected	Measure/equipment used	Time point (month)			
		0	6	12	24
Anthropometric measures and Demographics*					
Height (m)	Portable standing stadiometer (Seca 213, Birmingham, UK)	✓			
Body weight (kg) (primary endpoint)	Calibrated scales (Seca 875, Birmingham, UK)	✓	✓	✓	✓
Waist circumference (cm)	Tape measure (Seca 201, Birmingham, UK)	✓	✓	✓	✓
Demographic characteristics**	Trial-specific questions	✓			
Acceptability outcomes					
Satisfaction with text messages¥	Trial-specific questions		✓	✓	
Acceptability of intervention, active control and study methods¥	Trial-specific questions			✓	
Longer-term use of text messages¥	Trial-specific questions				✓
Qualitative interviews	-		✓	✓	
Interviews with stakeholders	-				✓
Economic evaluation - Within trial health and social care cost data¥					
Health service resources use	Questions on health resource usage	✓	✓	✓	✓
Medication usage	Questions on over-the-counter medications	✓	✓	✓	✓
Healthy lifestyle-related costs	Questions on personal expenditure	✓	✓	✓	✓
Exercise-related costs	Questions on costs of exercise	✓	✓	✓	✓
Food and drink costs	Questions on weekly cost of groceries, alcohol and smoking products	✓	✓	✓	✓
Change of employment status	Trial-specific questions	✓	✓	✓	✓
Health-related quality of life	EQ-5D-5L (61)	✓	✓	✓	✓
Capability well-being	ICEpop CAPability measure Adults (ICECAP-A)(62)	✓	✓	✓	✓
Secondary outcome measures¥					
Dietary intake	Fat and fibre barometer(63)	✓	✓	✓	✓
Sugar intake	Trial-specific questions	✓	✓	✓	✓
Alcohol consumption	Trial-specific questions	✓	✓	✓	✓
Physical activity	International Physical Activity Questionnaire (IPAQ) - Short form(64)	✓	✓	✓	✓
Infant feeding	Infant feeding survey(65)	✓	✓	✓	✓
Moderators of intervention effect¥					
Mental health – depression	Edinburgh Postnatal Depression Scale (EPDS)(66)	✓	✓	✓	✓
Mental health - anxiety	Generalised Anxiety Disorder (GAD-7)(67)	✓	✓	✓	✓
Mother and child relationship	MaMB/MaMC(51) and Mothers Object Relations Scale (MORS-Baby/Child)(68)	✓	✓	✓	✓
Sleep	Pittsburgh Sleep Quality Index(69)	✓	✓	✓	✓
Confidence/importance/desire for weight loss and maintenance	Trial-specific questions	✓	✓	✓	✓
Mediators of intervention effect¥					

For peer review only - http://bmjopen.bmj.com/site/about/guidelines.xhtml

Intention and self-efficacy for diet and physical activity	Health Action Process Approach (HAPA) including action and coping planning and self-efficacy (70, 71)	✓	✓	✓	✓
Habit behaviour	Self-report behavioural automaticity index(72)	✓	✓	✓	✓
Self-regulation of eating behaviour	Self-regulation of eating behaviour questionnaire(73)	✓	✓	✓	✓
Weight self-monitoring	Trial-specific questions	✓	✓	✓	✓
Diet and exercise monitoring	Trial-specific questions	✓	✓	✓	✓
Goal-setting for diet and exercise	Trial-specific questions	✓	✓	✓	✓
Weight loss motivation	Motivation for weight loss scale(74)	✓	✓	✓	✓
Taking part in other weight loss programme	Trial-specific questions	✓	✓	✓	✓
Social support	Social support for eating & exercise(75) plus general social support (BiB trial-specific questions)	✓	✓	✓	✓
Self-esteem	Rosenberg Self-Esteem Scale(76)	✓	✓	✓	✓

**Recorded in case report form (CRF).*

***Date of birth, where they heard about the trial, NHS number, ethnicity, income, postcode, employment, education, relationship status, weeks postpartum at trial entry, parity, medical information (disability status, medication use), infant feeding, weight history, smoking status, alcohol intake, technology usage*

¥Recorded in participant self-reported questionnaires.

For peer re

Participant timeline [13]

The trial flowchart includes the participant timeline for the RCT (Figure 1).

Sample size [14]

The target sample size is 888 women over 12 months, with site-specific targets across the five geographical areas (London and Bradford, n=189, respectively; Belfast, Stirling and Cardiff, n=170, respectively). Pregnancy-related withdrawals are not replaced unless the loss to follow-up (12%) or pregnancy rates (15%) observed in the pilot RCT (27) are exceeded by the 6 month timepoint, which is monitored during Project Management Team (PMT) meetings.

Sample size calculation

The pilot trial showed a mean weight loss of 1.75kg in the intervention group compared with a mean gain of 0.19kg in the active control group between baseline and 12 months, equating to a between-groups difference in mean weight change, adjusted for baseline, of -1.67 kg (95% CI -4.88 to 1.55)).(27) Based on pilot data for the active control group and a standard deviation of 7.5kg, 594 completing participants (297 per group) are required to give the trial over 90% power to detect a statistically significant difference of 2kg in mean weight change from baseline, at the 5% level, between-groups. A mean difference of 2kg is accepted as being associated with metabolic health benefits and is often used to power weight loss studies.(58)

We observed a 15% pregnancy dropout rate in the pilot study, based on a 99% white ethnicity population.(27) Data from South Asian women in the Born in Bradford's Better Start (BiBBS) cohort (77) indicates that pregnancy dropout rates could be higher in more ethnically diverse samples, around 22%. To have 119 participants completing per site, and accounting for a loss to follow-up rate of 15% and higher anticipated pregnancy exclusion rates in areas with greater ethnic diversity, the following site-specific sample sizes were calculated: 1) Belfast, Stirling and Cardiff; 15% pregnancy rate + 15% loss to follow-up = 170 women, 2) Bradford and London; 22% pregnancy rate + 15% loss to follow-up= 189 women. Therefore, the total sample size for the trial is 888 women (444 per group).

Recruitment [15]

Based on successful pilot study recruitment processes,(27) trial information is disseminated via community settings likely to be attended by mothers of young children (e.g., parent and toddler groups, libraries, breastfeeding support groups), social media and through contacts with healthcare professionals and GP practices, including both routine appointments and

contacting potentially eligible women identified using patient database searches. A range of recruitment pathways are used to target ethnically and socioeconomically diverse populations.

In Bradford, principal trial recruitment is through BiBBS, an existing birth cohort study, with trial information disseminated to participants who previously consented to be informed of other relevant research studies.

Monitoring recruitment progress

Based on the pilot study recruitment rate,(27) we plan to recruit the target sample over a 12 month period. We employ a recommended traffic light system (Figure 1) to review trial progression at the mid-point of the recruitment period,(78) with the following actions: proceed if >50% of target achieved; modify recruitment approach if 25-49% achieved and consider stopping (discuss with funder and TSC) if <25% of target recruited.

Screening [10]

All trial information materials disseminated via described recruitment pathways, direct women who are potentially interested in taking part to contact the research team via telephone, email or by accessing the trial website/scanning a QR code to submit a secure pro-forma with their contact details.

Site researchers contact women expressing an interest in their area to provide a PIS, privacy notice and copy of the consent form (supplemental appendix 2) via email or post. Audio versions of the PIS and privacy notice are available on the trial website to increase accessibility (go.qub.ac.uk/SMSstudy). To comply with the Welsh Language Act 1993, Welsh language versions of the PIS and Consent Forms are available, on request. Women are given at least 48 hours to consider the information before being contacted again by telephone by the research team to discuss the trial procedures and any queries then invited to take part. Up

For peer review only - http://bmjopen.bmj.com/site/about/guidelines.xhtml

to three contact attempts are made to invite all women expressing interest for screening. BMI eligibility is initially determined using women's self-reported height and weight but later measured and verified by the researcher prior to collecting informed consent. Women who are deemed ineligible due to their baby being less than six weeks old will be invited for rescreening when they reach six weeks postpartum.

Informed consent and withdrawal [26a]

For women meeting the eligibility criteria and willing to proceed with trial participation, a baseline researcher visit is arranged. Home visits are the primary approach to data collection, with women also offered the option of attending another venue if preferred (University building, community venue or their workplace).

Research staff, trained in Good Clinical Practice (GCP) guidelines, obtain written informed consent prior to collecting baseline data. At the baseline visit, women are offered the opportunity to ask further questions and confidentiality of data is explained. Informed consent is then taken. Women are reminded that they have the freedom to choose whether to take part or not. If willing to proceed, women are asked to initial and sign the trial consent form, with optional consent to be invited for telephone interview participation at 6 and 12 months. During the 6 and 12 month trial visits, women providing this optional consent at baseline are asked if they are still happy to be contacted to take part in a telephone interview. Participants completing telephone interviews are asked to provide their verbal consent for audio recording and for anonymised quotations to be used in trial publications before commencing interviews.

Once consented, participants are advised that they can withdraw from the trial at any time, without giving a reason, should they wish. Withdrawals are documented in the CRF along with any reason given for withdrawing. Participants excluded due to pregnancy are given the

option to continue receiving the text messages. Anyone choosing this option needs to complete and return a pregnancy form to acknowledge that the messages have not been designed for pregnancy but may be useful after pregnancy. Data collected up to the point of withdrawal is retained for analysis unless the participant specifies otherwise.

Additional consent provisions for collection and use of participant data and biological specimens [26b]

Optional aspects of trial consent include permission to be informed of future follow-up to this trial or other relevant research studies and permission for future data linkage to routinely collected health records for long term health outcomes (mother and youngest child) which is not within the current trial timeline. No biological specimens are collected.

Assignment of interventions: allocation

Sequence generation [16a]

Participants are randomised in blocks of size 4 and randomisation is stratified by site, with the allocation sequence generated in STATA (using ralloc) by an independent statistician.

Concealment mechanism and allocation implementation [16b and 16c]

The allocation sequence is sent directly to the platform manager at LSHTM for upload to the secure web-based text message delivery system. After collecting consent and baseline data, site researchers enrol participants using the LSHTM randomisation system linked directly to the text message platform, implementing the random allocation sequence by accordingly

For peer review only - http://bmjopen.bmj.com/site/about/guidelines.xhtml

sending automated intervention or control messages to the participant. Participants become aware of their group allocation when they start to receive the text messages; researchers do not have access to this information.

Blinding [17a]

Researchers responsible for recruitment and collecting outcome data are blinded to group allocation. It is not possible to blind participants, but they are requested not to discuss the text messages they are receiving with researchers at each visit. This worked well in the pilot study with blinding maintained.(27) All statistical analyses are conducted blinded to group allocation until analyses of the 12 month data point are complete at which point the randomisation sequence is provided by the independent statistician who prepared it. We record any circumstances in which unblinding has occurred. The trial clerical officer, Chief Investigator (CI) and LSHTM text message system developer are not blind; these individuals see what group individuals are assigned to on the text message system for the purposes of monitoring the intervention delivery and the clerical officer is responsible for preparing the group-specific 12 month questionnaires. Blinded researchers do not see these questionnaires until after primary outcome data is collected. Interviews at 6 and 12 months are carried out by a researcher who has not had any previous contact with the participant. Group allocation information for that participant is provided to the researcher before the interview so the correct interview guide can be used.

Emergency unblinding [17b]

Given the nature of the intervention, emergency unblinding is not required.

For peer review only - http://bmjopen.bmj.com/site/about/guidelines.xhtml

Data collection, management and analysis

Data collection methods [18a]

The details and schedule of trial assessments (Table 1) are based on the pilot RCT.(27) Assessments take place at baseline (before randomisation), 6 months, 12 months (end of intervention period) and 24 months (12 months after intervention end).

Screening is completed according to the trial eligibility criteria and guided by and documented in a trial screening sheet.

Researchers, trained in data collection processes, collect outcome data during visits to women's homes (or another venue of the participant's choosing e.g., University/Community venue). Height, weight and waist circumference are measured by the researcher using standardised protocols and calibrated scales. A hard board is available to place under the scales on uneven surfaces. Baseline demographic information is collected by the researcher. Health service identification number is collected from any woman who provides consent for future data linkage. All remaining outcome data including health economic outcomes are collected via participant self-report questionnaires, either on paper (during the visit or a hard copy left with the participant along with a paid return envelope) or online (link to questionnaire hosted on Qualtrics survey software sent to participant and managed by the trial coordinating team in Queen's University Belfast (QUB)), based on participant preference at each visit. Copies of the baseline and 12 month questionnaires are available in supplemental appendix 3. Researchers offer any required assistance with paper questionnaire completion at the visit or via telephone.

Interviews are conducted with approximately 50 women at each timepoint, with participants purposively sampled to include ethnic, geographical and socio-demographic diversity (i.e., including a spread across trial sites, ethnicity, income level, parity, mental health status, randomised groups and intervention engagement e.g., those who have discontinued

messages). Interviews follow a semi-structured interview guide (available in supplemental appendix 4) and a translator can be used when any woman would prefer to be interviewed in a language other than English.

Text message engagement data is captured by the LSHTM system, including all messages sent and received organised by participant ID and date.

If the intervention is shown to be effective at 12 months, we will invite a range of relevant stakeholders (e.g., midwives, GPs, PPI, commissioners, policymakers) identified from research team and TSC networks to take part in interviews to explore factors relevant to engagement, scale-up and implementation.

Qualitative data is collected from participants and stakeholders using telephone/Microsoft teams interviews, which are audio-recorded and transcribed.

Plans to promote participant retention and complete follow-up [18b]

Home visits are offered for data collection to facilitate women's participation in research assessments, although visits can take place elsewhere if preferred by the participant. Visits are flexibly arranged for a date and time of convenience for the participant. At the end of each visit, the appointment for the next visit is scheduled if possible. Participants are sent reminders about scheduled visits at one month, one week and one day before the scheduled date and time. We attempt to collect data in a 2-week window either side of the follow-up due date i.e., date ±2 weeks, but allow flexibility outside of this window when women are actively engaging with the research team to arrange the visit. Participants' preferred methods of contact are documented and prioritised. Three contact attempts using different methods e.g., text, phone, email, are made to all women to confirm scheduled visits and following non-attendance to reschedule. Women who are unable to complete a visit with the

researcher at 6 months are sent a link to complete the questionnaire (via Qualtrics email link). As a last resort, when all attempts to arrange a face-to-face visit at 12 (primary endpoint) and 24 months are unsuccessful, participants are asked to provide self-reported weight and complete a questionnaire (via Qualtrics email link). Participants are sent a £25 voucher at each trial timepoint (0, 6, 12 and 24 months; £100 maximum over 24 months) as a token of appreciation for the time committed to completing trial assessments. Participants lost to follow-up at one trial timepoint but who have not withdrawn, are invited to attend future visits.

To maximise data completeness, site researchers follow-up on any non-returned questionnaires and check all received data forms/e-forms to document data queries (e.g., missing data, invalid ranges etc) and any attempts made to resolve them e.g. clarify responses with the participant, using a data query log.

Data Management [19]

The hard copy of the CRF which records data at each timepoint including weight is the source data and requires sign off by the completing researcher. For questionnaires, the hard copy or electronic record is considered the source data depending on mode of completion.

The Northern Ireland Clinical Trials Unit (NICTU) are responsible for developing and managing trial databases on validated and secure systems that back-up data, maintain audit trails of data changes and only allow password-protected access to designated trained team members. Screening and CRF data are entered into trial databases by site researchers and a 10% random quality control check of data completed by a different researcher. For CONSORT reporting, screening data includes anonymised summary information for women who do not proceed to randomisation. Paper questionnaires are scanned at site then the original posted to NICTU for data entry directly into the trial database.

For peer review only - http://bmjopen.bmj.com/site/about/guidelines.xhtml

Entered data is processed as per the trial specific Data Management Plan. Data queries are generated electronically by NICTU and site staff are required to respond to clarify data, provide missing information or make relevant amendments within the trial databases.

Online questionnaire completion is stored by the Qualtrics system on EU based servers compliant with General Data Protection Regulation (GDPR) and exported by the Trial Manager for merging with the NICTU questionnaire database by the Trial Statistician (CC). Following completion of data collection, data is permanently deleted from Qualtrics.

Qualitative interview transcripts are anonymised and checked for accuracy against the audio recordings, then the recordings deleted.

A minimal amount of personal information (participant's first name, mobile phone number and weeks postpartum at baseline) is entered into the randomisation system to allow delivery of text messages according to group allocation. Only designated trained team members have password-protected access to the randomisation system.

Text message engagement data is downloaded from the LSHTM system at the end of the intervention period in the form of all messages sent to and replies received from participants.

At the trial end, sites and NICTU send original source documentation (hard copy or electronic) along with consent forms and contact details for individuals who provided consent to be contacted about follow-up to this trial/future-related research/future data linkage to routinely collected health records, to QUB for archiving in accordance with the Sponsor's requirements (10 years after end of trial/publication). The CI will conduct periodic reviews (every five years) to ensure necessity of data retention. Any other documents containing personal data are confidentially destroyed. Sites are responsible for archiving general site files accumulated during the day-to-day operation of the trial.

For peer review only - http://bmjopen.bmj.com/site/about/guidelines.xhtml

Data protection and confidentiality [27]

All data is collected, stored and handled in accordance with the QUB Research Management Policy (2015), GDPR and any future relevant data protection legislation and all trial team members must be compliant with these regulations. Detailed description of the trial data processing is documented in a Data Privacy Impact Assessment and in the privacy notice given to women expressing an interest in the trial.

Participants are allocated a unique 5-digit participant identifier at randomisation which is used on all data collection forms/files to allow identification of data for each participant, with the key for unlocking pseudonymised data held by site teams in password-protected files. Hard copies of forms are securely stored in locked filing cabinets in locked offices in buildings requiring keypad access and which are alarmed outside normal working hours. Consent and contact details forms, containing identifiable information, are stored separately to other data (CRFs and questionnaires) and will not be kept for longer than is required. All electronic forms or data files are held on password-protected systems that are routinely backed up and are only accessible using username, password and/or multi-factor authentication. Third-party transcription providers sign a confidentiality agreement detailing data handling requirements. Any file transfer between QUB and other parties (sites/transcription provider) is done, subject to relevant data sharing agreements, via secure and encrypted file transfer systems (e.g., QUB Dropoff). Participants will not be identifiable from any published trial report.

Data collected from women expressing an interest in trial participation but who are not recruited will be confidentially shredded/deleted.

Access to data [29/31c]

For peer review only - http://bmjopen.bmj.com/site/about/guidelines.xhtml

Access to trial records and source data can be granted to authorised Sponsor (or delegate), REC, host institution and regulatory authority representatives for trial-related monitoring, audits and inspections, as per participant consent.

Only employed trial team members have access to personal/sensitive data of participants and data is shared with those responsible for the healthcare of the participants only when health concerns are raised e.g., EPDS score of nine or greater, see [30].

The Trial Manager, Statistician and Health Economist will have access to the anonymised dataset at the trial end, to permit analysis.

The CI will manage access rights to the trial data set, in collaboration with the sponsor, and formal requests for data access need to be made in writing to the CI. The trial will comply with the good practice principles for sharing individual participant data from publicly funded trials and data sharing will be undertaken in accordance with the required regulatory requirements. Any transfer of data to other institutions will be governed by a Data Access Agreement and will be in the form of an anonymised dataset. In the event of publications arising from further data analyses, those responsible will need to provide the CI with a copy of any intended manuscript for approval prior to submission.

Statistics and analysis

Aligned with best practice and following appropriate reporting guidelines, a Statistical Analysis Plan,(79) Health Economics Analysis Plan (80, 81) and Process Evaluation Analysis Plan are written and approved by the PMT and TSC prior to analysis. Trial results will be reported in accordance with the CONSORT guidelines, with the flow of participants summarised in a CONSORT flow diagram (http://www.consort-statement.org/) and tables of

For peer review only - http://bmjopen.bmj.com/site/about/guidelines.xhtml

summary baseline characteristics presented, by total recruited sample and 12 month completers.

Primary outcome analysis [20a]

Weight change (kg) at 12 months will be compared on an intention to treat basis (i.e., all participants as randomised and regardless of whether or not they engaged with the text messages), between the intervention and active control groups, adjusted for weight at baseline, site, recruitment method (community vs. NHS) and ethnicity. The adjusted difference in means between the groups, corresponding 95% confidence intervals (CIs) and P-value will be reported. A complete-case approach will be used. Sensitivity analysis assessing the impact on the primary outcome analysis will be conducted to: 1) include participant self-reported weight, only where researcher-measured weight is not available; and 2) use last observation carried forward, multiple imputation to impute missing outcome values and delta methods to explore the impact of worse or better outcomes in the individuals with missing data.

The independent TSC in conjunction with NIHR will decide if the trial should progress to stage 3 (24 month follow-up and stakeholder engagement) based on assessment of the 12 month primary outcome data and any other core outcome data required to make a fully informed decision at 12 months (end of intervention). If the trial proceeds, a further analysis of weight change (kg) will be conducted at 24 months. Despite the sequential nature of this comparison, we do not intend to change the significance level because this test will be a secondary analysis.

All testing will be done at the two-sided 5% significance level. All analysis will be conducted using STATA software (Statcorp).

For peer review only - http://bmjopen.bmj.com/site/about/guidelines.xhtml

Secondary outcome analysis [20a]

Similar analyses will be conducted for secondary continuous outcomes and for secondary analyses of outcomes at other time points. Binary outcomes will be analysed using logistic regression, comparing the intervention and active control groups whilst adjusting for site, recruitment method and ethnicity. Adjusted odds ratios with 95% CIs and corresponding P-values will be reported. Secondary analyses will be considered exploratory and hence the P-values will not be altered to control for multiple testing. The focus of all secondary analyses will be on estimating the difference between-groups with 95% CIs rather than hypothesis testing.

Methods for additional analyses (e.g., subgroup analyses) [20b]

To understand any differential effects of the intervention, subgroup analyses will be conducted stratifying by pre-specified variables including site, SES, ethnicity, recruitment method, weeks postpartum (at baseline), BMI (at baseline) and parity. Interaction tests will be conducted, separately, by including interaction terms within regression models. The significance level for subgroup analyses will not be reduced but will be interpreted cautiously. As recommended, all subgroup analyses will be reported.(82)

Statistical methods to handle protocol non-adherence and missing data [20c]

The primary analysis of weight change (kg) at 12 months will be on an intention to treat basis.

Interim analyses [21b]

No interim analyses are planned.

Economic evaluation

The within trial economic evaluation builds on the methods successfully used in the pilot economic evaluation (27) including the identification, measurement and valuation of resource use and expenditure and quality of life/capability wellbeing (Table 1). The economic evaluation is conducted from a UK NHS and personal social services perspective with the addition of a societal perspective applied to capture broader impacts, consistent with the UK's NICE guidance for public health economic evaluations.(83) Direct costs to the health care system include the intervention implementation costs and any follow-up service use costs. These health and personal perspectives will be assessed to investigate a broader impact of the intervention, including direct cost to participants and indirect costs. Both costs and health outcomes will be discounted at the same annual rate of 1.5% for the reference case, as recommended by the NICE public health methods guidelines.(83) The economic analysis uses a 'multi-pronged' approach. The total and mean per-participant costs for the intervention and control groups will be calculated. A sensitivity analysis will incorporate development costs. Regression analysis, controlling for baseline differences, will be used to estimate the average cost per-participant and the average quality-adjusted life year (QALY) achieved, by group. A cost-utility analysis will report cost per QALY gained over the 24 month period using the area under the curve approach.(84) Results will be presented on the cost-effectiveness plan and 95% CIs for the incremental cost-effectiveness ratio will be determined.(85) Joint uncertainty in costs and outcomes will be represented using a cost-effectiveness acceptability curve to present the probability of the SMS intervention being cost-effective for prevailing UK ceiling thresholds for costs per QALY gained.(86) The cost-

effectiveness analysis will align with the primary outcome, by examining differences in weight and BMI and total costs between treatment groups, the incremental cost per weight gain averted will be calculated. The mean costs and effects for each trial arm will be calculated and presented along with the incremental costs and effects between arms (including 95% CIs).

The within trial economic analysis will be carried out for the 24 months follow-up and will form the primary analysis. However, this short time horizon may be insufficient to capture the total costs and benefits related to the intervention. If differences in quality of life or capability wellbeing are identified, the results will be extrapolated to a lifetime horizon using modelling methods.

Monitoring and auditing [23]

As per contractual agreements and participant consent, direct access to trial records and source documents will be granted to the Sponsor or regulatory agencies by all research staff and trial sites, for the purposes of trial related monitoring, audits and inspections. The trial is monitored and audited in accordance with the Sponsor's policy, which is consistent with the UK Policy Framework for Health and Social Care Research, and the TSC informed of the main findings of any monitoring, audit or inspection.

Plans to give access to the full protocol, participant-level data and statistical code

The full protocol can be obtained from https://fundingawards.nihr.ac.uk/award/NIHR131509 and the statistical analysis plan is available by request from the CI. Following publication of the results, anonymised participant-level datasets and statistical code used for generating the results are available from the corresponding author on reasonable request, as assessed

For peer review only - http://bmjopen.bmj.com/site/about/guidelines.xhtml

by the CI and PMT and subject to any necessary data sharing agreements (see [29/31c]). Data availability will be consistent with timelines for storage of research data (see [19]).

Trial Steering Committee (TSC) and Data Monitoring [5d/21a]

The independent TSC meet face-to-face or online at least annually, as required, to provide trial oversight on behalf of the sponsor and funder. The TSC determine trial progression at the recruitment mid-point (see Figure 1 and [15]) and primary outcome stage. As this RCT involves a low-risk behaviour change lifestyle intervention which is unlikely to raise concerns for participant safety (no evidence of harm was reported in the pilot study or in any other text message behaviour change studies), the sponsor and funder agreed that a Data Monitoring Committee would not be convened [21a]. Participant safety is a standing agenda item at each meeting, see [22].

Project Management Team (PMT) [5d]

The PMT is the key decision-making group consisting of the co-applicants, Trial Manager and PPI representative. The PMT meet online every 2-4 months to oversee the trial design, management and conduct, including monitoring participant safety, see [22].

Trial coordinating centre [5d]

The trial is coordinated by the CI (MMcK) and the Trial Manager (DG) in QUB. Weekly meetings between the CI and Trial Manager and regular meetings with site teams are held, to discuss trial activities across the five trial sites. Site PIs are responsible for managing staff and overseeing participant recruitment and follow-up at their sites.

For peer review only - http://bmjopen.bmj.com/site/about/guidelines.xhtml

Adverse event reporting and harms [22]

Serious adverse events (SAEs) are recorded by researchers from the time of the participant's consent into the trial until one month after the end of the 12 month intervention period and reported in line with GCP guidelines and the Sponsor's adverse event reporting procedures. Site PIs review SAEs within 24 hours to determine severity, relatedness and expectedness. The CI (or delegate) is informed of any event evaluated as being severe, related and unexpected, to conduct a second review. Onward reporting of any SAEs categorised as related and expected by both the PI and CI is actioned by the CI or Trial Manager to the Sponsor and REC within 24 hours and 15 days respectively. All SAEs are summarised and included in annual reports to the REC and reported during meetings of the PMT and TSC.

Ancillary and post-trial care [30]

This is deemed a low-risk public health trial and no evidence of harm has been reported in text message studies for other health behaviours or in our pilot RCT, so no post-trial care is delivered. All women are given a signposting 'Useful Contacts' leaflet during trial visits advising them on relevant agencies and organisations that they might contact for support on a variety of issues e.g., bereavement. We monitor any emerging safety issues or unintended consequences and any participants experiencing such issues will be referred to their GP for appropriate treatment. This includes the onward reporting of EPDS scores of nine or above (Table 1). The EPDS is a screening questionnaire used to detect postnatal depressive symptoms and the scale guidance indicates that women with scores meeting this threshold would benefit from further clinical assessment. We calculate scores within one week of

receiving the completed EPDS at each follow-up timepoint and letters are sent to the GP and the participant to inform of scores ≥9, as per PIS and consent processes.

Personal and Public Involvement (PPI)* Statement

(Note: In NI, PPI means Personal and Public Involvement due to integrated Health and Social Care systems. The rest of the UK use 'Patient and Public Involvement' also known as 'Patient and Public Involvement and Engagement')*

PPI was integral to the pilot study with guidance received on the development of the text message libraries and all trial methods and materials, as previously reported.(27) The design of the present trial mirrors the methods used in the pilot trial. We recruited PPI representatives from Scotland, London and Bradford to conduct a period of trial adaptation (Figure 1) whereby the library of intervention text messages and trial recruitment materials underwent further PPI to ensure content was acceptable and culturally relevant for women from a range of backgrounds and ethnicities across the UK.

We have PPI representatives on our PMT and independent TSC to contribute to decision making throughout the trial and we seek their advice on an ad-hoc basis, as matters arise. PPI representatives will help shape the dissemination plan for trial results and will review dissemination materials to ensure communication of findings in an appropriate manner.

PPI work is led by the trial team and conducted and reimbursed according to NIHR guidance for involving the public in research.(87)

Protocol amendments [25]

For peer review only - http://bmjopen.bmj.com/site/about/guidelines.xhtml

871 The CI, in consultation with the PMT and Sponsor, is responsible for the decision to amend the protocol and, in turn, for communicating substantive changes to relevant stakeholders. Substantial and non-substantial amendments are reviewed by the sponsor to determine the appropriate amendment category then submitted to the approving REC via the IRAS system for approval prior to implementation, and subsequently disseminated to participating centres (R&D offices, regulatory agencies). Version control and details of amendment history are clearly documented in the protocol and are disseminated to the trial sites, PMT and TSC members, funder and trial registries.

879 The current protocol is 3.0 (10/05/2023). Amendments to the protocol incorporated: 1) the added mental health objectives resulting from the additional NIHR funding; 2) an increase in trial sample size (from 850 to 888 women) to allow for higher estimated pregnancy exclusion rates in more ethnically diverse populations than were originally accounted for; 3) to include processes to collect participant self-reported weight at 12 and 24 months only as a last resort in cases where it is not possible to obtain researcher-measured weight; and 4) to offer a booklet summarising the intervention content to the active control group after completion of data collection at 24 months.

888 Protocol training and deviations

889 All trial staff undergo training on the protocol, trial processes and standard operating procedures (SOPs) for which they are responsible (detailed on the site delegation log). The Trial Manager monitors trial activities across sites to ensure adherence and consistency to the protocol. There are no planned protocol deviations. If a deviation occurs, it is documented and reported immediately to the CI and Sponsor, including corrective and preventative actions. Frequent recurrence of protocol deviations is unacceptable, requires immediate action and could be classified as a serious breach i.e., one which is likely to significantly affect the safety or physical or mental integrity of trial participants and/or the

For peer review only - http://bmjopen.bmj.com/site/about/guidelines.xhtml

scientific value of the trial. Any breach meeting this definition is immediately reported to the Sponsor, as per the Sponsor's SOP on 'Matters of non-compliance with study protocol'.

Peer review

This trial has undergone independent, expert peer-review as part of the NIHR PHR funding process. It has also been reviewed and approved by the QUB Research Governance, Ethics and Integrity Manager, on behalf of the Sponsor.

Ethics and dissemination

Ethical and Regulatory considerations [24]

The trial is conducted in accordance with the recommendations for physicians involved in research on human participants adopted by the 18th World Medical Assembly, Helsinki 1964, and later revisions, the UK Policy Framework for Health and Social Care, relevant UK data protection legislation and the principles of GCP. QUB acts as sponsor for the trial and has in place comprehensive SOPs for the approval and monitoring of research. This protocol and other relevant trial documents were reviewed and approved by the West of Scotland Research Ethics Service REC 4 22/WS/0003. Correspondence with the REC is retained in the Trial Master File. An annual progress report is submitted to REC until the trial end. The CI will report to REC when the trial ends (or is terminated prematurely) and a final report with trial results submitted within one year of this.

Appropriate approvals from participating organisations e.g., NHS R&D approvals are obtained before recruiting via applicable routes.

Dissemination [31a]

For peer review only - http://bmjopen.bmj.com/site/about/guidelines.xhtml

Dissemination plans informed by PPI and participant and stakeholder engagement will be developed and reviewed annually. A range of outputs including NIHR final report, peer-reviewed open access publications, conference presentations, policy briefings, non-academic research summaries and a draft scaling-up plan, are anticipated to be disseminated relevant for different audiences, including trial participants, postpartum women throughout the UK, healthcare professionals, service commissioners, academics, the Government and public health bodies.

References

1. Scott-Pillai R, Spence D, Cardwell CR, Hunter A, Holmes VA. The impact of body mass index on maternal and neonatal outcomes: a retrospective study in a UK obstetric population, 2004-2011. Bjog. 2013;120(8):932-9.
2. Marchi J, Berg M, Dencker A, Olander EK, Begley C. Risks associated with obesity in pregnancy, for the mother and baby: a systematic review of reviews. Obes Rev. 2015;16(8):621-38.
3. Bhattacharya S, Campbell DM, Liston WA, Bhattacharya S. Effect of Body Mass Index on pregnancy outcomes in nulliparous women delivering singleton babies. BMC Public Health. 2007;7:168.
4. Aune D, Saugstad OD, Henriksen T, Tonstad S. Maternal body mass index and the risk of fetal death, stillbirth, and infant death: a systematic review and meta-analysis. Jama. 2014;311(15):1536-46.
5. Crozier SR, Inskip HM, Godfrey KM, Cooper C, Harvey NC, Cole ZA, et al. Weight gain in pregnancy and childhood body composition: findings from the Southampton Women's Survey123. The American Journal of Clinical Nutrition. 2010;91(6):1745-51.
6. Crosby DA, Collins M, O'Higgins A, Mullaney L, Farah N, Turner MJ. Interpregnancy changes in maternal weight and body mass index. Am J Perinatol. 2015;30(2):199-204.
7. McKinley MC, Allen-Walker V, McGirr C, Rooney C, Woodside JV. Weight loss after pregnancy: challenges and opportunities. Nutr Res Rev. 2018;31(2):225-38.
8. Lim S, O'Reilly S, Behrens H, Skinner T, Ellis I, Dunbar JA. Effective strategies for weight loss in post-partum women: a systematic review and meta-analysis. Obes Rev. 2015;16(11):972-87.
9. Nascimento SL, Pudwell J, Surita FG, Adamo KB, Smith GN. The effect of physical exercise strategies on weight loss in postpartum women: a systematic review and meta-analysis. Int J Obes (Lond). 2014;38(5):626-35.
10. van der Pligt P, Willcox J, Hesketh KD, Ball K, Wilkinson S, Crawford D, et al. Systematic review of lifestyle interventions to limit postpartum weight retention: implications for future opportunities to prevent maternal overweight and obesity following childbirth. Obes Rev. 2013;14(10):792-805.
11. National Institute for Health and Care Excellence (NICE). Weight Management Before, During and After Pregnancy: NICE Public Health Guidance 27. 2010 [Available from: https://www.nice.org.uk/guidance/ph27.
12. Amorim Adegboye AR, Linne YM. Diet or exercise, or both, for weight reduction in women after childbirth. Cochrane Database Syst Rev. 2013;2013(7):Cd005627.

For peer review only - http://bmjopen.bmj.com/site/about/guidelines.xhtml

For peer review only

13. Dodd JM, Deussen AR, O'Brien CM, Schoenaker D, Poprzeczny A, Gordon A, et al. Targeting the postpartum period to promote weight loss: a systematic review and meta-analysis. Nutr Rev. 2018;76(8):639-54.
14. Farpour-Lambert NJ, Ells LJ, Martinez de Tejada B, Scott C. Obesity and Weight Gain in Pregnancy and Postpartum: an Evidence Review of Lifestyle Interventions to Inform Maternal and Child Health Policies. Front Endocrinol (Lausanne). 2018;9:546.
15. Joshi PP, Quintiliani LM, McCarthy AC, Gilmore A, Mahesri M, Sullivan LM, et al. A Randomized Controlled Feasibility Trial in Behavioral Weight Management for Underserved Postpartum African American Women: The RENEW Study. Prev Chronic Dis. 2018;15:E77.
16. Wright C, Mogul M, Acevedo G, Aysola J, Momplaisir F, Schwartz S, et al. Preparing for a trial to test a postpartum weight retention intervention among low income women: feasibility of a protocol in a community-based organization. BMC Womens Health. 2018;18(1):27.
17. Haste A, Adamson AJ, McColl E, Araujo-Soares V, Bell R. Problems recruiting and retaining postnatal women to a pilot randomised controlled trial of a web-delivered weight loss intervention. BMC Res Notes. 2018;11(1):203.
18. Montgomery KS, Bushee TD, Phillips JD, Kirkpatrick T, Catledge C, Braveboy K, et al. Women's challenges with postpartum weight loss. Matern Child Health J. 2011;15(8):1176-84.
19. Neiterman E, Fox B. Controlling the unruly maternal body: Losing and gaining control over the body during pregnancy and the postpartum period. Soc Sci Med. 2017;174:142-8.
20. Christenson A, Johansson E, Reynisdottir S, Torgerson J, Hemmingsson E. Women's Perceived Reasons for Their Excessive Postpartum Weight Retention: A Qualitative Interview Study. PLoS One. 2016;11(12):e0167731.
21. Saligheh M, McNamara B, Rooney R. Perceived barriers and enablers of physical activity in postpartum women: a qualitative approach. BMC Pregnancy and Childbirth. 2016;16(1):131.
22. Devine CM, Bove CF, Olson CM. Continuity and change in women's weight orientations and lifestyle practices through pregnancy and the postpartum period: the influence of life course trajectories and transitional events. Soc Sci Med. 2000;50(4):567-82.
23. O'Toole ML, Sawicki MA, Artal R. Structured diet and physical activity prevent postpartum weight retention. J Womens Health (Larchmt). 2003;12(10):991-8.
24. Macleod M, Craigie AM, Barton KL, Treweek S, Anderson AS. Recruiting and retaining postpartum women from areas of social disadvantage in a weight-loss trial--an assessment of strategies employed in the WeighWell feasibility study. Matern Child Nutr. 2013;9(3):322-31.
25. Taylor C, Bhavnani V, Zasada M, Ussher M, Bick D. Barriers and facilitators to uptake and retention of inner-city ethnically diverse women in a postnatal weight management intervention: a mixed-methods process evaluation within a feasibility trial in England. BMJ Open. 2020;10(7):e034747.
26. Østbye T, Krause KM, Lovelady CA, Morey MC, Bastian LA, Peterson BL, et al. Active Mothers Postpartum: a randomized controlled weight-loss intervention trial. Am J Prev Med. 2009;37(3):173-80.
27. McGirr C, Rooney C, Gallagher D, Dombrowski SU, Anderson AS, Cardwell CR, et al. Text messaging to help women with overweight or obesity lose weight after childbirth: the intervention adaptation and SMS feasibility RCT. NIHR Journals Library: Public Health Research. 2020;8(4).
28. Herring SJ, Cruice JF, Bennett GG, Darden N, Wallen JJ, Rose MZ, et al. Intervening during and after pregnancy to prevent weight retention among African American women. Prev Med Rep. 2017;7:119-23.
29. Waring ME, Moore Simas TA, Oleski J, Xiao RS, Mulcahy JA, May CN, et al. Feasibility and Acceptability of Delivering a Postpartum Weight Loss Intervention via Facebook: A Pilot Study. J Nutr Educ Behav. 2018;50(1):70-4.e1.
30. Willcox JC, Dobson R, Whittaker R. Old-Fashioned Technology in the Era of "Bling": Is There a Future for Text Messaging in Health Care? J Med Internet Res. 2019;21(12):e16630.

For peer review only
For peer review only - http://bmjopen.bmj.com/site/about/guidelines.xhtml

31. Job JR, Fjeldsoe BS, Eakin EG, Reeves MM. Effectiveness of extended contact interventions for weight management delivered via text messaging: a systematic review and meta-analysis. Obesity Reviews. 2018;19(4):538-49.

32. Samuel Holmes W, Anne Moorhead S, Coates VE, Bond RR, Zheng H. Impact of digital technologies for communicating messages on weight loss maintenance: a systematic literature review. European Journal of Public Health. 2018;29(2):320-8.

33. Schippers M, Adam PC, Smolenski DJ, Wong HT, de Wit JB. A meta-analysis of overall effects of weight loss interventions delivered via mobile phones and effect size differences according to delivery mode, personal contact, and intervention intensity and duration. Obes Rev. 2017;18(4):450-9.

34. Siopis G, Chey T, Allman-Farinelli M. A systematic review and meta-analysis of interventions for weight management using text messaging. J Hum Nutr Diet. 2015;28 Suppl 2:1-15.

35. Skinner R, Gonet V, Currie S, Hoddinott P, Dombrowski SU. A systematic review with meta-analyses of text message-delivered behaviour change interventions for weight loss and weight loss maintenance. Obes Rev. 2020;21(6):e12999.

36. Guerriero C, Cairns J, Roberts I, Rodgers A, Whittaker R, Free C. The cost-effectiveness of smoking cessation support delivered by mobile phone text messaging: Txt2stop. Eur J Health Econ. 2013;14(5):789-97.

37. Free C, Knight R, Robertson S, Whittaker R, Edwards P, Zhou W, et al. Smoking cessation support delivered via mobile phone text messaging (txt2stop): a single-blind, randomised trial. The Lancet. 2011;378(9785):49-55.

38. Morgan KL, Rahman MA, Macey S, Atkinson MD, Hill RA, Khanom A, et al. Obesity in pregnancy: a retrospective prevalence-based study on health service utilisation and costs on the NHS. BMJ Open. 2014;4(2):e003983.

39. Rawdin AC, Duenas A, Chilcott JB. The cost-effectiveness of weight management programmes in a postnatal population. Public Health. 2014;128(9):804-10.

40. Denison FC, Aedla NR, Keag O, Hor K, Reynolds RM, Milne A, et al. Care of women with obesity in pregnancy: Green-top guideline no.72. BJOG. 2018.

41. Phelan S. Pregnancy: a "teachable moment" for weight control and obesity prevention. Am J Obstet Gynecol. 2010;202(2):135.e1-8.

42. Dinsdale S, Branch K, Cook L, Shucksmith J. “As soon as you’ve had the baby that’s it…” a qualitative study of 24 postnatal women on their experience of maternal obesity care pathways. BMC Public Health. 2016;16(1):625.

43. Lipsky LM, Strawderman MS, Olson CM. Maternal weight change between 1 and 2 years postpartum: the importance of 1 year weight retention. Obesity (Silver Spring). 2012;20(7):1496-502.

44. Østbye T, Peterson BL, Krause KM, Swamy GK, Lovelady CA. Predictors of postpartum weight change among overweight and obese women: results from the Active Mothers Postpartum study. J Womens Health (Larchmt). 2012;21(2):215-22.

45. Cobiac L, Vos T, Veerman L. Cost-effectiveness of Weight Watchers and the Lighten Up to a Healthy Lifestyle program. Aust N Z J Public Health. 2010;34(3):240-7.

46. Hoddinott P, Allan K, Avenell A, Britten J. Group interventions to improve health outcomes: a framework for their design and delivery. BMC Public Health. 2010;10(1):800.

47. White M, Adams J, Heywood P. How and why do interventions that increase health overall widen inequalities within populations? In: Babones SJ, editor. Social inequality and public health: Policy Press; 2009. p. 0.

48. Shaw R, Bosworth H. Short message service (SMS) text messaging as an intervention medium for weight loss: A literature review. Health Informatics J. 2012;18(4):235-50.

49. Sherifali D, Nerenberg KA, Wilson S, Semeniuk K, Ali MU, Redman LM, et al. The Effectiveness of eHealth Technologies on Weight Management in Pregnant and Postpartum Women: Systematic Review and Meta-Analysis. J Med Internet Res. 2017;19(10):e337.

For peer review only - http://bmjopen.bmj.com/site/about/guidelines.xhtml

50. Scottish Government. Scotland's Digital Future. A Strategy for Scotland. Edinburgh: Scottish Government; 2011.
51. Born in Bradford. Me and my Baby Measure 2023 [Available from: https://borninbradford.nhs.uk/gallery/me-and-my-baby-measure/.
52. Chan A-W, Tetzlaff JM, Altman DG, Laupacis A, Gøtzsche PC, Krleža-Jerić K, et al. SPIRIT 2013 Statement: Defining standard protocol items for clinical trials. . Ann Intern Med. 2013;158:200-7.
53. Hoffmann TC, Glasziou PP, Boutron I, Milne R, Perera R, Moher D, et al. Better reporting of interventions: template for intervention description and replication (TIDieR) checklist and guide. BMJ : British Medical Journal. 2014;348:g1687.
54. Kwasnicka D, Dombrowski SU, White M, Sniehotta F. Theoretical explanations for maintenance of behaviour change: a systematic review of behaviour theories. Health Psychology Review. 2016;10(3):277-96.
55. Catenacci VA, Odgen L, Phelan S, Thomas JG, Hill J, Wing RR, et al. Dietary habits and weight maintenance success in high versus low exercisers in the National Weight Control Registry. J Phys Act Health. 2014;11(8):1540-8.
56. Marinilli Pinto A, Gorin AA, Raynor HA, Tate DF, Fava JL, Wing RR. Successful weight-loss maintenance in relation to method of weight loss. Obesity (Silver Spring). 2008;16(11):2456-61.
57. Tang J, Abraham C, Greaves C, Yates T. Self-directed interventions to promote weight loss: a systematic review of reviews. J Med Internet Res. 2014;16(2):e58.
58. Phelan S, Hagobian T, Brannen A, Hatley KE, Schaffner A, Muñoz-Christian K, et al. Effect of an Internet-Based Program on Weight Loss for Low-Income Postpartum Women: A Randomized Clinical Trial. Jama. 2017;317(23):2381-91.
59. Firetext. Firetext SMS provider 2023 [Available from: https://www.firetext.co.uk/.
60. National Health Service. Better Health: Start for Life 2023 [Available from: https://www.nhs.uk/start-for-life/.
61. Brooks R. EuroQol: the current state of play. Health Policy. 1996;37(1):53-72.
62. Al-Janabi H, Flynn TN, Coast J. Development of a self-report measure of capability wellbeing for adults: the ICECAP-A. Qual Life Res. 2012;21(1):167-76.
63. Wright JL, Scott JA. The Fat and Fibre Barometer, a short food behaviour questionnaire: reliability, relative validity and utility. Aus J Nutr Dietet. 2000;57:33-9.
64. Booth M. Assessment of physical activity: an international perspective. Res Q Exerc Sport. 2000;71 Suppl 2:114-20.
65. McAndrew F, Thompson J, Fellows L, Large A, Speed M, Renfrew MJ. Infant Feeding Survey 2010.: Health and Social Care Information Centre; 2012.
66. Cox JL, Holden JM, Sagovsky R. Detection of postnatal depression. Development of the 10-item Edinburgh Postnatal Depression Scale. Br J Psychiatry. 1987;150:782-6.
67. Spitzer RL, Kroenke K, Williams JB, Löwe B. A brief measure for assessing generalized anxiety disorder: the GAD-7. Arch Intern Med. 2006;166(10):1092-7.
68. Oates J, Gervai J. Mothers' Perceptions of Their Infants. Journal of Prenatal and Perinatal Psychology and Health. 2019;33(4):282-300.
69. Buysse DJ, Reynolds CF, 3rd, Monk TH, Berman SR, Kupfer DJ. The Pittsburgh Sleep Quality Index: a new instrument for psychiatric practice and research. Psychiatry Res. 1989;28(2):193-213.
70. Sniehotta FF, Schwarzer R, Scholz U, Schüz B. Action planning and coping planning for long-term lifestyle change: theory and assessment. European Journal of Social Psychology. 2005;35(4):565-76.
71. Schwarzer R, Renner B. Social-cognitive predictors of health behavior: action self-efficacy and coping self-efficacy. Health Psychol. 2000;19(5):487-95.
72. Gardner B, Abraham C, Lally P, de Bruijn G-J. Towards parsimony in habit measurement: Testing the convergent and predictive validity of an automaticity subscale of the Self-Report Habit Index. International Journal of Behavioral Nutrition and Physical Activity. 2012;9(1):102.

For peer review only

For peer review only - http://bmjopen.bmj.com/site/about/guidelines.xhtml

73. Kliemann N, Beeken RJ, Wardle J, Johnson F. Development and validation of the Self-Regulation of Eating Behaviour Questionnaire for adults. International Journal of Behavioral Nutrition and Physical Activity. 2016;13(1):87.

74. Meyer AH, Weissen-Schelling S, Munsch S, Margraf J. Initial development and reliability of a motivation for weight loss scale. Obes Facts. 2010;3(3):205-11.

75. Sallis JF, Grossman RM, Pinski RB, Patterson TL, Nader PR. The development of scales to measure social support for diet and exercise behaviors. Prev Med. 1987;16(6):825-36.

76. Rosenberg M. Conceiving the self. New York: Basic Books; 1979.

77. Born in Bradford. Born in Bradford's Better Start. 2023 [Available from: https://borninbradford.nhs.uk/what-we-do/cohort-studies/better-start/.

78. Avery KNL, Williamson PR, Gamble C, O'Connell Francischetto E, Metcalfe C, Davidson P, et al. Informing efficient randomised controlled trials: exploration of challenges in developing progression criteria for internal pilot studies. BMJ Open. 2017;7(2):e013537.

79. Gamble C, Krishan A, Stocken D, Lewis S, Juszczak E, Doré C, et al. Guidelines for the Content of Statistical Analysis Plans in Clinical Trials. JAMA. 2017;318(23):2337-43.

80. Thorn JC, Davies CF, Brookes ST, Noble SM, Dritsaki M, Gray E, et al. Content of Health Economics Analysis Plans (HEAPs) for Trial-Based Economic Evaluations: Expert Delphi Consensus Survey. Value Health. 2021;24(4):539-47.

81. Husereau D, Drummond M, Augustovski F, de Bekker-Grob E, Briggs AH, Carswell C, et al. Consolidated Health Economic Evaluation Reporting Standards (CHEERS) 2022 Explanation and Elaboration: A Report of the ISPOR CHEERS II Good Practices Task Force. Value in Health. 2022;25(1):10-31.

82. van Hoorn R, Tummers M, Booth A, Gerhardus A, Rehfuess E, Hind D, et al. The development of CHAMP: a checklist for the appraisal of moderators and predictors. BMC Medical Research Methodology. 2017;17(1):173.

83. National Institute for Health and Care Excellence (NICE). Methods for the development of NICE public health guidance (third edition): process and methods.; 2012.

84. Manca A, Hawkins N, Sculpher MJ. Estimating mean QALYs in trial-based cost-effectiveness analysis: the importance of controlling for baseline utility. Health Econ. 2005;14(5):487-96.

85. Drummond MF, Sculpher MJ, Claxton K, Stoddart GL, Torrance GW. Methods for the Economic Evaluation of Health Care Programmes (fourth edition). Oxford: Oxford University Press; 2015.

86. Fenwick E, Marshall DA, Levy AR, Nichol G. Using and interpreting cost-effectiveness acceptability curves: an example using data from a trial of management strategies for atrial fibrillation. BMC Health Services Research. 2006;6(1):52.

87. National Institute for Health and Care Research (NIHR). Payments guidance for members of the public, Version 1.4. 2023 [Available from: https://www.nihr.ac.uk/documents/payment-guidance-for-members-of-the-public-considering-involvement-in-research/27372.

Authors' contributions [31b]

MCM led the funding proposal and protocol development with support from all co-authors (DG, ES, ASA, SB, CC, EC, SUD, CF, SH, PH, FK, CMcD, EM, JVW) who provided expert input on trial design and intervention content. MCM, ES and DG co-ordinated the ethical approval. DG drafted the protocol manuscript. All authors (DG, ES, ASA, SB, CC, EC, SUD,

For peer review only - http://bmjopen.bmj.com/site/about/guidelines.xhtml

CF, SH, PH, FK, CMcD, EM, JVW, MCM) contributed to the revision of the manuscript and have read and approved the final version.

Funding Statement [4/5c]

This trial is supported by the NIHR Public Health Research (PHR) Programme (NIHR131509) and intervention costs provided by the Northern Ireland Public Health Agency. The funder is not responsible for the trial design, collection, management, analysis or interpretation of data or report writing but will oversee the trial progress, including publications.

For peer review only

Trial sponsor details [5b/5c]

This trial is sponsored by Queen's University Belfast (Contact details: Dr Paula Tighe, Research Governance, Ethics and Integrity Manager, Queen's University Belfast, p.tighe@qub.ac.uk). The sponsor is not responsible for the trial design, collection, management, analysis or interpretation of data or report writing but will oversee the trial progress, including publications.

Competing interests' statement [28]

The CI, PIs, co-investigators, trial staff and TSC are required to disclose information on competing interests that might influence the trial integrity, such as commercial ownership interests. A record of competing interests is retained by the Trial Manager for reporting in all trial publications. EMcl was a member of the NIHR PHR funding board 2017-2021 but was excluded from the decision-making process for present trial funding. CF was a member of the NIHR HTA funding board 2018-2022. JVW is a trustee of the Nutrition Society for which

For peer review only - http://bmjopen.bmj.com/site/about/guidelines.xhtml

she does not receive payment. PH is a co-investigator on other NIHR funded trials (NIHR 129703 and NIHR 129182), and is a current or past member of several advisory groups (member of the Scottish Government Peer Support Advisory Group for infant feeding and Chair of the Trial Steering Committees for other NIHR funded projects (RP-PG-1211-20015; NIHR 300895 Fellowship Award; NIHR HSDR 130995; NIHR PHR127793; and, NIHR HTA16/143/01) for which she does not receive payment other than reimbursement of travel expenses for face-to-face meetings.

Acknowledgements

For the purpose of open access, the author has applied a Creative Commons Attribution (CC BY) licence to any Author Accepted Manuscript version arising from this submission.

Word Count: 9251

For peer review only - http://bmjopen.bmj.com/site/about/guidelines.xhtml

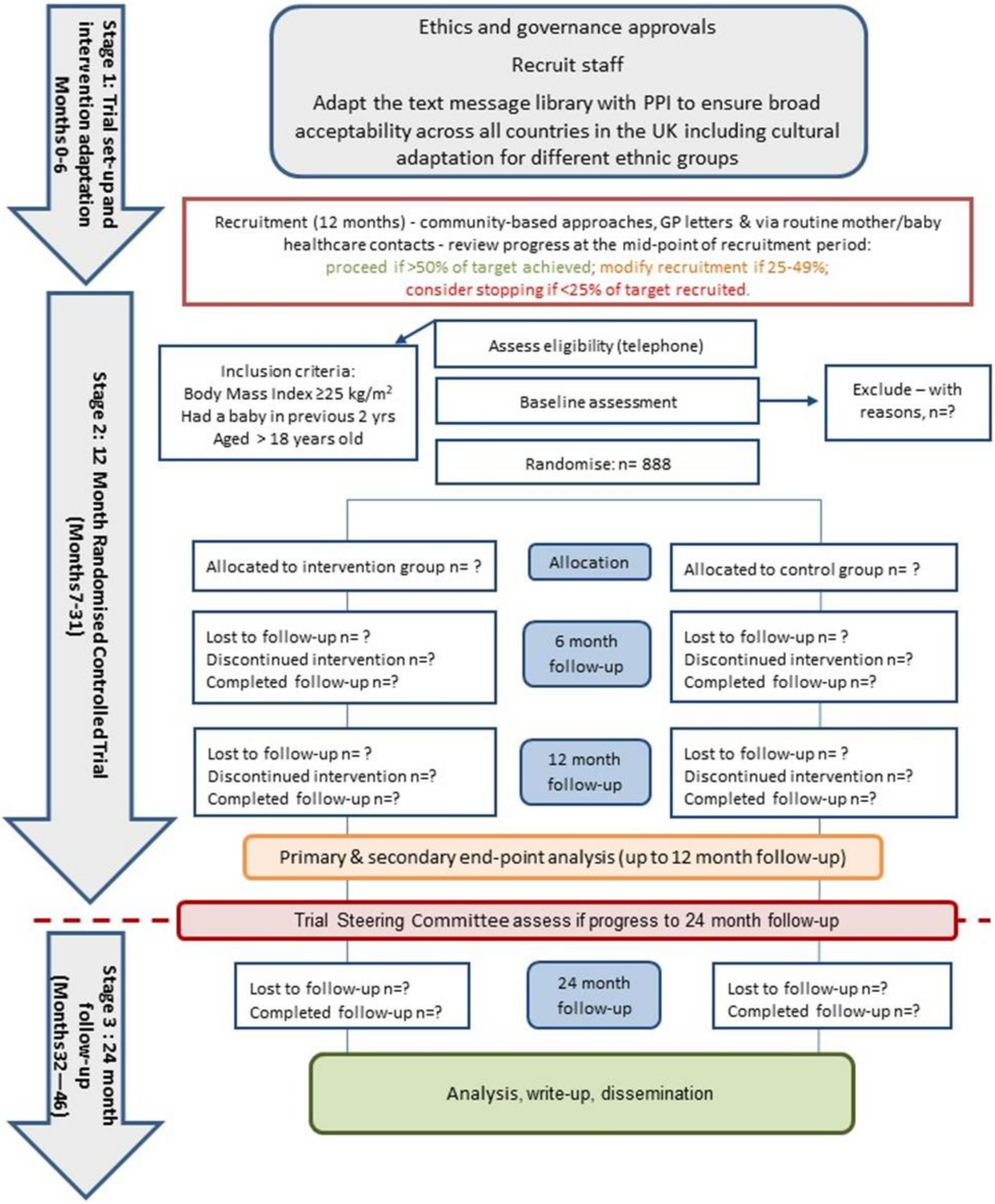
Stage 1: Trial set-up and intervention adaptation Months 0-6
Ethics and governance approvals
Recruit staff
Adapt the text message library with PPI to ensure broad acceptability across all countries in the UK including cultural adaptation for different ethnic groups
Recruitment (12 months) - community-based approaches, GP letters & via routine mother/baby healthcare contacts - review progress at the mid-point of recruitment period:
proceed if >50% of target achieved; modify recruitment if 25-49%;
consider stopping if <25% of target recruited.
Stage 2: 12 Month Randomised Controlled Trial (Months 7-31)
Assess eligibility (telephone)
Inclusion criteria:
Body Mass Index ≥25 kg/m²
Had a baby in previous 2 yrs
Aged > 18 years old
Baseline assessment
Exclude – with reasons, n=?
Randomise: n= 888
Allocated to intervention group n= ?
Allocation
Allocated to control group n= ?
Lost to follow-up n= ?
Discontinued intervention n=?
Completed follow-up n=?
6 month follow-up
Lost to follow-up n= ?
Discontinued intervention n=?
Completed follow-up n=?
Lost to follow-up n= ?
Discontinued intervention n=?
Completed follow-up n=?
12 month follow-up
Lost to follow-up n= ?
Discontinued intervention n=?
Completed follow-up n=?
Primary & secondary end-point analysis (up to 12 month follow-up)
Trial Steering Committee assess if progress to 24 month follow-up
Stage 3 : 24 month follow-up (Months 32—46)
Lost to follow-up n=?
Completed follow-up n=?
24 month follow-up
Lost to follow-up n=?
Completed follow-up n=?
Analysis, write-up, dissemination

Supplemental appendix 1- SMS Logic Model and sample text messages

a) Supporting MumS intervention logic model

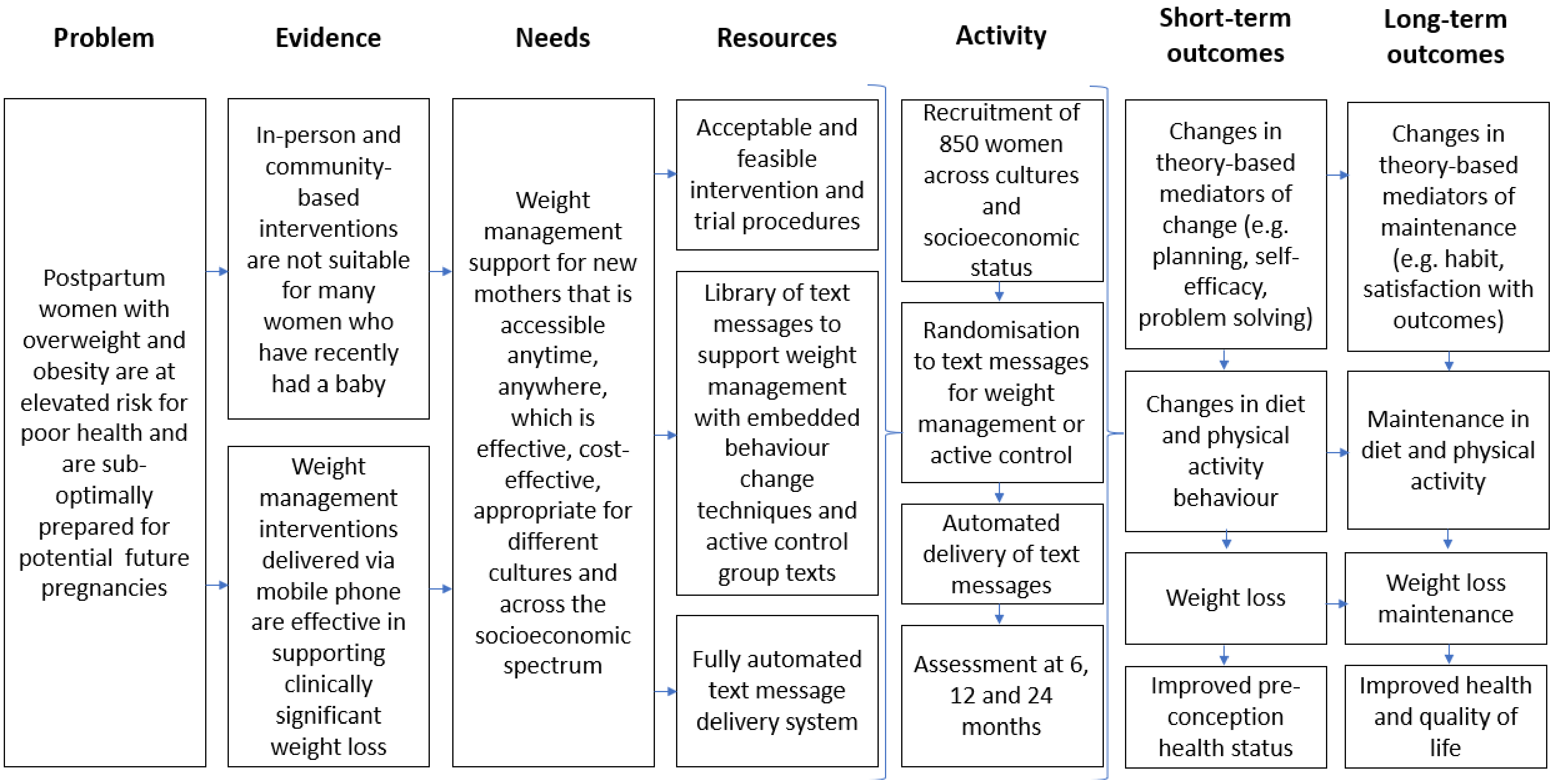

b) Supporting MumS sample text messages

**Type of text message**		**Example of message sent**	**Example of automated responses (if applicable)**
**Core intervention text message**		*'Another tip for success: Keep track of what you eat. People who regularly monitor what they eat are more likely to meet their weight loss target. Stay tuned for more texts on food tracking'.*	
**Weekly self-weighing reminder**	**Months 0-6**	*'What are the scales saying this week? Text us e.g. 11 st 5, or 73.3 kg'.*	N/A
	**Months 7-12**	*'Weekly weighing = your very own early warning system! Step on the scales today and text us your weight followed by UP, DOWN, SAME. e.g. "10 st 7 SAME" or "66.7 kg SAME'.*	<u>Up</u>: *'Don't give up! The best thing to do is prevent these small regains from turning into bigger regains. What could you do this week? E.g. maybe exercise a little more or be more mindful of portion sizes?'*
			<u>Down</u>: *'Success! Your efforts are paying off! All the hard work is worth it :)'.*

For peer review only

For peer review only - http://bmjopen.bmj.com/site/about/guidelines.xhtml

			Same: *'Maintaining your weight is something to be proud of! Treat yourself to a reward e.g. a relaxing bath or simply 10 minutes to yourself!'*
**Yes/No question**		*'WEEK 14. Have you set your activity goal for the week ahead? Text Y11 for yes or N11 for no'.*	Y11 (yes): *'Great job, setting goals is clearly becoming one of your weekly habits :) Alongside your activity goal, set a food goal too and you're onto a double winner!'*
			N11 (no): *'New to the whole activity thing? Set a goal to do a small amount of an activity that you enjoy - for example, a brisk 15-minute walk or a zumba routine when your little one goes to sleep. It will help motivate you and give you a boost of confidence to do more next time'.*

For peer review only

**Trigger words**	*'You can always text us back for more help – text us with one of these trigger words and texts with instant support will appear on your phone: 'BAD DAY', 'SLIP-UP', 'TIRED' and 'CRAVE'.*	Bad day: *'Everyone has these type of days. Don't be hard on yourself! Press delete for the day so far and start afresh!'*
		Slip-up: *'Don't let one slip-up discourage you. You have the ability to pick up right where you left off!'*
		Tired response: *'Fresh air can work wonders! Getting out of the house for a while can help you think more clearly and boost your energy'.*
		Crave response: *'Tried brushing your teeth or chewing a piece of sugar free gum? It might work!'*
**Weight management when breastfeeding**	*'Breastfeeding can make you hungry. Just fill up on the good stuff (fruit and vegetables, wholegrain cereals) and the good eating habits will stay with you even when the breastfeeding stops :) '*	N/A

For peer review only

**Smoking cessation and weight management**	*'It is only normal to have cravings! Why not exercise to combat it? Try a short walk (even for just 5 mins) if a craving hits, the distraction can really help.'*	N/A
**Intervention "buddy system"**	*'Welcome to WEEK 34. Many people that have lost weight and kept it off have done so with support from family and friends! You can opt in to get your friend/partner/family member to receive SMS texts for extra support - text 'SUPPORT' followed by the person's mobile number to sign up (ask their permission first). E.g. Support 0780000000'.*	N/A
**Active control**	*'1, 2, 3! Your toddler might know how to count to 3 or more...or they might get some of the numbers mixed up. Whatever stage they are at, just keep encouraging them by counting from 1 to 10'.*	N/A

For peer review only - http://bmjopen.bmj.com/site/about/guidelines.xhtml

Supplemental appendix 2- Informed consent materials [32]

a) Participant Information Sheet

PARTICIPANT INFORMATION SHEET

The Supporting MumS (SMS) study

We would like to invite you to take part in this research study which is taking place in Northern Ireland, England, Scotland and Wales.

Before you decide whether to take part, it is important that you understand why we are doing this research and what it will involve.

Please take time to read the information carefully and discuss it with friends/relatives, if you wish. If you have any questions, please contact us using the details given at the end of the sheet.

For peer review only - http://bmjopen.bmj.com/site/about/guidelines.xhtml

Why are we doing this study?

Welcoming a baby brings lots of joy and happiness but being a new mum also means life is a bit busy! There may be areas where mums could benefit from more support. Mums often talk about having a baby as a time when their weight started to creep up and how they would welcome support with this. We are interested in finding out if text messages can help support weight management in the first two years after having a baby.

For this study, the term 'mum' or 'mother' includes people who do not identify as women but are pregnant or have given birth. The term 'your child' means your youngest child at the time of signing up to the study.

Why have I been asked to take part?

You have been chosen to take part, as you are over 18 years old and have had a baby within the past two years and have a body mass index over 25kg/m^2.

Do I have to take part?

No, it is up to you whether or not you want to take part. If you do decide to take part, you are free to leave the study at any stage without giving a reason for doing so. Your usual health care will not be affected at any time.

What will happen if I take part?

After you read this leaflet we will contact you to discuss the study in more detail and answer any further questions you may have.

If you decide to take part, you will be asked to sign the consent form for the study and complete some study measurements (described below). You will then receive text messages to your mobile phone for 12 months. The number of text messages you receive each week will vary between 3 and 14 per week. The text messages are delivered to you by a secure text message system owned and operated by the London School of Hygiene and Tropical Medicine.

Mums who take part in the study will get text messages on **ONE** of the two topics shown below. It is important that you understand that the topic is randomly chosen for you and that there is a 50:50 chance you will get texts on either topic 1 or topic 2:

1. Information and advice on making healthy food choices and keeping physically active to help you lose weight.

OR

2. Information and advice on your child's health and development.

For peer review only - http://bmjopen.bmj.com/site/about/guidelines.xhtml

What information will you collect from me if I take part?

We would meet with you 4 times (during a 24 month period) to collect some measurements and information. These visits will take place at the start of the study, at 6 months, at 12 months (when the text messages stop) and at 24 months (12 months after you stopped receiving the text messages).

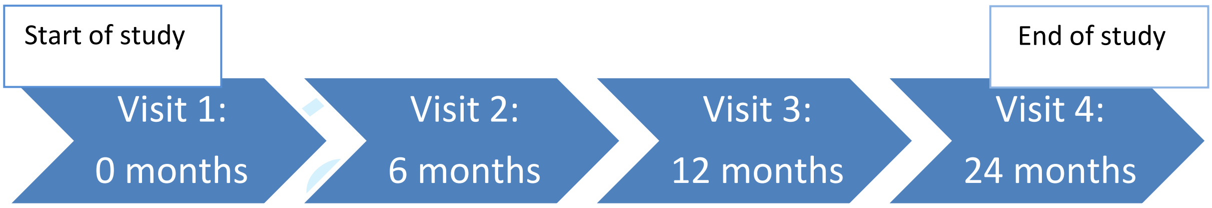

For the visits, the researcher will either visit you at your home or you can come and meet the researcher at a convenient location such as a University building or a local community venue; whichever is most convenient for you.

You will receive a £25 voucher on completion of each visit in recognition of your time to complete the research measures. This will be a total of £100 over the 24-month study, if all visits are completed. Each visit will last approximately 1 hour.

At each visit we will collect the following information:

a) We will measure your height (only at visit 1) and will ask you to step on the scales to collect information on weight and will measure your waist circumference (this will take about 10 minutes).

b) We will ask you to complete a questionnaire booklet about your physical and mental health, lifestyle and wellbeing. The booklet will take approximately 35 minutes to complete. You will have the option of:
   - completing a printed questionnaire booklet in your own time and posting it back to us in a stamped addressed envelope we provide; OR
   - completing the questionnaire online by accessing a link we give you; OR
   - completing the questionnaire with the researcher at the study visit or over the phone.

When (a) and (b) above has been collected from you, we will arrange for the £25 voucher to be sent to you.

We would also ask you to consider taking part in a short (approximately 20 minutes) telephone interview twice during the study (at the 6 month visit and the 12 month visit) to help us understand how you found the text messages. *This part is optional.*

For peer review only - http://bmjopen.bmj.com/site/about/guidelines.xhtml

Linking to routine health-related data in the future (this part is optional)

We would also ask you to consider allowing us to collect health-related data about you and your child in the future. We would do this by linking with organisations that collect data about the services they provide to women and children. For example, the NHS keeps records, known as 'routine data' about how we use different services such as the health service.

Routine data is stored electronically on different systems. To obtain your routine data from other systems, we would share some personally-identifiable details such as name, date of birth, address and NHS number to the organisations that manage these systems and they will send us data back. This will all be done using secure data transfer systems that have been set up carefully to keep your information safe.

Doing this helps us to understand if a study like this has any longer-term benefits and how we might improve health services in the future.

We may access routine data from these sources:

- Health records such as GP and dental records, maternity and health visiting records and disease registers
- Local authority and social care
- Family/children's centres
- Education or school
- National child measurement programme
- Voluntary organisations

For example, from maternity records we may collect information on any further pregnancies you have and from GP records we may collect information on any diagnosed conditions such as high blood pressure or type 2 diabetes.

If you consent to us linking to routine health-related data on the consent form, we will ask you for your NHS number. This number will only be stored by QUB for 15 years and will only be used for this purpose. It will be stored securely, separately from your other data, in a password protected, encrypted file on computers that are only accessible to authorised members of the research team. If at any time you want us to remove your NHS number from our records, you can do so by contacting us as per details given at the end of this information sheet. Otherwise, it will be removed from our records after 15 years. An example of how we would link your routine data is shown below.

You do not have to consent to data linkage to participate in this study. This part is optional.

For peer review only - http://bmjopen.bmj.com/site/about/guidelines.xhtml

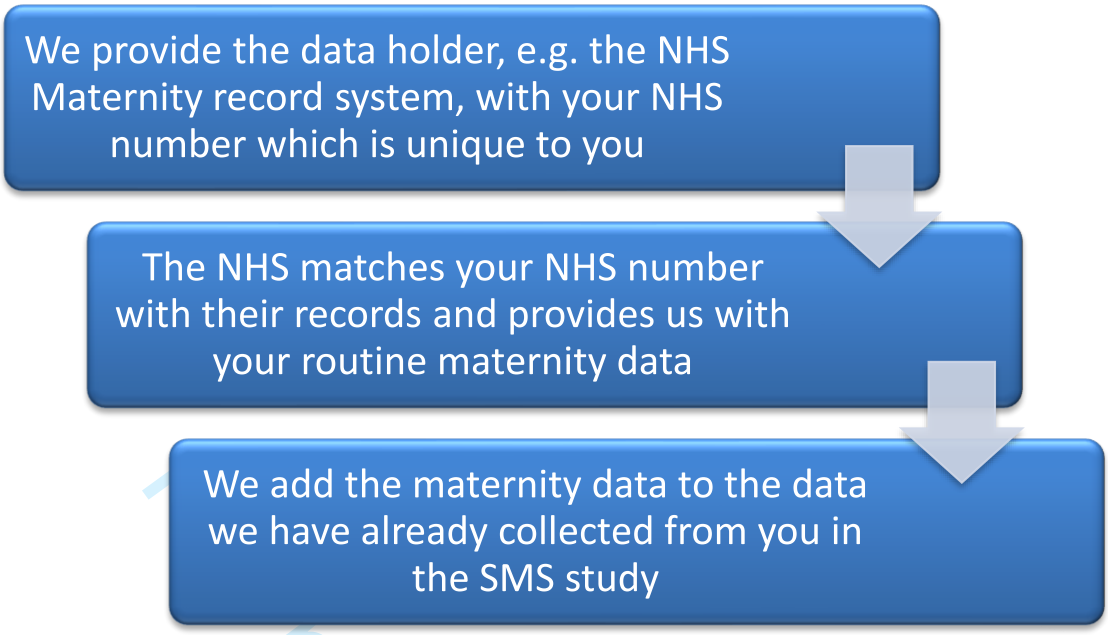

Example of routine data linkage process

What are the possible advantages and disadvantages of taking part?

By taking part in this research you will be helping us find the best ways to support new mums. In the study questionnaires and interviews, we will collect information about your postnatal physical and mental health. This information will be used to help us understand how we might improve services for women after they have a baby. Some of the messages you receive may be helpful for you and/or your baby by helping you to lose weight or by providing you with information about child health and development. We do not anticipate any risks from taking part in this study.

How will we use information about you?

We will need to use information from you for this research project. This information will include your name, address, NHS number, date of birth, telephone number, email address and date of birth of your youngest child. People will use this information to do the research or to check your records to make sure that the research is being done properly.

People who do not need to know who you are will not be able to see your name or contact details. Your data will have a code number instead.

We will keep all information about you safe and secure.

For peer review only - http://bmjopen.bmj.com/site/about/guidelines.xhtml

Once we have finished the study, we will keep some of the data so we can check the results. We will write our reports in a way that no-one can work out that you took part in the study.

Will my taking part in the study be kept confidential?

Yes, all your data will be treated with the strictest confidence and your details will not be shared with anybody outside of the research team, unless there is a serious risk of harm to you or others.

Any information collected from you will be stored securely on password protected files on password protected computers that only the research team can access. Hard copies of documents will be kept in locked filing cabinets in locked offices that are only accessible to the research team and are located in a building that is locked outside normal working hours.

Any interviews conducted over the telephone will be audio-recorded and the recording will then be typed up for research purposes. The typed transcript will not contain any names and will be labelled with your unique study number. The recording will be destroyed once the typed transcript is prepared.

Study data will be kept separate from personal information (such as name and address). Only members of the research team will have access to view identifiable data. However, in some instances, inspectors from regulatory authorities may need to access data for checking the quality of the research. All members of the research team and regulatory bodies are trained in data protection and will comply with the requirements of data protection legislation.

Once the study is complete and it is no longer necessary to keep identifiable information or contact details, we will destroy our records of this personal information.

The study questionnaire asks some questions about mental and physical health. If we have any concerns that your responses to such questions may indicate you are at risk of postpartum depression, we will inform you and your GP by letter as part of our duty of care to you. If at any stage we have concerns that you, or someone else, is at risk of harm then we are obliged to tell the relevant services, for example your GP or social services.

What are your choices about how your information is used?

- You can stop being part of the study at any time, without giving a reason, but we will keep information about you that we already have.

For peer review only - http://bmjopen.bmj.com/site/about/guidelines.xhtml

- We need to manage your records in specific ways for the research to be reliable. This means that we won't be able to let you see or change the data we hold about you.
- If you agree to take part in this study, you will have the option to take part in future research using your data saved from this study.

Where can you find out more about how your information is used?

You can find out more about how we use your information:

- at www.hra.nhs.uk/information-about-patients/
- in our privacy notice from go.qub.ac.uk/SMSstudy or ask the research team for a copy
- by asking one of the research team
- by sending an email to info.compliance@qub.ac.uk, or
- by ringing us on 07341 888415.

How will I find out the results of this project?

When the study is finished and the study information has been analysed, we will send you a summary of the results.

Who is organising and funding the research?

For this study we will be recruiting women from all four countries in the UK. The work is being led by Professor Michelle McKinley from Queen's University Belfast. Researchers from Universities in London, Cardiff, Stirling and the Bradford Teaching Hospitals NHS Foundation Trust are leading recruitment in their areas.

Mums have helped us design our text messages and advise on how the study is conducted and will continue to do so.

The study is funded by National Institute of Health Research and the Public Health Agency Northern Ireland.

Has this study been approved for safety by an ethics committee? Is this study safe?

This study has been reviewed and approved by the West of Scotland Research Ethics Committee 4 and IRAS ID 305557.

For peer review only - http://bmjopen.bmj.com/site/about/guidelines.xhtml

What will happen if I don't want to carry on with the study?

You can withdraw from the study at any time, without giving a reason by contacting the research team (contact details at the end of this leaflet). If you do decide to withdraw from the study, we will use the data collected up to that point, but we will not collect any more data.

What if something goes wrong?

There are no special compensation arrangements. Queen's University Belfast will provide indemnity for this study. If you are harmed due to someone's negligence, then you may have grounds for legal action but you may have to pay for it.

What if there is a problem?

If you have a concern about any aspect of this study, you can speak to the researchers who will do their best to answer your questions (contact details on the last page).

Should you remain unhappy and wish to make a formal complaint, you can contact:

Research Governance Team at Queen's University Belfast,
63 University Road, Belfast
BT7 1NF
(Telephone: 028 9097 2529; Email: researchgovernance@qub.ac.uk).

Contact for Further Information:

Researchers' Name, Address, Email address, Telephone (site specific)	Dr Dunla Gallagher<br>SMS Trial Manager<br>Centre for Public Health<br>Queen's University Belfast<br>School of Medicine, Dentistry & Biomedical Sciences<br>Institute of Clinical Sciences (Block B)<br>Grosvenor Road<br>Belfast, N.Ireland<br>BT12 6BA<br>Tel: 07341 888415<br>Email: d.gallagher@qub.ac.uk

Please ask us if there is anything that is not clear or if you would like more information.

For peer review only

b) Privacy Notice

PRIVACY NOTICE

The Supporting MumS (SMS) study

Queen's University Belfast, Bradford Teaching Hospitals NHS Foundation Trust, Cardiff University, the University of Stirling, and the London School of Hygiene & Tropical Medicine ("we", "us" and "our") are committed to protecting your personal data.

The notice is addressed to individuals who are taking part in the research study entitled 'The Supporting MumS (SMS) study' ("you" and "your").

This Privacy Notice tells you why we need to collect personal information about you, what we will do with it, and how we will look after it. It also tells you about your legal rights in relation to your Personal Data.

If you have any questions about this privacy notice, please contact us. Contact details are provided below.

WHO WE ARE

1. We are a group of expert researchers from the organisations listed above. The study is led by Queen's University Belfast and is funded by National Institute of Health Research and the Public Health Agency Northern Ireland.

2. All our research is underpinned by policies and procedures that ensure we comply with regulations and legislation that govern the conduct of research. This includes data protection legislation: the UK General Data Protection Regulation (GDPR) and the Data Protection Act 2018 (DPA). In the case of health and social care research, which should serve the public interest, where we have to demonstrate that our research serves the interests of society as a whole.

HOW YOUR PERSONAL DATA IS COLLECTED

3. **Information you provide:** When you consent to take part in the SMS study we will ask for information about you, such as your name, address, NHS number, date of birth, telephone number, email address and date of birth of your youngest child. This is known as your "Personal Data". We may also ask you for some special categories of information (for example information about your physical and mental health). This is known as your "Sensitive Personal Data" and has additional protections.

4. **Data from other sources:** We will also collect information about you from other sources and this also forms part of "Personal Data" and "Sensitive Personal Data". This includes information from:

For peer review only - http://bmjopen.bmj.com/site/about/guidelines.xhtml

- the system we are using to send and receive text messages for the study, for example we will automatically collect the replies you send to the text messages. This system is owned and operated by the London School of Hygiene & Tropical Medicine.
- routine health-related data (if you have consented to this option) such as NHS records, GP and dental records, maternity and health visiting records and disease registers. To obtain your routine data from these systems, we will share some personally-identifiable details such as name, date of birth, address and NHS number to each of these organisations and they will send us your data back. This will all be done using secure data transfer systems that have been set up carefully to keep your information safe and complies with regulations and legislation governing the conduct of research.

HOW WE USE YOUR PERSONAL DATA

5. We use your Personal Data and Sensitive Personal Data in the following ways:
   - to contact you to invite you to take part in the study and to discuss research-related queries. If you do not consent to take part in the study, your personal data, such as contact details (e.g. name, address, phone number), will be confidentially destroyed and will not be retained by us.
   - after consent to take part is provided, personal data is used to contact you to arrange and conduct study visits, to request information or to send you relevant study documents or equipment and to inform you about any relevant study developments.
   - access to, and sharing of, special category and sensitive personal data is controlled very carefully. Sensitive personal data is collected and pseudonymised – this means each person who takes part in this study will be given a unique study number and this unique study number is used on all the study paperwork and in study databases. Only authorised members of the SMS research team will be able to link your study number to your name, contact details and NHS number.

6. In order to protect your rights and freedoms when using your personal information for research and to process special category information we have special safeguards in place to protect your information, and all information is kept in line with our policies and regulatory requirements.

7. In addition to the above safeguards, data protection legislation requires us to meet the following standards when we conduct research with your personal information:
   - the research will not cause damage or distress to someone (e.g., physical harm, financial loss or psychological pain).
   - the research is not carried out in order to do or decide something in relation to an individual person, unless the processing is for medical research approved by a research ethics committee.
   - the Data Controller has technical and organisational safeguards in place (e.g. appropriate staff training and security measures).
   - if processing a special category of data, this must be subject to a further public interest test to make sure this particularly sensitive information is required to meet the research objectives.

For peer review only - http://bmjopen.bmj.com/site/about/guidelines.xhtml

LEGAL BASIS FOR COLLECTING AND USING YOUR PERSONAL DATA

8. We will only use your Personal Data if we have valid reasons for doing so. These reasons are known as our "legal basis for processing".

9. In the context of research, the lawful basis upon which we will process your personal information is usually where "Processing is necessary for the performance of a task carried out in the public interest or in the exercise of official authority vested in the controller" (Article 6 of GDPR).

10. We also collect and use more sensitive personal information (Special Category data) where "the processing is necessary for archiving purposes in the public interest, scientific or historical research purposes or statistical purposes… which shall be proportionate to the aim pursued, respect the essence of the right to data protection and provide for suitable and specific measures to safeguard the fundamental rights and the interests of the data subject". (Article 9 of GDPR).

WHO WE SHARE YOUR DATA WITH

11. In line with our Data Protection Policy and Procedures we can share your information, including Personal Data and Sensitive Personal Data, with the following parties for the following research purposes:
    - London School of Hygiene and Tropical Medicine (LSHTM) text message system – to allow you to receive and reply to the text messages (the LSHTM is a study collaborator)
    - Designated research personnel for the purposes of achieving the research outcomes
    - The Northern Ireland Clinical Trials Unit (NICTU) for the purposes of entering your completed questionnaires onto a study database (NICTU is a study collaborator)
    - Qualtrics research software supplier for the purposes of the online completion of the study questionnaires (privacy notice can be viewed here - https://www.qualtrics.com/support/survey-platform/getting-started/data-protection-privacy/).
    - Transcription services for the purpose of transcribing recorded interviews (if consent provided for this optional element)
    - Organisations in-charge of the collection and administration of routine data for the purposes of achieving the research outcomes (if consent provided for this optional element)

12. In all cases, information shared will be on a need to know basis, not excessive and with all appropriate safeguards in place to ensure the security of your information.

13. When we use third parties (known as data processors) to carry out a task on our behalf, such as a transcription service as indicated above, we have contractual terms, policies and procedures to ensure confidentiality is respected.

DATA PROCESSING OUTSIDE EUROPE

14. We will not transfer your Personal Data and Sensitive Personal Data outside of the United Kingdom and European Economic Area.

For peer review only - http://bmjopen.bmj.com/site/about/guidelines.xhtml

HOW LONG YOUR INFORMATION WILL BE KEPT?

15. Information where you can be identified will be kept for a minimum amount of time and in accordance with research objectives. Researchers will de-identify information, i.e. anonymise or pseudonymise, as soon as possible as described in point 5.

16. We will keep your Personal Data and Sensitive Personal Data for up to 10 years from when the research has been completed to allow for full and final publication of the research results. If you have provided consent (this is optional), we will retain your record of consent and personal data (name, telephone number, email address, address) for the purpose of conducting longer-term follow-up for the SMS study or to inform you about future studies related to health and lifestyle that we may be conducting. If you have consented, we will retain your NHS number for data linkage purposes. You can decline to receive communications at any stage. We will only keep your information if we need it for one of the reasons described above.

17. We place great importance on the security of the Personal Data that we hold, including the use of physical, technological and organisational measures to ensure your information is protected from unauthorised access and against unlawful processing, accidental loss, alteration, disclosure, destruction and damage.

YOUR RIGHTS

18. The DPA provides you with a number of legal rights in relation to your Personal Data, including the right:

- to request access to your Personal Data;
- to request correction of your Personal Data that is wrong or incomplete;
- to request erasure or the restriction of processing of your Personal Data;
- to request the transfer of your Personal Data in a structured; commonly used machine-readable format;
- not to be subject to automated decision making; and
- to withdraw your consent.

19. If you wish to exercise any of the rights set out above, or require further information about any of the rights, please contact us.

20. There may also be times where we cannot stop using your Personal Data when you ask us to, but we will tell you about this if you make a request.

For peer review only - http://bmjopen.bmj.com/site/about/guidelines.xhtml

CONTACTING US

21. If you have any questions or comments about this privacy notice, please contact the SMS team:

Dr Dunla Gallagher
SMS Trial Manager
Centre for Public Health
Institute Clinical Science A
School of Medicine, Dentistry & Biomedical Sciences
Queen's University Belfast
d.gallagher@qub.ac.uk

Professor Michelle McKinley
SMS Chief Investigator
Centre for Public Health
Institute Clinical Science A
School of Medicine, Dentistry & Biomedical Sciences
Queen's University Belfast
m.mckinley@qub.ac.uk

Alternatively, you can contact:
Data Protection Officer
Information Compliance Unit
Lanyon South
Queen's University Belfast
University Road
BT7 1NN
Info.compliance@qub.ac.uk

COMPLAINTS

22. You have the right to complain about how we treat your Personal Data and Sensitive Personal Data to the Information Commissioner's Office (ICO). The ICO can be contacted at:

Information Commissioner's Office
Wycliffe House
Water Lane
Wilmslow
Cheshire
SK9 5AF

CHANGES TO THIS NOTICE

23. We may update this Privacy Notice from time to time. We will notify you of the changes where we are required by law to do so.

For peer review only - http://bmjopen.bmj.com/site/about/guidelines.xhtml

c) Study Consent Form

CONSENT FORM

The Supporting MumS (SMS) study

Screening Number: __ __ __ __ __ Participant ID number: __ __ __ __ __

		Please **initial** box
1.	I confirm that I have read and understood the Participant Information Sheet dated 05/04/2022 (Version 4.0) and privacy notice dated 30/11/2021 (Version 1.0) for the above study and have been given copies to keep.	
2.	I have had the opportunity to consider the information, ask questions and have had these answered to my satisfaction.	
3.	I understand that my participation is voluntary and that I am free to withdraw at any time, without giving any reason and without my medical care or legal rights being affected.	
4.	I understand that information collected about me for the study (including personally identifiable information) may be looked at by responsible individuals in the study team and regulatory authorities supervising the study.	
5.	I understand that all information collected about me during the course of the research will be kept strictly confidential and processed in compliance with data protection legislation.	
6.	I understand that some of the data collected about me will be processed by third parties (known as data processors), such as software and transcription companies, and that contractual terms, policies and procedures will be put in place to ensure confidentiality is respected.	
7.	I give permission for my GP to be informed of any concerning medical issues detected during study visits.	
8.	I am aware of the potential risks and benefits of this research study, as described in the Participant Information Sheet and discussed with the researcher.	
9.	I understand that I will not be identifiable in any published report using data from this study.	
10.	I agree to my contact details being kept so I can be informed of the study findings.	
**11.**	**I consent to take part in the above study.**	

For peer review only - http://bmjopen.bmj.com/site/about/guidelines.xhtml

	**OPTIONAL ELEMENTS (please initial box if you consent)**	
12.	I am willing to be contacted to take part in two short (20-30 minute) telephone interviews during the study to provide feedback to the research team.	☐
13.	I am willing to be contacted in the future about a follow-up to this study.	☐
14.	I consent to be contacted by the University about future research studies related to diet or lifestyle and health for which I may be eligible.	☐
15.	I consent to my personal health information, including my NHS number and date of birth, being released to organisations that hold routine health-related data so they can locate information about me that is held in their database.	☐
16.	I consent to organisations holding my routine health-related data performing data linkage and releasing the information about me from its databases to the SMS Study research team.	☐

Name of Participant (Block capitals)	Signature	Date

To be completed by the Principal Investigator or nominee

I, the undersigned, have taken the time to fully explain to the above patient the nature and purpose of the study in a way that they could understand. I have explained the risks involved as well as the possible benefits. I have invited them to ask questions on any aspect of the study that concerned them.

Name of Researcher (Block capitals)	Signature	Date

Researchers' Name, Address, Email address, Telephone (site specific)	Dr Dunla Gallagher<br>SMS Trial Manager<br>Centre for Public Health, Queen's University Belfast<br>School of Medicine, Dentistry & Biomedical Sciences<br>Institute of Clinical Sciences (Block B)<br>Grosvenor Road, Belfast, N.Ireland, BT12 6BA<br>Tel: 07341 888415<br>Email: d.gallagher@qub.ac.uk

For peer review only

Supplemental appendix 3- Participant self-report questionnaires (baseline and 12 months)

Supporting MumS (SMS) study

Questionnaire Booklet

Month 0

**Screening ID:**	_ _ _ _ _
**Participant ID:**	_ _ _ _ _
**Researcher name:**	
**Today's date:**	_ _ / _ _ / _ _ _ _<br>**e.g. 05 / 01 / 2017**

For peer review only - http://bmjopen.bmj.com/site/about/guidelines.xhtml

About this questionnaire booklet

Thank you for taking the time to fill out this questionnaire booklet.

The questionnaire booklet is made up of questions that will help us find out more about your experience of the postpartum period. Please complete the questions as accurately as you can. **There are no right or wrong answers**. If there are any questions you do not wish to answer, you can leave them blank. Note that your responses will be **anonymous,** this means your name will never be given with your responses.

The questionnaire booklet is split up into the short sections shown below. You can fill it all out at once, or do it in chunks, as your time allows.

**Section 1**	**Health and well-being**	**Pages 3-5**
**Section 2**	**Health resources**	**Pages 6-8**
**Section 3**	**Eating and activity approaches**	**Pages 9-13**
**Section 4**	**Social support**	**Page 14-15**
**Section 5**	**Lifestyle behaviours and attitudes**	**Page 16-18**
**Section 6**	**Food patterns**	**Page 19-23**
**Section 7**	**Physical activity**	**Page 24-26**
**Section 8**	**Infant feeding**	**Page 27-28**
**Section 9**	**Mood**	**Page 29-33**
**Section 10**	**Self-esteem**	**Page 34**
**Section 11**	**Sleep**	**Page 35-38**

For peer review only - http://bmjopen.bmj.com/site/about/guidelines.xhtml

Section 1: Health and well-being

For questions 1-5, under each heading, please tick (✓) the **ONE** box that best describes your health TODAY.

**1. Mobility**	
I have no problems in walking about	☐
I have slight problems in walking about	☐
I have moderate problems in walking about	☐
I have severe problems in walking about	☐
I am unable to walk about	☐

**2. Self-care**	
I have no problems washing or dressing myself	☐
I have slight problems washing or dressing myself	☐
I have moderate problems washing or dressing myself	☐
I have severe problems washing or dressing myself	☐
I am unable to wash or dress myself	☐

**3. Usual Activities** *(e.g. work, study, housework, family or leisure activities)*	
I have no problems doing my usual activities	☐
I have slight problems doing my usual activities	☐
I have moderate problems doing my usual activities	☐
I have severe problems doing my usual activities	☐
I am unable to do my usual activities	☐

**4. Pain/ Discomfort**	
I have no pain or discomfort	☐
I have slight pain or discomfort	☐
I have moderate pain or discomfort	☐
I have severe pain or discomfort	☐
I have extreme pain or discomfort	☐

For peer review only - http://bmjopen.bmj.com/site/about/guidelines.xhtml

**5. Anxiety/ Depression**	
I am not anxious or depressed	☐
I am slightly anxious or depressed	☐
I am moderately anxious or depressed	☐
I am severely anxious or depressed	☐
I am extremely anxious or depressed	☐

6. We would like to know how good or bad your health is TODAY.

This scale is numbered from 0 to 100.

100 means the <u>best</u> health you can imagine.

0 means the <u>worst</u> health you can imagine.

Please mark an X on the scale to indicate how your health is TODAY.

Now, write the number you marked on the scale in the box below.

Your health today = ☐

The best health you can imagine

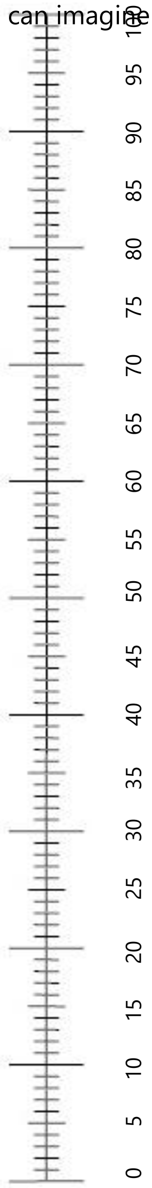

The worst health you can imagine

For peer review only - http://bmjopen.bmj.com/site/about/guidelines.xhtml

**7. Feeling settled and secure** *(Please tick (✓)* ***one*** *box that best describes your overall quality of life at the moment).*	
I am able to feel settled and secure in **all** areas of my life	☐
I am able to feel settled and secure in **many** areas of my life	☐
I am able to feel settled and secure in **a few** areas of my life	☐
I am unable to feel settled and secure in **any** areas of my life	☐

**8. Love, friendship and support** *Please tick (✓)* ***one*** *box that best describes your overall quality of life at the moment).*	
I can have **a lot** of love, friendship and support	☐
I can have **quite a lot** of love, friendship and support	☐
I can have **a little** love, friendship and support	☐
I **cannot** have **any** love, friendship and support	☐

**9. Being independent** *Please tick (✓)* ***one*** *box that best describes your overall quality of life at the moment).*	
I am able to be **completely** independent	☐
I am able to be independent in **many** things	☐
I am able to be independent in **a few** things	☐
I am **unable** to be at all independent	☐

**10. Achievement and progress** *Please tick (✓)* ***one*** *box that best describes your overall quality of life at the moment).*	
I can achieve and progress in **all** aspects of my life	☐
I can achieve and progress in **many** aspects of my life	☐
I can achieve and progress in **a few** aspects of my life	☐
I cannot achieve and progress in **any** aspects of my life	☐

**11. Enjoyment and pleasure** *Please tick (✓)* ***one*** *box that best describes your overall quality of life at the moment).*	
I can have **a lot** of enjoyment and pleasure	☐
I can have **quite a lot** of enjoyment and pleasure	☐
I can have **a little** enjoyment and pleasure	☐
I **cannot** have **any** enjoyment and pleasure	☐

For peer review only - http://bmjopen.bmj.com/site/about/guidelines.xhtml

Section 2: Health resources

1. In the last 3 months, have you seen any health professional at your GP surgery? (this includes phone/online or face-to-face appointments) *(Please tick (✓) **one** box).*

Yes	☐
No	☐

If YES, how many times were you seen by: *(Please enter a number)*

GP	
Practice nurse	
Other (please state)	
Other (please state)	

2. In the last 3 months, have you attended an Accident and Emergency (Casualty) department? *(Please tick (✓) **one** box).*

Yes	☐	If YES, how many times?	
No	☐		

3. In the last 3 months, have you attended hospital as an out-patient? *(Please tick (✓) **one** box).*

Yes	☐	If YES, how many times?	
No	☐		

4. In the last 3 months, have you attended hospital as an in-patient? *(Note: do not include stays for, or immediately after, giving birth)* *(Please tick (✓) **one** box).*

Yes	☐	If YES, how many times?	
		If YES, how many nights did you spend in hospital in the last 3 months in total?	
No	☐		

5. In the last 3 months, have you received any prescriptions for medicine? *(Please tick (✓) **one** box).*

Yes	☐	If YES, how many prescriptions have you received?	
No	☐		

For peer review only - http://bmjopen.bmj.com/site/about/guidelines.xhtml

6. In the last 3 months, have you purchased any over-the-counter medications or lifestyle products (e.g. slimming aids, vitamin supplements)? *(Please tick (✓)* ***one*** *box).*

Yes	☐	If YES, please state total amount spent in last 3 months	£
No	☐		

582

7. In the last 3 months have you paid for any services or clubs for the specific purpose of helping you with your lifestyle - for example slimming clubs, health clubs, gyms, swimming pools, exercise classes? *(Please tick (✓)* ***one*** *box).*

Yes	☐	If YES, approximately how much have you spent in <u>total</u> on all of these services/clubs in the <u>last 3 months?</u>	£
No	☐		

8. Please think about <u>last week's</u> food and drink purchases for your household (i.e. you and your family) and tell us the cost to your household of the following: *(Please write '**0**' if you don't purchase a specific type of food/product) .*

Type of food and drink purchased	Weekly Cost to you (your household)
Food and non-alcoholic drinks (e.g. supermarket shopping)	£
Alcoholic drinks e.g. wine & beer	£
Takeaway meals and snacks eaten AT HOME e.g. pizza delivery	£
Meals, snacks and drinks CONSUMED AWAY FROM HOME (e.g. restaurant)	£
Cigarettes or vaping products	£

583

9. Have you previously purchased any apps for improving your fitness/health or wellbeing? *(Please tick (✓)* ***one*** *box).*

Yes	☐	If YES, Please state the total amount you spent on fitness/health apps in the <u>last year?</u>	£
No	☐		

584

For peer review only - http://bmjopen.bmj.com/site/about/guidelines.xhtml

10. On average, <u>per day</u> how much time have you spent exercising/walking in the last 3 months in total?

*(Please answer in hours **AND** minutes. E.g. If you did 30 minutes of exercise per day, you can enter '0' under Hours and '30' under the Minutes column)*

On average per day:	Hours	☐	Minutes	☐

585

11. Has your employment status changed in the last three months? *(Please tick (✓) **one** box).*

Yes	☐
No	☐

586

587

588

589

For peer review only

For peer review only - http://bmjopen.bmj.com/site/about/guidelines.xhtml

Section 3: Eating and activity approaches

This section asks you about your diet. *(Please circle **one** number in each row to indicate how much you agree or disagree with the following statements).*

**1. Eating healthier is something…**	**Strongly Disagree**						**Strongly Agree**
I do automatically	1	2	3	4	5	6	7
I do without having to consciously remember	1	2	3	4	5	6	7
I do without thinking	1	2	3	4	5	6	7
I start doing before I realise I'm doing it	1	2	3	4	5	6	7

**2. Over the next few months I intend to…**	**Strongly Disagree**						**Strongly Agree**
eat a healthy diet (e.g. reduce portion size, eat less sugary and high fact snacks and eat more fruit and vegetables)	1	2	3	4	5	6	7
be physically active (e.g. by walking more or taking exercise classes)	1	2	3	4	5	6	7

**3. I already have concrete plans…**	**Strongly Disagree**						**Strongly Agree**
on how to eat a healthy diet	1	2	3	4	5	6	7
on when to eat a healthy diet	1	2	3	4	5	6	7

**4. I already have concrete plans…**	**Strongly Disagree**						**Strongly Agree**
on when I need to be especially careful to maintain my healthy diet	1	2	3	4	5	6	7
about what to do in difficult situations to stick to my healthy diet	1	2	3	4	5	6	7
on how I will cope if I slip back into old eating habits	1	2	3	4	5	6	7

For peer review only - http://bmjopen.bmj.com/site/about/guidelines.xhtml

**5. I am confident that I can stick to a healthy diet even…**	**Strongly Disagree**					**Strongly Agree**
if I have to learn a lot about nutrition	1	2	3	4	5	6	7
if I initially have to watch out in many situations	1	2	3	4	5	6	7
if I have to start all over again several times until I succeed	1	2	3	4	5	6	7
if I initially have to make plans	1	2	3	4	5	6	7
if initially food doesn't taste as good	1	2	3	4	5	6	7
if I initially don't get much support	1	2	3	4	5	6	7
if it takes a long time to get used to it	1	2	3	4	5	6	7
if I have worries and troubles	1	2	3	4	5	6	7
if my partner/ my family don't change their nutrition habits	1	2	3	4	5	6	7
if I am tired	1	2	3	4	5	6	7
if I am stressed out	1	2	3	4	5	6	7
if I don't lose weight initially	1	2	3	4	5	6	7

6. In spite of good intentions when losing weight, slip-ups may occur. Imagine you relapse back into your old eating habits. How confident are you about restarting your new healthy eating habits?

**I am sure I can start my new healthy eating habits again regularly, even if I…**	**Strongly Disagree**						**Strongly Agree**
have several bad days in a row	1	2	3	4	5	6	7
lose my motivation to keep up my new healthy eating habits	1	2	3	4	5	6	7
have had a relapse and have fallen back into old eating habits for several weeks	1	2	3	4	5	6	7

For peer review only - http://bmjopen.bmj.com/site/about/guidelines.xhtml

7. For the next few questions:

'Tempting foods' are any food you want to eat more of than you think you should.
'Eating intentions' refer to the way you are aiming to eat, for example you may intend to avoid tempting foods or eat healthy foods.

*(Please tick (✓) **one** box for each of the following statements).*	**Never**	**Rarely**	**Sometimes**	**Often**	**Always**
I give up too easily on my eating intentions	☐	☐	☐	☐	☐
I'm good at resisting tempting food	☐	☐	☐	☐	☐
I easily get distracted from the way I intend to eat	☐	☐	☐	☐	☐
If I am not eating in the way I intend to I make changes	☐	☐	☐	☐	☐
I find it hard to remember what I have eaten throughout the day	☐	☐	☐	☐	☐

<u>This section asks about your physical activity</u>. *(Please circle **one** number in each row to indicate how much you agree or disagree with the following statements).*

**1. Being active every day is something…**	**Strongly Disagree**						**Strongly Agree**
I do automatically	1	2	3	4	5	6	7
I do without having to consciously remember	1	2	3	4	5	6	7
I do without thinking	1	2	3	4	5	6	7
I start doing before I realise I'm doing it	1	2	3	4	5	6	7

**2. I already have concrete plans…**	**Strongly Disagree**						**Strongly Agree**
on when to be physically active	1	2	3	4	5	6	7
on where to be physically active	1	2	3	4	5	6	7
on how to be physically active	1	2	3	4	5	6	7
on how often be physically active	1	2	3	4	5	6	7
on who I can be physically active with	1	2	3	4	5	6	7
**3. I already have concrete plans…**	**Strongly Disagree**						**Strongly Agree**

For peer review only - http://bmjopen.bmj.com/site/about/guidelines.xhtml

about what to do if something gets in the way	1	2	3	4	5	6	7
about what to do if I miss a physical activity session	1	2	3	4	5	6	7
about what to do in difficult situations to stick to my physical activity intentions	1	2	3	4	5	6	7
for times when I will need to be extra careful to stay committed	1	2	3	4	5	6	7

4a. Certain barriers make it hard to begin physical activity. How sure are you that you can begin exercising regularly?

**I am sure that...**	**Strongly Disagree**						**Strongly Agree**
I can change to a physically active lifestyle	1	2	3	4	5	6	7
I can be physically active once a week	1	2	3	4	5	6	7
I can be physically active at least 3 times a week for 30 minutes	1	2	3	4	5	6	7

**4b. I am sure I can start being physically active immediately, even if...**	**Strongly Disagree**						**Strongly Agree**
the planning for this takes a lot of time and effort	1	2	3	4	5	6	7
I have to force myself to start immediately	1	2	3	4	5	6	7
I have to push myself	1	2	3	4	5	6	7

For peer review only - http://bmjopen.bmj.com/site/about/guidelines.xhtml

**5**. Are you confident that you can manage staying physically active?							
**I am sure I can keep being physically active regularly, even if...**	**Strongly Disagree**						**Strongly Agree**
it takes me a long time to make a habit	1	2	3	4	5	6	7
I am worried and troubled	1	2	3	4	5	6	7
I am tired	1	2	3	4	5	6	7
I am stressed out	1	2	3	4	5	6	7
I don't lose weight at once	1	2	3	4	5	6	7
I have to start all over again several times until I succeed	1	2	3	4	5	6	7
my partner/family isn't physically active	1	2	3	4	5	6	7

626

627

**6**. In spite of good intentions, slip ups may occur. How confident are you about restarting exercises?							
**I am sure I can keep being physically active regularly, even if...**	**Strongly Disagree**						**Strongly Agree**
I postpone my plans several times	1	2	3	4	5	6	7
I lose my motivation	1	2	3	4	5	6	7
I have not been active for several weeks	1	2	3	4	5	6	7

628

629

630

631

632

633

634

635

636

637

For peer review only - http://bmjopen.bmj.com/site/about/guidelines.xhtml

Section 4: Social Support

1. Below is a list of statements about social support. *(Please tick (✓)* ***one*** *box in each row to indicate how much you agree or disagree with the following statements).*

Within the past 3 months, I have got support from my friends and/or family to help me:

	Strongly Agree	Agree	Neither agree or disagree	Disagree	Strongly disagree
eat well	☐	☐	☐	☐	☐
be physically active	☐	☐	☐	☐	☐
lose weight	☐	☐	☐	☐	☐

2. How often have you felt lonely during the past week?

(Please tick (✓) ***one*** *box).*

None, or almost none of the time	☐
Some of the time	☐
Most of the time	☐
All, or almost all of the time	☐
Don't know	☐
Do not wish to answer	☐

3. How many people can you count on in times of need?

(Please tick (✓) ***one*** *box).*

0	☐	→ Please go to Section 5 on Page 16
1	☐	→ Please complete the next question in this section (i.e. question 4)
2	☐	
3	☐	
4	☐	
5	☐	
6	☐	
7	☐	
8	☐	
9	☐	
10 or more	☐	

For peer review only - http://bmjopen.bmj.com/site/about/guidelines.xhtml

4. How many of these people live in your local area? That is, within about a mile or a 20 minute walk from your home.

*(Please tick (✓) **one** box).*

0	☐
1	☐
2	☐
3	☐
4	☐
5	☐
6	☐
7	☐
8	☐
9	☐
10 or more	☐

For peer review only - http://bmjopen.bmj.com/site/about/guidelines.xhtml

Section 5: Lifestyle behaviours and attitudes

1. How often do you weigh yourself currently? *(Please tick (✓) **one** box).*

Never	☐
About once a year or less	☐
Every couple of months	☐
Every month	☐
Every week	☐
Every day	☐
More than once a day	☐

2. Within the past 3 months, have you taken part in any weight loss programmes (e.g. Slimming World). *(Please tick (✓) **one** box).*

Yes	☐
No	☐

If yes, please give details below;

Name of programme	*How long you attended for*

3. Do you want to lose weight? *(Please tick (✓) **one** box).*

Yes	☐	→ Please complete the rest of the questions in this section (i.e. questions 4, 5 & 6)
No	☐	→ Please go to Section 6 on Page 19

4. How confident are you in your ability to:

*(Please circle **one** number in each row to indicate how confident you feel about each of the following statements).*

	**Not confident**						**Very confident**
Lose weight	1	2	3	4	5	6	7
Keep lost weight off in the long term	1	2	3	4	5	6	7
Stay at current weight	1	2	3	4	5	6	7

For peer review only

For peer review only - http://bmjopen.bmj.com/site/about/guidelines.xhtml

5. How important is losing weight for you at the moment? *(Please circle **one** number).*

Not important						Very important
1	2	3	4	5	6	7

6. The questions below ask you about why you want to lose weight. *(Please tick (✓) **one** box for each statement).*

I want to lose weight...

	Absolutely not	Somewhat	Moderately	Strongly
Because it is commonly said that living with overweight is unhealthy	☐	☐	☐	☐
To be healthier	☐	☐	☐	☐
To be more agile	☐	☐	☐	☐
For health reasons	☐	☐	☐	☐
Because I read that it is healthier	☐	☐	☐	☐
To decrease my health risks	☐	☐	☐	☐
To live long	☐	☐	☐	☐
Because acquaintances have advised me to	☐	☐	☐	☐
To not attract attention	☐	☐	☐	☐
Because I'll be more successful in my job	☐	☐	☐	☐
So I will be accepted by society	☐	☐	☐	☐
To dare to socialise again	☐	☐	☐	☐
Because I would be luckier in love	☐	☐	☐	☐
To be more appreciated/liked	☐	☐	☐	☐
To have more friends	☐	☐	☐	☐
To have better success with others	☐	☐	☐	☐
So that other people will think better of me	☐	☐	☐	☐
To be more attractive	☐	☐	☐	☐
To like to look at myself in the mirror again	☐	☐	☐	☐
Because I want to like myself more	☐	☐	☐	☐

For peer review only - http://bmjopen.bmj.com/site/about/guidelines.xhtml

**I want to lose weight…**	**Absolutely not**	**Somewhat**	**Moderately**	**Strongly**
Because I want to be more attractive	☐	☐	☐	☐
To be able to dress more fashionably	☐	☐	☐	☐
To fit into my clothes again	☐	☐	☐	☐
To feel more self-confident	☐	☐	☐	☐

For peer review only

For peer review only - http://bmjopen.bmj.com/site/about/guidelines.xhtml

Section 6: Food patterns

*(Please tick (✓) **one** box for each of the following questions).*

1. How many times a week do you eat two or more pieces of fruit?	
6 or more days a week	☐
3-5 days a week	☐
1-2 days a week	☐
Less than one day a week	☐
Never	☐

2. When eating cheese, how often do you choose reduced fat cheese in preference to regular cheese?	
Always	☐
Usually	☐
Occasionally	☐
Rarely	☐
Never	☐
I don't eat cheese	☐

3. How many days a week do you eat fried food with a batter or bread crumb coating?	
4 or more days a week	☐
2 or 3 days a week	☐
Once a week	☐
Less than once a week	☐
Never	☐

4. How often do you eat fried or roasted vegetables?	
Always	☐
Usually	☐
Occasionally	☐
Rarely	☐
Never	☐

5. When eating bread (as toast, sandwiches or a snack) how often do you spread butter or margarine on it?	
Always	☐
Usually	☐
Occasionally	☐
Rarely	☐
Never	☐

For peer review only

For peer review only - http://bmjopen.bmj.com/site/about/guidelines.xhtml

**6. How many portions of vegetables do you eat in a typical day? (a portion is about three tablespoons of vegetables or a small bowl of salad).**	
5 or more portions	☐
3 or 4 portions	☐
1 or 2 portions	☐
Less than one portion per day	☐
None	☐

662

**7. How often do you trim all the visible fat off the meat you eat?**	
Always	☐
Usually	☐
Occasionally	☐
Rarely	☐
Never	☐
I don't eat meat	☐

663

**8. How many times a week do you eat meat pies, pasties or sausage rolls?**	
3 or more times a week	☐
Once or twice a week	☐
Once a fortnight	☐
Less than once a fortnight	☐
Never	☐

664

**9. How often do you (or the person who cooks for you) remove the skin from chicken before it is cooked?**	
Always	☐
Usually	☐
Occasionally	☐
Rarely	☐
Never	☐
I don't eat chicken	☐

665

**10. How many days a week do you eat fried potato (e.g. hot chips or potato crisps)?**	
6 or more days a week	☐
3-5 days a week	☐
1-2 days a week	☐
Less than one day a week	☐
Never	☐

For peer review only

**11. How many days a week do you eat take-away foods such as: fried or BBQ chicken; fish and chips; Chinese; pizza; hamburger etc.?**	
6 or more days a week	☐
3-5 days a week	☐
1-2 days a week	☐
Less than one day a week	☐
Never	☐

**12. How often do you (or the person who cooks for you) use fat when cooking? (e.g. butter, margarine, oil, lard etc)**	
Always	☐
Usually	☐
Occasionally	☐
Rarely	☐
Never	☐

**13. How often do you choose wholemeal spaghetti or pasta in preference to regular spaghetti or pasta?**	
Always	☐
Usually	☐
Occasionally	☐
Rarely	☐
Never	☐
I don't eat spaghetti/pasta	☐

**14. How often do you choose wholemeal bread in preference to white bread?**	
Always	☐
Usually	☐
Occasionally	☐
Rarely	☐
Never	☐

**15. How many days a week do you eat legumes? (e.g. baked beans, three bean mix, lentils, split peas, dried beans etc)**	
4 or more days a week	☐
2 or 3 days a week	☐
Once a week	☐
Less than once a week	☐
Never	☐

For peer review only - http://bmjopen.bmj.com/site/about/guidelines.xhtml

**16. How many days a week do you eat a high fibre breakfast cereal? (e.g. Weetabix, All-Bran, untoasted muesli, porridge)**	
6 or more days a week	☐
3-5 days a week	☐
1-2 days a week	☐
Less than one day a week	☐
Never	☐

671

**17. How many different types of vegetables would you eat on a typical day?**	
5 or more types	☐
4 types	☐
3 types	☐
1 or 2 types	☐
None	☐

672

**18. How many days a week do you eat high fat cheeses? (e.g. cheddar or cream cheese)**	
6 or more days a week	☐
3-5 days a week	☐
1-2 days a week	☐
Less than one day a week	☐
Never	☐

673

**19. How often do you choose low-fat milk (semi-skimmed or skimmed) in preference to whole milk?**	
Always	☐
Usually	☐
Occasionally	☐
Rarely	☐
Never	☐
I don't drink milk	☐

674

**20. How many days a week do you eat processed meats? (e.g. bacon, salami, ham etc)**	
4 or more days a week	☐
2 or 3 days a week	☐
Once a week	☐
Less than one day a week	☐
Never	☐

For peer review only - http://bmjopen.bmj.com/site/about/guidelines.xhtml

**21. How often do you eat or drink any of the following?** *(Please tick (✓) **one** box for each row).*	**More than once a day**	**Once a day**	**3-6 days a week**	**1-2 days a week**	**less than weekly**	**Never**
Sweets, chocolate bars or biscuits (including wrapped chocolate biscuits like Twix or KitKat)	☐	☐	☐	☐	☐	☐
Buns, cakes or pastries or desserts	☐	☐	☐	☐	☐	☐
Fizzy drinks or squashes that contain sugar (e.g. coca cola, Ribena, Club Orange)	☐	☐	☐	☐	☐	☐
Diet drinks (e.g. Diet Coke, Sprite Zero, Diet Club)	☐	☐	☐	☐	☐	☐

675

**22. How often do you have a drink containing alcohol?**	
Never	☐
Monthly or less	☐
2 - 4 times a month	☐
2 – 3 times a week	☐
4 or more times a week	☐

676

**23. Do you keep a record of what you eat and drink? (e.g. writing it down, using an App etc).**	
Yes	☐
No	☐

677

**24. Do you set yourself food and drink related goals?** *(Please tick (✓) **one** box).*	
Yes	☐
No	☐

678

679

680

681

682

683

For peer review only - http://bmjopen.bmj.com/site/about/guidelines.xhtml

Section 7: Physical activity

We are interested in finding out about the kinds of physical activities that people do as part of their everyday lives. The questions will ask you about the time you spent being physically active **in the last 7 days**. Please answer each question even if you do not consider yourself to be an active person. Please think about the activities you do at work, as part of your house and yard work, to get from place to place, and in your spare time for recreation, exercise or sport.

VIGOROUS ACTIVITIES

Think about all the **vigorous** activities that you did **in the last 7 days**. **Vigorous** physical activities refer to activities that take hard physical effort and make you breathe much harder than normal. Think *only* about those physical activities that you did for at least 10 minutes at a time.

**1. During the last 7 days, on how many days did you do vigorous physical activities like heavy lifting, digging, aerobics, or fast bicycling?**		
________	Days per week	
☐	No vigorous physical activities ⟶	***Skip to question 3***

**2. How much time did you usually spend doing vigorous physical activities on one of those days?** *(Please answer in hours **AND** minutes. E.g. If you did 30 minutes of vigorous exercise, you can enter '0' in the Hours box and '30' in the Minutes box)*	
________	Hours per day
________	Minutes per day
☐	Don't know/not sure

For peer review only - http://bmjopen.bmj.com/site/about/guidelines.xhtml

MODERATE ACTIVITIES

Think about all the **moderate** activities that you did **in the last 7 days**. **Moderate** activities refer to activities that take moderate physical effort and make you breathe somewhat harder than normal. Think only about those physical activities that you did for at least 10 minutes at a time.

3. During the last 7 days, on how many days did you do moderate physical activities like carrying light loads, bicycling at a regular pace, or doubles tennis? Do not include walking.

_______ Days per week

☐ No moderate physical activities ⟶ ***Skip to question 5***

4. How much time did you usually spend doing moderate physical activities on one of those days?

*(Please answer in hours **AND** minutes. E.g. If you did 30 minutes of moderate exercise, you can enter '0' in the Hours box and '30' in the Minutes box)*

_______ Hours per day

_______ Minutes per day

☐ Don't know/not sure

WALKING

Think about the time you spent **walking in the last 7 days**. This includes at work and at home, walking to travel from place to place, and any other walking that you have done solely for recreation, sport, exercise, or leisure.

5. During the last 7 days, on how many days did you walk for at least 10 minutes at a time?

_______ Days per week

☐ No walking ⟶ ***Skip to question 7***

6. How much time did you usually spend walking on one of those days?

*(Please answer in hours **AND** minutes. E.g. If you walked for 2 hours, you can enter '2' in the Hours box and '0' in the Minutes box)*

_______ Hours per day

_______ Minutes per day

☐ Don't know/not sure

For peer review only

SITTING

The last question is about the time you spent **sitting** on weekdays during the **last 7 days**. Include time spent at work, at home, while doing course work and during leisure time. This may include time spent sitting at a desk, visiting friends, reading, or sitting or lying down to watch television.

**7. During the last 7 days, how much time did you spend sitting on a week day?** *(Please answer in hours* ***AND*** *minutes. E.g. If you spent 8 hours sitting, you can enter '8' in the Hours box and '0' in the Minutes box)*	
________	Hours per day
________	Minutes per day
☐	Don't know/not sure

STEPS

**8. Do you use a step counter? (e.g. pedometer, App, Fitbit etc)** *(Please tick (✓)* ***one*** *box).*	
Yes	☐
No	☐

**9. Do you set yourself physical activity related goals?** *(Please tick (✓)* ***one*** *box).*	
Yes	☐
No	☐

**10. Did you have any problem/s that limited your physical activity during the past 3 months?** *(Please tick (✓)* ***one*** *box).*		
Yes	☐	If yes, please give details:
No	☐	

For peer review only

For peer review only - http://bmjopen.bmj.com/site/about/guidelines.xhtml

Section 8: Infant Feeding

The questions in this section are designed to explore how you feed your youngest child. If your most recent pregnancy was a twin/multiple pregnancy, please complete these questions for the youngest twin/multiple.

1. What is your current method of feeding for your youngest child? *(Please tick (✓) **one** box for each row).*

Breast feeding	☐ yes	☐ no
Infant formula feeding	☐ yes	☐ no
Combined feeding (Breast and infant formula)	☐ yes	☐ no
Follow-on milk	☐ yes	☐ no
Baby is on solids	☐ yes	☐ no

NOTE: If you ticked 'Baby is on solids', **please go to question 2 below**.
If you did not tick 'Baby is on solids', **please go to Section 9 on Page 29**.

2. How often do you usually give your baby these particular TYPES of solid food? *(Please tick (✓) **one** box for each food).*

	More than once a day	Once a day	3 or more times a week	Once or twice a week	Less than once a week	Never
Breakfast Cereals	☐	☐	☐	☐	☐	☐
Rice or Pasta	☐	☐	☐	☐	☐	☐
Bread	☐	☐	☐	☐	☐	☐
Potatoes	☐	☐	☐	☐	☐	☐
Potato products (incl. chips, waffles, shapes)	☐	☐	☐	☐	☐	☐
Butter/Margarine and other spreads	☐	☐	☐	☐	☐	☐
Red meat	☐	☐	☐	☐	☐	☐
Processed meat (e.g. ham)	☐	☐	☐	☐	☐	☐

For peer review only - http://bmjopen.bmj.com/site/about/guidelines.xhtml

	More than once a day	Once a day	3 or more times a week	Once or twice a week	Less than once a week	Never
Chicken/other poultry	☐	☐	☐	☐	☐	☐
Fish (incl. tuna)	☐	☐	☐	☐	☐	☐
Eggs	☐	☐	☐	☐	☐	☐
Beans, lentils, chickpeas	☐	☐	☐	☐	☐	☐
Tofu, Quorn, textured vegetable protein	☐	☐	☐	☐	☐	☐
Nuts	☐	☐	☐	☐	☐	☐
Fruit	☐	☐	☐	☐	☐	☐
Vegetables	☐	☐	☐	☐	☐	☐
Cheese, yoghurt, fromage frais	☐	☐	☐	☐	☐	☐
Puddings or desserts	☐	☐	☐	☐	☐	☐
Biscuits, sweets, chocolate or cakes	☐	☐	☐	☐	☐	☐
Crisps and corn snacks	☐	☐	☐	☐	☐	☐
Follow on formula	☐	☐	☐	☐	☐	☐
Or something else (please tick and then write below)	☐	☐	☐	☐	☐	☐

3. Do you ever add salt to your baby's solid food, including adding salt when the food is being cooked? *(Please tick (✓)* ***one*** *box).*

Yes, often	☐
Yes, sometimes	☐
Never	☐

For peer review only - http://bmjopen.bmj.com/site/about/guidelines.xhtml

Section 9: Mood

*(Please tick (✓) **one** box for each statement below).*

In the past 7 days...

**1. I have been able to laugh and see the funny side of things.**	
As much as I always could	☐
Not quite so much now	☐
Definitely not so much now	☐
Not at all	☐

**2. I have looked forward with enjoyment to things.**	
As much as I ever did	☐
Rather less than I used to	☐
Definitely less than I used to	☐
Hardly at all	☐

**3. I have blamed myself unnecessarily when things went wrong.**	
Yes, most of the time	☐
Yes, some of the time	☐
Not very often	☐
No, never	☐

**4. I have been anxious or worried for no good reason.**	
No, not at all	☐
Hardly ever	☐
Yes, sometimes	☐
Yes, very often	☐

**5. I have felt scared or panicky for no very good reason.**	
Yes, quite a lot	☐
Yes, sometimes	☐
No, not much	☐
No, not at all	☐

For peer review only

**6. Things have been getting on top of me.**	
Yes, most of the time I haven’t been able to cope at all	☐
Yes, sometimes I haven’t been coping as well as usual	☐
No, most of the time I have coped quite well	☐
No, I have been coping as well as ever	☐

**7. I have been so unhappy that I have had difficulty sleeping.**	
Yes, most of the time	☐
Yes, sometimes	☐
Not very often	☐
No, not at all	☐

**8. I have felt sad or miserable.**	
Yes, most of the time	☐
Yes, quite often	☐
Not very often	☐
No, not at all	☐

**9. I have been so unhappy that I have been crying.**	
Yes, most of the time	☐
Yes, quite often	☐
Only occasionally	☐
No, never	☐

**10. The thought of harming myself has occurred to me.**	
Yes, quite often	☐
Sometimes	☐
Hardly ever	☐
Never	☐

For peer review only

(Please tick (✓) ***one*** *box for each statement below).*

<u>Over the last two weeks</u>, how often have you been bothered by the following problems?

**11. Feeling nervous, anxious, or on edge?**	
Not at all	☐
Several days	☐
More than half of the days	☐
Nearly every day	☐

**12. Not being able to stop or control worrying?**	
Not at all	☐
Several days	☐
More than half of the days	☐
Nearly every day	☐

**13. Worrying too much about different things?**	
Not at all	☐
Several days	☐
More than half of the days	☐
Nearly every day	☐

**14. Trouble relaxing?**	
Not at all	☐
Several days	☐
More than half of the days	☐
Nearly every day	☐

**15. Being so restless that it is hard to sit still?**	
Not at all	☐
Several days	☐
More than half of the days	☐
Nearly every day	☐

For peer review only

**16. Becoming easily annoyed or irritable?**	
Not at all	☐
Several days	☐
More than half of the days	☐
Nearly every day	☐

**17. Feeling afraid as if something awful might happen?**	
Not at all	☐
Several days	☐
More than half of the days	☐
Nearly every day	☐

Having a new baby can bring up lots of different feelings and emotions. The next questions are designed to explore how you are feeling about being a parent to your baby. Answering these questions will help us to understand how things are going for you. If your most recent pregnancy was a twin/multiple pregnancy, please complete these questions for the youngest twin/multiple.

18. Thinking about your feelings about your baby, choose the response for each statement that feels right to you. *(Please tick (✓)* ***one*** *box for each of the following statements).*

	**Never**	**Sometimes**	**Always**
I enjoy looking after my baby	☐	☐	☐
I feel irritated with my baby when we are together	☐	☐	☐
I feel affectionate towards my baby	☐	☐	☐
I feel that my baby is being difficult or trying to upset me on purpose	☐	☐	☐
I can work out what my baby needs from me	☐	☐	☐
I feel like I can't do things I enjoy because of my baby.	☐	☐	☐
I feel the changes in my life are worth it to look after my baby.	☐	☐	☐
I miss my baby when we are not together	☐	☐	☐
I feel like I'm looking after my baby for someone else.	☐	☐	☐

For peer review

For peer review only - http://bmjopen.bmj.com/site/about/guidelines.xhtml

	Never	Sometimes	Always
When we've been apart I look forward to seeing my baby again.	☐	☐	☐
I enjoy playing with my baby.	☐	☐	☐

782

19. Thinking about your baby, please tick one of the choices for each of the questions below. There are no 'right' or 'wrong' answers; many of these are true of all babies at times. *(Please tick (✓)* ***one*** *box for each of the following statements).*

	Always	Very often	Quite often	Sometimes	Rarely	Never
My baby smiles at me.	☐	☐	☐	☐	☐	☐
My baby annoys me.	☐	☐	☐	☐	☐	☐
My baby likes doing things with me.	☐	☐	☐	☐	☐	☐
My baby 'talks' to me.	☐	☐	☐	☐	☐	☐
My baby irritates me.	☐	☐	☐	☐	☐	☐
My baby likes me.	☐	☐	☐	☐	☐	☐
My baby wants too much attention.	☐	☐	☐	☐	☐	☐
My baby laughs.	☐	☐	☐	☐	☐	☐
My baby gets moody.	☐	☐	☐	☐	☐	☐
My baby dominates me.	☐	☐	☐	☐	☐	☐
My baby likes to please me.	☐	☐	☐	☐	☐	☐
My baby cries for no obvious reason.	☐	☐	☐	☐	☐	☐
My baby is affectionate towards me.	☐	☐	☐	☐	☐	☐
My baby winds me up.	☐	☐	☐	☐	☐	☐

783

For peer review only

For peer review only - http://bmjopen.bmj.com/site/about/guidelines.xhtml

Section 10: Self-esteem

Below is a list of statements dealing with your general feelings about yourself.

*(Please tick (✓) **one** box to indicate how much you agree or disagree with each of the following statements).*

	Strongly Agree	Agree	Disagree	Strongly disagree
I feel that I'm a person of worth, at least on an equal plane with others.	☐	☐	☐	☐
I feel that I have a number of good qualities.	☐	☐	☐	☐
All in all, I am inclined to feel that I am a failure.	☐	☐	☐	☐
I am able to do things as well as most other people.	☐	☐	☐	☐
I feel I do not have much to be proud of.	☐	☐	☐	☐
I take a positive attitude toward myself.	☐	☐	☐	☐
On the whole I am satisfied with myself.	☐	☐	☐	☐
I wish I could have more respect for myself.	☐	☐	☐	☐
I certainly feel useless at times.	☐	☐	☐	☐
At times I think I am no good at all.	☐	☐	☐	☐

Section 11: Sleep

For peer review only - http://bmjopen.bmj.com/site/about/guidelines.xhtml

1. At the moment, how often does your baby sleep all night? *(Please tick (✓)* ***one*** *box).*

Always	☐
Mostly	☐
Sometimes	☐
Rarely	☐
Never	☐

The following questions relate to **your** usual sleep habits during the **past month only**. Your answers should indicate the most accurate reply for the majority of days and nights in the past month. Please answer all questions.

2. During the past month, what time have you usually gone to bed at night?

Usual bed time ☐

3. During the past month, how long (in minutes) has it usually taken you to fall asleep each night?

Number of minutes ☐

4. During the past month, what has been your usual getting up time in the morning?

Usual getting up time ☐

5. During the past month, on average how many hours of actual sleep did you get at night? (This may be different than the number of hours you spent in bed.)

Hours of sleep per night ☐

6. During the past month, how often have you had trouble sleeping because you…

For peer review only - http://bmjopen.bmj.com/site/about/guidelines.xhtml

*(Please tick (✓) **one** box for each statement).*	**Not during the past month**	**Less than once a week**	**Once or twice a week**	**Three or more times a week**
Cannot get to sleep within 30 minutes	☐	☐	☐	☐
Wake up in the middle of the night or early morning	☐	☐	☐	☐
Have to get up to use the bathroom	☐	☐	☐	☐
Cannot breathe comfortably	☐	☐	☐	☐
Cough or snore loudly	☐	☐	☐	☐
Feel too cold	☐	☐	☐	☐
Feel too hot	☐	☐	☐	☐
Had bad dreams	☐	☐	☐	☐
Have pain	☐	☐	☐	☐
Have to take care of your baby	☐	☐	☐	☐
Other reason(s), please describe and indicate how often during the past month you had trouble sleeping because of this:				
☐	☐	☐	☐	☐
☐	☐	☐	☐	☐
☐	☐	☐	☐	☐

818
819
820
821
822
823
824

7. During the past month, how would you rate your sleep quality overall?
*(Please tick (✓) **one** box).*

Very Good	Fairly Good	Fairly Bad	Very Bad
☐	☐	☐	☐

**8**. (Please tick (✓) ***one*** *box for each question below.)*	**Not during the past month**	**Less than once a week**	**Once or twice a week**	**Three or more times a week**
**During the past month, how often have you taken medicine (prescribed or "over the counter") to help you sleep?**	☐	☐	☐	☐
**During the past month, how often have you had trouble staying awake while driving, eating meals, or engaging in social activity?**	☐	☐	☐	☐

9. During the past month, how much of a problem has it been for you to keep up enough enthusiasm to get things done? (Please tick *(✓)* ***one*** *box).*

No problem at all	Only a very slight problem	Somewhat of a problem	A very big problem
☐	☐	☐	☐

10. Do you usually have a bed partner or room-mate? *(Please tick (✓)* ***one*** *box).*

No bed partner or room-mate	Partner/room-mate in other room	Partner in same room but not in same bed	Partner in same bed
☐	☐	☐	☐

**11. If you usually have a room-mate or bed partner, ask him/her how often in the past month you have had:** Please tick *(✓)* ***one*** *box for each statement).*	**Not during the**	**Less than**	**Once or**	**Three or more**

For peer review only - http://bmjopen.bmj.com/site/about/guidelines.xhtml

	past month	once a week	twice a week	times a week
Loud snoring	☐	☐	☐	☐
Long pauses between breaths while asleep	☐	☐	☐	☐
Legs twitching or jerking while you sleep	☐	☐	☐	☐
Episodes of confusion during sleep	☐	☐	☐	☐
Other restlessness while you sleep, please describe and indicate how often in the past month you have had this:				
☐	☐	☐	☐	☐
☐	☐	☐	☐	☐
☐	☐	☐	☐	☐

For peer review only - http://bmjopen.bmj.com/site/about/guidelines.xhtml

You have now completed the questionnaire.
Thank you very much for your time and effort!
We really appreciate your help with our research.

For peer review only

For peer review only - http://bmjopen.bmj.com/site/about/guidelines.xhtml

Supporting MumS (SMS) study Questionnaire Booklet

Month 12 Intervention

**Participant ID:**	_ _ _ _ _
**Researcher name:**	
**Today's date:**	_ _ / _ _ / _ _ _ _<br>**e.g. 05 / 01 / 2017**

About this questionnaire booklet

Thank you for taking the time to fill out this questionnaire booklet.

The questionnaire booklet is made up of questions that will help us find out more about your experience of the postpartum period. Please complete the questions as accurately as you can. **There are no right or wrong answers**. If there are any questions you do not wish to answer, you can leave them blank. Note that your responses will be **anonymous,** this means your name will never be given with your responses.

The questionnaire booklet is split up into the short sections shown below. You can fill it all out at once, or do it in chunks, as your time allows.

**Section 1**	**SMS evaluation**	**Pages 3-8**
**Section 2**	**Health and well-being**	**Pages 9-11**
**Section 3**	**Health resources**	**Pages 12-14**
**Section 4**	**Eating and activity approaches**	**Pages 15-19**
**Section 5**	**Social support**	**Page 20-21**
**Section 6**	**Lifestyle behaviours and attitudes**	**Page 22-24**
**Section 7**	**Food patterns**	**Page 25-29**
**Section 8**	**Physical activity**	**Page 30-32**
**Section 9**	**Infant feeding**	**Page 33-34**
**Section 10**	**Mood**	**Page 35-42**
**Section 11**	**Self-esteem**	**Page 43**
**Section 12**	**Sleep**	**Page 44-47**

For peer review only - http://bmjopen.bmj.com/site/about/guidelines.xhtml

Section 1: SMS Evaluation

Thank you for participating in our study. We would like to ask you some questions about how you have found participating in this research. Your responses to this survey will help us to improve the studies we carry out in the future.

1. How satisfied were you with your overall experience of the SMS study? *(Please tick (✓) **one** box).*

Very Dissatisfied	Mostly Dissatisfied	Neither Satisfied Nor Dissatisfied	Mostly Satisfied	Very Satisfied
☐	☐	☐	☐	☐

If you were very or mostly dissatisfied, please explain what could have been improved:

____

____

____

2. Please answer the questions below about your experience of taking part in the SMS study. *(Please tick (✓) **one** box to indicate how much you agree or disagree with each of the following statements).*

	Strongly disagree	Disagree	Neither agree or disagree	Agree	Strongly Agree
At the start of the study, the information I was given about the study was clear and informative	☐	☐	☐	☐	☐
The amount of information and level of support provided by the researchers during the study was suited to my needs	☐	☐	☐	☐	☐

If you have disagreed or strongly disagreed with any of the above statements, please explain what could have been improved:

____

____

____

For peer review only - http://bmjopen.bmj.com/site/about/guidelines.xhtml

For peer review only

909

3. How satisfied were you with the text messages? *(Please tick (✓) **one** box).*

Very Dissatisfied	Mostly Dissatisfied	Neither Satisfied Nor Dissatisfied	Mostly Satisfied	Very Satisfied
☐	☐	☐	☐	☐

910

If you were very or mostly dissatisfied, please explain what could have been improved:

____

____

____

911

4. **Please answer the questions below about your experience of the text messages you received during the SMS study.** *(Please tick (✓) **one** box to indicate how much you agree or disagree with each of the following statements).*

	Strongly Disagree	Disagree	Neither agree or disagree	Agree	Strongly agree
SMS texts were easy to understand	☐	☐	☐	☐	☐
SMS texts were helpful	☐	☐	☐	☐	☐
SMS texts were interesting	☐	☐	☐	☐	☐
An appropriate amount of SMS texts were sent during the study	☐	☐	☐	☐	☐
SMS texts were delivered at appropriate times of the day	☐	☐	☐	☐	☐

912

If you have disagreed or strongly disagreed with any of the above statements, please explain what could have been improved:

____

____

____

913

For peer review only - http://bmjopen.bmj.com/site/about/guidelines.xhtml

5. How helpful did you find the following parts of SMS in helping you to lose weight and maintain weight loss? *(Please tick (✓) **one** response for each statement below)*

	Very unhelpful	Somewhat unhelpful	Neither helpful or unhelpful	Somewhat helpful	Very helpful	Didn't use it
Reminders to set goals	☐	☐	☐	☐	☐	☐
Reminders to weigh yourself weekly	☐	☐	☐	☐	☐	☐
Trigger words (e.g. 'crave', 'tired' etc.)	☐	☐	☐	☐	☐	☐
Support system (i.e. having a friend receive SMS messages as well)	☐	☐	☐	☐	☐	☐
The Yes/No replies	☐	☐	☐	☐	☐	☐
Web links provided within the text messages	☐	☐	☐	☐	☐	☐
The feedback messages about your weight (i.e. replies to UP, DOWN, SAME texts)	☐	☐	☐	☐	☐	☐

If you have felt any aspect was not helpful, please explain what could have been improved:

___

___

___

For peer review only - http://bmjopen.bmj.com/site/about/guidelines.xhtml

6. During the study you were asked to attend 3 study visits. Please rate how easy or difficult you found each aspect of these visits. *(Please tick (✓) **one** response that most applies to you in relation to your experience).*

	Very difficult	Difficult	No strong opinion	Easy	Very easy
Location of visits	☐	☐	☐	☐	☐
Length of visits	☐	☐	☐	☐	☐
Having your height measurement taken	☐	☐	☐	☐	☐
Having your waist measurements taken	☐	☐	☐	☐	☐
Having your weight taken	☐	☐	☐	☐	☐
Completing the questionnaires	☐	☐	☐	☐	☐

If you have felt that any aspects of the visits were difficult, please explain how they could have been improved:

____

____

____

For peer review only

For peer review only - http://bmjopen.bmj.com/site/about/guidelines.xhtml

7. Did you sign-up to receive additional text messages on giving up smoking during SMS? *(Please tick (✓) **one** box).*

Yes	☐	→ Please complete Question 8.
No	☐	→ Please go to Question 9.

8. Did you give up smoking during SMS? *(Please tick (✓) **one** box).*

Yes	☐
No	☐

9. Did you sign-up to receive additional text messages on breastfeeding during SMS? *(Please tick (✓) **one** box).*

Yes	☐	→ Please complete Question 10.
No	☐	→ Please go to Question 11.

10. How helpful did you find the breastfeeding messages? *(Please tick (✓) **one** box).*

Very Unhelpful	Somewhat Unhelpful	Neither helpful or unhelpful	Somewhat helpful	Very helpful
☐	☐	☐	☐	☐

If you felt these messages were not helpful, please explain what could have been improved:

____

____

____

For peer review only

For peer review only - http://bmjopen.bmj.com/site/about/guidelines.xhtml

933

**11. Based on your experience in taking part in SMS, do you think the programme should be offered to other mums?** *(Please tick (✓)* ***one*** *response).*	
Yes	☐
No	☐

934

If you answered no, please explain why:

____

____

____

935

12. Do you have any other comments? We would welcome any suggestions you have regarding how we could improve future studies for those taking part:

____

____

____

936
937
938
939
940
941

For peer review only

For peer review only - http://bmjopen.bmj.com/site/about/guidelines.xhtml

Section 2: Health and well-being

For questions 1-5, under each heading, please tick (✓) the **ONE** box that best describes your health TODAY.

**1. Mobility**	
I have no problems in walking about	☐
I have slight problems in walking about	☐
I have moderate problems in walking about	☐
I have severe problems in walking about	☐
I am unable to walk about	☐

**2. Self-care**	
I have no problems washing or dressing myself	☐
I have slight problems washing or dressing myself	☐
I have moderate problems washing or dressing myself	☐
I have severe problems washing or dressing myself	☐
I am unable to wash or dress myself	☐

**3. Usual Activities** *(e.g. work, study, housework, family or leisure activities)*	
I have no problems doing my usual activities	☐
I have slight problems doing my usual activities	☐
I have moderate problems doing my usual activities	☐
I have severe problems doing my usual activities	☐
I am unable to do my usual activities	☐

**4. Pain/ Discomfort**	
I have no pain or discomfort	☐
I have slight pain or discomfort	☐
I have moderate pain or discomfort	☐
I have severe pain or discomfort	☐
I have extreme pain or discomfort	☐

For peer review only - http://bmjopen.bmj.com/site/about/guidelines.xhtml

5. Anxiety/ Depression	
I am not anxious or depressed	☐
I am slightly anxious or depressed	☐
I am moderately anxious or depressed	☐
I am severely anxious or depressed	☐
I am extremely anxious or depressed	☐

6. We would like to know how good or bad your health is TODAY.

This scale is numbered from 0 to 100.

100 means the best health you can imagine.

0 means the worst health you can imagine.

Please mark an X on the scale to indicate how your health is TODAY.

Now, write the number you marked on the scale in the box below.

Your health today = ☐

The best health you can imagine

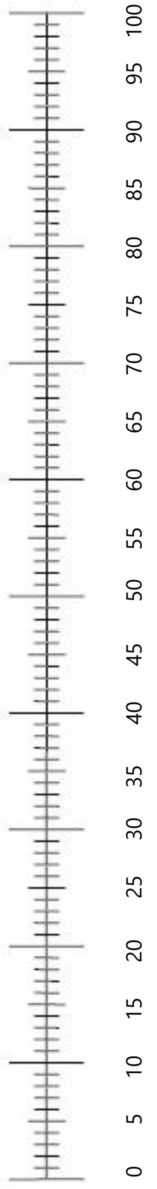

The worst health you can imagine

**7. Feeling settled and secure** *(Please tick (✓) **one** box that best describes your overall quality of life at the moment).*	
I am able to feel settled and secure in **all** areas of my life	☐
I am able to feel settled and secure in **many** areas of my life	☐
I am able to feel settled and secure in **a few** areas of my life	☐
I am unable to feel settled and secure in **any** areas of my life	☐

**8. Love, friendship and support** *Please tick (✓) **one** box that best describes your overall quality of life at the moment).*	
I can have **a lot** of love, friendship and support	☐
I can have **quite a lot** of love, friendship and support	☐
I can have **a little** love, friendship and support	☐
I **cannot** have **any** love, friendship and support	☐

**9. Being independent** *Please tick (✓) **one** box that best describes your overall quality of life at the moment).*	
I am able to be **completely** independent	☐
I am able to be independent in **many** things	☐
I am able to be independent in **a few** things	☐
I am **unable** to be at all independent	☐

**10. Achievement and progress** *Please tick (✓) **one** box that best describes your overall quality of life at the moment).*	
I can achieve and progress in **all** aspects of my life	☐
I can achieve and progress in **many** aspects of my life	☐
I can achieve and progress in **a few** aspects of my life	☐
I cannot achieve and progress in **any** aspects of my life	☐

**11. Enjoyment and pleasure** *Please tick (✓) **one** box that best describes your overall quality of life at the moment).*	
I can have **a lot** of enjoyment and pleasure	☐
I can have **quite a lot** of enjoyment and pleasure	☐
I can have **a little** enjoyment and pleasure	☐
I **cannot** have **any** enjoyment and pleasure	☐

For peer review only - http://bmjopen.bmj.com/site/about/guidelines.xhtml

Section 3: Health resources

1. 1. In the last 3 months, have you seen <u>any</u> health professional at your GP surgery? (this includes phone/online or face-to-face appointments) *(Please tick (✓) **one** box).*

Yes	☐
No	☐

If YES, <u>how many times</u> were you seen by: *(Please enter a number)*

GP	
Practice nurse	
Other (please state)	
Other (please state)	

2. In the last 3 months, have you attended an Accident and Emergency (Casualty) department? *(Please tick (✓) **one** box).*

Yes	☐	If YES, how many <u>times?</u>	
No	☐		

3. In the last 3 months, have you attended hospital as an <u>out-patient?</u> *(Please tick (✓) **one** box).*

Yes	☐	If YES, how many <u>times?</u>	
No	☐		

4. In the last 3 months, have you attended hospital as an <u>in-patient?</u> *(Please tick (✓) **one** box).*

Yes	☐	If YES, how many <u>times?</u>	
		If YES, how many nights did you spend in hospital in the last 3 months in total?	
No	☐		

5. In the last 3 months, have you received any prescriptions for medicine? *(Please tick (✓) **one** box)***.**

Yes	☐	If YES, how many prescriptions have you received?	
No	☐		

For peer review only - http://bmjopen.bmj.com/site/about/guidelines.xhtml

6. In the last 3 months, have you purchased any over-the-counter medications or lifestyle products (e.g. slimming aids, vitamin supplements)? *(Please tick (✓)* ***one*** *box).*

Yes	☐	If YES, please state total amount spent in last 3 months	£
No	☐		

7. In the last 3 months have you paid for any services or clubs for the specific purpose of helping you with your lifestyle - for example slimming clubs, health clubs, gyms, swimming pools, exercise classes?
(Please tick (✓) ***one*** *box).*

Yes	☐	If YES, approximately how much have you spent in total on all of these services/clubs in the last 3 months?	£
No	☐		

8. Please think about last week's food and drink purchases for your household (i.e. you and your family) and tell us the cost to your household of the following:
*(Please write '**0**' if you don't purchase a specific type of food/product) .*

Type of food and drink purchased	Weekly Cost to you (your household)
Food and non-alcoholic drinks (e.g. supermarket shopping)	£
Alcoholic drinks e.g. wine & beer	£
Takeaway meals and snacks eaten AT HOME e.g. pizza delivery	£
Meals, snacks and drinks CONSUMED AWAY FROM HOME (e.g. restaurant)	£
Cigarettes or vaping products	£

9. Have you previously purchased any apps for improving your fitness/health or wellbeing? *(Please tick (✓)* ***one*** *box).*

Yes	☐	If YES, Please state the total amount you spent on fitness/health apps in the last year?	£
No	☐		

For peer review only - http://bmjopen.bmj.com/site/about/guidelines.xhtml

10. On average, <u>per day</u> how much time have you spent exercising/walking in the last 3 months in total?

*(Please answer in hours **AND** minutes. E.g. If you did 30 minutes of exercise per day, you can enter '0' under Hours and '30' under the Minutes column)*

On average per day: Hours ☐ Minutes ☐

11. Has your employment status changed in the last three months?

(Note: returning to work after maternity leave should **not** be included as a change) *(Please tick (✓) **one** box).*

Yes ☐

No ☐

If YES, what is your **current** employment status? *(Please tick (✓) **one** box)*

☐ Full-time employment

☐ Part-time employment

☐ Unemployed

☐ Student or training

☐ Retired

☐ Not working due to illness

☐ Prefer not to answer

☐ Other (please specify) ________

For peer review only

For peer review only - http://bmjopen.bmj.com/site/about/guidelines.xhtml

Section 4: Eating and activity approaches

This section asks you about your diet. *(Please circle **one** number in each row to indicate how much you agree or disagree with the following statements).*

**1. Eating healthier is something…**	**Strongly Disagree**						**Strongly Agree**
I do automatically	1	2	3	4	5	6	7
I do without having to consciously remember	1	2	3	4	5	6	7
I do without thinking	1	2	3	4	5	6	7
I start doing before I realise I'm doing it	1	2	3	4	5	6	7

**2. Over the next few months I intend to…**	**Strongly Disagree**						**Strongly Agree**
eat a healthy diet (e.g. reduce portion size, eat less sugary and high fact snacks and eat more fruit and vegetables)	1	2	3	4	5	6	7
be physically active (e.g. by walking more or taking exercise classes)	1	2	3	4	5	6	7

**3. I already have concrete plans…**	**Strongly Disagree**						**Strongly Agree**
on how to eat a healthy diet	1	2	3	4	5	6	7
on when to eat a healthy diet	1	2	3	4	5	6	7

**4. I already have concrete plans…**	**Strongly Disagree**						**Strongly Agree**
on when I need to be especially careful to maintain my healthy diet	1	2	3	4	5	6	7
about what to do in difficult situations to stick to my healthy diet	1	2	3	4	5	6	7
on how I will cope if I slip back into old eating habits	1	2	3	4	5	6	7

For peer review only - http://bmjopen.bmj.com/site/about/guidelines.xhtml

**5. I am confident that I can stick to a healthy diet even…**	**Strongly Disagree**						**Strongly Agree**
if I have to learn a lot about nutrition	1	2	3	4	5	6	7
if I initially have to watch out in many situations	1	2	3	4	5	6	7
if I have to start all over again several times until I succeed	1	2	3	4	5	6	7
if I initially have to make plans	1	2	3	4	5	6	7
if initially food doesn't taste as good	1	2	3	4	5	6	7
if I initially don't get much support	1	2	3	4	5	6	7
if it takes a long time to get used to it	1	2	3	4	5	6	7
if I have worries and troubles	1	2	3	4	5	6	7
if my partner/ my family don't change their nutrition habits	1	2	3	4	5	6	7
if I am tired	1	2	3	4	5	6	7
if I am stressed out	1	2	3	4	5	6	7
if I don't lose weight initially	1	2	3	4	5	6	7

6. In spite of good intentions when losing weight, slip-ups may occur. Imagine you relapse back into your old eating habits. How confident are you about restarting your new healthy eating habits?

**I am sure I can start my new healthy eating habits again regularly, even if I…**	**Strongly Disagree**						**Strongly Agree**
have several bad days in a row	1	2	3	4	5	6	7
lose my motivation to keep up my new healthy eating habits	1	2	3	4	5	6	7
have had a relapse and have fallen back into old eating habits for several weeks	1	2	3	4	5	6	7

For peer review only - http://bmjopen.bmj.com/site/about/guidelines.xhtml

7. For the next few questions:

'Tempting foods' are any food you want to eat more of than you think you should. **'Eating intentions'** refer to the way you are aiming to eat, for example you may intend to avoid tempting foods or eat healthy foods.

*(Please tick (✓) **one** box for each of the following statements).*	**Never**	**Rarely**	**Sometimes**	**Often**	**Always**
I give up too easily on my eating intentions	☐	☐	☐	☐	☐
I'm good at resisting tempting food	☐	☐	☐	☐	☐
I easily get distracted from the way I intend to eat	☐	☐	☐	☐	☐
If I am not eating in the way I intend to I make changes	☐	☐	☐	☐	☐
I find it hard to remember what I have eaten throughout the day	☐	☐	☐	☐	☐

<u>This section asks about your physical activity</u>. *(Please circle **one** number in each row to indicate how much you agree or disagree with the following statements).*

**1. Being active every day is something…**	**Strongly Disagree**						**Strongly Agree**
I do automatically	1	2	3	4	5	6	7
I do without having to consciously remember	1	2	3	4	5	6	7
I do without thinking	1	2	3	4	5	6	7
I start doing before I realise I'm doing it	1	2	3	4	5	6	7

**2. I already have concrete plans…**	**Strongly Disagree**						**Strongly Agree**
on when to be physically active	1	2	3	4	5	6	7
on where to be physically active	1	2	3	4	5	6	7
on how to be physically active	1	2	3	4	5	6	7
on how often be physically active	1	2	3	4	5	6	7
on who I can be physically active with	1	2	3	4	5	6	7

For peer review only - http://bmjopen.bmj.com/site/about/guidelines.xhtml

**3. I already have concrete plans...**	**Strongly Disagree**						**Strongly Agree**
about what to do if something gets in the way	1	2	3	4	5	6	7
about what to do if I miss a physical activity session	1	2	3	4	5	6	7
about what to do in difficult situations to stick to my physical activity intentions	1	2	3	4	5	6	7
for times when I will need to be extra careful to stay committed	1	2	3	4	5	6	7

4a. Certain barriers make it hard to begin physical activity. How sure are you that you can begin exercising regularly?

**I am sure that...**	**Strongly Disagree**						**Strongly Agree**
I can change to a physically active lifestyle	1	2	3	4	5	6	7
I can be physically active once a week	1	2	3	4	5	6	7
I can be physically active at least 3 times a week for 30 minutes	1	2	3	4	5	6	7

**4b. I am sure I can start being physically active immediately, even if...**	**Strongly Disagree**						**Strongly Agree**
the planning for this takes a lot of time and effort	1	2	3	4	5	6	7
I have to force myself to start immediately	1	2	3	4	5	6	7
I have to push myself	1	2	3	4	5	6	7

For peer review only - http://bmjopen.bmj.com/site/about/guidelines.xhtml

5. Are you confident that you can manage staying physically active?

**I am sure I can keep being physically active regularly, even if...**	**Strongly Disagree**						**Strongly Agree**
it takes me a long time to make a habit	1	2	3	4	5	6	7
I am worried and troubled	1	2	3	4	5	6	7
I am tired	1	2	3	4	5	6	7
I am stressed out	1	2	3	4	5	6	7
I don't lose weight at once	1	2	3	4	5	6	7
I have to start all over again several times until I succeed	1	2	3	4	5	6	7
my partner/family isn't physically active	1	2	3	4	5	6	7

6. In spite of good intentions, slip ups may occur. How confident are you about restarting exercises?

**I am sure I can keep being physically active regularly, even if...**	**Strongly Disagree**						**Strongly Agree**
I postpone my plans several times	1	2	3	4	5	6	7
I lose my motivation	1	2	3	4	5	6	7
I have not been active for several weeks	1	2	3	4	5	6	7

For peer review only

Section 5: Social Support

1. Below is a list of statements about social support. *(Please tick (✓) **one** box in each row to indicate how much you agree or disagree with the following statements).*
Within the past 3 months, I have got support from my friends and/or family to help me:

	Strongly Agree	Agree	Neither agree or disagree	Disagree	Strongly disagree
...eat well	☐	☐	☐	☐	☐
...be physically active	☐	☐	☐	☐	☐
...lose weight	☐	☐	☐	☐	☐

2. How often have you felt lonely during the past week?
*(Please tick (✓) **one** box).*

None, or almost none of the time	☐
Some of the time	☐
Most of the time	☐
All, or almost all of the time	☐
Don't know	☐
suDo not wish to answer	☐

3. How many people can you count on in times of need?
*(Please tick (✓) **one** box).*

0	☐	→ Please go to Section 6 on Page 22
1	☐	
2	☐	
3	☐	
4	☐	
5	☐	→ Please complete the next question in this section (i.e. question 4)
6	☐	
7	☐	
8	☐	
9	☐	
10 or more	☐	

For peer review only - http://bmjopen.bmj.com/site/about/guidelines.xhtml

4. How many of these people live in your local area? That is, within about a mile or a 20 minute walk from your home.

*(Please tick (✓) **one** box).*

0	☐
1	☐
2	☐
3	☐
4	☐
5	☐
6	☐
7	☐
8	☐
9	☐
10 or more	☐

eview only

For peer review only - http://bmjopen.bmj.com/site/about/guidelines.xhtml

Section 6: Lifestyle behaviours and attitudes

4. How often do you weigh yourself currently? *(Please tick (✓) **one** box).*

Never	☐
About once a year or less	☐
Every couple of months	☐
Every month	☐
Every week	☐
Every day	☐
More than once a day	☐

5. Within the past 3 months, have you taken part in any weight loss programmes (e.g. Slimming World). *(Please tick (✓) **one** box).*

Yes	☐
No	☐

If yes, please give details below;

Name of programme	*How long you attended for*

6. Do you want to lose weight? *(Please tick (✓) **one** box).*

Yes	☐	→ Please complete the rest of the questions in this section (i.e. questions 4, 5 & 6)
No	☐	→ Please go to Section 7 on Page 25

4. How confident are you in your ability to:

*(Please circle **one** number in each row to indicate how confident you feel about each of the following statements).*

	**Not confident**						**Very confident**
Lose weight	1	2	3	4	5	6	7
Keep lost weight off in the long term	1	2	3	4	5	6	7
Stay at current weight	1	2	3	4	5	6	7

For peer review only - http://bmjopen.bmj.com/site/about/guidelines.xhtml

5. How important is losing weight for you at the moment? *(Please circle **one** number).*

	Not important						Very important
	1	2	3	4	5	6	7

6. The questions below ask you about why you want to lose weight. *(Please tick (✓) **one** box for each statement).*

I want to lose weight…

	Absolutely not	Somewhat	Moderately	Strongly
Because it is commonly said that living with overweight is unhealthy	☐	☐	☐	☐
To be healthier	☐	☐	☐	☐
To be more agile	☐	☐	☐	☐
For health reasons	☐	☐	☐	☐
Because I read that it is healthier	☐	☐	☐	☐
To decrease my health risks	☐	☐	☐	☐
To live long	☐	☐	☐	☐
Because acquaintances have advised me to	☐	☐	☐	☐
To not attract attention	☐	☐	☐	☐
Because I'll be more successful in my job	☐	☐	☐	☐
So I will be accepted by society	☐	☐	☐	☐
To dare to socialise again	☐	☐	☐	☐
Because I would be luckier in love	☐	☐	☐	☐
To be more appreciated/liked	☐	☐	☐	☐
To have more friends	☐	☐	☐	☐
To have better success with others	☐	☐	☐	☐
So that other people will think better of me	☐	☐	☐	☐
To be more attractive	☐	☐	☐	☐
To like to look at myself in the mirror again	☐	☐	☐	☐
Because I want to like myself more	☐	☐	☐	☐

For peer review only - http://bmjopen.bmj.com/site/about/guidelines.xhtml

I want to lose weight...	Absolutely not	Somewhat	Moderately	Strongly
Because I want to be more attractive	☐	☐	☐	☐
To be able to dress more fashionably	☐	☐	☐	☐
To fit into my clothes again	☐	☐	☐	☐
To feel more self-confident	☐	☐	☐	☐

For peer review only

Section 7: Food patterns

*(Please tick (✓) **one** box for each of the following questions).*

25. How many times a week do you eat two or more pieces of fruit?	
6 or more days a week	☐
3-5 days a week	☐
1-2 days a week	☐
Less than one day a week	☐
Never	☐

26. When eating cheese, how often do you choose reduced fat cheese in preference to regular cheese?	
Always	☐
Usually	☐
Occasionally	☐
Rarely	☐
Never	☐
I don't eat cheese	☐

27. How many days a week do you eat fried food with a batter or bread crumb coating?	
4 or more days a week	☐
2 or 3 days a week	☐
Once a week	☐
Less than once a week	☐
Never	☐

28. How often do you eat fried or roasted vegetables?	
Always	☐
Usually	☐
Occasionally	☐
Rarely	☐
Never	☐

29. When eating bread (as toast, sandwiches or a snack) how often do you spread butter or margarine on it?	
Always	☐
Usually	☐
Occasionally	☐
Rarely	☐
Never	☐

For peer review only

For peer review only - http://bmjopen.bmj.com/site/about/guidelines.xhtml

**30. How many portions of vegetables do you eat in a typical day? (a portion is about three tablespoons of vegetables or a small bowl of salad).**	
5 or more portions	☐
3 or 4 portions	☐
1 or 2 portions	☐
Less than one portion per day	☐
None	☐

**31. How often do you trim all the visible fat off the meat you eat?**	
Always	☐
Usually	☐
Occasionally	☐
Rarely	☐
Never	☐
I don't eat meat	☐

**32. How many times a week do you eat meat pies, pasties or sausage rolls?**	
3 or more times a week	☐
Once or twice a week	☐
Once a fortnight	☐
Less than once a fortnight	☐
Never	☐

**33. How often do you (or the person who cooks for you) remove the skin from chicken before it is cooked?**	
Always	☐
Usually	☐
Occasionally	☐
Rarely	☐
Never	☐
I don’t eat chicken	☐

**34. How many days a week do you eat fried potato (e.g. hot chips or potato crisps)?**	
6 or more days a week	☐
3-5 days a week	☐
1-2 days a week	☐
Less than one day a week	☐
Never	☐

For peer review only - http://bmjopen.bmj.com/site/about/guidelines.xhtml

**35. How many days a week do you eat take-away foods such as: fried or BBQ chicken; fish and chips; Chinese; pizza; hamburger etc.?**	
6 or more days a week	☐
3-5 days a week	☐
1-2 days a week	☐
Less than one day a week	☐
Never	☐

**36. How often do you (or the person who cooks for you) use fat when cooking? (e.g. butter, margarine, oil, lard etc)**	
Always	☐
Usually	☐
Occasionally	☐
Rarely	☐
Never	☐

**37. How often do you choose wholemeal spaghetti or pasta in preference to regular spaghetti or pasta?**	
Always	☐
Usually	☐
Occasionally	☐
Rarely	☐
Never	☐
I don't eat spaghetti/pasta	☐

**38. How often do you choose wholemeal bread in preference to white bread?**	
Always	☐
Usually	☐
Occasionally	☐
Rarely	☐
Never	☐

**39. How many days a week do you eat legumes? (e.g. baked beans, three bean mix, lentils, split peas, dried beans etc)**	
4 or more days a week	☐
2 or 3 days a week	☐
Once a week	☐
Less than once a week	☐
Never	☐

For peer review only - http://bmjopen.bmj.com/site/about/guidelines.xhtml

**40. How many days a week do you eat a high fibre breakfast cereal? (e.g. Weetabix, All-Bran, untoasted muesli, porridge)**	
6 or more days a week	☐
3-5 days a week	☐
1-2 days a week	☐
Less than one day a week	☐
Never	☐

1065

**41. How many different types of vegetables would you eat on a typical day?**	
5 or more types	☐
4 types	☐
3 types	☐
1 or 2 types	☐
None	☐

1066

**42. How many days a week do you eat high fat cheeses? (e.g. cheddar or cream cheese)**	
6 or more days a week	☐
3-5 days a week	☐
1-2 days a week	☐
Less than one day a week	☐
Never	☐

1067

**43. How often do you choose low-fat milk (semi-skimmed or skimmed) in preference to whole milk?**	
Always	☐
Usually	☐
Occasionally	☐
Rarely	☐
Never	☐
I don't drink milk	☐

1068

**44. How many days a week do you eat processed meats? (e.g. bacon, salami, ham etc)**	
4 or more days a week	☐
2 or 3 days a week	☐
Once a week	☐
Less than one day a week	☐
Never	☐

For peer review only

**45. How often do you eat or drink any of the following?** *(Please tick (✓)* ***one*** *box for each row).*	**More than once a day**	**Once a day**	**3-6 days a week**	**1-2 days a week**	**less than weekly**	**Never**
Sweets, chocolate bars or biscuits (including wrapped chocolate biscuits like Twix or KitKat)	☐	☐	☐	☐	☐	☐
Buns, cakes or pastries or desserts	☐	☐	☐	☐	☐	☐
Fizzy drinks or squashes that contain sugar (e.g. coca cola, Ribena, Club Orange)	☐	☐	☐	☐	☐	☐
Diet drinks (e.g. Diet Coke, Sprite Zero, Diet Club)	☐	☐	☐	☐	☐	☐

**46. How often do you have a drink containing alcohol?**	
Never	☐
Monthly or less	☐
2 - 4 times a month	☐
2 - 3 times a week	☐
4 or more times a week	☐

**47. Do you keep a record of what you eat and drink? (e.g. writing it down, using an App etc).**	
Yes	☐
No	☐

**48. Do you set yourself food and drink related goals?** *(Please tick (✓)* ***one*** *box).*	
Yes	☐
No	☐

For peer review only - http://bmjopen.bmj.com/site/about/guidelines.xhtml

<u>Section 8: Physical activity</u>

We are interested in finding out about the kinds of physical activities that people do as part of their everyday lives. The questions will ask you about the time you spent being physically active **<u>in the last 7 days</u>**. Please answer each question even if you do not consider yourself to be an active person. Please think about the activities you do at work, as part of your house and yard work, to get from place to place, and in your spare time for recreation, exercise or sport.

<u>VIGOROUS ACTIVITIES</u>

Think about all the **vigorous** activities that you did **in the last 7 days**. **Vigorous** physical activities refer to activities that take hard physical effort and make you breathe much harder than normal. Think *only* about those physical activities that you did for at least 10 minutes at a time.

1. During the <u>last 7 days</u>, on how many days did you do <u>vigorous</u> physical activities like heavy lifting, digging, aerobics, or fast bicycling?

_________ Days per week

☐ No vigorous physical activities ⟶ ***Skip to question 3***

2. How much time did you usually spend doing <u>vigorous</u> physical activities on one of those days?
*(Please answer in hours **AND** minutes. E.g. If you did 30 minutes of vigorous exercise, you can enter '0' in the Hours box and '30' in the Minutes box)*

_________ Hours per day

_________ Minutes per day

☐ Don't know/not sure

For peer review only

For peer review only - http://bmjopen.bmj.com/site/about/guidelines.xhtml

MODERATE ACTIVITIES

Think about all the **moderate** activities that you did **in the last 7 days**. **Moderate** activities refer to activities that take moderate physical effort and make you breathe somewhat harder than normal. Think only about those physical activities that you did for at least 10 minutes at a time.

3. During the last 7 days, on how many days did you do moderate physical activities like carrying light loads, bicycling at a regular pace, or doubles tennis? Do not include walking.

________ Days per week

☐ No moderate physical activities → ***Skip to question 5***

4. How much time did you usually spend doing moderate physical activities on one of those days?

*(Please answer in hours **AND** minutes. E.g. If you did 30 minutes of moderate exercise, you can enter '0' in the Hours box and '30' in the Minutes box)*

________ Hours per day

________ Minutes per day

☐ Don't know/not sure

WALKING

Think about the time you spent **walking in the last 7 days**. This includes at work and at home, walking to travel from place to place, and any other walking that you have done solely for recreation, sport, exercise, or leisure.

5. During the last 7 days, on how many days did you walk for at least 10 minutes at a time?

________ Days per week

☐ No walking → ***Skip to question 7***

6. How much time did you usually spend walking on one of those days?

*(Please answer in hours **AND** minutes. E.g. If you walked for 2 hours, you can enter '2' in the Hours box and '0' in the Minutes box)*

________ Hours per day

________ Minutes per day

☐ Don't know/not sure

SITTING

For peer review only - http://bmjopen.bmj.com/site/about/guidelines.xhtml

The last question is about the time you spent **sitting** on weekdays during the **last 7 days**. Include time spent at work, at home, while doing course work and during leisure time. This may include time spent sitting at a desk, visiting friends, reading, or sitting or lying down to watch television.

7. During the <u>last 7 days,</u> how much time did you spend <u>sitting</u> on a <u>week day</u>? *(Please answer in hours **AND** minutes. E.g. If you spent 8 hours sitting, you can enter '8' in the Hours box and '0' in the Minutes box)*

________	Hours per day
________	Minutes per day
☐	Don't know/not sure

STEPS

8. Do you use a step counter? (e.g. pedometer, App, Fitbit etc) *(Please tick (✓) **one** box).*

Yes	☐
No	☐

9. Do you set yourself physical activity related goals? *(Please tick (✓) **one** box).*

Yes	☐
No	☐

10. Did you have any problem/s that limited your physical activity during the past 3 months? *(Please tick (✓) **one** box).*

Yes	☐	If yes, please give details:
No	☐	

For peer review only

For peer review only - http://bmjopen.bmj.com/site/about/guidelines.xhtml

Section 9: Infant Feeding

The questions in this section are designed to explore how you feed your youngest child. If your most recent pregnancy was a twin/multiple pregnancy, please complete these questions for the youngest twin/multiple.

4. What is your current method of feeding for your youngest child? *(Please tick (✓) **one** box for each row).*

Breast feeding	☐ yes	☐ no
Infant formula feeding	☐ yes	☐ no
Combined feeding (Breast and infant formula)	☐ yes	☐ no
Follow-on milk	☐ yes	☐ no
Baby is on solids	☐ yes	☐ no

NOTE: If you ticked 'Baby is on solids', **please go to question 2 below.**

If you did not tick 'Baby is on solids', **please go to Section 10 on Page 35.**

5. How often do you usually give your baby these particular TYPES of solid food? *(Please tick (✓) **one** box for each food).*

	More than once a day	Once a day	3 or more times a week	Once or twice a week	Less than once a week	Never
Breakfast Cereals	☐	☐	☐	☐	☐	☐
Rice or Pasta	☐	☐	☐	☐	☐	☐
Bread	☐	☐	☐	☐	☐	☐
Potatoes	☐	☐	☐	☐	☐	☐
Potato products (incl. chips, waffles, shapes)	☐	☐	☐	☐	☐	☐
Butter/Margarine and other spreads	☐	☐	☐	☐	☐	☐
Red meat	☐	☐	☐	☐	☐	☐
Processed meat (e.g. ham)	☐	☐	☐	☐	☐	☐

For peer review only - http://bmjopen.bmj.com/site/about/guidelines.xhtml

	More than once a day	Once a day	3 or more times a week	Once or twice a week	Less than once a week	Never
Chicken/other poultry	☐	☐	☐	☐	☐	☐
Fish (incl. tuna)	☐	☐	☐	☐	☐	☐
Eggs	☐	☐	☐	☐	☐	☐
Beans, lentils, chickpeas	☐	☐	☐	☐	☐	☐
Tofu, Quorn, textured vegetable protein	☐	☐	☐	☐	☐	☐
Nuts	☐	☐	☐	☐	☐	☐
Fruit	☐	☐	☐	☐	☐	☐
Vegetables	☐	☐	☐	☐	☐	☐
Cheese, yoghurt, fromage frais	☐	☐	☐	☐	☐	☐
Puddings or desserts	☐	☐	☐	☐	☐	☐
Biscuits, sweets, chocolate or cakes	☐	☐	☐	☐	☐	☐
Crisps and corn snacks	☐	☐	☐	☐	☐	☐
Follow on formula	☐	☐	☐	☐	☐	☐
Or something else (please tick and then write below)	☐	☐	☐	☐	☐	☐

6. Do you ever add salt to your baby's solid food, including adding salt when the food is being cooked? *(Please tick (✓)* ***one*** *box).*

Yes, often	☐
Yes, sometimes	☐
Never	☐

For peer review only - http://bmjopen.bmj.com/site/about/guidelines.xhtml

Section 10: Mood

*(Please tick (✓) **one** box for each statement below).*

In the past 7 days...

**9. I have been able to laugh and see the funny side of things.**	
As much as I always could	☐
Not quite so much now	☐
Definitely not so much now	☐
Not at all	☐

**10. I have looked forward with enjoyment to things.**	
As much as I ever did	☐
Rather less than I used to	☐
Definitely less than I used to	☐
Hardly at all	☐

**11. I have blamed myself unnecessarily when things went wrong.**	
Yes, most of the time	☐
Yes, some of the time	☐
Not very often	☐
No, never	☐

**12. I have been anxious or worried for no good reason.**	
No, not at all	☐
Hardly ever	☐
Yes, sometimes	☐
Yes, very often	☐

**13. I have felt scared or panicky for no very good reason.**	
Yes, quite a lot	☐
Yes, sometimes	☐
No, not much	☐
No, not at all	☐

For peer review only - http://bmjopen.bmj.com/site/about/guidelines.xhtml

**14. Things have been getting on top of me.**	
Yes, most of the time I haven't been able to cope at all	☐
Yes, sometimes I haven't been coping as well as usual	☐
No, most of the time I have coped quite well	☐
No, I have been coping as well as ever	☐

**15. I have been so unhappy that I have had difficulty sleeping.**	
Yes, most of the time	☐
Yes, sometimes	☐
Not very often	☐
No, not at all	☐

**16. I have felt sad or miserable.**	
Yes, most of the time	☐
Yes, quite often	☐
Not very often	☐
No, not at all	☐

**9. I have been so unhappy that I have been crying.**	
Yes, most of the time	☐
Yes, quite often	☐
Only occasionally	☐
No, never	☐

**10. The thought of harming myself has occurred to me.**	
Yes, quite often	☐
Sometimes	☐
Hardly ever	☐
Never	☐

For peer review only

*(Please tick (✓) **one** box for each statement below).*

<u>Over the last two weeks</u>, how often have you been bothered by the following problems?

**18. Feeling nervous, anxious, or on edge?**	
Not at all	☐
Several days	☐
More than half of the days	☐
Nearly every day	☐

**19. Not being able to stop or control worrying?**	
Not at all	☐
Several days	☐
More than half of the days	☐
Nearly every day	☐

**20. Worrying too much about different things?**	
Not at all	☐
Several days	☐
More than half of the days	☐
Nearly every day	☐

**21. Trouble relaxing?**	
Not at all	☐
Several days	☐
More than half of the days	☐
Nearly every day	☐

**22. Being so restless that it is hard to sit still?**	
Not at all	☐
Several days	☐
More than half of the days	☐
Nearly every day	☐

For peer review only

For peer review only - http://bmjopen.bmj.com/site/about/guidelines.xhtml

**23. Becoming easily annoyed or irritable?**	
Not at all	☐
Several days	☐
More than half of the days	☐
Nearly every day	☐

**24. Feeling afraid as if something awful might happen?**	
Not at all	☐
Several days	☐
More than half of the days	☐
Nearly every day	☐

Having a young baby or child can bring up lots of different feelings and emotions. The next questions are designed to explore how you are feeling about being a parent to your baby/child. Answering these questions will help us to understand how things are going for you. If your most recent pregnancy was a twin/multiple pregnancy, please complete these questions for the youngest twin/multiple.

**25. Has your child reached their second birthday?** *(Please tick (✓)* ***one*** *box).*		
No, my child has not yet had their second birthday	☐	→ Please go to question 19 on page 39.
Yes, my child has celebrated their second birthday	☐	→ Please go to question 21 on Page 41.

For peer review only - http://bmjopen.bmj.com/site/about/guidelines.xhtml

19. Thinking about your feelings about your baby, choose the response for each statement that feels right to you. *(Please tick (✓)* ***one*** *box for each of the following statements).*

	Never	Sometimes	Always
I enjoy looking after my baby	☐	☐	☐
I feel irritated with my baby when we are together	☐	☐	☐
I feel affectionate towards my baby	☐	☐	☐
I feel that my baby is being difficult or trying to upset me on purpose	☐	☐	☐
I can work out what my baby needs from me	☐	☐	☐
I feel like I can't do things I enjoy because of my baby.	☐	☐	☐
I feel the changes in my life are worth it to look after my baby.	☐	☐	☐
I miss my baby when we are not together	☐	☐	☐
I feel like I'm looking after my baby for someone else.	☐	☐	☐
When we've been apart I look forward to seeing my baby again.	☐	☐	☐
I enjoy playing with my baby.	☐	☐	☐

20. Thinking about your baby, please tick one of the choices for each of the questions below. There are no 'right' or 'wrong' answers; many of these are

For peer review only - http://bmjopen.bmj.com/site/about/guidelines.xhtml

true of all babies at times. *(Please tick (✓) **one** box for each of the following statements).*

	Always	Very often	Quite often	Sometimes	Rarely	Never
My baby smiles at me.	☐	☐	☐	☐	☐	☐
My baby annoys me.	☐	☐	☐	☐	☐	☐
My baby likes doing things with me.	☐	☐	☐	☐	☐	☐
My baby 'talks' to me.	☐	☐	☐	☐	☐	☐
My baby irritates me.	☐	☐	☐	☐	☐	☐
My baby likes me.	☐	☐	☐	☐	☐	☐
My baby wants too much attention.	☐	☐	☐	☐	☐	☐
My baby laughs.	☐	☐	☐	☐	☐	☐
My baby gets moody.	☐	☐	☐	☐	☐	☐
My baby dominates me.	☐	☐	☐	☐	☐	☐
My baby likes to please me.	☐	☐	☐	☐	☐	☐
My baby cries for no obvious reason.	☐	☐	☐	☐	☐	☐
My baby is affectionate towards me.	☐	☐	☐	☐	☐	☐
My baby winds me up.	☐	☐	☐	☐	☐	☐

Please go to section 11 on Page 43

For peer review only - http://bmjopen.bmj.com/site/about/guidelines.xhtml

21. Thinking about your feelings about your child, choose the response for each statement that feels right to you. *(Please tick (✓)* ***one*** *box for each of the following statements).*

	Never	Sometimes	Always
I enjoy looking after my child	☐	☐	☐
I feel irritated with my child when we are together	☐	☐	☐
I feel affectionate towards my child	☐	☐	☐
I feel that my child is being difficult or trying to upset me on purpose	☐	☐	☐
I can work out what my child needs from me	☐	☐	☐
I feel like I can't do things I enjoy because of my child.	☐	☐	☐
I feel the changes in my life are worth it to look after my child.	☐	☐	☐
I miss my child when we are not together	☐	☐	☐
I feel like I'm looking after my child for someone else.	☐	☐	☐
When we've been apart I look forward to seeing my child again.	☐	☐	☐
I enjoy playing with my child.	☐	☐	☐

22. Thinking about your child, please tick one of the choices for each of the questions below. There are no 'right' or 'wrong' answers; many of these are

For peer review only - http://bmjopen.bmj.com/site/about/guidelines.xhtml

true of all children at times. *(Please tick (✓)* ***one*** *box for each of the following statements).*

	Always	Very often	Quite often	Sometimes	Rarely	Never
My child smiles at me.	☐	☐	☐	☐	☐	☐
My child annoys me.	☐	☐	☐	☐	☐	☐
My child likes doing things with me.	☐	☐	☐	☐	☐	☐
My child talks to me.	☐	☐	☐	☐	☐	☐
My child irritates me.	☐	☐	☐	☐	☐	☐
My child likes me.	☐	☐	☐	☐	☐	☐
My child wants too much attention.	☐	☐	☐	☐	☐	☐
My child laughs.	☐	☐	☐	☐	☐	☐
My child gets moody.	☐	☐	☐	☐	☐	☐
My child dominates me.	☐	☐	☐	☐	☐	☐
My child likes to please me.	☐	☐	☐	☐	☐	☐
My child cries for no obvious reason.	☐	☐	☐	☐	☐	☐
My child is affectionate towards me.	☐	☐	☐	☐	☐	☐
My child winds me up.	☐	☐	☐	☐	☐	☐

review only

For peer review only - http://bmjopen.bmj.com/site/about/guidelines.xhtml

Section 11: Self-esteem

Below is a list of statements dealing with your general feelings about yourself.

(Please tick (✓) ***one*** *box to indicate how much you agree or disagree with each of the following statements).*

	Strongly Agree	Agree	Disagree	Strongly disagree
I feel that I'm a person of worth, at least on an equal plane with others.	☐	☐	☐	☐
I feel that I have a number of good qualities.	☐	☐	☐	☐
All in all, I am inclined to feel that I am a failure.	☐	☐	☐	☐
I am able to do things as well as most other people.	☐	☐	☐	☐
I feel I do not have much to be proud of.	☐	☐	☐	☐
I take a positive attitude toward myself.	☐	☐	☐	☐
On the whole I am satisfied with myself.	☐	☐	☐	☐
I wish I could have more respect for myself.	☐	☐	☐	☐
I certainly feel useless at times.	☐	☐	☐	☐
At times I think I am no good at all.	☐	☐	☐	☐

For peer review only - http://bmjopen.bmj.com/site/about/guidelines.xhtml

Section 12: Sleep

1. At the moment, how often does your baby sleep all night? *(Please tick (✓)* ***one*** *box).*

Always	☐
Mostly	☐
Sometimes	☐
Rarely	☐
Never	☐

The following questions relate to **your** usual sleep habits during the **past month only**. Your answers should indicate the most accurate reply for the majority of days and nights in the past month. Please answer all questions.

2. During the past month, what time have you usually gone to bed at night?

Usual bed time ☐

3. During the past month, how long (in minutes) has it usually taken you to fall asleep each night?

Number of minutes ☐

4. During the past month, what has been your usual getting up time in the morning?

Usual getting up time ☐

5. During the past month, on average how many hours of actual sleep did you get at night? (This may be different than the number of hours you spent in bed.)

Hours of sleep per night ☐

6. During the past month, how often have you had trouble sleeping because you…

*(Please tick (✓) **one** box for each statement).*

	Not during the past month	Less than once a week	Once or twice a week	Three or more times a week
Cannot get to sleep within 30 minutes	☐	☐	☐	☐
Wake up in the middle of the night or early morning	☐	☐	☐	☐
Have to get up to use the bathroom	☐	☐	☐	☐
Cannot breathe comfortably	☐	☐	☐	☐
Cough or snore loudly	☐	☐	☐	☐
Feel too cold	☐	☐	☐	☐
Feel too hot	☐	☐	☐	☐
Had bad dreams	☐	☐	☐	☐
Have pain	☐	☐	☐	☐
Have to take care of your baby	☐	☐	☐	☐
Other reason(s), please describe and indicate how often during the past month you had trouble sleeping because of this:				
☐	☐	☐	☐	☐
☐	☐	☐	☐	☐
☐	☐	☐	☐	☐

7. During the past month, how would you rate your sleep quality overall?

*(Please tick (✓) **one** box).*

Very Good	Fairly Good	Fairly Bad	Very Bad

☐	☐	☐	☐

8. (Please tick (✓) ***one*** *box for each question below.)*	Not during the past month	Less than once a week	Once or twice a week	Three or more times a week
**During the past month, how often have you taken medicine (prescribed or "over the counter") to help you sleep?**	☐	☐	☐	☐
**During the past month, how often have you had trouble staying awake while driving, eating meals, or engaging in social activity?**	☐	☐	☐	☐

9. During the past month, how much of a problem has it been for you to keep up enough enthusiasm to get things done? (Please tick *(✓)* ***one*** *box).*

No problem at all	Only a very slight problem	Somewhat of a problem	A very big problem
☐	☐	☐	☐

10. Do you usually have a bed partner or room-mate? *(Please tick (✓)* ***one*** *box).*

No bed partner or room-mate	Partner/room-mate in other room	Partner in same room but not in same bed	Partner in same bed
☐	☐	☐	☐

For peer review only - http://bmjopen.bmj.com/site/about/guidelines.xhtml

11. If you usually have a room-mate or bed partner, ask him/her how often in the past month you have had:

Please tick (✓) ***one*** *box for each statement).*	**Not during the past month**	**Less than once a week**	**Once or twice a week**	**Three or more times a week**
Loud snoring	☐	☐	☐	☐
Long pauses between breaths while asleep	☐	☐	☐	☐
Legs twitching or jerking while you sleep	☐	☐	☐	☐
Episodes of confusion during sleep	☐	☐	☐	☐
Other restlessness while you sleep, please describe and indicate how often in the past month you have had this:				
	☐	☐	☐	☐
	☐	☐	☐	☐
	☐	☐	☐	☐

For peer review only - http://bmjopen.bmj.com/site/about/guidelines.xhtml

You have now completed the questionnaire. Thank you very much for your time and effort! We really appreciate your help with our research.

For peer review only

For peer review only - http://bmjopen.bmj.com/site/about/guidelines.xhtml

Supporting MumS (SMS) study Questionnaire Booklet

Month 12 Control Group

**Participant ID:**	_ _ _ _ _
**Researcher name:**	
**Today's date:**	_ _ / _ _ / _ _ _ _<br>**e.g. 05 / 01 / 2017**

For peer review only - http://bmjopen.bmj.com/site/about/guidelines.xhtml

About this questionnaire booklet

Thank you for taking the time to fill out this questionnaire booklet.

The questionnaire booklet is made up of questions that will help us find out more about your experience of the postpartum period. Please complete the questions as accurately as you can. **There are no right or wrong answers**. If there are any questions you do not wish to answer, you can leave them blank. Note that your responses will be **anonymous,** this means your name will never be given with your responses.

The questionnaire booklet is split up into the short sections shown below. You can fill it all out at once, or do it in chunks, as your time allows.

**Section 1**	**SMS evaluation**	**Pages 3-6**
**Section 2**	**Health and well-being**	**Pages 7-9**
**Section 3**	**Health resources**	**Pages 10-12**
**Section 4**	**Eating and activity approaches**	**Pages 13-17**
**Section 5**	**Social support**	**Page 18-19**
**Section 6**	**Lifestyle behaviours and attitudes**	**Page 20-22**
**Section 7**	**Food patterns**	**Page 23-27**
**Section 8**	**Physical activity**	**Page 28-30**
**Section 9**	**Infant feeding**	**Page 31-32**
**Section 10**	**Mood**	**Page 33-40**
**Section 11**	**Self-esteem**	**Page 41**
**Section 12**	**Sleep**	**Page 42-45**

For peer review only - http://bmjopen.bmj.com/site/about/guidelines.xhtml

Section 1: SMS Evaluation

Thank you for participating in our study. We would like to ask you some questions about how you have found participating in this research. Your responses to this survey will help us to improve the studies we carry out in the future.

3. How satisfied were you with your overall experience of the SMS study? *(Please tick (✓)* ***one*** *box).*

Very Dissatisfied	Mostly Dissatisfied	Neither Satisfied Nor Dissatisfied	Mostly Satisfied	Very Satisfied
☐	☐	☐	☐	☐

If you were very or mostly dissatisfied, please explain what could have been improved:

____

____

____

4. Please answer the questions below about your experience of taking part in the SMS study. *(Please tick (✓)* ***one*** *box to indicate how much you agree or disagree with each of the following statements).*

	Strongly disagree	Disagree	Neither agree or disagree	Agree	Strongly Agree
At the start of the study, the information I was given about the study was clear and informative	☐	☐	☐	☐	☐
The amount of information and level of support provided by the researchers during the study was suited to my needs	☐	☐	☐	☐	☐

If you have disagreed or strongly disagreed with any of the above statements, please explain what could have been improved:

____

____

____

For peer review only - http://bmjopen.bmj.com/site/about/guidelines.xhtml

For peer review only

3. How satisfied were you with the text messages? *(Please tick (✓) **one** box).*

Very Dissatisfied	Mostly Dissatisfied	Neither Satisfied Nor Dissatisfied	Mostly Satisfied	Very Satisfied
☐	☐	☐	☐	☐

If you were very or mostly dissatisfied, please explain what could have been improved:

____

____

____

13. Please answer the questions below about your experience of the text messages you received during the SMS study. *(Please tick (✓) **one** box to indicate how much you agree or disagree with each of the following statements).*

	Strongly Disagree	Disagree	Neither agree or disagree	Agree	Strongly agree
SMS texts were easy to understand	☐	☐	☐	☐	☐
SMS texts were helpful	☐	☐	☐	☐	☐
SMS texts were interesting	☐	☐	☐	☐	☐
An appropriate amount of SMS texts were sent during the study	☐	☐	☐	☐	☐
SMS texts were delivered at appropriate times of the day	☐	☐	☐	☐	☐

If you have disagreed or strongly disagreed with any of the above statements, please explain what could have been improved:

____

____

____

For peer review

For peer review only - http://bmjopen.bmj.com/site/about/guidelines.xhtml

14. During the study you were asked to attend 3 study visits. Please rate how easy or difficult you found each aspect of these visits. *(Please tick (✓) **one** response that most applies to you in relation to your experience).*

	Very difficult	Difficult	No strong opinion	Easy	Very easy
Location of visits	☐	☐	☐	☐	☐
Length of visits	☐	☐	☐	☐	☐
Having your height measurement taken	☐	☐	☐	☐	☐
Having your waist measurements taken	☐	☐	☐	☐	☐
Having your weight taken	☐	☐	☐	☐	☐
Completing the questionnaires	☐	☐	☐	☐	☐

If you have felt that any aspects of the visits were difficult, please explain how they could have been improved:

____

____

____

For peer review only

15. Based on your experience in taking part in SMS, do you think the programme should be offered to other mums? *(Please tick (✓)* ***one*** *response).*

Yes ☐

No ☐

If you answered no, please explain why:

____

____

____

16. Do you have any other comments? We would welcome any suggestions you have regarding how we could improve future studies for those taking part:

____

____

____

For peer review only

Section 2: Health and well-being

For questions 1-5, under each heading, please tick (✓) the **ONE** box that best describes your health TODAY.

**1. Mobility**	
I have no problems in walking about	☐
I have slight problems in walking about	☐
I have moderate problems in walking about	☐
I have severe problems in walking about	☐
I am unable to walk about	☐

**2. Self-care**	
I have no problems washing or dressing myself	☐
I have slight problems washing or dressing myself	☐
I have moderate problems washing or dressing myself	☐
I have severe problems washing or dressing myself	☐
I am unable to wash or dress myself	☐

**3. Usual Activities** *(e.g. work, study, housework, family or leisure activities)*	
I have no problems doing my usual activities	☐
I have slight problems doing my usual activities	☐
I have moderate problems doing my usual activities	☐
I have severe problems doing my usual activities	☐
I am unable to do my usual activities	☐

**4. Pain/ Discomfort**	
I have no pain or discomfort	☐
I have slight pain or discomfort	☐
I have moderate pain or discomfort	☐
I have severe pain or discomfort	☐
I have extreme pain or discomfort	☐

For peer review only - http://bmjopen.bmj.com/site/about/guidelines.xhtml

5. Anxiety/ Depression	
I am not anxious or depressed	☐
I am slightly anxious or depressed	☐
I am moderately anxious or depressed	☐
I am severely anxious or depressed	☐
I am extremely anxious or depressed	☐

6. We would like to know how good or bad your health is TODAY.

This scale is numbered from 0 to 100.

100 means the <u>best</u> health you can imagine.

0 means the <u>worst</u> health you can imagine.

Please mark an X on the scale to indicate how your health is TODAY.

Now, write the number you marked on the scale in the box below.

Your health today = ☐

The best health you can imagine

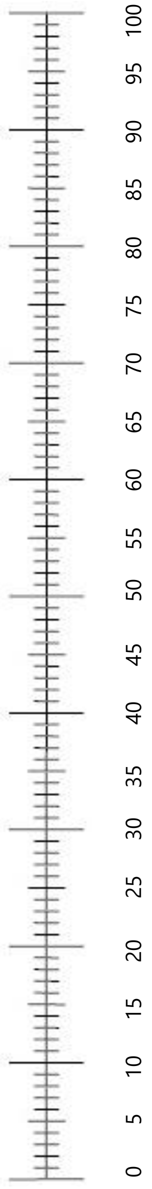

The worst health you can imagine

For peer review only - http://bmjopen.bmj.com/site/about/guidelines.xhtml

7. Feeling settled and secure *(Please tick (✓)* ***one*** *box that best describes your overall quality of life at the moment).*

I am able to feel settled and secure in **all** areas of my life	☐
I am able to feel settled and secure in **many** areas of my life	☐
I am able to feel settled and secure in **a few** areas of my life	☐
I am unable to feel settled and secure in **any** areas of my life	☐

1365

8. Love, friendship and support *Please tick (✓)* ***one*** *box that best describes your overall quality of life at the moment).*

I can have **a lot** of love, friendship and support	☐
I can have **quite a lot** of love, friendship and support	☐
I can have **a little** love, friendship and support	☐
I **cannot** have **any** love, friendship and support	☐

1366

9. Being independent *Please tick (✓)* ***one*** *box that best describes your overall quality of life at the moment).*

I am able to be **completely** independent	☐
I am able to be independent in **many** things	☐
I am able to be independent in **a few** things	☐
I am **unable** to be at all independent	☐

1367

10. Achievement and progress *Please tick (✓)* ***one*** *box that best describes your overall quality of life at the moment).*

I can achieve and progress in **all** aspects of my life	☐
I can achieve and progress in **many** aspects of my life	☐
I can achieve and progress in **a few** aspects of my life	☐
I cannot achieve and progress in **any** aspects of my life	☐

1368

11. Enjoyment and pleasure *Please tick (✓)* ***one*** *box that best describes your overall quality of life at the moment).*

I can have **a lot** of enjoyment and pleasure	☐
I can have **quite a lot** of enjoyment and pleasure	☐
I can have **a little** enjoyment and pleasure	☐
I **cannot** have **any** enjoyment and pleasure	☐

For peer review only - http://bmjopen.bmj.com/site/about/guidelines.xhtml

Section 3: Health resources

2. 1. In the last 3 months, have you seen any health professional at your GP surgery? (this includes phone/online or face-to-face appointments) *(Please tick (✓) **one** box).*

Yes	☐
No	☐

If YES, how many times were you seen by: *(Please enter a number)*

GP	
Practice nurse	
Other (please state)	
Other (please state)	

2. In the last 3 months, have you attended an Accident and Emergency (Casualty) department? *(Please tick (✓) **one** box).*

Yes	☐	If YES, how many times?	
No	☐		

3. In the last 3 months, have you attended hospital as an out-patient? *(Please tick (✓) **one** box).*

Yes	☐	If YES, how many times?	
No	☐		

4. In the last 3 months, have you attended hospital as an in-patient? *(Please tick (✓) **one** box).*

Yes	☐	If YES, how many times?	
		If YES, how many nights did you spend in hospital in the last 3 months in total?	
No	☐		

5. In the last 3 months, have you received any prescriptions for medicine? *(Please tick (✓) **one** box).*

Yes	☐	If YES, how many prescriptions have you received?	
No	☐		

For peer review only - http://bmjopen.bmj.com/site/about/guidelines.xhtml

**6. In the last 3 months, have you purchased any over-the-counter medications or lifestyle products (e.g. slimming aids, vitamin supplements)?** *(Please tick (✓) **one** box).*			
Yes	☐	If YES, please state total amount spent in last 3 months	£
No	☐		

1374

**7. In the last 3 months have you paid for any services or clubs for the specific purpose of helping you with your lifestyle - for example slimming clubs, health clubs, gyms, swimming pools, exercise classes?** *(Please tick (✓) **one** box).*			
Yes	☐	If YES, approximately how much have you spent in total on all of these services/clubs in the last 3 months?	£
No	☐		

8. Please think about last week's food and drink purchases for your household (i.e. you and your family) and tell us the cost to your household of the following:
*(Please write '**0**' if you don't purchase a specific type of food/product) .*

Type of food and drink purchased	Weekly Cost to you (your household)
Food and non-alcoholic drinks (e.g. supermarket shopping)	£
Alcoholic drinks e.g. wine & beer	£
Takeaway meals and snacks eaten AT HOME e.g. pizza delivery	£
Meals, snacks and drinks CONSUMED AWAY FROM HOME (e.g. restaurant)	£
Cigarettes or vaping products	£

1375

**9. Have you previously purchased any apps for improving your fitness/health or wellbeing?** *(Please tick (✓) **one** box).*			
Yes	☐	If YES, Please state the total amount you spent on fitness/health apps in the last year?	£
No	☐		

1376

For peer review only - http://bmjopen.bmj.com/site/about/guidelines.xhtml

10. On average, <u>per day</u> how much time have you spent exercising/walking in the last 3 months in total?

*(Please answer in hours **AND** minutes. E.g. If you did 30 minutes of exercise per day, you can enter '0' under Hours and '30' under the Minutes column)*

On average per day: Hours ☐ Minutes ☐

11. Has your employment status changed in the last three months?
(Note: returning to work after maternity leave should **not** be included as a change) *(Please tick (✓) **one** box).*

Yes ☐

No ☐

If YES, what is your **current** employment status? *(Please tick (✓) **one** box)*

☐ Full-time employment
☐ Part-time employment
☐ Unemployed
☐ Student or training
☐ Retired
☐ Not working due to illness
☐ Prefer not to answer
☐ Other (please specify) ______

For peer review only

For peer review only - http://bmjopen.bmj.com/site/about/guidelines.xhtml

Section 4: Eating and activity approaches

This section asks you about your diet. *(Please circle **one** number in each row to indicate how much you agree or disagree with the following statements).*

**1. Eating healthier is something…**	**Strongly Disagree**						**Strongly Agree**
I do automatically	1	2	3	4	5	6	7
I do without having to consciously remember	1	2	3	4	5	6	7
I do without thinking	1	2	3	4	5	6	7
I start doing before I realise I'm doing it	1	2	3	4	5	6	7

**2. Over the next few months I intend to…**	**Strongly Disagree**						**Strongly Agree**
eat a healthy diet (e.g. reduce portion size, eat less sugary and high fact snacks and eat more fruit and vegetables)	1	2	3	4	5	6	7
be physically active (e.g. by walking more or taking exercise classes)	1	2	3	4	5	6	7

**3. I already have concrete plans…**	**Strongly Disagree**						**Strongly Agree**
on how to eat a healthy diet	1	2	3	4	5	6	7
on when to eat a healthy diet	1	2	3	4	5	6	7

**4. I already have concrete plans…**	**Strongly Disagree**						**Strongly Agree**
on when I need to be especially careful to maintain my healthy diet	1	2	3	4	5	6	7
about what to do in difficult situations to stick to my healthy diet	1	2	3	4	5	6	7
on how I will cope if I slip back into old eating habits	1	2	3	4	5	6	7

For peer review only - http://bmjopen.bmj.com/site/about/guidelines.xhtml

**5. I am confident that I can stick to a healthy diet even…**	**Strongly Disagree**						**Strongly Agree**
if I have to learn a lot about nutrition	1	2	3	4	5	6	7
if I initially have to watch out in many situations	1	2	3	4	5	6	7
if I have to start all over again several times until I succeed	1	2	3	4	5	6	7
if I initially have to make plans	1	2	3	4	5	6	7
if initially food doesn't taste as good	1	2	3	4	5	6	7
if I initially don't get much support	1	2	3	4	5	6	7
if it takes a long time to get used to it	1	2	3	4	5	6	7
if I have worries and troubles	1	2	3	4	5	6	7
if my partner/ my family don't change their nutrition habits	1	2	3	4	5	6	7
if I am tired	1	2	3	4	5	6	7
if I am stressed out	1	2	3	4	5	6	7
if I don't lose weight initially	1	2	3	4	5	6	7

6. In spite of good intentions when losing weight, slip-ups may occur. Imagine you relapse back into your old eating habits. How confident are you about restarting your new healthy eating habits?

**I am sure I can start my new healthy eating habits again regularly, even if I…**	**Strongly Disagree**						**Strongly Agree**
have several bad days in a row	1	2	3	4	5	6	7
lose my motivation to keep up my new healthy eating habits	1	2	3	4	5	6	7
have had a relapse and have fallen back into old eating habits for several weeks	1	2	3	4	5	6	7

For peer review only - http://bmjopen.bmj.com/site/about/guidelines.xhtml

**7. For the next few questions:**<br><br>**'Tempting foods'** are any food you want to eat more of than you think you should. **'Eating intentions'** refer to the way you are aiming to eat, for example you may intend to avoid tempting foods or eat healthy foods.<br><br>*(Please tick (✓) **one** box for each of the following statements).*	**Never**	**Rarely**	**Sometimes**	**Often**	**Always**
I give up too easily on my eating intentions	☐	☐	☐	☐	☐
I'm good at resisting tempting food	☐	☐	☐	☐	☐
I easily get distracted from the way I intend to eat	☐	☐	☐	☐	☐
If I am not eating in the way I intend to I make changes	☐	☐	☐	☐	☐
I find it hard to remember what I have eaten throughout the day	☐	☐	☐	☐	☐

<u>**This section asks about your physical activity**</u>. *(Please circle **one** number in each row to indicate how much you agree or disagree with the following statements).*

**1. Being active every day is something…**	**Strongly Disagree**						**Strongly Agree**
I do automatically	1	2	3	4	5	6	7
I do without having to consciously remember	1	2	3	4	5	6	7
I do without thinking	1	2	3	4	5	6	7
I start doing before I realise I'm doing it	1	2	3	4	5	6	7

**2. I already have concrete plans…**	**Strongly Disagree**						**Strongly Agree**
on when to be physically active	1	2	3	4	5	6	7
on where to be physically active	1	2	3	4	5	6	7
on how to be physically active	1	2	3	4	5	6	7
on how often be physically active	1	2	3	4	5	6	7
on who I can be physically active with	1	2	3	4	5	6	7
**3. I already have concrete plans…**	**Strongly Disagree**						**Strongly Agree**

For peer review only - http://bmjopen.bmj.com/site/about/guidelines.xhtml

about what to do if something gets in the way	1	2	3	4	5	6	7
about what to do if I miss a physical activity session	1	2	3	4	5	6	7
about what to do in difficult situations to stick to my physical activity intentions	1	2	3	4	5	6	7
for times when I will need to be extra careful to stay committed	1	2	3	4	5	6	7

4a. Certain barriers make it hard to begin physical activity. How sure are you that you can begin exercising regularly?

**I am sure that…**	**Strongly Disagree**						**Strongly Agree**
I can change to a physically active lifestyle	1	2	3	4	5	6	7
I can be physically active once a week	1	2	3	4	5	6	7
I can be physically active at least 3 times a week for 30 minutes	1	2	3	4	5	6	7

**4b. I am sure I can start being physically active immediately, even if…**	**Strongly Disagree**						**Strongly Agree**
the planning for this takes a lot of time and effort	1	2	3	4	5	6	7
I have to force myself to start immediately	1	2	3	4	5	6	7
I have to push myself	1	2	3	4	5	6	7

For peer review only - http://bmjopen.bmj.com/site/about/guidelines.xhtml

5. Are you confident that you can manage staying physically active?

**I am sure I can keep being physically active regularly, even if...**	**Strongly Disagree**						**Strongly Agree**
it takes me a long time to make a habit	1	2	3	4	5	6	7
I am worried and troubled	1	2	3	4	5	6	7
I am tired	1	2	3	4	5	6	7
I am stressed out	1	2	3	4	5	6	7
I don't lose weight at once	1	2	3	4	5	6	7
I have to start all over again several times until I succeed	1	2	3	4	5	6	7
my partner/family isn't physically active	1	2	3	4	5	6	7

6. In spite of good intentions, slip ups may occur. How confident are you about restarting exercises?

**I am sure I can keep being physically active regularly, even if...**	**Strongly Disagree**						**Strongly Agree**
I postpone my plans several times	1	2	3	4	5	6	7
I lose my motivation	1	2	3	4	5	6	7
I have not been active for several weeks	1	2	3	4	5	6	7

For peer review only - http://bmjopen.bmj.com/site/about/guidelines.xhtml

Section 5: Social Support

1. Below is a list of statements about social support. *(Please tick (✓) **one** box in each row to indicate how much you agree or disagree with the following statements).*
Within the past <u>3 months</u>, I have got support from my friends and/or family to help me:

	Strongly Agree	Agree	Neither agree or disagree	Disagree	Strongly disagree
...eat well	☐	☐	☐	☐	☐
...be physically active	☐	☐	☐	☐	☐
...lose weight	☐	☐	☐	☐	☐

2. How often have you felt lonely during the past week?
*(Please tick (✓) **one** box).*

None, or almost none of the time	☐
Some of the time	☐
Most of the time	☐
All, or almost all of the time	☐
Don't know	☐
Do not wish to answer	☐

3. How many people can you count on in times of need?
*(Please tick (✓) **one** box).*

0	☐	→ Please go to Section 6 on Page 20
1	☐	
2	☐	
3	☐	
4	☐	
5	☐	→ Please complete the next question in this section (i.e. question 4)
6	☐	
7	☐	
8	☐	
9	☐	
10 or more	☐	

For peer review only - http://bmjopen.bmj.com/site/about/guidelines.xhtml

4. How many of these people live in your local area? That is, within about a mile or a 20 minute walk from your home.

*(Please tick (✓) **one** box).*

0	☐
1	☐
2	☐
3	☐
4	☐
5	☐
6	☐
7	☐
8	☐
9	☐
10 or more	☐

eview only

For peer review only - http://bmjopen.bmj.com/site/about/guidelines.xhtml

Section 6: Lifestyle behaviours and attitudes

7. How often do you weigh yourself currently? *(Please tick (✓) **one** box).*

Never	☐
About once a year or less	☐
Every couple of months	☐
Every month	☐
Every week	☐
Every day	☐
More than once a day	☐

8. Within the past 3 months, have you taken part in any weight loss programmes (e.g. Slimming World). *(Please tick (✓) **one** box).*

Yes	☐
No	☐

If yes, please give details below;

Name of programme	*How long you attended for*

9. Do you want to lose weight? *(Please tick (✓) **one** box).*

Yes	☐	→ Please complete the rest of the questions in this section (i.e. questions 4, 5 & 6)
No	☐	→ Please go to Section 7 on Page 23

4. How confident are you in your ability to:

*(Please circle **one** number in each row to indicate how confident you feel about each of the following statements).*	**Not confident**						**Very confident**
Lose weight	1	2	3	4	5	6	7
Keep lost weight off in the long term	1	2	3	4	5	6	7
Stay at current weight	1	2	3	4	5	6	7

For peer review only - http://bmjopen.bmj.com/site/about/guidelines.xhtml

5. How important is losing weight for you at the moment? *(Please circle **one** number).*

Not important						Very important
1	2	3	4	5	6	7

6. The questions below ask you about why you want to lose weight. *(Please tick (✓) **one** box for each statement).*

I want to lose weight…

	Absolutely not	Somewhat	Moderately	Strongly
Because it is commonly said that living with overweight is unhealthy	☐	☐	☐	☐
To be healthier	☐	☐	☐	☐
To be more agile	☐	☐	☐	☐
For health reasons	☐	☐	☐	☐
Because I read that it is healthier	☐	☐	☐	☐
To decrease my health risks	☐	☐	☐	☐
To live long	☐	☐	☐	☐
Because acquaintances have advised me to	☐	☐	☐	☐
To not attract attention	☐	☐	☐	☐
Because I'll be more successful in my job	☐	☐	☐	☐
So I will be accepted by society	☐	☐	☐	☐
To dare to socialise again	☐	☐	☐	☐
Because I would be luckier in love	☐	☐	☐	☐
To be more appreciated/liked	☐	☐	☐	☐
To have more friends	☐	☐	☐	☐
To have better success with others	☐	☐	☐	☐
So that other people will think better of me	☐	☐	☐	☐
To be more attractive	☐	☐	☐	☐
To like to look at myself in the mirror again	☐	☐	☐	☐
Because I want to like myself more	☐	☐	☐	☐

For peer review only - http://bmjopen.bmj.com/site/about/guidelines.xhtml

**I want to lose weight…**	**Absolutely not**	**Somewhat**	**Moderately**	**Strongly**
Because I want to be more attractive	☐	☐	☐	☐
To be able to dress more fashionably	☐	☐	☐	☐
To fit into my clothes again	☐	☐	☐	☐
To feel more self-confident	☐	☐	☐	☐

For peer review only

For peer review only - http://bmjopen.bmj.com/site/about/guidelines.xhtml

Section 7: Food patterns

*(Please tick (✓) **one** box for each of the following questions).*

**49. How many times a week do you eat two or more pieces of fruit?**	
6 or more days a week	☐
3-5 days a week	☐
1-2 days a week	☐
Less than one day a week	☐
Never	☐

**50. When eating cheese, how often do you choose reduced fat cheese in preference to regular cheese?**	
Always	☐
Usually	☐
Occasionally	☐
Rarely	☐
Never	☐
I don't eat cheese	☐

**51. How many days a week do you eat fried food with a batter or bread crumb coating?**	
4 or more days a week	☐
2 or 3 days a week	☐
Once a week	☐
Less than once a week	☐
Never	☐

**52. How often do you eat fried or roasted vegetables?**	
Always	☐
Usually	☐
Occasionally	☐
Rarely	☐
Never	☐

**53. When eating bread (as toast, sandwiches or a snack) how often do you spread butter or margarine on it?**	
Always	☐
Usually	☐
Occasionally	☐
Rarely	☐
Never	☐

For peer review only

For peer review only - http://bmjopen.bmj.com/site/about/guidelines.xhtml

54. How many portions of vegetables do you eat in a typical day? (a portion is about three tablespoons of vegetables or a small bowl of salad).	
5 or more portions	☐
3 or 4 portions	☐
1 or 2 portions	☐
Less than one portion per day	☐
None	☐

55. How often do you trim all the visible fat off the meat you eat?	
Always	☐
Usually	☐
Occasionally	☐
Rarely	☐
Never	☐
I don't eat meat	☐

56. How many times a week do you eat meat pies, pasties or sausage rolls?	
3 or more times a week	☐
Once or twice a week	☐
Once a fortnight	☐
Less than once a fortnight	☐
Never	☐

57. How often do you (or the person who cooks for you) remove the skin from chicken before it is cooked?	
Always	☐
Usually	☐
Occasionally	☐
Rarely	☐
Never	☐
I don't eat chicken	☐

58. How many days a week do you eat fried potato (e.g. hot chips or potato crisps)?	
6 or more days a week	☐
3-5 days a week	☐
1-2 days a week	☐
Less than one day a week	☐
Never	☐

For peer review only - http://bmjopen.bmj.com/site/about/guidelines.xhtml

**59. How many days a week do you eat take-away foods such as: fried or BBQ chicken; fish and chips; Chinese; pizza; hamburger etc.?**	
6 or more days a week	☐
3-5 days a week	☐
1-2 days a week	☐
Less than one day a week	☐
Never	☐

1466

**60. How often do you (or the person who cooks for you) use fat when cooking? (e.g. butter, margarine, oil, lard etc)**	
Always	☐
Usually	☐
Occasionally	☐
Rarely	☐
Never	☐

1467

**61. How often do you choose wholemeal spaghetti or pasta in preference to regular spaghetti or pasta?**	
Always	☐
Usually	☐
Occasionally	☐
Rarely	☐
Never	☐
I don't eat spaghetti/pasta	☐

1468

**62. How often do you choose wholemeal bread in preference to white bread?**	
Always	☐
Usually	☐
Occasionally	☐
Rarely	☐
Never	☐

1469

**63. How many days a week do you eat legumes? (e.g. baked beans, three bean mix, lentils, split peas, dried beans etc)**	
4 or more days a week	☐
2 or 3 days a week	☐
Once a week	☐
Less than once a week	☐
Never	☐

1470

For peer review only - http://bmjopen.bmj.com/site/about/guidelines.xhtml

64. How many days a week do you eat a high fibre breakfast cereal? (e.g. Weetabix, All-Bran, untoasted muesli, porridge)

6 or more days a week	☐
3-5 days a week	☐
1-2 days a week	☐
Less than one day a week	☐
Never	☐

1471

65. How many different types of vegetables would you eat on a typical day?

5 or more types	☐
4 types	☐
3 types	☐
1 or 2 types	☐
None	☐

1472

66. How many days a week do you eat high fat cheeses? (e.g. cheddar or cream cheese)

6 or more days a week	☐
3-5 days a week	☐
1-2 days a week	☐
Less than one day a week	☐
Never	☐

1473

67. How often do you choose low-fat milk (semi-skimmed or skimmed) in preference to whole milk?

Always	☐
Usually	☐
Occasionally	☐
Rarely	☐
Never	☐
I don't drink milk	☐

1474

68. How many days a week do you eat processed meats? (e.g. bacon, salami, ham etc)

4 or more days a week	☐
2 or 3 days a week	☐
Once a week	☐
Less than one day a week	☐
Never	☐

For peer review only

For peer review only - http://bmjopen.bmj.com/site/about/guidelines.xhtml

**69. How often do you eat or drink any of the following?** *(Please tick (✓)* ***one*** *box for each row).*	**More than once a day**	**Once a day**	**3-6 days a week**	**1-2 days a week**	**less than weekly**	**Never**
Sweets, chocolate bars or biscuits (including wrapped chocolate biscuits like Twix or KitKat)	☐	☐	☐	☐	☐	☐
Buns, cakes or pastries or desserts	☐	☐	☐	☐	☐	☐
Fizzy drinks or squashes that contain sugar (e.g. coca cola, Ribena, Club Orange)	☐	☐	☐	☐	☐	☐
Diet drinks (e.g. Diet Coke, Sprite Zero, Diet Club)	☐	☐	☐	☐	☐	☐

**70. How often do you have a drink containing alcohol?**	
Never	☐
Monthly or less	☐
2 - 4 times a month	☐
2 - 3 times a week	☐
4 or more times a week	☐

**71. Do you keep a record of what you eat and drink? (e.g. writing it down, using an App etc).**	
Yes	☐
No	☐

**72. Do you set yourself food and drink related goals?** *(Please tick (✓)* ***one*** *box).*	
Yes	☐
No	☐

For peer review only - http://bmjopen.bmj.com/site/about/guidelines.xhtml

Section 8: Physical activity

We are interested in finding out about the kinds of physical activities that people do as part of their everyday lives. The questions will ask you about the time you spent being physically active **in the last 7 days**. Please answer each question even if you do not consider yourself to be an active person. Please think about the activities you do at work, as part of your house and yard work, to get from place to place, and in your spare time for recreation, exercise or sport.

VIGOROUS ACTIVITIES

Think about all the **vigorous** activities that you did **in the last 7 days**. **Vigorous** physical activities refer to activities that take hard physical effort and make you breathe much harder than normal. Think *only* about those physical activities that you did for at least 10 minutes at a time.

1. During the last 7 days, on how many days did you do vigorous physical activities like heavy lifting, digging, aerobics, or fast bicycling?

________	Days per week	
☐	No vigorous physical activities →	***Skip to question 3***

2. How much time did you usually spend doing vigorous physical activities on one of those days?

*(Please answer in hours **AND** minutes. E.g. If you did 30 minutes of vigorous exercise, you can enter '0' in the Hours box and '30' in the Minutes box)*

________	Hours per day
________	Minutes per day
☐	Don't know/not sure

For peer review only

MODERATE ACTIVITIES

Think about all the **moderate** activities that you did **in the last 7 days**. **Moderate** activities refer to activities that take moderate physical effort and make you breathe somewhat harder than normal. Think only about those physical activities that you did for at least 10 minutes at a time.

3. During the last 7 days, on how many days did you do moderate physical activities like carrying light loads, bicycling at a regular pace, or doubles tennis? Do not include walking.

_______ Days per week

☐ No moderate physical activities → ***Skip to question 5***

4. How much time did you usually spend doing moderate physical activities on one of those days?

*(Please answer in hours **AND** minutes. E.g. If you did 30 minutes of moderate exercise, you can enter '0' in the Hours box and '30' in the Minutes box)*

_______ Hours per day

_______ Minutes per day

☐ Don't know/not sure

WALKING

Think about the time you spent **walking in the last 7 days**. This includes at work and at home, walking to travel from place to place, and any other walking that you have done solely for recreation, sport, exercise, or leisure.

5. During the last 7 days, on how many days did you walk for at least 10 minutes at a time?

_______ Days per week

☐ No walking → ***Skip to question 7***

6. How much time did you usually spend walking on one of those days?

*(Please answer in hours **AND** minutes. E.g. If you walked for 2 hours, you can enter '2' in the Hours box and '0' in the Minutes box)*

_______ Hours per day

_______ Minutes per day

☐ Don't know/not sure

For peer review only - http://bmjopen.bmj.com/site/about/guidelines.xhtml

SITTING

The last question is about the time you spent **sitting** on weekdays during the **last 7 days**. Include time spent at work, at home, while doing course work and during leisure time. This may include time spent sitting at a desk, visiting friends, reading, or sitting or lying down to watch television.

**7. During the last 7 days, how much time did you spend sitting on a week day?** *(Please answer in hours **AND** minutes. E.g. If you spent 8 hours sitting, you can enter '8' in the Hours box and '0' in the Minutes box)*	
_________	Hours per day
_________	Minutes per day
☐	Don't know/not sure

STEPS

**8. Do you use a step counter? (e.g. pedometer, App, Fitbit etc)** *(Please tick (✓) **one** box).*	
Yes	☐
No	☐

**9. Do you set yourself physical activity related goals?** *(Please tick (✓) **one** box).*	
Yes	☐
No	☐

**10. Did you have any problem/s that limited your physical activity during the past 3 months?** *(Please tick (✓) **one** box).*		
Yes	☐	If yes, please give details:
No	☐	

For peer review only

Section 9: Infant Feeding

The questions in this section are designed to explore how you feed your youngest child. If your most recent pregnancy was a twin/multiple pregnancy, please complete these questions for the youngest twin/multiple.

7. What is your current method of feeding for your youngest child? *(Please tick (✓)* ***one*** *box for each row).*

Breast feeding	☐ yes	☐ no
Infant formula feeding	☐ yes	☐ no
Combined feeding (Breast and infant formula)	☐ yes	☐ no
Follow-on milk	☐ yes	☐ no
Baby is on solids	☐ yes	☐ no

NOTE: If you ticked 'Baby is on solids', **please go to question 2 below**.

If you did not tick 'Baby is on solids', **please go to Section 10 on Page 33**.

8. How often do you usually give your baby these particular TYPES of solid food? *(Please tick (✓)* ***one*** *box for each food).*

	**More than once a day**	**Once a day**	**3 or more times a week**	**Once or twice a week**	**Less than once a week**	**Never**
Breakfast Cereals	☐	☐	☐	☐	☐	☐
Rice or Pasta	☐	☐	☐	☐	☐	☐
Bread	☐	☐	☐	☐	☐	☐
Potatoes	☐	☐	☐	☐	☐	☐
Potato products (incl. chips, waffles, shapes)	☐	☐	☐	☐	☐	☐
Butter/Margarine and other spreads	☐	☐	☐	☐	☐	☐
Red meat	☐	☐	☐	☐	☐	☐
Processed meat (e.g. ham)	☐	☐	☐	☐	☐	☐

For peer review only - http://bmjopen.bmj.com/site/about/guidelines.xhtml

	More than once a day	Once a day	3 or more times a week	Once or twice a week	Less than once a week	Never
Chicken/other poultry	☐	☐	☐	☐	☐	☐
Fish (incl. tuna)	☐	☐	☐	☐	☐	☐
Eggs	☐	☐	☐	☐	☐	☐
Beans, lentils, chickpeas	☐	☐	☐	☐	☐	☐
Tofu, Quorn, textured vegetable protein	☐	☐	☐	☐	☐	☐
Nuts	☐	☐	☐	☐	☐	☐
Fruit	☐	☐	☐	☐	☐	☐
Vegetables	☐	☐	☐	☐	☐	☐
Cheese, yoghurt, fromage frais	☐	☐	☐	☐	☐	☐
Puddings or desserts	☐	☐	☐	☐	☐	☐
Biscuits, sweets, chocolate or cakes	☐	☐	☐	☐	☐	☐
Crisps and corn snacks	☐	☐	☐	☐	☐	☐
Follow on formula	☐	☐	☐	☐	☐	☐
Or something else (please tick and then write below)	☐	☐	☐	☐	☐	☐

1542

9. Do you ever add salt to your baby's solid food, including adding salt when the food is being cooked? *(Please tick (✓)* ***one*** *box).*

Yes, often	☐
Yes, sometimes	☐
Never	☐

For peer review only - http://bmjopen.bmj.com/site/about/guidelines.xhtml

Section 10: Mood

*(Please tick (✓) **one** box for each statement below).*

In the past 7 days…

**17.I have been able to laugh and see the funny side of things.**	
As much as I always could	☐
Not quite so much now	☐
Definitely not so much now	☐
Not at all	☐

**18.I have looked forward with enjoyment to things.**	
As much as I ever did	☐
Rather less than I used to	☐
Definitely less than I used to	☐
Hardly at all	☐

**19.I have blamed myself unnecessarily when things went wrong.**	
Yes, most of the time	☐
Yes, some of the time	☐
Not very often	☐
No, never	☐

**20.I have been anxious or worried for no good reason.**	
No, not at all	☐
Hardly ever	☐
Yes, sometimes	☐
Yes, very often	☐

**21.I have felt scared or panicky for no very good reason.**	
Yes, quite a lot	☐
Yes, sometimes	☐
No, not much	☐
No, not at all	☐

For peer review only

**22. Things have been getting on top of me.**	
Yes, most of the time I haven't been able to cope at all	☐
Yes, sometimes I haven't been coping as well as usual	☐
No, most of the time I have coped quite well	☐
No, I have been coping as well as ever	☐

**23. I have been so unhappy that I have had difficulty sleeping.**	
Yes, most of the time	☐
Yes, sometimes	☐
Not very often	☐
No, not at all	☐

**24. I have felt sad or miserable.**	
Yes, most of the time	☐
Yes, quite often	☐
Not very often	☐
No, not at all	☐

**9. I have been so unhappy that I have been crying.**	
Yes, most of the time	☐
Yes, quite often	☐
Only occasionally	☐
No, never	☐

**10. The thought of harming myself has occurred to me.**	
Yes, quite often	☐
Sometimes	☐
Hardly ever	☐
Never	☐

For peer review only - http://bmjopen.bmj.com/site/about/guidelines.xhtml

*(Please tick (✓) **one** box for each statement below).*

<u>Over the last two weeks</u>, how often have you been bothered by the following problems?

**26. Feeling nervous, anxious, or on edge?**	
Not at all	☐
Several days	☐
More than half of the days	☐
Nearly every day	☐

**27. Not being able to stop or control worrying?**	
Not at all	☐
Several days	☐
More than half of the days	☐
Nearly every day	☐

**28. Worrying too much about different things?**	
Not at all	☐
Several days	☐
More than half of the days	☐
Nearly every day	☐

**29. Trouble relaxing?**	
Not at all	☐
Several days	☐
More than half of the days	☐
Nearly every day	☐

**30. Being so restless that it is hard to sit still?**	
Not at all	☐
Several days	☐
More than half of the days	☐
Nearly every day	☐

For peer review only - http://bmjopen.bmj.com/site/about/guidelines.xhtml

For peer review only

**31. Becoming easily annoyed or irritable?**	
Not at all	☐
Several days	☐
More than half of the days	☐
Nearly every day	☐

**32. Feeling afraid as if something awful might happen?**	
Not at all	☐
Several days	☐
More than half of the days	☐
Nearly every day	☐

Having a young baby or child can bring up lots of different feelings and emotions. The next questions are designed to explore how you are feeling about being a parent to your baby/child. Answering these questions will help us to understand how things are going for you. If your most recent pregnancy was a twin/multiple pregnancy, please complete these questions for the youngest twin/multiple.

**33. Has your child reached their second birthday?** *(Please tick (✓)* ***one*** *box).*		
No, my child has not yet had their second birthday	☐	→ Please go to question 19 on page 37.
Yes, my child has celebrated their second birthday	☐	→ Please go to question 21 on Page 39

For peer review only

19. Thinking about your feelings about your baby, choose the response for each statement that feels right to you. *(Please tick (✓)* ***one*** *box for each of the following statements).*

	Never	Sometimes	Always
I enjoy looking after my baby	☐	☐	☐
I feel irritated with my baby when we are together	☐	☐	☐
I feel affectionate towards my baby	☐	☐	☐
I feel that my baby is being difficult or trying to upset me on purpose	☐	☐	☐
I can work out what my baby needs from me	☐	☐	☐
I feel like I can't do things I enjoy because of my baby.	☐	☐	☐
I feel the changes in my life are worth it to look after my baby.	☐	☐	☐
I miss my baby when we are not together	☐	☐	☐
I feel like I'm looking after my baby for someone else.	☐	☐	☐
When we've been apart I look forward to seeing my baby again.	☐	☐	☐
I enjoy playing with my baby.	☐	☐	☐

20. Thinking about your baby, please tick one of the choices for each of the questions below. There are no 'right' or 'wrong' answers; many of these are

For peer review only - http://bmjopen.bmj.com/site/about/guidelines.xhtml

true of all babies at times. *(Please tick (✓)* ***one*** *box for each of the following statements).*

	Always	Very often	Quite often	Sometimes	Rarely	Never
My baby smiles at me.	☐	☐	☐	☐	☐	☐
My baby annoys me.	☐	☐	☐	☐	☐	☐
My baby likes doing things with me.	☐	☐	☐	☐	☐	☐
My baby ‘talks’ to me.	☐	☐	☐	☐	☐	☐
My baby irritates me.	☐	☐	☐	☐	☐	☐
My baby likes me.	☐	☐	☐	☐	☐	☐
My baby wants too much attention.	☐	☐	☐	☐	☐	☐
My baby laughs.	☐	☐	☐	☐	☐	☐
My baby gets moody.	☐	☐	☐	☐	☐	☐
My baby dominates me.	☐	☐	☐	☐	☐	☐
My baby likes to please me.	☐	☐	☐	☐	☐	☐
My baby cries for no obvious reason.	☐	☐	☐	☐	☐	☐
My baby is affectionate towards me.	☐	☐	☐	☐	☐	☐
My baby winds me up.	☐	☐	☐	☐	☐	☐

Please go to section 11 on Page 41

For peer review only

For peer review only - http://bmjopen.bmj.com/site/about/guidelines.xhtml

21. Thinking about your feelings about your child, choose the response for each statement that feels right to you. *(Please tick (✓)* ***one*** *box for each of the following statements).*

	Never	Sometimes	Always
I enjoy looking after my child	☐	☐	☐
I feel irritated with my child when we are together	☐	☐	☐
I feel affectionate towards my child	☐	☐	☐
I feel that my child is being difficult or trying to upset me on purpose	☐	☐	☐
I can work out what my child needs from me	☐	☐	☐
I feel like I can't do things I enjoy because of my child.	☐	☐	☐
I feel the changes in my life are worth it to look after my child.	☐	☐	☐
I miss my child when we are not together	☐	☐	☐
I feel like I'm looking after my child for someone else.	☐	☐	☐
When we've been apart I look forward to seeing my child again.	☐	☐	☐
I enjoy playing with my child.	☐	☐	☐

22. Thinking about your child, please tick one of the choices for each of the questions below. There are no 'right' or 'wrong' answers; many of these are

For peer review only - http://bmjopen.bmj.com/site/about/guidelines.xhtml

true of all children at times. *(Please tick (✓)* ***one*** *box for each of the following statements).*

	Always	Very often	Quite often	Sometimes	Rarely	Never
My child smiles at me.	☐	☐	☐	☐	☐	☐
My child annoys me.	☐	☐	☐	☐	☐	☐
My child likes doing things with me.	☐	☐	☐	☐	☐	☐
My child talks to me.	☐	☐	☐	☐	☐	☐
My child irritates me.	☐	☐	☐	☐	☐	☐
My child likes me.	☐	☐	☐	☐	☐	☐
My child wants too much attention.	☐	☐	☐	☐	☐	☐
My child laughs.	☐	☐	☐	☐	☐	☐
My child gets moody.	☐	☐	☐	☐	☐	☐
My child dominates me.	☐	☐	☐	☐	☐	☐
My child likes to please me.	☐	☐	☐	☐	☐	☐
My child cries for no obvious reason.	☐	☐	☐	☐	☐	☐
My child is affectionate towards me.	☐	☐	☐	☐	☐	☐
My child winds me up.	☐	☐	☐	☐	☐	☐

1617

1618

For peer review only

Section 11: Self-esteem

Below is a list of statements dealing with your general feelings about yourself.

*(Please tick (✓) **one** box to indicate how much you agree or disagree with each of the following statements).*

	Strongly Agree	Agree	Disagree	Strongly disagree
I feel that I'm a person of worth, at least on an equal plane with others.	☐	☐	☐	☐
I feel that I have a number of good qualities.	☐	☐	☐	☐
All in all, I am inclined to feel that I am a failure.	☐	☐	☐	☐
I am able to do things as well as most other people.	☐	☐	☐	☐
I feel I do not have much to be proud of.	☐	☐	☐	☐
I take a positive attitude toward myself.	☐	☐	☐	☐
On the whole I am satisfied with myself.	☐	☐	☐	☐
I wish I could have more respect for myself.	☐	☐	☐	☐
I certainly feel useless at times.	☐	☐	☐	☐
At times I think I am no good at all.	☐	☐	☐	☐

For peer review only - http://bmjopen.bmj.com/site/about/guidelines.xhtml

Section 12: Sleep

1. At the moment, how often does your baby sleep all night? *(Please tick (✓) **one** box).*

Always	☐
Mostly	☐
Sometimes	☐
Rarely	☐
Never	☐

The following questions relate to **your** usual sleep habits during the **past month only**. Your answers should indicate the most accurate reply for the majority of days and nights in the past month. Please answer all questions.

2. During the past month, what time have you usually gone to bed at night?

Usual bed time ☐

3. During the past month, how long (in minutes) has it usually taken you to fall asleep each night?

Number of minutes ☐

4. During the past month, what has been your usual getting up time in the morning?

Usual getting up time ☐

5. During the past month, on average how many hours of actual sleep did you get at night? (This may be different than the number of hours you spent in bed.)

Hours of sleep per night ☐

For peer review only - http://bmjopen.bmj.com/site/about/guidelines.xhtml

6. During the past month, how often have you had trouble sleeping because you…

*(Please tick (✓) **one** box for each statement).*

	Not during the past month	Less than once a week	Once or twice a week	Three or more times a week
Cannot get to sleep within 30 minutes	☐	☐	☐	☐
Wake up in the middle of the night or early morning	☐	☐	☐	☐
Have to get up to use the bathroom	☐	☐	☐	☐
Cannot breathe comfortably	☐	☐	☐	☐
Cough or snore loudly	☐	☐	☐	☐
Feel too cold	☐	☐	☐	☐
Feel too hot	☐	☐	☐	☐
Had bad dreams	☐	☐	☐	☐
Have pain	☐	☐	☐	☐
Have to take care of your baby	☐	☐	☐	☐
Other reason(s), please describe and indicate how often during the past month you had trouble sleeping because of this:				
________	☐	☐	☐	☐
________	☐	☐	☐	☐
________	☐	☐	☐	☐

For peer review only - http://bmjopen.bmj.com/site/about/guidelines.xhtml

**7. During the past month, how would you rate your sleep quality overall?** *(Please tick (✓)* ***one*** *box).*			
**Very Good**	**Fairly Good**	**Fairly Bad**	**Very Bad**
☐	☐	☐	☐

**8**. (Please tick *(✓)* ***one*** *box for each question below.)*	**Not during the past month**	**Less than once a week**	**Once or twice a week**	**Three or more times a week**
**During the past month, how often have you taken medicine (prescribed or "over the counter") to help you sleep?**	☐	☐	☐	☐
**During the past month, how often have you had trouble staying awake while driving, eating meals, or engaging in social activity?**	☐	☐	☐	☐

**9. During the past month, how much of a problem has it been for you to keep up enough enthusiasm to get things done?** (Please tick *(✓)* ***one*** *box).*			
**No problem at all**	**Only a very slight problem**	**Somewhat of a problem**	**A very big problem**
☐	☐	☐	☐

**10. Do you usually have a bed partner or room-mate?** *(Please tick (✓)* ***one*** *box).*			
**No bed partner or room-mate**	**Partner/room-mate in other room**	**Partner in same room but not in same bed**	**Partner in same bed**
☐	☐	☐	☐

For peer review only - http://bmjopen.bmj.com/site/about/guidelines.xhtml

11. If you usually have a room-mate or bed partner, ask him/her how often in the past month you have had:

Please tick (✓) ***one*** *box for each statement).*	**Not during the past month**	**Less than once a week**	**Once or twice a week**	**Three or more times a week**
Loud snoring	☐	☐	☐	☐
Long pauses between breaths while asleep	☐	☐	☐	☐
Legs twitching or jerking while you sleep	☐	☐	☐	☐
Episodes of confusion during sleep	☐	☐	☐	☐
Other restlessness while you sleep, please describe and indicate how often in the past month you have had this:				
______________________	☐	☐	☐	☐
______________________	☐	☐	☐	☐
______________________	☐	☐	☐	☐

only

For peer review only - http://bmjopen.bmj.com/site/about/guidelines.xhtml

You have now completed the questionnaire.
Thank you very much for your time and effort!
We really appreciate your help with our research.

For peer review only - http://bmjopen.bmj.com/site/about/guidelines.xhtml

Supplemental appendix 4- Interview guides

SEMI-STRUCTURED INTERVIEW TOPIC GUIDE

6 months – Intervention Group

The facilitator will introduce him/herself and reiterate the purpose of the interview i.e. to gather thoughts and opinions on the Supporting MumS study.

The facilitator will emphasise that although the interview will be audio-recorded for research purposes, all information provided by the respondent will be kept anonymous (i.e. their name will never be disclosed). The approximate time of the interview (20-30 minutes) will be stated. Individuals will also be informed that if at any stage they wish to discontinue with the interview they are free to do so. In addition, it will be made clear to respondents that they can inform the facilitator if there are any particular statements they do not wish to be transcribed at the end of the session.

Before commencing, participants will be asked to verbally confirm consent as below:

- Do you agree to the interview being audio-recorded?
- Do you agree that anonymised quotations from these discussions can be used in research publications?

The interview will only proceed if the participant answers yes to the statements above.

____

Topic Guide:

Opinions on the SMS study

- Why did you sign up to take part in the SMS study? *(Prompt: What were your motivations for signing up to take part?)*
- What do you think of the information you received at the beginning of the study? (i.e. the poster, information sheet, flyer, info from researchers via telephone etc.)
- What do you think of the SMS study so far?
- Did you have a preference for which group you would be assigned to at the start (intervention/control – remind participants of the groups)?

Opinions on the text messages

For peer review only

For peer review only - http://bmjopen.bmj.com/site/about/guidelines.xhtml

- Overall, how have you found the text messages you have received so far? (*Prompt: content, tone, length, clarity, relevance)*
- Have you been receiving any of the messages related specifically to breastfeeding or smoking? *(Prompt: If yes, were they useful?)*
- What type of text messages do you like the most/least? *(Prompt: Why?)*
- What do you think of the messages that prompt you to check your weight each week? *(Probe: to what extent do you engage with these messages? Prompt: do you reply to these messages? Why?)*
- What do you think of the messages that prompt you to set goals and review your goals each week? *(Probe: to what extent do you engage with these messages? Prompt: do you reply to these messages? Why?)*
- When you get a text message, how likely are you to read it?
- Do you ever go back and re-read messages? *(Prompt: Do you keep the messages or delete them?)*
- Have you used any of the trigger words?
- Do you click on the links in the messages to websites? *If not, why not?*
- What did you think about the frequency of text messages (i.e. how often you are receiving them)?
- Could the text messages you have received so far have been improved in any way? If so, how?
- At any stage, did you notice or were you informed of an issue with the text messages? E.g. text about a glitch in the system, repeated messages? *Probe: what did this make you think about the messages? Did it impact how you engage with the messages?*
- *Mothers of twins/multiples only:* What do you think of the suitability of the messages for women with twins or multiples?
- *Mothers of twins/multiples only:* How do feel having twins/multiples has impacted your receipt of the intervention? *(Prompts: in what ways has it impacted your ability to make changes to your eating habits or physical activity?)*

<u>Behaviour change</u>

- How do you feel your eating habits or physical activity have changed as a result of the text messages? (*Prompts: in what way? What are the main changes you have made)*
- Have there been any negatives about signing up for the messages?

For peer review only

- What do you think are the main benefits, if any, you have gained so far from signing up for the messages?
- Do you think receiving the messages has had any wider impacts or benefits for your family?

Social support

- You were given the opportunity to have a friend or family member also receive the messages. Have you done this so far? *(Prompts: If so how are you finding it? If not, why not?)*
- To what extent do you feel your friends and family supported you to make changes to your eating habits or physical activity?

Postnatal mental health

- How do you feel your mental health has been since having your baby?
- How do you feel your mental health has impacted your ability to follow the weight management advice that you received?
- In what ways has receiving the intervention impacted your mental health, if at all?
- *For women who discuss experiencing postnatal mental health issues:* what support services have you accessed to help with your mental health since having a baby? *(Prompts: to what extent did this help you? What further support do you feel you would have benefitted from?)*

Data collection

- How did you find the study visits (i.e. meeting with a researcher) so far?
- What did you think of having the option of being able to meet a researcher at your home instead of travelling somewhere for the visit?
- What do you think of the questionnaires you have had to complete? (*Prompts: what did you think of the length? How easy were they to understand?)*
- Did you receive a voucher following your study visit? What did you think of this part of the study i.e. receiving vouchers? *Prompts: Did it influence your decision to sign-up to the study? Why do you think we gave vouchers to participants taking part?)*

INTERVIEW ENDS

For peer review only

For peer review only - http://bmjopen.bmj.com/site/about/guidelines.xhtml

SEMI-STRUCTURED INTERVIEW TOPIC GUIDE

6 months – Control Group

The facilitator will introduce him/herself and reiterate the purpose of the interview i.e. to gather thoughts and opinions on the Supporting MumS study.

The facilitator will emphasise that although the interview will be audio-recorded for research purposes, all information provided by the respondent will be kept anonymous (i.e. their name will never be disclosed). The approximate time of the interview (20-30 minutes) will be stated. Individuals will also be informed that if at any stage they wish to discontinue with the interview they are free to do so. In addition, it will be made clear to respondents that they can inform the facilitator if there are any particular statements they do not wish to be transcribed at the end of the session.

Before commencing, participants will be asked to verbally confirm consent as below:

- Do you agree to the interview being audio-recorded?
- Do you agree that anonymised quotations from these discussions can be used in research publications?

The interview will only proceed if the participant answers yes to the statements above.

Topic Guide:

Opinions on the SMS study

- Why did you sign up to take part in the SMS study? *(Prompt: what were your motivations for signing up to take part?)*
- What do you think of the information you received at the beginning of the study? (i.e. the poster, information sheet, flyer, info from researchers via telephone etc.)
- What do you think of the SMS study so far?
- Did you have a preference for which group you were assigned to at the start (intervention/control – remind participants of the groups)?

Opinions on the text messages

- Overall, how have you found the text messages you have received so far? (*Prompt: content, tone, length, clarity, relevance)*

For peer review only

For peer review only - http://bmjopen.bmj.com/site/about/guidelines.xhtml

- What type of text messages do you like the most/least? *(Prompts: e.g. developmental messages; play ideas; Why do you like these the most/least?)*
- When you receive a text message, how likely are you to read it?
- Do you click on the links in the messages to websites? *(Prompt: If not, why not?)*
- What do you think about the frequency of text messages (i.e. how often you are receiving them)?
- Could the text messages you have received so far have been improved in any way? *(Prompt: If so, how?)*
- *Mothers of twins/multiples only:* What do you think of the suitability of the messages for women with twins or multiples?

<u>Impact of study</u>

- Have there been any negatives about signing up for the messages?
- What do you think are the main benefits, if any, you have gained so far from signing up for the messages?
- Do you think receiving the messages has had any wider impacts or benefits for your family?

<u>Postnatal mental health</u>

- How do you feel your mental health has been since having your baby?
- *For women who discuss experiencing postnatal mental health issues:* what support services have you accessed to help with your mental health since having a baby? *(Prompts: to what extent did this help you? What further support do you feel you would have benefitted from?)*

<u>Data collection</u>

- How have you found the study visits (i.e. meeting with a researcher) so far?
- What did you think of having the option of being able to meet a researcher at your home instead of travelling somewhere for the visit?
- What do you think of the questionnaires you have had to complete? (*Prompts: what did you think of the length? How easy were they to understand?)*
- Did you receive a voucher following your study visit? *(Prompts: What did you think of this part of the study i.e. receiving vouchers? Did it influence your decision to sign-up to the study? Why do you think we gave vouchers to participants taking part?)*

INTERVIEW ENDS

For peer review only

SEMI-STRUCTURED INTERVIEW TOPIC GUIDE

12 months – Intervention Group

The facilitator will introduce him/herself and reiterate the purpose of the interview i.e. to gather thoughts and opinions on the Supporting MumS study.

The facilitator will emphasise that although the interview will be audio-recorded for research purposes, all information provided by the respondent will be kept anonymous (i.e. their name will never be disclosed). The approximate time of the interview (20-30 minutes) will be stated. Individuals will also be informed that if at any stage they wish to discontinue with the interview they are free to do so. In addition, it will be made clear to respondents that they can inform the facilitator if there are any particular statements they do not wish to be transcribed at the end of the session.

Before commencing, participants will be asked to verbally confirm consent as below:

- Do you agree to the interview being audio-recorded?
- Do you agree that anonymised quotations from these discussions can be used in research publications?

The interview will only proceed if the participant answers yes to the statements above.

Topic Guide:

<u>Opinions on the SMS study</u>

- What did you think of the SMS study?
- What did you think of the information you received at the beginning of the study?
- Did you have a preference for which group you were assigned to at the start (intervention/control – remind participants of the groups)?

<u>Opinions on the text messages</u>

- Overall, did you like the messages you received during the SMS study? (*Prompt: tone, length, clarity, relevance)*
- Did you receive any of the messages related specifically to breastfeeding or smoking? *(Prompt: If yes, were they useful?)*
- Overall, did you find the text messages useful or not useful?
- What type of text message did you like the most/least? *(Prompt: Why did you like these the most/least?)*

For peer review only

For peer review only - http://bmjopen.bmj.com/site/about/guidelines.xhtml

- What do you think of the messages that prompt you to check your weight each week? *(Probe: to what extent do you engage with these messages? Prompt: do you reply to these messages? Why?)*
- What do you think of the messages that prompt you to set goals and review your goals each week? *(Probe: to what extent do you engage with these messages? Prompt: do you reply to these messages? Why?)*
- When you got a text message, how likely were you to read it?
- Do you ever go back and re-read messages? *(Prompt: Do you keep the messages or delete them?)*
- Have you used any trigger words (i.e. tired, bad day, crave, slip-up)?
- What do you think about the frequency of text messages (i.e. how often you received them)?
- What do you think of the texts that had links to the SMS website? (*Prompt: did you click on the links?)*
- Could the text messages you received have been improved in any way? *(Prompt: If so, how?)*
- Women were able to join the Supporting MumS study up to two years after having a baby. How useful do you think that is? *(Prompt: do you think there is a best time for receiving the text messages? How ready were you to engage with the advice?)*
- *Mothers of twins/multiples only:* What do you think of the suitability of the messages for women with twins or multiples?
- *Mothers of twins/multiples only:* How do feel having twins/multiples has impacted your receipt of the intervention? *(Prompt: in what ways has it impacted your ability to make changes to your eating habits or physical activity?).*

<u>Behaviour change</u>

- Have you changed your eating habits or physical activity as a result of the text messages? *(Prompts: In what way? What are the main changes you have made?)*
- Have there been any negatives about signing up for the messages?
- What do you think are the main benefits, if any, you gained from signing up for the messages?
- Do you think receiving the messages has had any wider impacts or benefits for your family?
- Do you think you will continue with the changes you have made now that the study has finished?

For peer review only

For peer review only - http://bmjopen.bmj.com/site/about/guidelines.xhtml

Social support

- You were given the opportunity to have a friend or family member also receive the messages, did you do this? *(Prompt: If not, why not?)*
- To what extent do you feel your friends and family supported you to make changes to your eating habits or physical activity?

Postnatal mental health

- How do you feel your mental health has been since having your baby?
- How do you feel your mental health has impacted your ability to follow the weight management advice that you received?
- In what ways has receiving the intervention impacted your mental health, if at all?
- *For women who discuss experiencing postnatal mental health issues:* what support services have you accessed to help with your mental health since having a baby? *(Prompt: to what extent did this help you? What further support do you feel you would have benefitted from?)*

Data collection

- What did you think about having your measurements taken? (Prompts: How did you feel about being weighed? How did you feel about having your waist circumference measured? Is there anything that could have been done better in taking these measurements?)
- What do you think of the questionnaires you have had to complete during the study visits? (*Prompts: what did you think of the length? How easy were they to understand?)*
- What did you think of having the option of being able to meet a researcher at your home instead of travelling somewhere for the visit?

Overall evaluation

- What was the best thing about the SMS study?
- What was the worst thing about the SMS study?

For peer review only

- Is there anything we could have done that would have made the experience better? (*Prompts: is there anything you feel should be added to the intervention? Is there any part of the study you would recommend changing?)*
- Would you take part in the SMS study again if you had the chance?
- Would you recommend the SMS study to new mums?

INTERVIEW ENDS

For peer review only

For peer review only - http://bmjopen.bmj.com/site/about/guidelines.xhtml

SEMI-STRUCTURED INTERVIEW TOPIC GUIDE

12 months – Control Group

The facilitator will introduce him/herself and reiterate the purpose of the interview i.e. to gather thoughts and opinions on the Supporting MumS study.

The facilitator will emphasise that although the interview will be audio-recorded for research purposes, all information provided by the respondent will be kept anonymous (i.e. their name will never be disclosed). The approximate time of the interview (20 minutes) will be stated. Individuals will also be informed that if at any stage they wish to discontinue with the interview they are free to do so. In addition, it will be made clear to respondents that they can inform the facilitator if there are any particular statements they do not wish to be transcribed at the end of the session.

Before commencing, participants will be asked to verbally confirm consent as below:

- Do you agree to the interview being audio-recorded?
- Do you agree that anonymised quotations from these discussions can be used in research publications?

The interview will only proceed if the participant answers yes to the statements above.

Topic Guide:

Opinions on the SMS study

- What did you think of the SMS study?
- What did you think of the information you received at the beginning of the study?
- Did you have a preference for which group you would be assigned to at the start (intervention/control – remind participants of the groups)?

Opinions on the text messages

- Overall, did you like the messages you received during the SMS study? *(Prompt: tone, length, clarity, relevance)*
- Did you find the text messages useful?
- Which type of text messages did you like the most/least? *(Prompt: Why did you like these the most/least?)*
- When you received a text message, how likely were you to read the message?
- What did you think of the texts that had links to the SMS website? (*Prompt: did you click on the links?)*
- What did you think about the frequency of text messages (i.e. how often you received them)?
- Could the text messages have been improved? *(Prompt: If so, how?)*

For peer review only

For peer review only - http://bmjopen.bmj.com/site/about/guidelines.xhtml

- *Mothers of twins/multiples only:* What do you think of the suitability of the messages for women with twins or multiples?

Impact of the study

- Have there been any negatives about signing up for the messages?
- What do you think are the main benefits, if any, you have gained from signing up for the messages?
- Do you think receiving the messages has had any wider impacts or benefits for your family?

Postnatal mental health

- How do you feel your mental health has been since having your baby?
- *For women who discuss experiencing postnatal mental health issues:* what support services have you accessed to help with your mental health since having a baby? *(Prompts: to what extent did this help you? What further support do you feel you would have benefitted from?)*

Data collection

- What did you think about having your measurements taken? (Prompts: How did you feel about being weighed? How did you feel about having your waist circumference measured? Is there anything that could have been done better in taking these measurements?)
- What did you think of the questionnaires you had to complete during the study visits? (*Prompts: what did you think of the length? How easy were they to understand?*
- What did you think of having the option of being able to meet a researcher at your home instead of travelling somewhere for the visit?

Overall evaluation

- What was the best thing about the SMS study?
- What was the worst thing about the SMS study?
- Is there anything we could have done that would have made the experience better? *(Prompts: is there anything you feel could be added to the text messages? Is there any part of the study you would recommend changing?)*
- Would you take part in the SMS study again if you had the chance?
- Would you recommend the SMS study to new mums?

INTERVIEW ENDS

For peer review only

For peer review only - http://bmjopen.bmj.com/site/about/guidelines.xhtml

2024 **Supplemental appendix 5- World Health Organization Trial Registration Data Set**

**Data Category**	**Information**
Primary Registry and Trial Identifying Number	ISRCTN Registry; ISRCTN16299220
Date of Registration in Primary Registry	10/02/2022
Secondary Identifying Numbers	FUNDERS Number: NIHR131509<br>Sponsor Number: B21/24<br>IRAS Number: 305557<br>REC Reference: 22/WS/0003
Source(s) of Monetary or Material Support	National Institute for Health and Care Research (NIHR) Public Health Research (PHR). Intervention costs provided by the Public Health Agency (Northern Ireland).
Primary Sponsor	Queen's University Belfast
Secondary Sponsor(s)	None
Contact for Public Queries	Dr Dunla Gallagher<br>SMS Trial Manager<br>d.gallagher@qub.ac.uk<br>07341 888415<br>Centre for Public Health<br>Queen's University Belfast<br>Institute of Clinical Sciences B<br>Grosvenor Road<br>Belfast<br>BT12 6BJ
Contact for Scientific Queries	Professor Michelle McKinley<br>Chief Investigator<br>m.mckinley@qub.ac.uk<br>028 9097 8936<br>Centre for Public Health<br>Queen's University Belfast<br>Institute of Clinical Sciences A<br>Grosvenor Road<br>Belfast<br>BT12 6BA

For peer review only

For peer review only - http://bmjopen.bmj.com/site/about/guidelines.xhtml

Public Title	The Supporting MumS (SMS) study: can we use an automated text message intervention for weight management in women after childbirth?
Scientific Title	Effectiveness and cost effectiveness of an automated text message intervention for weight management in postpartum women with overweight or obesity: the Supporting MumS (SMS) Randomised Controlled Trial.
Countries of Recruitment	United Kingdom (Northern Ireland, Scotland, England and Wales).
Health Condition(s) or Problem(s) Studied	Overweight and obesity in postpartum women.
Intervention(s)	Supporting MumS intervention group- will receive automated text messages about weight loss and maintenance of weight loss for 12 months. The text messages will focus on diet and physical activity with embedded behaviour change techniques known to be positively associated with weight management.<br><br>Active control group- will receive automated text messages about child health and development for 12 months.
Key Inclusion and Exclusion Criteria	Inclusion criteria<br>• Women (as per NICE Postnatal care guideline NG194[1], the term 'woman' is taken to include people who do not identify as women but who are pregnant or have given birth)<br>• Aged > 18 years old<br>• BMI ≥25 kg/m^2<br>• Have had a baby within the last two years<br><br>Exclusion criteria<br>• Baby less than 6 weeks old<br>• No access to a mobile phone to receive personal messages<br>• Insufficient English to understand short written messages<br>• Currently pregnant<br>• Recent or planned bariatric surgery<br>• Previous diagnosis of anorexia or bulimia by a doctor

For peer review only - http://bmjopen.bmj.com/site/about/guidelines.xhtml

	• On a specialist diet and receiving dietetic care<br>• Taking part in another weight management research study currently, or in the last 3 months
Study Type	Two-arm parallel groups randomised controlled trial to test the effectiveness and cost-effectiveness of a 12-month text-messaging behavioural weight management intervention in supporting weight loss for women with overweight and obesity in the postpartum period, compared with an active control group receiving text messages related to child health and development for 12 months.<br><br>Women will be recruited from all four countries in the UK using community-based recruitment as well as signposting via routine contact with health professionals.<br><br>Written informed consent will be obtained, baseline data collected and then participants will be randomised. Participants will be block randomised and randomisation will be stratified by site. The randomisation sequence will be developed in STATA by a statistician who is independent of the study team. The randomisation will be implemented via the London School of Hygiene and Tropical Medicine secure remote web-based system which will link directly with the text message database and will deliver the intervention or active control content according to the random allocation sequence.<br><br>Participants will become aware of their group allocation when they start to receive the messages. Researchers who are recruiting women to the study and collecting outcome data are blinded to group allocation.
Date of First Enrolment	Actual date of enrolment of the first participant: 20/05/2022
Sample Size	Target sample size: 888<br>Final number of participants that the trial enrolled: 892
Recruitment Status	Complete: participants are no longer being recruited or enrolled.

For peer review only - http://bmjopen.bmj.com/site/about/guidelines.xhtml

Primary Outcome	Weight change from baseline to 12 months (kg), measured using calibrated scales.
Secondary Outcome	1. Waist circumference measured by a flexible measuring tape at baseline, 6, 12 and 24 months, and mean BMI (kg/m^2) and the proportions of women gaining a substantial amount of weight (>5kg) at 12 and 24 months.<br><br>2. Health behaviours are measured by: Fat and Fibre Barometer; self-report questionnaire on sugar and alcohol intake; International Physical Activity Questionnaire (IPAQ) - Short form; and self-report questionnaire on infant feeding practices at baseline, 6, 12 and 24 months.<br><br>3. Study acceptability is measured by: recruitment; retention; engagement with the two-way text messages over 12 months; self-reported questionnaire on participant satisfaction with SMS messages (at 6 and 12 months) and rating of intervention (at 12 months); and qualitative interviews (at 6 and 12 months).<br><br>4. Economic evaluation outcomes are measured by: self-report questionnaire on health service resources use, medication usage and lifestyle-related costs; the EuroQol 5-dimension (EQ-5D) quality of life questionnaire; and the ICEpop Capability Measure for Adults (ICECAP-A) at baseline, 6, 12 and 24 months.<br><br>5. Moderators of intervention effect are measured by: Edinburgh Postnatal Depression Scale (EPDS); Generalised Anxiety Disorder (GAD-7); Pittsburgh Sleep Quality Index; and self-report questionnaire on confidence and desire for weight loss and maintenance at baseline, 6, 12 and 24 months.<br><br>6. Mediators of intervention effect are measured by: Health Action Process Approach (HAPA) for diet and exercise; Self-Report Behavioural Automaticity Index for diet and exercise; Self-regulation of eating behaviour questionnaire; self-report questionnaire on monitoring and goal setting for diet and

For peer review only - http://bmjopen.bmj.com/site/about/guidelines.xhtml

	exercise; Motivation for weight loss scale; Social support for eating & exercise questionnaire; and Rosenberg Self-Esteem Scale at baseline, 6, 12 and 24 months.
Ethics Review	Approved 24/02/2022 (Ref: 22/WS/0003) by West of Scotland REC 4 (West of Scotland Research Ethics Service, Ward 11, Dykebar Hospital, Grahamston Road, Paisley, PA2 7DE, United Kingdom; +44 (0)141 3140213; WoSREC4@ggc.scot.nhs.uk).
Completion date	30/04/2025
Summary results	Intention to publish date: 30/04/2026.
IPD Sharing Statement	Following publication of the results, the anonymised participant-level datasets generated during and/or analysed during the current study, along with the statistical code used for generating the results, will be available upon request from the Chief Investigator on reasonable request, as assessed by the Chief Investigator and Project Management Team and subject to any necessary data sharing agreements. Formal requests to be made in writing to the Chief Investigator (Prof M McKinley; m.mckinley@qub.ac.uk). Data availability will be consistent with timelines for storage of research data.

2025

2026

For peer review only

For peer review only - http://bmjopen.bmj.com/site/about/guidelines.xhtml

SPIRIT checklist

SPIRIT 2013 Checklist: Recommended items to address in a clinical trial protocol and related documents*

Section/item	Item No	Description	Addressed on page number
**Administrative information**			
Title	1	Descriptive title identifying the study design, population, interventions, and, if applicable, trial acronym	4
Trial registration	2a	Trial identifier and registry name. If not yet registered, name of intended registry	2
	2b	All items from the World Health Organization Trial Registration Data Set	Supplemental appendix 5
Protocol version	3	Date and version identifier	4
Funding	4	Sources and types of financial, material, and other support	49
Roles and responsibilities	5a	Names, affiliations, and roles of protocol contributors	4-7, 48-49
	5b	Name and contact information for the trial sponsor	7
	5c	Role of study sponsor and funders, if any, in study design; collection, management, analysis, and interpretation of data; writing of the report; and the decision to submit the report for publication, including whether they will have ultimate authority over any of these activities	7
	5d	Composition, roles, and responsibilities of the coordinating centre, steering committee, endpoint adjudication committee, data management team, and other individuals or groups overseeing the trial, if applicable (see Item 21a for data monitoring committee)	39
**Introduction**			
Background and rationale	6a	Description of research question and justification for undertaking the trial, including summary of relevant studies (published and unpublished) examining benefits and harms for each intervention	8-12
	6b	Explanation for choice of comparators	12-13
Objectives	7	Specific objectives or hypotheses	13-14
Trial design	8	Description of trial design including type of trial (eg, parallel group, crossover, factorial, single group), allocation ratio, and framework (eg, superiority, equivalence, noninferiority, exploratory)	14

For peer review only

For peer review only - http://bmjopen.bmj.com/site/about/guidelines.xhtml

Methods: Participants, interventions, and outcomes

Study setting	9	Description of study settings (eg, community clinic, academic hospital) and list of countries where data will be collected. Reference to where list of study sites can be obtained	14
Eligibility criteria	10	Inclusion and exclusion criteria for participants. If applicable, eligibility criteria for study centres and individuals who will perform the interventions (eg, surgeons, psychotherapists)	14-15, 25-26
Interventions	11a	Interventions for each group with sufficient detail to allow replication, including how and when they will be administered	15-19
	11b	Criteria for discontinuing or modifying allocated interventions for a given trial participant (eg, drug dose change in response to harms, participant request, or improving/worsening disease)	19
	11c	Strategies to improve adherence to intervention protocols, and any procedures for monitoring adherence (eg, drug tablet return, laboratory tests)	19
	11d	Relevant concomitant care and interventions that are permitted or prohibited during the trial	19-20
Outcomes	12	Primary, secondary, and other outcomes, including the specific measurement variable (eg, systolic blood pressure), analysis metric (eg, change from baseline, final value, time to event), method of aggregation (eg, median, proportion), and time point for each outcome. Explanation of the clinical relevance of chosen efficacy and harm outcomes is strongly recommended	20-23
Participant timeline	13	Time schedule of enrolment, interventions (including any run-ins and washouts), assessments, and visits for participants. A schematic diagram is highly recommended (see Figure)	23 and Figure 1
Sample size	14	Estimated number of participants needed to achieve study objectives and how it was determined, including clinical and statistical assumptions supporting any sample size calculations	23-24
Recruitment	15	Strategies for achieving adequate participant enrolment to reach target sample size	24-25

Methods: Assignment of interventions (for controlled trials)

Allocation:

Sequence generation	16a	Method of generating the allocation sequence (eg, computer-generated random numbers), and list of any factors for stratification. To reduce predictability of a random sequence, details of any planned restriction (eg, blocking) should be provided in a separate document that is unavailable to those who enrol participants or assign interventions	27
Allocation concealment mechanism	16b	Mechanism of implementing the allocation sequence (eg, central telephone; sequentially numbered, opaque, sealed envelopes), describing any steps to conceal the sequence until interventions are assigned	27-28
Implementation	16c	Who will generate the allocation sequence, who will enrol participants, and who will assign participants to interventions	27-28
Blinding (masking)	17a	Who will be blinded after assignment to interventions (eg, trial participants, care providers, outcome assessors, data analysts), and how	28
	17b	If blinded, circumstances under which unblinding is permissible, and procedure for revealing a participant's allocated intervention during the trial	28
**Methods: Data collection, management, and analysis**			
Data collection methods	18a	Plans for assessment and collection of outcome, baseline, and other trial data, including any related processes to promote data quality (eg, duplicate measurements, training of assessors) and a description of study instruments (eg, questionnaires, laboratory tests) along with their reliability and validity, if known. Reference to where data collection forms can be found, if not in the protocol	29-30
	18b	Plans to promote participant retention and complete follow-up, including list of any outcome data to be collected for participants who discontinue or deviate from intervention protocols	30-31
Data management	19	Plans for data entry, coding, security, and storage, including any related processes to promote data quality (eg, double data entry; range checks for data values). Reference to where details of data management procedures can be found, if not in the protocol	31-32
Statistical methods	20a	Statistical methods for analysing primary and secondary outcomes. Reference to where other details of the statistical analysis plan can be found, if not in the protocol	35-36
	20b	Methods for any additional analyses (eg, subgroup and adjusted analyses)	36

For peer review only - http://bmjopen.bmj.com/site/about/guidelines.xhtml

	20c	Definition of analysis population relating to protocol non-adherence (eg, as randomised analysis), and any statistical methods to handle missing data (eg, multiple imputation)	36
**Methods: Monitoring**			
Data monitoring	21a	Composition of data monitoring committee (DMC); summary of its role and reporting structure; statement of whether it is independent from the sponsor and competing interests; and reference to where further details about its charter can be found, if not in the protocol. Alternatively, an explanation of why a DMC is not needed	39
	21b	Description of any interim analyses and stopping guidelines, including who will have access to these interim results and make the final decision to terminate the trial	37
Harms	22	Plans for collecting, assessing, reporting, and managing solicited and spontaneously reported adverse events and other unintended effects of trial interventions or trial conduct	40
Auditing	23	Frequency and procedures for auditing trial conduct, if any, and whether the process will be independent from investigators and the sponsor	38
**Ethics and dissemination**			
Research ethics approval	24	Plans for seeking research ethics committee/institutional review board (REC/IRB) approval	2, 43
Protocol amendments	25	Plans for communicating important protocol modifications (eg, changes to eligibility criteria, outcomes, analyses) to relevant parties (eg, investigators, REC/IRBs, trial participants, trial registries, journals, regulators)	41-42
Consent or assent	26a	Who will obtain informed consent or assent from potential trial participants or authorised surrogates, and how (see Item 32)	26-27
	26b	Additional consent provisions for collection and use of participant data and biological specimens in ancillary studies, if applicable	27
Confidentiality	27	How personal information about potential and enrolled participants will be collected, shared, and maintained in order to protect confidentiality before, during, and after the trial	33
Declaration of interests	28	Financial and other competing interests for principal investigators for the overall trial and each study site	49
Access to data	29	Statement of who will have access to the final trial dataset, and disclosure of contractual agreements that limit such access for investigators	33-34

For peer review only - http://bmjopen.bmj.com/site/about/guidelines.xhtml

Ancillary and post-trial care	30	Provisions, if any, for ancillary and post-trial care, and for compensation to those who suffer harm from trial participation	40
Dissemination policy	31a	Plans for investigators and sponsor to communicate trial results to participants, healthcare professionals, the public, and other relevant groups (eg, via publication, reporting in results databases, or other data sharing arrangements), including any publication restrictions	43-44
	31b	Authorship eligibility guidelines and any intended use of professional writers	48-49
	31c	Plans, if any, for granting public access to the full protocol, participant-level dataset, and statistical code	38-39
**Appendices**			
Informed consent materials	32	Model consent form and other related documentation given to participants and authorised surrogates	Supplemental appendix 2
Biological specimens	33	Plans for collection, laboratory evaluation, and storage of biological specimens for genetic or molecular analysis in the current trial and for future use in ancillary studies, if applicable	N/A

4

For peer review only

For peer review only - http://bmjopen.bmj.com/site/about/guidelines.xhtml

1 **TIDieR checklist**

Item number	Item	Where located ** Primary paper (page or appendix number)	Other † (details)
	**BRIEF NAME**		
1.	Provide the name or a phrase that describes the intervention.	15	
	**WHY**		
2.	Describe any rationale, theory, or goal of the elements essential to the intervention.	15-16 and Supplemental appendix 1	https://doi.org/10.3310/phr08040 (28)
	**WHAT**		
3.	Materials: Describe any physical or informational materials used in the intervention, including those provided to participants or used in intervention delivery or in training of intervention providers. Provide information on where the materials can be accessed (e.g. online appendix, URL).	16-17 and Supplemental appendix 1	______________
4.	Procedures: Describe each of the procedures, activities, and/or processes used in the intervention, including any enabling or support activities.	16-17	______________
	**WHO PROVIDED**		
5.	For each category of intervention provider (e.g. psychologist, nursing assistant), describe their expertise, background and any specific training given.	17	______________
	**HOW**		
6.	Describe the modes of delivery (e.g. face-to-face or by some other mechanism, such as internet or telephone) of the intervention and whether it was provided individually or in a group.	17	______________
	**WHERE**		
7.	Describe the type(s) of location(s) where the intervention occurred, including any necessary infrastructure or relevant features.	17	______________
	**WHEN and HOW MUCH**		
8.	Describe the number of times the intervention was delivered and over what period of time including the number of sessions, their schedule, and their duration, intensity or dose.	18	https://doi.org/10.3310/phr08040 (28)

For peer review only

	**TAILORING**		
**9.**	If the intervention was planned to be personalised, titrated or adapted, then describe what, why, when, and how.	15-18	______________
	**MODIFICATIONS**		
**10.‡**	If the intervention was modified during the course of the study, describe the changes (what, why, when, and how).	N/A	______________
	**HOW WELL**		
**11.**	Planned: If intervention adherence or fidelity was assessed, describe how and by whom, and if any strategies were used to maintain or improve fidelity, describe them.	19	______________
**12.‡**	Actual: If intervention adherence or fidelity was assessed, describe the extent to which the intervention was delivered as planned.	N/A	______________

** **Authors** - use N/A if an item is not applicable for the intervention being described. **Reviewers** – use '?' if information about the element is not reported/not sufficiently reported.

† If the information is not provided in the primary paper, give details of where this information is available. This may include locations such as a published protocol or other published papers (provide citation details) or a website (provide the URL).

‡ If completing the TIDieR checklist for a protocol, these items are not relevant to the protocol and cannot be described until the study is complete.

For peer review only
