# Supplementary material for: Effectiveness and cost-effectiveness of a 12-month automated text message intervention for weight management in postpartum women with overweight or obesity: protocol for the Supporting MumS (SMS) multisite, parallel-group, randomised controlled trial
Source: BMJ Open. 2024 May 6;14(5):e084075. doi: 10.1136/bmjopen-2024-084075 (PMC11086389; doi:10.1136/bmjopen-2024-084075)
Supplement: Reviewer comments [file bmjopen-2024-084075.reviewer_comments.pdf]

PEER REVIEW HISTORY

BMJ Open publishes all reviews undertaken for accepted manuscripts. Reviewers are asked to complete a checklist review form (**http://bmjopen.bmj.com/site/about/resources/checklist.pdf**) and are provided with free text boxes to elaborate on their assessment. These free text comments are reproduced below.

ARTICLE DETAILS

**TITLE (PROVISIONAL)**	Effectiveness and cost effectiveness of a 12 month automated text message intervention for weight management in postpartum women with overweight or obesity: protocol for the Supporting MumS (SMS) multi-site, parallel-group, randomised controlled trial.
**AUTHORS**	Gallagher, Dunla; Spyreli, Eleni; Anderson, Annie; Bridges, Sally; Cardwell, Christopher; Coulman, Elinor; Dombrowski, Stephan; Free, Caroline; Heaney, Suzie; Hoddinott, Pat; Kee, Frank; McDowell, Clíona; McIntosh, Emma; Woodside, Jayne; McKinley, Michelle C.

This article was not externally reviewed at BMJ Open. Protocols that have been independently assessed prior to submission to BMJ Open are usually fast-tracked to publication on the grounds that further substantial changes will not be possible. This independent assessment will usually be external, independent review for both a substantial grant award from a non-commercial or government funder and ethics approval.
